# Validating the knowledge bank approach for personalized prediction of survival in acute myeloid leukemia: a reproducibility study

**DOI:** 10.1007/s00439-022-02455-8

**Published:** 2022-04-16

**Authors:** Yujun Xu, Ulrich Mansmann

**Affiliations:** Institute for Medical Information Processing, Biometry, and Epidemiology, Ludwig-Maximilians-Universität München, Marchioninistr. 15, 81377 Munich, Germany

## Abstract

Reproducibility is not only essential for the integrity of scientific research but is also a prerequisite for model validation and refinement for the future application of predictive algorithms. However, reproducible research is becoming increasingly challenging, particularly in high-dimensional genomic data analyses with complex statistical or algorithmic techniques. Given that there are no mandatory requirements in most biomedical and statistical journals to provide the original data, analytical source code, or other relevant materials for publication, accessibility to these supplements naturally suggests a greater credibility of the published work. In this study, we performed a reproducibility assessment of the notable paper by Gerstung et al. (Nat Genet 49:332–340, 2017) by rerunning the analysis using their original code and data, which are publicly accessible. Despite an open science setting, it was challenging to reproduce the entire research project; reasons included: incomplete data and documentation, suboptimal code readability, coding errors, limited portability of intensive computing performed on a specific platform, and an R computing environment that could no longer be re-established. We learn that the availability of code and data does not guarantee transparency and reproducibility of a study; paradoxically, the source code is still liable to error and obsolescence, essentially due to methodological and computational complexity, a lack of reproducibility checking at submission, and updates for software and operating environment. The complex code may also hide problematic methodological aspects of the proposed research. Building on the experience gained, we discuss the best programming and software engineering practices that could have been employed to improve reproducibility, and propose practical criteria for the conduct and reporting of reproducibility studies for future researchers.

**Supplementary Information:**

The online version contains supplementary material available at 10.1007/s00439-022-02455-8.

## Introduction

Acute myeloid leukemia (AML), characterized by pathological proliferation and accumulation of clonal myeloid cells, is the most common acute hematologic malignancy in adults (Röllig and Ossenkoppele 2021). Primarily due to biological heterogeneity, patients with AML are expected to have varying post-therapeutic prognoses. Modern molecular techniques today have made the cytogenetic and genetic information of AML available, and it has become standard in clinical settings to initiate therapy by incorporating these individual profiles into a risk stratification. Compared to the current WHO or ELN classifications (Arber et al. 2016; Dohner et al. 2017), which define risk groups according to the presence of a long list of genetic aberrations, statistical algorithms are thought to be more capable of processing the high-dimensional AML data comprehensively, leading to a more accurate prediction of prognosis among risk subgroups who have been given a specific treatment strategy.

One promising example is the personalized, therapeutic decision support tool for AML patients proposed by Gerstung et al. (2017), subsequently referred to as *the knowledge bank approach*, or the KBA. The KBA considered the choice between allogeneic hematopoietic cell transplant (allograft) in first complete remission (CR1), on the one hand, and standard chemotherapy in CR1, followed by salvage treatments (either allograft or more intensive chemotherapy) after relapse, on the other. They used a training database of 1540 patients from three clinical trials (AMLHD98A, AMLHD98B, and AMLSG0704) of the German–Austrian AML Study Group (Schlenk et al. 2004, 2010, 2016), to construct the KBA prediction algorithm. It integrated 105 clinical, cytogenetic, and genetic factors to predict three-year overall survival as the primary endpoint, and demonstrated an improved predictive accuracy as opposed to the ELN classification (Harrell’s C-index: 0.72 vs 0.64). The KBA also allowed individual prediction of different survival rates (e.g. alive without CR1, alive in CR1, and alive after relapse) in possible therapeutic scenarios: receiving allograft in CR1, salvage allograft after relapse, or standard chemotherapy only.

Although of high clinical relevance, their results do not seem to have been translated into clinical practice. The improved predictive value of the KBA was validated later by Huet et al. (2018) using a retrospective cohort of 155 AML patients. Recently, Fenwarth et al. (2021) took a step further by looking at *NPM1* minimal residual disease and using the KBA to reclassify the current ELN rule. They emphasized the potential applicability of the KBA among a younger cohort (C-index for five-year overall survival using their modified KBA compared to the ELN: 68.9 vs 63.0). Yet neither study investigated the black box of the KBA.

Notwithstanding the fact that Gerstung et al. provided a web portal (https://cancer.sanger.ac.uk/aml-multistage/) which allows readers to generate outcome predictions effortlessly, it hides the complexity of their algorithm and hence leaves limited information about the implicit statistical assumptions and the derivation details behind the multistage KBA. To find these, one must reference the 135-page Supplementary Note (https://github.com/gerstung-lab/AML-multistage/tree/master/doc). Their main article published in *Nature Genetics* remains, however, the tip of a methodological iceberg. Therefore, reproducing their research is a precondition for a better understanding of their methods and for performing credible external validation (preferably with newer data that reflect substantial therapeutic advances in recent decades). Only then can the refinement and application of the KBA be possible.

Fortunately, the original data and analysis code were publicly accessible and allowed for deeper inspection. It enabled us to perform a reproducibility assessment through accessibility evaluation, code testing, error modification (if needed), and algorithm evaluation. This work can be seen as the first step towards a larger project, with the goal of validating and modifying the KBA with a more current, external AML database in Germany. In the following sections, we describe the results and findings of our reproducibility study.

## Materials and methods

### Reproducibility criteria

To date, there are no established guidelines available concerning how to perform and report a reproducibility study, and many scientists report their attempts to reproduce published results as case studies in a rather ad hoc fashion (Gentleman 2005; Hothorn and Leisch 2011; Kitzes et al. 2017). In the light of the study by Seibold et al. (2021), the Fair principles (Wilkinson et al. 2016), the reproducibility standards proposed by Heil et al. (2021), as well as the editorial guidance on reproducible research by Hofner et al. (2016) for the Biometrical Journal—one of the few (if not the only) statistical and biomedical journals requiring mandatory reproducibility checks—we propose the following criteria (summarized as a checklist in Table 1) and processes to understand the extent to which the findings of Gerstung et al. could be reproduced.

**Table 1:** Reproducibility checklist

Aspect	Item No	Item	Note
Accessibility (yes/partially/no)	1a	Data	Are the FAIR principles respected? Is there sufficient metadata to understand the data? Is the data provided in an easy-to-use format?
	1b	Is the data (if available) original, processed, anonymized, or simulated?	Simulated data is usually provided when the original data is confidential
	1c	Data dictionary	A collection of names, definitions, descriptions, etc., of variables in the dataset(s) of the research project
	2	Source code	Is the source code a plain text script or presented as a dynamic report?
	3	Documentation of the project	Is there a README file, technical note, and/or study protocol?
	4	Statistical software	Is the specific version, e.g. R (v.3.1.2), accessible?
	5	Software extensions	Are the specific versions, e.g. survival (v.3.2–13), accessible?
	6	Operating system and hardware layer	Does the reproducer have access to the same computing platform, e.g. Debian GNU/Linux 11?
	7	Can dependencies be set up easily on the reproducer’s computing platform?	e.g. By running simple commands
	8	If not, are there any compatibility issues hindering the setup process?	
Clarity (yes/partially/no)	9	Description of methods	e.g. Theoretical concepts, analytical strategies, algorithmic considerations
	10	Code readability	Is the code self-explanatory, regardless of comments? Does the code follow any style guide? Are compiled languages like C or C + + used?
	11	Inline comments	Are the comments comprehensible? Are there unnecessary obsolete code lines?
	12	Documentation of custom packages and functions, if applicable	e.g. R package vignette
Code execution	13	Is any form of testing on the functions/packages performed?	e.g. *R CMD check, testthat*
	14	(Running analysis code) On mouse-clicks / Minor modifications required / Major modifications with expertise required (e.g. reverse engineering of results) / Impossible to rerun	
Implementation of the theoretical methods	15	Consistent / Largely consistent / Largely inconsistent / Unable to identify	Does the code reflect the methods described in the paper?
Matching of outputs	16	Format of the results	e.g. tables, figures, GUI (graphical user interface)
	17	Identical with exactly the same results / Same interpretation with deviations in numbers / Inconsistent conclusions / Unable to reproduce the results	
Overall reproducibility	18	Reproducible / Partially reproducible / Irreproducible	
	19	Background of researcher(s) performing the assessment	e.g. Clinician, epidemiologist, bioinformatician, (bio)statistician, engineer

### Evaluation processes

Two researchers (YX is a clinical epidemiologist and UM is a mathematician) undertook the rerun collaboratively. First, the accessibility of materials essential for re-computation was evaluated, including data, analysis code, relevant documents (e.g. description of its theoretical background), and computing environment (i.e. the specific version of R with accompanying R packages and the corresponding operating system (OS) used in the original paper). Second, during the run of the code, checks were performed to ascertain whether there were any warnings or errors occurring and, if so, whether they could be eliminated. Third, code readability was assessed to determine if the source code was self-explanatory and whether the inline comments, as well as the documentation of custom R packages, were understandable. Fourth, to check the reproducibility, we strictly followed the data processing strategies in the original paper, with respect to the preparation of genetic covariates, and statistical analyses, such as rules for covariate selection. Modifications were only made when code errors from the provided R script prevented the continuation of the process. In other words, analytical errors such as incorrect subsetting of the dataset, if any, were not modified if they did not abort the execution. Finally, the rerun of the code allowed for insights into the conceptual ideas behind the KBA so that the appropriateness of the implemented analytical methods could be clarified.

The code was first inspected on a standard personal computer (macOS Monterey Version 12.0.1/M1 with R v.4.1.1, R Core Team, 2021), and then run in an RStudio Server Pro (Debian GNU/Linux 11 with R v.4.0.4) on a cloud server. Due to software updates, dependency conflicts occurred at the time of our study (2021) when using R (v.3.1.2) and the accompanying R packages (the version the original analysis would have been running in 2016). In general, however, we believe the source code of scientific work should be robust enough against the upgrade of a computing environment to at least generate results leading to comparable conclusions, even if identical numbers cannot be obtained. Moreover, as Gerstung et al. also provided a Dockerfile in addition to the source code, we tried to use the Docker container to build an identical computing environment to increase the chance of reproducibility.

### Data and materials

Gerstung et al. states in their paper: “To maximize reproducibility, details of statistical methods and all of the analysis code used are provided in the Supplementary Note and as a git repository online”. Specifically, the original data in an anonymized form, accompanied by source R code and other supplementary materials are publicly available in the online GitHub repository (https://github.com/gerstung-lab/aml-multistage). This repository is licensed under the GNU General Public License v3.0, which grants end users the freedom to distribute and modify the published content for commercial, patent, or private use (https://github.com/gerstung-lab/AML-multistage/blob/master/LICENSE).

## Results

### Reproducibility assessment

Overall, rerunning the analysis was challenging and required considerable time commitment, sufficient expertise in biomedicine, statistics, and R programming, and even some background knowledge in systems engineering. In the end, our efforts to reproduce their analysis were only partly successful. We present our assessment below with a summary (Table 2) using the proposed checklist (Table 1).

1. Accessibility

**Table 2:** Reproducibility assessment of the study by Gerstung et al.

Aspect	Item No	Item	Assessment	Note
Accessibility (yes/partially/no)	1a	Data	Partially	Most data available, TCGA clinical data not provided
	1b	Is the data (if available) original, processed, anonymized, or simulated?	Anonymized	
	1c	Data dictionary	Partially	Data dictionary incomplete
	2	Source code	Yes	Documented in the accompanying Supplementary Note
	3	Documentation of the project	Yes	Supplementary Note and README files provided
	4	Statistical software	Yes	R (v.3.1.2)
	5	Software extensions	Yes	Version information listed in the Supplementary Note
	6	Operating system and hardware layer	Partially	The authors have no access to the LSF environment used by Gerstung et al.
	7	Can dependencies be set up easily on the reproducer’s computing platform?	No	Dockerfile is not complete enough to allow a rebuild of the original computing environment
	8	If not, are there any compatibility issues hindering the setup process?	Yes	Today’s R (v.3.1.2) does not support many packages used; conflicts between OS and R (v.3.1.2) occurred
Clarity (yes/partially/no)	9	Description of methods	Yes	Documented in the Supplementary Note
	10	Code readability	Partially	Some variable names not self-explanatory or consistent; coding errors spotted; R style guide seemingly not followed; C + + used via Rcpp package
	11	Inline comments	Partially	Inline comments are helpful however not sufficient; several code lines are commented out but not deleted
	12	Documentation of custom packages and functions, if applicable	Yes	Two custom packages with documentation provided: *mg14* and *CoxHD*
Code execution	13	Is any form of testing on the functions/packages performed?	Partially	*R CMD check* for *CoxHD* performed; testing for the analysis code seemingly not performed
	14	(Running analysis code) On mouse-clicks / Minor modifications required / Major modifications with expertise required (e.g. reverse engineering of results) / Impossible to rerun	Major modifications with expertise required	Coding errors, limited cross-platform portability
Implementation of the theoretical methods	15	Consistent /Largely consistent / Largely inconsistent / Unable to identify	Consistent	
Matching of outputs	16	Format of the results	Main paper: figures; Supplementary: tables and figures; *Shiny* web portal	
	17	Identical with exactly the same results / Same interpretation with deviations in numbers / Inconsistent conclusions / Unable to reproduce the results	Same interpretation with deviations in numbers / Unable to reproduce the results	Minor deviations spotted among the results that could be regenerated, see **Supplementary File 2**; these did not alter the study conclusions substantially if at all. However, some results remained irreproducible
Overall reproducibility	18	Reproducible / Partially reproducible / Irreproducible	Partially reproducible	
	19	Background of researcher(s) performing the assessment	A clinical epidemiologist and a mathematician

As we noted, the GitHub repository provided a folder containing anonymized data, a Supplementary Note, together with other relevant materials (Dockerfile, README, etc.) sorted in a readily comprehensible way.

However, after running through the provided R script, we noticed that one of the two TCGA data files (i.e. TCGA_Clinical.txt) from the cancer genome atlas, used for external validation, was not provided. Although the TCGA database is publicly available (Cancer Genome Atlas Research et al. 2013), it remained unclear to us how this file was extracted and processed from the TCGA portal (https://portal.gdc.cancer.gov/projects/TARGET-AML). Thus, the corresponding results could not be reproduced (Sects. 4.4.3 and 4.4.4 in the Supplementary Note). A data dictionary was attached, yet it was found to be incomplete (Supplementary File 1.1), and one needed to refer to additional papers (Schlenk et al. 2004, 2010, 2016) before making sense of the Supplementary Note (Supplementary File 1.2). For instance, the Note mentioned the abbreviations *CIR, MUD,* and *RD* without explaining their meanings. They are, in fact, short for *Cumulative Incidence of Relapse*, *Matched Unrelated Donors*, and *Refractory Acute Myeloid Leukemia*, respectively. Two different versions of the Supplementary Note were found: one that went with the main paper on *Nature Genetics’* website (version: Wed Sep 7 14:26:11 2016, see https://www.nature.com/articles/ng.3756) and another available in the repository, which was incomplete (version: Tue Dec 15 17:54:15 2015). The more recent version was referred to in this study.

The published results were originally obtained using R (v.3.1.2); however, many packages used in the analysis were no longer supported by R (v.3.1.2). Of note, the latest available R (v.4.1.1) used in this study in 2021 did not support several packages either, such as *graph* or *hilbertVis*. As a result, other than the common way of executing the command *install.packages()* for the installation, additional searching was needed to avoid errors. Some intensive computations, such as leave-one-out cross-validation, were performed by Gerstung et al. in a Load Sharing Facility (LSF) environment. Without access to such an environment, one must manually modify the code considerably to proceed in another computing platform, whilst these modifications are also prone to error.

**Fig. 1:**
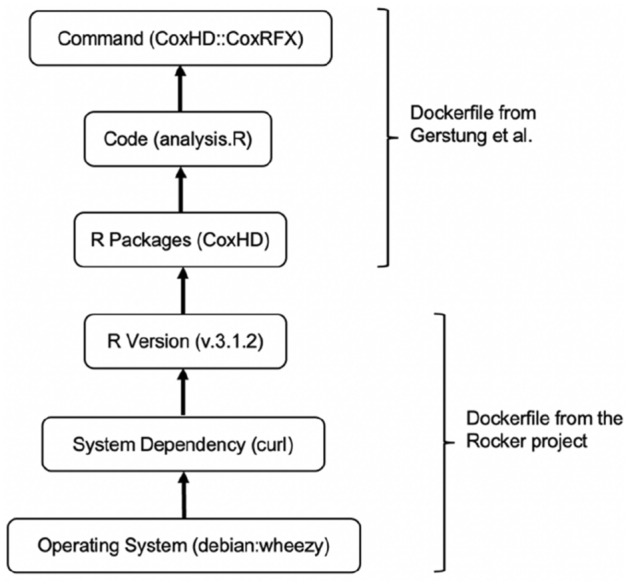
Six-layered structure of an R computing environment. The Dockerfile from Gerstung et al. constructs layers upon a pre-built Docker-based R (v.3.1.2), which was built by reading the instructions described in the Dockerfile from the Rocker project; however, dependency conflicts occur across these layers

The Dockerfile was once deemed an opportunity through which we could easily re-establish an identical computing environment with R (v.3.1.2) and the corresponding packages. However, our attempts were unsuccessful, since the given Dockerfile assumed the existence of a Docker-based R (v.3.1.2), and only described how their author-customized R packages (i.e. *mg14* and *CoxHD*) could be built on top of that. Today, this is insufficient to build a reproducible environment due to compatibility issues. Specifically, the OS (i.e. *DebianWheezy*) specified in the Dockerfile from the Rocker project (https://www.rocker-project.org), upon which the R (v.3.1.2) is to be built, has been too old to support essential R packages for this analysis (Fig. 1 depicts the layered structure of an R computing environment).

2. Clarity

The methodological details and analysis code were provided in the Supplementary Note. Overall, the source code was consistently written and relative paths were used to allow data to be read into R on different devices without manual alterations. Inline comments, although with a few insignificant inaccuracies and undeleted obsolete commands, remained very helpful for readers to understand the analysis. Documentation of user-defined packages and functions could also be easily acquired.

Still, as the research project is very complex (needing more than 5000 code lines in total), the source code did not appear to have followed the programming style guide for R and was not clear enough to us. Inconsistencies were identified throughout the code, which impacted the code readability, such as: different names created for the same concept (e.g. *Time_Diag_TPL* and *TPL_date*, *Cir* and *Rel*, *kmPrs* and *kmPrd*); different concepts with the same name (e.g. *CIR* could mean either *cumulative incidence of relapse* or *Kaplan–Meier survival estimate for relapse*); non-existent variables or values called in the commands (e.g. variable *TPL_efs* was not found in R data frames, but appeared in the code); objects that did not match the assigned purposes as suggested by the inline comments; and objects that were created, however, were not used (see details in Supplementary File 2).

3. Code execution

A mouse-clicking rerun through the code to reproduce the results was impossible. The first coding error appeared due to a command line (i.e. *dataList$Genetics* = *dataList$Genetics* + *0*) in the 8th code chunk of the Supplementary Note (p. 13), which introduced undue factor variables to the list *dataList$Genetics* and stopped one from moving forward. We corrected this error by changing the command to *dataList$Genetics[,52:58]* *<-* *as.data.frame(lapply(dataList$Genetics[,52:58], as.numeric))*, one of the aforementioned modifications we made to enable progress. More errors occurred throughout the Note and aborted the execution within their corresponding sections. Specifically, Sects. 3.6.5.1–3.6.5.9, 3.6.6.4, 3.6.6.6–3.6.6.7, 3.6.7.2–3.6.7.3, 4.4.1.3, 5.4.2.0.4, 5.4.2.0.5, 5.4.2.1.1, 5.4.2.2.1, and 5.4.2.3.1 contained parallel processing and were originally executed on an LSF environment, which was different from our computing environments. For this, we were able to tailor only parts of the environment-dependent R script (Sects. 3.6.6.4 and 3.6.6.6) in our rerun. Even so, our alterations were liable to mistakes and might have exacerbated the irreproducibility. We reported the errors and our modifications in Supplementary File 2, together with our GitHub repository (https://github.com/YX-IBE/AML-multistage-reproducibility-study) with version control to track the modifications.

4. Implementation of the methods described

The multistage KBA algorithm in the code reflected the ideas described in the paper. Still, it is noteworthy that the random-effect Cox models—the building blocks for the multistage KBA—assumed grouped predictors to be following normal distribution, and then computationally led to a ridge regularization (Therneau et al. 2003). This simplified the computations and was realized by specifying the ridge regression function argument in *coxph()*, which was integrated into a user-defined function *CoxRFX()* (to learn this, we used the function *debug()* to step through the execution of *CoxRFX()*). It should be noted, as Gerstung et al. claimed in the Supplementary Note and in their function help file, that the parameter estimation was done via an Expectation–Maximization algorithm as suggested by a simulation study of Perperoglou (2014), which, in fact, favored the Restricted Maximum Likelihood-type method for the handling of random effects. Therefore, we could not fully understand how the *CoxRFX()* fits the random-effect Cox model.

5. Matching of outputs

We were able to partly execute the R script after necessary modifications. With the random seed fixed, deviations were spotted throughout the outputs in contrast with the published results at various locations, yet most of them were negligible and did not alter the corresponding study conclusions, with numbers differing in the last few decimal places, or figures with minor alterations. For instance, the predicted three-year survival rate given the optimal therapeutic strategy was 63.68%, as compared to 62.41% in our rerun (relative difference: 0.02). More comparisons of the outputs from our rerunning and the published results were documented in Supplementary File 2 (see p. 67–68 for three-year optimal survival). Notably, our multiple rerunning attempts returned the same warnings and errors occurring at the same locations, while discrepancies between our results (tables and figures) also appeared. This suggests that, besides the impact of a different computing platform (with the consequent difference in numerical precision, operation sequence and so on), there might still be hidden issues relating to random number generation, which led to deviations between the outputs from our multiple attempts.

**Fig. 2:**
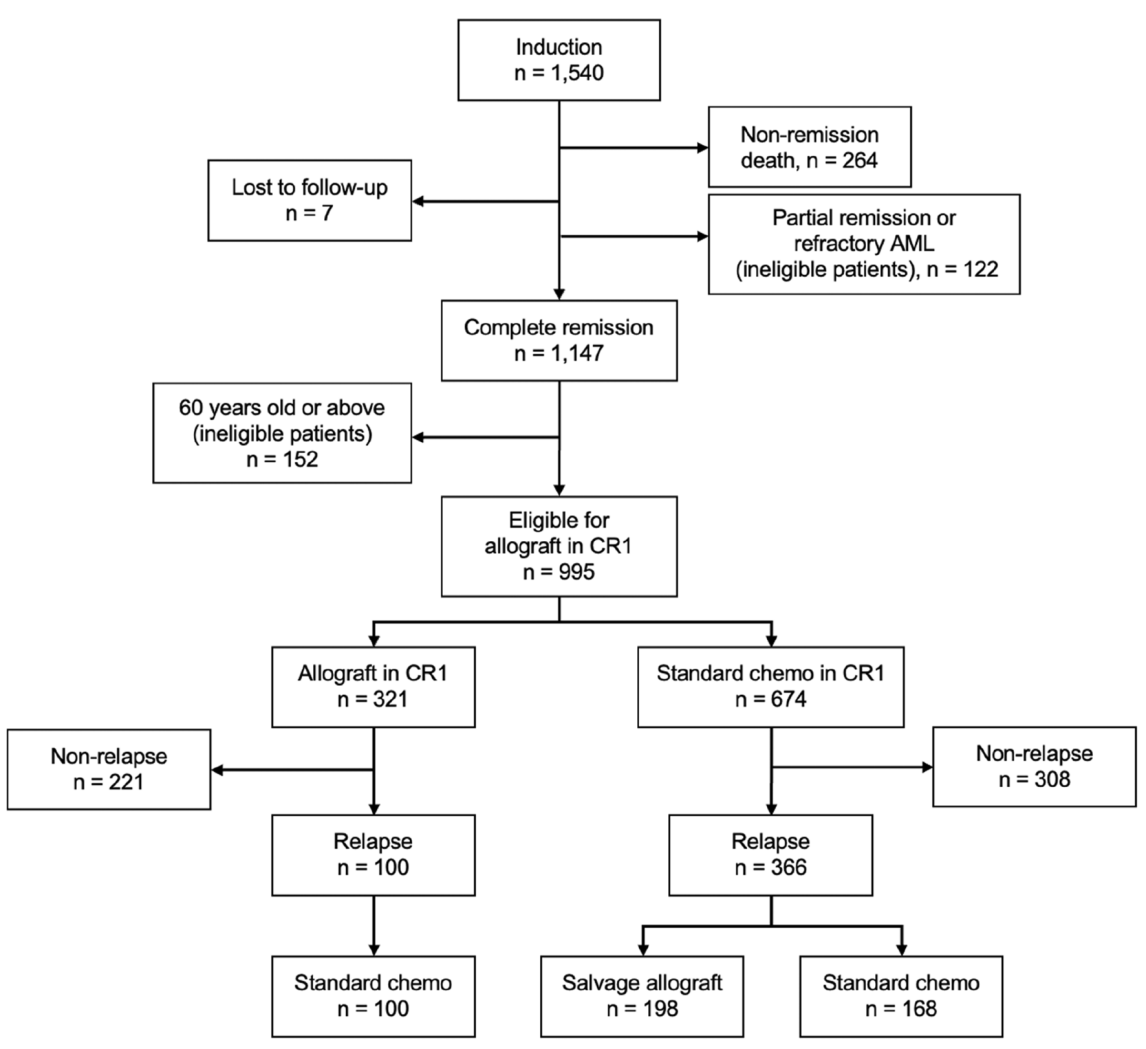
Flow chart showing treatments and prognoses of AML patients in the knowledge bank database

**Fig. 3:**
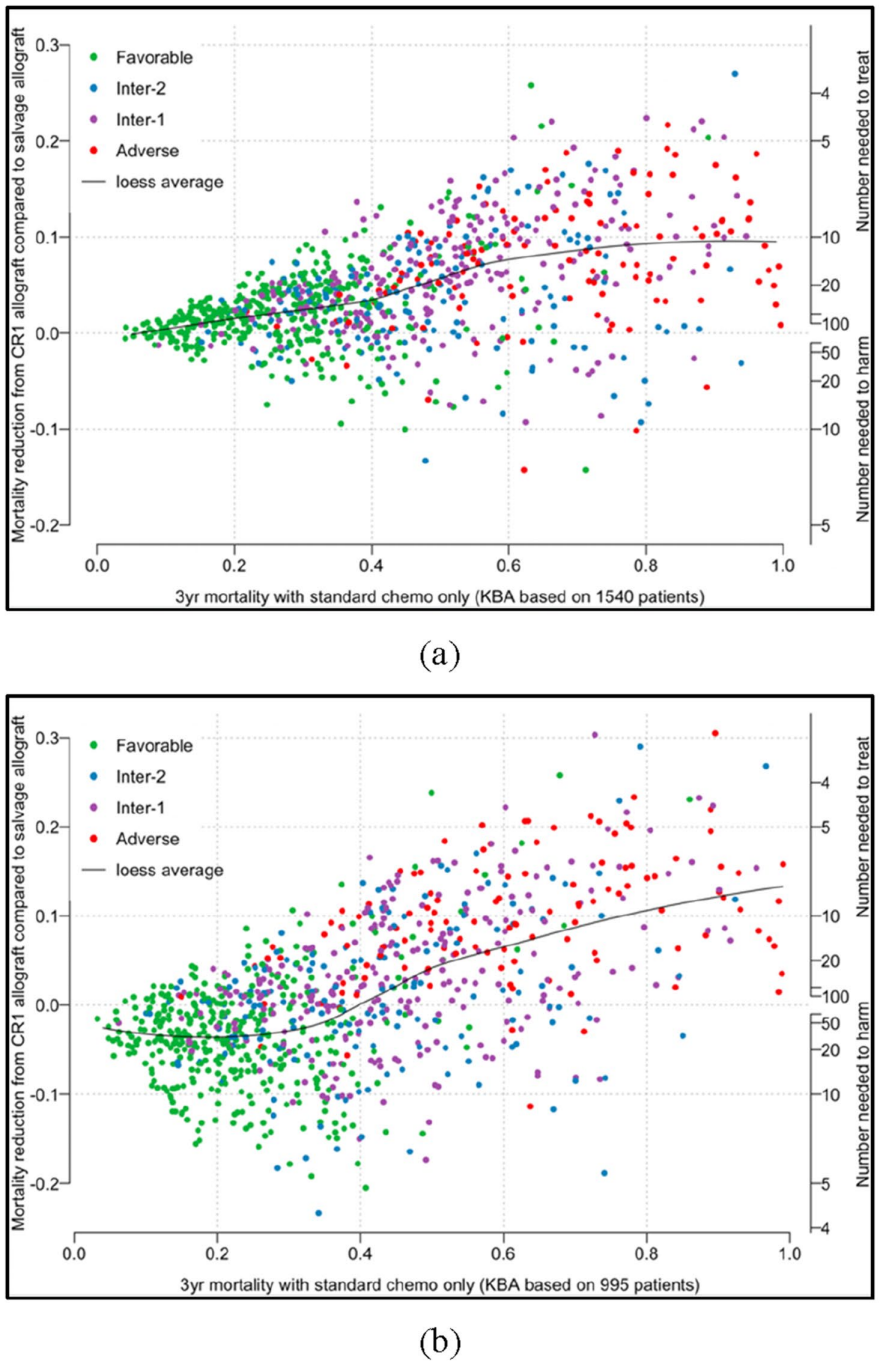
Predicted 3-year mortality reduction from allograft in CR1, as opposed to salvage allograft after relapse (*y*-axis), predicted 3-year mortality of standard chemotherapy only (*x*-axis). Date of complete remission as the starting point. Individual patients denoted by the points and colored by ELN risk classifications. Population average mortality fitted and presented by the curve. **a** The KBA was based on the entire 1540 patients, while predictions were calculated for 995 patients eligible for allograft at CR1, adapted from Gerstung et al. (2017); **b** Modified KBA based on the 995 eligible patients

There were in total eighteen sections in the Supplementary Note involving parallel computing (as mentioned above), for which we were unable to modify the R script to rerun the analysis. This relates to the major prediction results presented by Gerstung et al. (i.e. Figs. 1b, 2c, 2d, 2e, 3, 4, and 6c in the original paper), as well as the web portal provided. As noted earlier, two sections involving the TCGA data could not be rerun either, which further impacted the regeneration of Figs. 1a and 1c in their paper. Therefore, only Figs. 2b, 2f, 5, 6a, and 6b were fully or partially reproduced by us. More results in the Supplementary Note that were reproducible were documented in Supplementary File 2.

6. Further notes regarding the published results

After the rerun of the analysis, we established the full picture of prognostic trajectories among the 1540 patients in the KBA database (Fig. 2); of those, only 995 patients were considered eligible for allograft in CR1.

At this point, more details concerning the theoretical concepts in the paper by Gerstung et al. could be further clarified. The core of the methodological strategy for the KBA was a random-effect multistage model combined with competing risk adjustments, where the effects of oncogenic lesions were assumed following a Gaussian distribution. The KBA was trained in a data-driven manner to optimize its predictive accuracy. Depending on the moment when the prediction is to be made: at diagnosis or on reaching CR1, two separate predictive algorithms were constructed using the data of all 1540 patients. The impartial integration into the two models of post-diagnosis information (e.g. the length of induction or the length of CR1) appeared questionable since these predictors were chronologically unavailable for making predictions at diagnosis. Furthermore, the second model starting from CR1 was used to answer the question of who should be offered allografts in CR1 by comparing the predicted three-year mortality between receiving allografts in CR1 and salvage allografts (Fig. 3a, adapted from Fig. 5a in the original paper). In the original paper, the predictions were restricted to the 995 patients eligible for allograft and were done by altering the predictor value of treatments given in CR1 for each patient, which represented the conflation between prediction and causal inference.

## Discussion

We have evaluated the reproducibility of the research by Gerstung et al. published in *Nature Genetics*—the KBA that generated personally tailored predictions of survival using individual clinical and genomic profiles. This reproducibility study gives insight into the theoretical details and how to replicate the algorithm in future studies. Implications for the original research can be considered in two ways.

Firstly, the primary objective of the original study is risk prediction. Regardless of the internal validation via subsampling techniques and simulations run on the cluster, which we failed to execute, our reproduced results (Supplementary File 2) were overall in line with the conclusions from their paper with regard to the predictive potential of the KBA, as compared to the current ELN classification. However, it is necessary to determine the time point when the prediction is to be made and modify the KBA accordingly. In particular, information accumulated after prediction should be discarded from the KBA, as such information leakage usually leads to over-optimistic predictive accuracy. To illustrate, the length of CR1 is associated with a higher chance of receiving allograft as well as with an improved survival rate, hence including this information tends to increase the model predictive accuracy. Furthermore, to predict risk in hypothetical scenarios where an individual receives different treatments, e.g. receiving allograft in CR1 or after relapse, a counterfactual prediction approach is needed for future model development (Dickerman and Hernán 2020; van Geloven et al. 2020).

Secondly, our reproducibility study clarified that the KBA was trained and assessed empirically so as to optimize the predictive performance with all predictors in combination; in other words, the underlying causal structure was not considered, and hence one cannot be sure if confounding effects have been handled appropriately (Ramspek et al. 2021; Shmueli 2010). Although not explicitly described as causal inference, the comparison of mortality between different treatments (Fig. 3a) incorrectly invites etiological interpretation in favour of receiving allograft in CR1, particularly among patients with higher baseline risk: a potentially dangerous interpretation which has been accepted in two subsequent studies (Fenwarth et al. 2021; Huet et al. 2018).

The impact of mistakenly attributing the observed mortality reduction to receiving allograft in CR1 could be detrimental in clinical settings, as it can encourage offering a treatment while the asserted causal efficacy might not, in fact, exist. The conflation of prediction and causal inference affects each step of the modeling process, and even one single step could easily alter the research conclusions (Breiman 2001; Shmueli 2010). For illustrative purposes, to demonstrate how the original research findings would be impacted, we rebuilt the KBA by restricting it to the 995 patients who were eligible for allograft in CR1 and consequently were facing clinical uncertainty, as is required to answer the causal question. It can be seen in Fig. 3b that, assuming the confounding effects have been sufficiently addressed by the KBA, the estimated benefits from timely allograft in CR1 no longer pertain among those who were predicted to have a lower 3-year mortality (i.e. < 40% if receiving standard chemotherapy, as indicated by the clusters to the left of *x* = 0.4).

The value of open policies regarding data and relevant supplements (e.g. code and documents) has been widely recognized by the scientific community, and tremendous efforts have been made to foster sharing processes enacting these open policies (Open Science 2015; Wilkinson et al. 2016). Today, data repositories, either generalist such as Dryad (https://datadryad.org/) and Zenodo (https://zenodo.org/) or discipline-specific like BioModels (https://www.ebi.ac.uk/biomodels/) and PhysioNet (https://physionet.org/); code repository hosts, e.g. GitHub (https://github.com/) and GitLab (https://gitlab.com/); and the corresponding solutions to data transferring, e.g. SFTP (https://www.ssh.com/) and Aspera (https://www.ibm.com/products/aspera), and repo-to-repo code obtaining, e.g. git clone (https://github.com/git-guides/git-clone), have materially removed the obstacles in accessing data and analysis code.

With full access to source materials, however, our study again attests to the fact that openness is merely a starting point for reproducible research. Based on the experience of the first author (YX) serving as a reproducible research editor at Biometrical Journal and informal correspondence with a senior colleague, at present only about two out of every ten submissions (i.e. manuscripts with supplementary data and code) are found to be reproducible from the start, while for the rest, three to four rounds of communication and correction between authors and editors, just to improve reproducibility, are standard. In the context of this study, we discuss three aspects of reproducibility given that open data and code policies have been followed: code readability, executability, and development.

1. Readability

Code readability is not necessarily a prerequisite for a successful rerun of the code, provided best practices in software engineering have been employed. In biomedical research, however, clean and comprehensible code not just increases the likelihood that negligent errors will be spotted, more importantly, it enables downstream researchers to grasp the underlying theoretical assumptions or uncover biases that were unseen earlier on, as some implementation details are often omitted in the original paper. Even a successful rerun of the code does not guarantee that the outputs provide the information originally planned to be extracted from the data. In this regard, code readability is central to reproducibility.

Practically, following programming style guides, e.g. PEP 8 for Python (https://peps.python.org/) and Tidyverse Style Guide for R (https://style.tidyverse.org/) is the basis of enhancing code readability (Schwab and Held 2021), and there are packages automatically formatting the code to conform to a given style guide, e.g. *autopep8* for Python (https://pypi.org/project/autopep8/) and *lintr* for R (https://github.com/r-lib/lintr). Another critical element of improving code readability is to avoid duplication and unnecessary complexity, in that these inevitably entangle the analysis flow and are ‘bug-prone’ due to the decreased code maintainability. Although code and functions were substantially abstracted by Gerstung et al. through building up a package (Caffo et al. 2016), duplicated and complex code were still appearing and hence code refactorings such as function extraction and variable renaming are recommended (Fowler 2018). Moreover, the myth that ‘good code explains itself’ also has repercussions in reproducible research, and the ensuing obstacles to comprehending the code are magnified when a research project involves multidisciplinary knowledge. For this reason, while ‘what’ and ‘how’ comments are deemed unnecessary, we believe inline comments remain an indispensable complement in providing discipline-specific backstory and in explaining the purposes of certain execution steps. The availability of computational notebooks such as R Markdown (https://rmarkdown.rstudio.com/) and Jupyter Notebook (https://jupyter.org/), which provide a dynamic narrative of all the data analysis steps, from data preprocessing to visualization, has greatly facilitated collaboration and has provided substantial versatility in improving code quality (Perkel 2018). Nonetheless, one should note that simply implementing the notebooks is not a catch-all solution for best programming practices, and criticism has also been made of these notebooks encouraging poor coding practices and hence hindering reproducibility (Perkel 2021; Pimentel et al. 2019). Of note is a recent study proposing a framework of five-step code quality assurance to improve computational reproducibility before the fact (Sanchez et al. 2021).

2. Executability

From the perspective of software engineering, the essence of reproducibility is to ensure code execution by the reproducer, independent of the original author’s computing environment. There are many best engineering practices in this field that can help in achieving this objective. Documentation of code, statement of dependencies, modular programming (which in our case boils down to separating the code into individual analytical purposes), and function abstraction appear to have been broadly implemented by Gerstung et al.. But, four years after the original paper was published, we encountered dependency conflicts and portability issues (i.e. executing the same code on another OS or machine) when performing this study. Next, we elaborate on how the setup of dependencies and testing could have been improved.

Modern software like R or Python is continuously updated, and the untoward impact of running the code in an upgraded computing environment might result in small output deviations, code execution abortions, or even a complete dependencies setup failure. Containers, e.g. Docker (https://www.docker.com/) and Singularity (https://sylabs.io/singularity), are open-source tools providing an opportunity to address the compatibility issues and to re-install an identical computing environment for specific tasks, increasing the chance of reproducibility (Boettiger and Eddelbuettel 2017). In contrast to the incomplete Dockerfile (see Fig. 1) provided by Gerstung et al., a reusable one should describe all steps needed to build a layer-structured Docker image from the bottom OS, and even if this were provided, the unavailability of required downloads would still prohibit the setup process. Alternatively, an easier and more maintainable option for future researchers would be to directly provide a complete Docker image and share it via a public container repository, e.g. Docker Hub (https://hub.docker.com/). To further prevent the conflicts due to container’s reliance on OS, using a virtual machine such as VirtualBox (https://www.virtualbox.org/) or QEMU-KVM (https://www.qemu.org/) by providing virtual machine files is also recommended. Importantly, either container or virtual machine files should be successfully tested offline. Moreover, dependency management tools such as Conda (https://docs.conda.io/), Python *pip* (https://pypi.org/project/pip/), and R *packrat* (https://rstudio.github.io/packrat/) have been very helpful to track the versions of packages and dependencies in a specific project.

In data-intensive fields requiring high-performance computing, code portability is another key factor to be considered with respect to compatibility issues. In this study, R script for parallel tasks was written depending on the LSF scheduler and hence cannot be run unmodified for a different platform. For future authors, workflow managers such as Nextflow (https://www.nextflow.io/) and Snakemake (https://snakemake.readthedocs.io/) could present a solution, as they provide an abstraction to separate analysis pipelines from computing environments, and hence allow seamless execution of the pipelines across different platforms (Di Tommaso et al. 2017; Mölder et al. 2021).

Unlike a standard approach to improving code executability in software development, testing is perceived rather arbitrarily in some fields such as biomedicine, where writing code for individual purposes (so-called end-user programming) is typical (McConnell 2004; Silva et al. 2017). With regard to testing in a biomedical research project, functions/packages and analysis code should be considered separately. Unit testing is pertinent mainly to testing the functions/packages at individual levels. For the provided package *CoxHD* in the original paper, the *R CMD check* was performed. The *R CMD check* is an essential testing facility provided by R running a variety of checks automatically (https://r-lib.github.io/rcmdcheck/). Passing such checks is required when submitting an R package to the CRAN (https://cran.r-project.org/web/packages/policies.html), but it only ensures the executability of functions/packages. To test the correctness of individual outputs, packages such as *testthat* (https://testthat.r-lib.org/) and *RUnit* (https://rdrr.io/cran/RUnit/) can be used alongside programming, and it is also possible to merge them into the *R CMD check* for future testing. On the other hand, integration testing of analysis code (i.e. checking whether different modules of the code, including dependencies setup, work well together) is more relevant to uncover the code errors and dependency conflicts that we experienced. To this end, original authors should test their code through putting together all modules or combining several modules into higher-level modules, depending on the project complexity. Where the virtual machine is not used, it is recommended to perform integration testing in as many different computing environments as possible, particularly in those that are most likely to be used by future reproducers, i.e. mainstream cloud computing environments like AWS (https://aws.amazon.com/) and Azure (https://azure.microsoft.com/). In addition, testing tools like Selenium (https://www.selenium.dev/) can further automate the manual testing so as to free developers from the long wait for the completion of individual executions.

3. Development process

The development process of predictive models is similar to that of software, in that the cycle of code updating and testing is common to both. A key element to smooth such a process is version control, which tracks changes to the code, prevents and resolves conflicts due to concurrent development, or restores previous versions with ease. Continuous integration, continuous delivery and/or continuous deployment (CI/CD) workflows further streamline the development process. CI allows every new change made to the code to trigger the testing in an unattended manner, while CD automates the delivery and deployment of the changes once testing is passed, such as uploading a new Docker image to Docker Hub and updating the result models. The automation allows for early detection of errors, accelerates the development cycle, and therefore increases research efficiency. We noticed that in the work by Gerstung et al., version control was enabled while CI/CD was done for the provided *CoxHD* package with Travis CI (https://travis-ci.org/). Other common CI/CD tools that can be used in R include Jenkins (https://www.jenkins.io/), GitHub Actions (https://github.com/actions), and GitLab CI (https://docs.gitlab.com/ee/ci/). On the other hand, the graphical user interface, such as the *Shiny* web portal in the original paper, is especially useful to communicate sophisticated models and promote external validation. An alternative to *Shiny* (https://shiny.rstudio.com/) is *Plumber* (https://www.rplumber.io/), which brings about greater flexibility in connecting the source code to a custom interface other than the standalone *Shiny* webpage.

It should be noted that based primarily on the context of this reproducibility study, our discussion did not cover some best practices that are commonly seen in other fields, for example, automatically pulling an updated container image from the repository to deploy. Moreover, the three aspects (code readability, executability, and development) are not mutually exclusive. To illustrate, version control is a key element in facilitating code executability and development, but it also improves code readability and comprehensibility by displaying how the code evolves. In addition, trade-offs are to be made depending on the researcher’s engineering resources; where these are scarce, much more attention should be paid to code readability for reproducibility. Still, researchers should always strive to keep up with the best programming and software engineering practices, otherwise a reproducibility study would be a post-mortem examination of the original paper.

## Conclusion

The scientific community arguably values the novelty of research more than reproducibility, which systematically discourages the practice of reproducibility studies (Atmanspacher and Maasen 2016). Compared to the upfront research that generates and collects data through wet-lab experiments or large-scale population surveys, computational reproducibility remains in some ways the easiest part, even taking into account the technical challenges we have encountered in this study. However, this study reaffirms the scientific value of sound reproducibility practices; even a single reproducibility study can add to the credibility of published results (Ioannidis 2014; Seibold et al. 2021). Moreover, publishing and studying code offers access to a rich source of statistical wisdom. The code provided by Gerstung et al. has taught us an innovative application of methodological concepts which are undocumented elsewhere. It was a very valuable learning experience to study the code, even without rerunning it successfully, to deepen our understanding of Cox proportional hazards regression models with grouped random effects and the handling of the multistage processes. Lastly, we noticed that most of the current publication standards, including individual journals’ submission guidelines for authors and general reporting guidelines such as TRIPOD (Collins et al. 2015) or STROBE (von Elm et al. 2007), do not require the scrutiny of reproducibility before a submission can be accepted. The lack of relevant guidelines for the conduct and reporting of reproducibility studies means that good practice in this matter remains an open issue. Therefore, this paper proposes criteria for reproducibility (presented in Table 1), which are open for discussion and improvement, in the hope that this will not only encourage the publication of more reproducibility studies but also help future researchers to perform reproducibility checks for themselves.

## Supplementary Information

Below is the link to the electronic supplementary material.

Supplementary file1 (PDF 58 KB)

Supplementary file2 (PDF 26978 KB)

## Data Availability

The datasets analyzed in the current study and supplementary materials are available in an online GitHub repository provided by Gerstung et al.: https://github.com/gerstung-lab/aml-multistage.

## Abbreviations

**AML**: Acute myeloid leukemia
**Allograft**: Allogeneic hematopoietic cell transplant
**CI/CD**: Continuous integration, continuous delivery and/or continuous deployment
**CR1**: First complete remission
**ELN**: European LeukemiaNet
**GUI**: Graphical user interface
**KBA**: Knowledge bank approach
**LSF**: Load sharing facility
**OS**: Operating system

## Code availability

The source R code is publicly accessible in the GitHub repository (https://github.com/gerstung-lab/aml-multistage). In addition, we provide our own GitHub repository to track the modifications made in this reproducibility study with version control: https://github.com/YX-IBE/AML-multistage-reproducibility-study
