# Supplementary material for: Validating the knowledge bank approach for personalized prediction of survival in acute myeloid leukemia: a reproducibility study
Source: Hum Genet. 2022 Apr 16;141(9):1467–80. doi: 10.1007/s00439-022-02455-8 (PMC9360099; doi:10.1007/s00439-022-02455-8)
Supplement: Supplementary file 1 — Supplementary file1 (PDF 58 KB) [file 439_2022_2455_MOESM1_ESM.pdf]

Supplementary File 1 - Data dictionary

1. Data dictionary accompanying the original paper by Gerstung et al.

An `R` data dump containing a table with the following columns

```
Study Study ID; AMLSG0704; AMLHD98A, AMLHD98B
PDID  Sample ID
WHOcat  Not used
TypeAML AML: primary AML; sAML: secondary AML; tAML: therapy-associated AML; oAML other
Type2 Not used
C_Risk  Cytogenetic risk
M_Risk  ELN risk (2011)
NK  Normal Karyotype
AOD Age at diagnosis
Center  Treatment center
ERDate  Not used
Intergroup  Not used
Study1  Not used
BaselineTherapy_  Not used
VPA Valproic acid treament
ATRA_arm  ATRA treatment
TPL_o HSCT
TPL_type  HSCT type: ALLOgeneic, AUTOlogous, FREMD=ALLO, HAPLO
Date_LF Time to last followup
TPL_Art Not used
CR_date Time to CR
Recurrence_date Time to Recurrence
TPL_date  Time of HSCT
TPL_Phase Phase of HSCT
Time_Diag_TPL Not used
Time_1CR_TPL  Not used
Family_donnor Not used
Post_Induction_Therapy_Remission  Not used
Status  Survival status at last follow up
OS  Time of OS
EFSSTAT Status for EFS
ereignart Event Type
efs Time to EFS event
rfsstat RFS status
rfs Time to RFS event
cuminc  Not used
rfsstat_allo  Not used
rfs_allo  Not used
cuminc_allo Not used
HB  Haemoglobin level
platelet  Platelet count
wbc White cell count
```

```
LDH LDH levels
gender  Gender (1: Male, 2: Female)
BM_Blasts Bone marrow blast %
Performance_ECOG  ECOG status
PB_Blasts Peripheral blood blast %
Splenomegaly  Splenomegaly
MLL_PTD MLL partial tandem duplication
inv3_t3_3 inv(3;3)
t_9_22  t(9;22)
minus5_5q -5/5q
minus7  -7
minus7q -7q
abn7other other abnormality on chr 7
plus8_8q  "+8/8q"
minus9q -9q
mono12_12p_abn12p -12/12p
plus13  "+13"
mono17_17p_abn17p -17/17p
minus18_18q -18/18q
minus20_20q -20/20q
plus21  "+21"
plus22  "+22"
minusY  -Y
t_15_17 t(15;17) PML-RARA
t_8_21  t(8;21)
inv16_t16_16  inv(16)/t(16;16)
t_6_9 t(6;9)
abn3q_other other abnormality on chr 3
plus11_11q  "+11/11q"
mono4_4q_abn4q  -4/4q
complex Complex karyotype
NONC_CYTO Numer of oncogenic mutations, including cytogenetic aberrations
NALL_CYTO Toal number of mutations, including cytogenetic aberrations
X1  Not used; aberration on chromosome 1
X2  Not used
X3  Not used
X4  Not used
X5  Not used
X6  Not used
X7  Not used
X8  Not used
X9  Not used
X10 Not used
X11 Not used
X12 Not used
X13 Not used
X14 Not used
X15 Not used
X16 Not used
```

```
X17 Not used
X18 Not used
X19 Not used
X20 Not used
X21 Not used
X22 Not used
X Not used
Y Not used
CEBPA Mutation in CEBPA
NPM1  Mutation in NPM1
FLT3_TKD  FLT3 TKD mutations
FLT3_ITD  FLT3 ITD mutation
FLT3_other  FLT3 other mutation
t_9_11  t(9;11)
t_v_11  t(x;11)
```

2. Data dictionary updated from literature

Variable	Definition
Study	Trial ID
PDID	Patient ID
WHOcat	Not used
TypeAML	AML: primary AML; sAML: secondary AML; tAML: therapy-associated AML; oAML: other
Type2	Not used
C_Risk	Cytogenetic risk
M_Risk	ELN_criteria (2011)
NK	Normal Karyotype
AOD	Age at diagnosis
Center	Treatment center
ERDate	Actual date of diagnosis
Intergroup	Not used
Study1	1=AMLSG0704; 2=AMLHD98A; 3=AMLHD98B
BaselineTherapy_	Not used
VPA	valproic acid treatment (applies to AMLSG 07-04 only)
ATRA_arm	all-trans retinoic acid treatment

TPL_o	hematopoietic stem cell transplantation
TPL_type	HSCT type: ALLO, AUTO, FREMD, HAPLO, TPL_ (Spenderart_unbekannt)
Date_LF	Time from diagnosis to last follow-up
TPL_Art	Not used
CR_date	Time from diagnosis to CR
Recurrence_date	Time from diagnosis to Recurrence
TPL_date	Time from diagnosis to HSCT
TPL_Phase	AML phases when receiving HSCT
Time_Diag_TPL	time from diagnosis to TPL
Time_1CR_TPL	time from first complete remission to TPL
Family_donnor	Not used
Post_Induction_Therapy_Remission	Not used
Status	Survival status at last follow up
OS	Time from diagnosis to death
EFSSTAT	Status for Event-free survival
ereignart	Event Type
efs	Time from diagnosis to EFS event
rfsstat	Status for relapse-free survival
rfs	Time from diagnosis to RFS event
cuminc	Not used
rfsstat_allo	Not used
rfs_allo	Not used
cuminc_allo	Not used
HB	Haemoglobin level
platelet	Platelet count
wbc	White cell count
LDH	LDH levels
gender	Gender (1: Male, 2: Female)
BM_Blasts	Bone marrow blast %
Performance_ECOG	ECOG status

PB_Blasts	Peripheral blood blast %
Splenomegaly	
MLL_PTD	MLL partial tandem duplication
inv3_t3_3	inv(3;3)
t_9_22	t(9;22)
minus5_5q	-5/5q
minus7	-7
minus7q	-7q
abn7other	other abnormality on chr 7
plus8_8q	+8/8q
minus9q	-9q
mono12_12p_abn12p	-12/12p
plus13	+13
mono17_17p_abn17p	-17/17p
minus18_18q	-18/18q
minus20_20q	-20/20q
plus21	+21
plus22	+22
minusY	-Y
t_15_17	t(15;17) PML-RARA
t_8_21	t(8;21)
inv16_t16_16	inv(16)/t(16;16)
t_6_9	t(6;9)
abn3q_other	other abnormality on chr 3
plus11_11q	+11/11q
mono4_4q_abn4q	-4/4q
complex	Complex karyotype
NONC_CYTO	Numer of oncogenic mutations, including cytogenetic aberrations
NALL_CYTO	Toal number of mutations, including cytogenetic aberrations
CEBPA	Mutation in CEBPA

NPM1	Mutation in NPM1
FLT3_TKD	FLT3 TKD mutations
FLT3_ITD	FLT3 ITD mutation
FLT3_other	FLT3 other mutation
t_9_11	t(9;11)
t_v_11	t(x;11)
TPL_Phase	
CR1	first complete remission
PR	partial remission
RD	refractory AML
CIR	cumulative incidence of relapse
Treatment	
ATRA	all-trans retinoic acid
VPA	valproic acid (AMLSG 07-04 only)
HSCT	hematopoietic stem cell transplantation
TPL_type	
ALLO	Allogene
AUTO	autologen
FREMD	Fremdspender
HAPLO	haplokompatible
allografts (HSCT)	
MRD	matched related donors
MUD	matched unrelated donors
