# Supplementary material for: Validating the knowledge bank approach for personalized prediction of survival in acute myeloid leukemia: a reproducibility study
Source: Hum Genet. 2022 Apr 16;141(9):1467–80. doi: 10.1007/s00439-022-02455-8 (PMC9360099; doi:10.1007/s00439-022-02455-8)
Supplement: Supplementary file 2 — Supplementary file2 (PDF 26978 KB) [file 439_2022_2455_MOESM2_ESM.pdf]

Supplementary File 2

Compare our rerunning results with Gerstung et al.'s Supplementary Note (Wed Sep 7 14:26:11 2016)

- 1 Data
  - 1.1 Variables
  - 1.2 Preprocessing
    - 1.2.1 Genomic variables
    - 1.2.2 Gene:gene product terms
    - 1.2.3 Clinical and demographic variables
    - 1.2.4 Treatment
    - 1.2.5 Nuisance
  - 1.3 Code
    - 1.3.1 Libraries
    - 1.3.2 Raw data
      - 1.3.2.1 Clinical data
      - 1.3.2.2 Mutation data
    - 1.3.3 Survival data
    - 1.3.4 Covariates
    - 1.3.5 Subclonal mutations
      - 1.3.5.1 Bradley-Terry Model
      - 1.3.5.2 Supplemenary Figure S8
- 2 Models for overall survival
  - 2.1 Random effects modelling
    - 2.1.1 Implementation
    - 2.1.2 Parameter estimation
    - 2.1.3 Semiparametric bootstrap
    - 2.1.4 Analytical confidence intervals of individual parameters
    - 2.1.5 Wald test of individual parameters
    - 2.1.6 Variance components
      - 2.1.6.1 Partial log hazard
      - 2.1.6.2 Variance components
      - 2.1.6.3 Relation to concordance
    - 2.1.7 Prediction error
    - 2.1.8 Covariance-based imputation
      - 2.1.8.1 Prediction error
  - 2.2 Other survival models
    - 2.2.1 Stepwise variable selection
    - 2.2.2 Complementary pairs stability selection
    - 2.2.3 Random survival forests
  - 2.3 Code
    - 2.3.1 Number of oncogenic mutations
      - 2.3.1.1 Number of oncogenics
    - 2.3.2 Random effects models
      - 2.3.2.1 Variance components
    - 2.3.3 Confidence intervals and significance tests
    - 2.3.4 Risk constellation plots
      - 2.3.4.1 Figure S1A
      - 2.3.4.2 Figure S1B
- 3 Multistage modelling
  - 3.1 Definitions
    - 3.1.1 Nomenclature
    - 3.1.2 Transitions
    - 3.1.3 States
    - 3.1.4 Factorisation of the joint probability
  - 3.2 Static multistage models
  - 3.3 Multistage random effects modelling
    - 3.3.1 Unconditional densities
    - 3.3.2 Conditional densities
    - 3.3.3 Competing risk adjustment
    - 3.3.4 Encoding of events
    - 3.3.5 Probabilities of each state
      - 3.3.5.1 Death without complete remission
      - 3.3.5.2 Complete remission
      - 3.3.5.3 Alive in induction
      - 3.3.5.4 Non-relapse deaths
      - 3.3.5.5 Alive in complete remission
      - 3.3.5.6 Post-relapse death
      - 3.3.5.7 Alive after relapse
    - 3.3.6 Comments
  - 3.4 Confidence intervals
    - 3.4.1 Marginal probabilities
    - 3.4.2 Survival after remission
      - 3.4.2.1 Analytical confidence intervals
      - 3.4.2.2 Simulated

        - 3.4.3 Differential survival
        - 3.4.4 Overall survival from diagnosis
    - 3.5 Measures of absolute prediction errors
    - 3.6 Code
        - 3.6.1 Static multistage model
            - 3.6.1.1 Figure 2B
        - 3.6.2 Prepare covariates
        - 3.6.3 RFX fit of transitions
            - 3.6.3.1 OS
            - 3.6.3.2 Early deaths
        - 3.6.4 Variance components
            - 3.6.4.1 Figure 2F
            - 3.6.4.2 Supplementary Tables S2-6
        - 3.6.5 Predicting outcome from diagnosis
            - 3.6.5.1 Leave-one-out cross-validation
            - 3.6.5.2 Figure 3
            - 3.6.5.3 Comparison with RFX
            - 3.6.5.4 Figure 2C
            - 3.6.5.5 Figure 2D
            - 3.6.5.6 Figure 2E
            - 3.6.5.7 Figure 1B
            - 3.6.5.8 Figure 4
            - 3.6.5.9 Supplementary Figure S2
        - 3.6.6 Predicting outcome after CR
            - 3.6.6.1 Model assessment
            - 3.6.6.2 Absolute survival probabilities
            - 3.6.6.3 Allogeneic hematopoietic stem cell transplants
            - 3.6.6.4 Leave one out cross-validation
            - 3.6.6.5 Best treatment options
            - 3.6.6.6 Three patients with numerical CI's and LOO
            - 3.6.6.7 LOO predictions of HSCT with CI's accounting for correlation
            - 3.6.6.8 Figure 5A
            - 3.6.6.9 Figure 5B
            - 3.6.6.10 Figure 5C
            - 3.6.6.11 Supplementary Figure S3
            - 3.6.6.12 Figure 5D
            - 3.6.6.13 Supplementary Figure S4
            - 3.6.6.14 Prediction errors
        - 3.6.7 Imputation of missing genes
            - 3.6.7.1 RFX model on OS
            - 3.6.7.2 Genetic imputation multi stage
            - 3.6.7.3 Supplementary Figure S6B
- 4 Model comparison
    - 4.1 Random cross validation
    - 4.2 Inter-trial
    - 4.3 TCGA
    - 4.4 Code
        - 4.4.1 Systematic cross-validation
            - 4.4.1.1 Static models
            - 4.4.1.2 Different RFX models
            - 4.4.1.3 Time-dependent models
        - 4.4.2 Inter-study CV
            - 4.4.2.1 Time-dependent
        - 4.4.3 TCGA validation
            - 4.4.3.1 Fit models
            - 4.4.3.2 TCGA data
            - 4.4.3.3 Analyse risk
        - 4.4.4 Multistage models
            - 4.4.4.1 Figure 1C
            - 4.4.4.2 Figure 1A
- 5 Simulations
    - 5.1 Survival
    - 5.2 Interpolations
        - 5.2.1 Subsampling of genes
        - 5.2.2 Subsampling of patients
    - 5.3 Extrapolations
        - 5.3.1 Patients
        - 5.3.2 Genes
            - 5.3.2.1 TCGA
    - 5.4 Code
        - 5.4.1 Interpolations
            - 5.4.1.1 Figure 6A
            - 5.4.1.2 Supplementary Figure S6A
        - 5.4.2 Extrapolations
            - 5.4.2.1 Supplementary Figure S7B
            - 5.4.2.2 Supplementary Figure S7A
            - 5.4.2.3 Figure 6C
            - 5.4.2.4 Figure 6B

- 5.4.3 Multistage simulations
  - 5.4.3.1 Simulation function
  - 5.4.3.2 Simulate outcomes
  - 5.4.3.3 Estimation based on simulated data
  - 5.4.3.4 HSCTs
- 6 Web tool
  - 6.1 Code
    - 6.1.1 Data
    - 6.1.2 server.R
    - 6.1.3 ui.R
- 7 R session

A machine readable version of this document and associated data can be found at github.com/mg14/AML-multistage (http://www.github.com/mg14/AML-multistage).

1 Data

1.1 Variables

We use the data from N=1,540 AML cases as described in our companion paper (Papaemmanuil et al. 2015).

These can be summarised as follows:

Table 1. Variables

Group	Symbol	p	Variables
Fusion genes	Fusions	8	inv3_t3_3, t_9_22, t_15_17, t_8_21, inv16_t16_16, t_6_9, t_9_11, t_v_11
Copy number alterations	CNA	18	minus5_5q, minus7, minus7q, abn7other, plus8_8q, minus9q, mono12_12p_abn12p, plus13, mono17_17p_abn17p, minus18_18q, minus20_20q, plus21, plus22, minusY, abn3q_other, plus11_11q, mono4_4q_abn4q, complex
Point mutations in single genes	Genetics	58	ASXL1, ATRX, BCOR, BRAF, CBL, CBLB, CDKN2A, CREBBP, CUX1, DNMT3A, EP300, ETV6, EZH2, FBXW7, GATA2, GNAS, IDH1, IKZF1, JAK2, KDM5A, KDM6A, KIT, KRAS, MLL, MLL2, MLL3, MLL5, MPL, MYC, NF1, NPM1, NRAS, PHF6, PRPF40B, PTEN, PTPN11, RAD21, RB1, RUNX1, SF1, SF3A1, SF3B1, SFRS2, SH2B3, STAG2, TET2, TP53, U2AF1, U2AF2, WT1, ZRSR2, CEBPA_mono, CEBPA_bi, FLT3_ITD, FLT3_TKD, FLT3_other, IDH2_p172, IDH2_p140
Gene:Gene product terms	Gene:Gene	126	BCOR:DNMT3A, ASXL1:EZH2, DNMT3A:IDH1, DNMT3A:KRAS, DNMT3A:MLL, IDH1:MLL, DNMT3A:MYC, DNMT3A:NF1, CBL:NPM1, DNMT3A:NPM1, GATA2:NPM1, IDH1:NPM1, KIT:NPM1, KRAS:NPM1, MYC:NPM1, NF1:NPM1, ASXL1:NRAS, BCOR:NRAS, DNMT3A:NRAS, EZH2:NRAS, GATA2:NRAS, IDH1:NRAS, KIT:NRAS, KRAS:NRAS, MLL:NRAS, NPM1:NRAS, NPM1:PHF6, DNMT3A:PTPN11, IDH1:PTPN11, KRAS:PTPN11, NPM1:PTPN11, NRAS:PTPN11, DNMT3A:RAD21, NPM1:RAD21, NRAS:RAD21, PTPN11:RAD21, ASXL1:RUNX1, BCOR:RUNX1, DNMT3A:RUNX1, EZH2:RUNX1, IDH1:RUNX1, MLL:RUNX1, NRAS:RUNX1, PHF6:RUNX1, NRAS:SF3B1, ASXL1:SFRS2, DNMT3A:SFRS2, IDH1:SFRS2, NPM1:SFRS2, NRAS:SFRS2, RUNX1:SFRS2, ASXL1:STAG2, DNMT3A:STAG2, EZH2:STAG2, MLL:STAG2, NPM1:STAG2, NRAS:STAG2, RUNX1:STAG2, SFRS2:STAG2, ASXL1:TET2, DNMT3A:TET2, KIT:TET2, MLL:TET2, NPM1:TET2, NRAS:TET2, PTPN11:TET2, RUNX1:TET2, SFRS2:TET2, STAG2:TET2, DNMT3A:TP53, NRAS:TP53, NRAS:U2AF1, NPM1:WT1, NRAS:WT1, DNMT3A:CEBPA_mono, NPM1:CEBPA_mono, TET2:CEBPA_mono, GATA2:CEBPA_bi, NRAS:CEBPA_bi, WT1:CEBPA_bi, DNMT3A:FLT3_ITD, EZH2:FLT3_ITD, IDH1:FLT3_ITD, MLL:FLT3_ITD, MYC:FLT3_ITD, NPM1:FLT3_ITD, NRAS:FLT3_ITD, PHF6:FLT3_ITD, PTPN11:FLT3_ITD, RAD21:FLT3_ITD, RUNX1:FLT3_ITD, STAG2:FLT3_ITD, TET2:FLT3_ITD, WT1:FLT3_ITD, CEBPA_mono:FLT3_ITD, CEBPA_bi:FLT3_ITD, DNMT3A:FLT3_TKD, IDH1:FLT3_TKD, MLL:FLT3_TKD, NPM1:FLT3_TKD, NRAS:FLT3_TKD, RAD21:FLT3_TKD, RUNX1:FLT3_TKD, TET2:FLT3_TKD, WT1:FLT3_TKD, FLT3_ITD:FLT3_TKD, DNMT3A:FLT3_other, NPM1:FLT3_other, NRAS:FLT3_other, PTPN11:FLT3_other, RUNX1:FLT3_other, TET2:FLT3_other, FLT3_ITD:FLT3_other, DNMT3A:IDH2_p172, ASXL1:IDH2_p140, DNMT3A:IDH2_p140, MLL:IDH2_p140, NPM1:IDH2_p140, NRAS:IDH2_p140, PTPN11:IDH2_p140, RUNX1:IDH2_p140, SFRS2:IDH2_p140, STAG2:IDH2_p140, FLT3_ITD:IDH2_p140, FLT3_TKD:IDH2_p140, NPM1:FLT3_ITD:DNMT3A
Clinical parameters	Clinical	11	Performance_ECOG, BM_Blasts_100, PB_Blasts_100, wbc_100, LDH_1000, HB_10, platelet_100, Splenomegaly, oAML, sAML, tAML
Demographical variables	Demographics	2	AOD_10, gender
Treatment	Treatment	4	ATRA, VPA, HSCT in CR1, HSCT after relapse
Nuisance terms	Nuisance	4	AMLHD98A, AMLHD98B, Date_1000, MissingCyto

Note that *Point mutations in single genes* also includes *FLT3* intra-tandem duplications (ITD), as well as *MLL* partial tandem duplications (PTD), which are large indels.

1.2 Preprocessing

The following preprocessing steps were applied:

- Agnostic imputation of missing variables by mean.
- Quantitative variables linearly rescaled by a power of 10 to a magnitude of 1. This is necessary, as we are working with a penalty on the

coefficient size.

1.2.1 Genomic variables

Fusion genes, Copy number alterations, Genetics and Gene:Gene interactions were encoded as 0 (absent) and 1 (present) based on the same annotation as in (Papaemmanuil et al. 2015). A gene was considered mutated and encoded as 1 if it contained at least one oncogenic mutation, and 0 otherwise. In addition, we used the following rules:

- For *CEBPA* we additionally differentiated mono- and bi-allelic lesions.
- For *FLT3* we distinguished between ITD, TKD and other mutations.
- For *IDH2* we separately encoded p172 and p140 point mutations.

1.2.2 Gene:gene product terms

Gene:Gene product terms were computed indicating whether a combination of two genes was present. This allows to account for non-additive genetic interaction. To limit the number of variables, product terms included if there were at least 8 occurrences.

1.2.3 Clinical and demographic variables

Quantitative clinical variables were rescaled to a magnitude of 1 as described above. To assess the validity of out log-linear risk model we computed spline fits, that allow for a non-linear dependence between log-hazard and each variable. We did not observe a measurable improvement of our model fits in cross-validation.

1.2.4 Treatment

The following variables were included in the model:

1. Allograft (MRD, MUD) in CR1 as a time-dependent covariate. Not considered for RFS, CPSS (no time-dependence allowed)
2. Allograft (MRD, MUD) after relapse as a time-dependent covariate. Multi-stage model only.
3. Extra cycles of ATRA encoded as 0/1.
4. VPA (AMLSG 07/04 only).

1.2.5 Nuisance

In addition to the aforementioned explanatory variables we used the following multiplicative strata to account for potential confounding factors:

1. Missing cytogenetic information (0/1). We observed that cytogenetic data was missing more frequently for patients having died early. To avoid a negative bias we included this as an additional factor
2. Trial (2/3), AMLSG07/04, AMLHD98A, AMLHD98B. A factor was included to account for systematic differences between trials.
3. Date. The date of diagnosis was included to account for and improvement of patient care over time.

1.3 Code

1.3.1 Libraries

Load a few libraries - see end of document for a full list of libraries and their versions.

```
library(CoxHD)
library(mg14)
set1 <- brewer.pal(9, "Set1")
```

1.3.2 Raw data

1.3.2.1 Clinical data

We use the following steps for processing of the original data. Note that, for privacy reasons, we cannot distribute clinical data with the actual event dates and instead provide these data in an anonymised form.

Hence one should skip the following two chunks
Start from ***load("../data/AMLSG_Clinical_Anon.RData")***

```
clinicalData <- read.table("../data/AMLSG_Clinical.txt", sep="\t", header=TRUE, na.strings = "na", comment.char =
"", quote="\"")
clinicalData <- clinicalData[order(clinicalData$PDID),]
clinicalData$ERDate <- as.Date(as.character(clinicalData$ERDate), "%d-%b-%y")
clinicalData$CR_date <- as.Date(as.character(clinicalData$CR_date), "%d-%b-%y")
clinicalData$TPL_date <- as.Date(as.character(clinicalData$TPL_date), "%d-%b-%y")
clinicalData$Date_LF <- as.Date(as.character(clinicalData$Date_LF), "%d-%b-%y")
clinicalData$Recurrence_date <- as.Date(as.character(clinicalData$Recurrence_date), "%d-%b-%y")
levels(clinicalData$Study) <- c(`_07-04`="AMLSG0704" ,  `98A`="AMLHD98A" ,  `98B`="AMLHD98B")[levels(clinicalDat
a$Study)]
clinicalData$Study <- factor(as.character(clinicalData$Study))
clinicalData$VPA[is.na(clinicalData$VPA)] <- 0
clinicalData$ATRA_arm[is.na(clinicalData$ATRA_arm)] <- 0
colnames(clinicalData) <- gsub('\\.',"",colnames(clinicalData))
clinicalData <- clinicalData[!is.na(clinicalData$TypeAML),] ## remove unknown patients
clinicalData$PDID <- factor(as.character(clinicalData$PDID))
t <- read.table("../data/AMLSG_Karyotypes.txt", header=T, sep="\t", na.strings = "na",comment.char = "", quote="\
"")
karyotypes <- t$karyotype[match(clinicalData$PDID,t$PDID)]
rm(t)
clinicalData$t_9_11 <- grepl("t\\(9;11\\)\\(p22;q23\\)", karyotypes) + 0 # t(9;11)
clinicalData$t_v_11 <- clinicalData$t_MLL &! clinicalData$t_9_11
clinicalData$t_MLL <- NULL

dim(clinicalData)
```

```
## [1] 1540  106
```

Here we store the data in an anonymised form, where all references to the actual date of diagnoses are removed. These data are available on github.

```
e <- clinicalData$ERDate
clinicalData$ERDate <- clinicalData$ERDate - e
clinicalData$CR_date <- clinicalData$CR_date - e
clinicalData$Date_LF <- clinicalData$Date_LF - e
clinicalData$TPL_date <- clinicalData$TPL_date - e
clinicalData$Recurrence_date <- clinicalData$Recurrence_date - e
save(clinicalData, file="../data/AMLSG_Clinical_Anon.RData")
```

Load the data using

```
load("../data/AMLSG_Clinical_Anon.RData")
```

1.3.2.2 Mutation data

```
mutationData = read.table("../data/AMLSG_Genetic.txt", sep="\t", header=TRUE, strip.white = TRUE)
mutationData$SAMPLE_NAME <- factor(as.character(mutationData$SAMPLE_NAME), levels = levels(clinicalData$PDID)) ##
Refactor
mutationTable <- (table(mutationData[mutationData$Result %in% c("ONCOGENIC","POSSIBLE") & mutationData$FINAL_CALL
== "OK" ,c("SAMPLE_NAME","GENE")]) > 0)+0
dim(mutationTable)
```

```
## [1] 1540   54
```

```
all(rownames(mutationTable)==clinicalData$PDID)
```

```
## [1] TRUE
```

1.3.3 Survival data

```
os <- Surv(clinicalData$OS, clinicalData$Status) #OS
t <- clinicalData$Time_Diag_TPL # In this Note, variables Time_Diag_TPL and TPL_date both denote time (days) from diagnosis to allograft
t[is.na(t) | !clinicalData$TPL_Phase %in% "CR1" | !clinicalData$TPL_type %in% c("ALLO","FREMD") ] <- Inf ## Only
allografts in CR1
o <- clinicalData$OS
tplIndexOs <-  t < o
osTD <-  Surv(time = rep(0, nrow(clinicalData)), time2=pmin(o, t), event=ifelse(tplIndexOs, 0, clinicalData$Statu
s) )
osTD <- rbind(osTD,
        Surv(time=t[which(tplIndexOs)],
                time2=o[which(tplIndexOs)],
                event=clinicalData$Status[which(tplIndexOs)])
)
osTD = Surv(osTD[,1],osTD[,2],osTD[,3])
rm(o,t)
tplSplitOs <- c(1:nrow(clinicalData), which(tplIndexOs))
osYr <- os
osYr[,1] <- osYr[,1]/365
osYrTD <- osTD
osYrTD[,1] <- osYrTD[,1]/365
```

1.3.4 Covariates

All data as list

```
dataList <-list(Genetics = data.frame(mutationTable[,colSums(mutationTable)>0]),
        Cytogenetics = clinicalData[,grep("^(t_)|(inv)|(abn)|(plus)|(minus)|(mono)|(complex)",colnames(clinicalDa
ta))],
        Nuisance = data.frame( MakeInteger(clinicalData$Study)[,1:2], Date=scale(as.numeric(clinicalData$ERDate),
scale=FALSE), MissingCyto=is.na(clinicalData$t_15_17)+0),
        Treatment = data.frame(ATRA = clinicalData$ATRA_arm, VPA=clinicalData$VPA, TPL_os=tplIndexOs),
        Demographics = clinicalData[,c("AOD","gender")],
        Clinical = cbind(clinicalData[, c("Performance_ECOG","BM_Blasts","PB_Blasts","wbc","LDH","HB","platelet",
"Splenomegaly")], MakeInteger(clinicalData$TypeAML)[,-1]))#,
#MolRisk = makeInteger(clinicalData$M_Risk))
#dataList$Genetics$CEBPA <-  clinicalData$CEBPA # encoded as 0,1,2
dataList$Genetics$CEBPA_mono <-  clinicalData$CEBPA == 1 # encoded as 0,1,2
dataList$Genetics$CEBPA_bi <-  clinicalData$CEBPA == 2 # encoded as 0,1,2
dataList$Genetics$CEBPA <- NULL
dataList$Genetics$FLT3 <- NULL
dataList$Genetics$FLT3_ITD <- clinicalData$FLT3_ITD != "0"
dataList$Genetics$FLT3_TKD <- clinicalData$FLT3_TKD != "0"
dataList$Genetics$FLT3_other <- clinicalData$FLT3_other != "0"
dataList$Genetics$IDH2_p172 <- table(mutationData$SAMPLE_NAME[mutationData$GENE=='IDH2' & grepl("172", mutationDa
ta$AA_CHANGE)])[]
dataList$Genetics$IDH2_p140 <-  table(mutationData$SAMPLE_NAME[mutationData$GENE=='IDH2' & grepl("140", mutationD
ata$AA_CHANGE)])[]
dataList$Genetics$IDH2 <- NULL
dataList$Genetics$NPM1 <- clinicalData$NPM1
dataList$Cytogenetics$MLL_PTD <- NULL
dataList$Genetics = dataList$Genetics + 0
dataList$GeneGene <- MakeInteractions(data.frame(dataList$Genetics), data.frame(dataList$Genetics))[,as.vector(up
per.tri(matrix(0,ncol=ncol(dataList$Genetics), nrow=ncol(dataList$Genetics))))]
dataList$GeneGene <- dataList$GeneGene[,colSums(dataList$GeneGene, na.rm=TRUE)>0]
dataList$GeneGene$`NPM1:FLT3_ITD:DNMT3A` <- (rowSums(dataList$Genetics[c('NPM1',"FLT3_ITD","DNMT3A")])==3)+0 ## A
dd NPM1:FLT3_ITD:DNMT3A product term as well
dataList$CytoCyto <- MakeInteractions(dataList$Cytogenetics, dataList$Cytogenetics)[,sapply(1:ncol(dataList$Cytog
enetics), `<`, 1:ncol(dataList$Cytogenetics))]
dataList$CytoCyto <- dataList$CytoCyto[, colSums(dataList$CytoCyto, na.rm=TRUE) > 0]
dataList$GeneCyto <- MakeInteractions(dataList$Genetics, dataList$Cytogenetics)
dataList$GeneCyto <- dataList$GeneCyto[,colSums(dataList$GeneCyto, na.rm=TRUE) > 0]
dataList$GeneTreat <- MakeInteractions(dataList$Genetics, dataList$Treatment)
dataList$GeneTreat <- dataList$GeneTreat[,colSums(dataList$GeneTreat, na.rm=TRUE) > 0]
dataList$CytoTreat <- MakeInteractions(dataList$Cytogenetics, dataList$Treatment)
dataList$CytoTreat <- dataList$CytoTreat[,colSums(dataList$CytoTreat, na.rm=TRUE) > 0]
```

Error in FUN(X[[i]], ...) : non-numeric argument to binary operator
In addition: There were 50 or more warnings (use warnings() to see the first 50)
Two new factor variables are unexpectedly created in ***dataList$Genetics*** by the first command
[Correction] Change the first command to: ***dataList$Genetics[,52:58]<-as.data.frame(lapply(dataList$Genetics[,52:58], as.numeric))***

Condensing to a data.frame

```
dataRaw <- do.call(cbind,dataList)
names(dataRaw) <- unlist(sapply(dataList, names))
dataFrame <- StandardizeMagnitude(dataRaw)
dim(dataFrame)
```

```
## [1] 1540 1827
```

```
groups <- unlist(sapply(names(dataList), function(x) rep(x, ncol(dataList[[x]]))))
groups[grepl("^(t_)|(inv)", colnames(dataFrame)) &! grepl(":", colnames(dataFrame))] <- "Fusions"
groups[groups=="Cytogenetics"] <- "CNA"
groups <- factor(groups)
names(groups) <- colnames(dataFrame)
table(groups)
```

```
## groups
##          CNA     Clinical     CytoCyto    CytoTreat Demographics      Fusions     GeneCyto     GeneGene    Gen
eTreat
##           18           11          183           70            2            8          606          721
143
##     Genetics     Nuisance    Treatment
##           58            4            3
```

Poor man's imputation by column means

```
poorMansImpute <- function(x) {x[is.na(x)] <- mean(x, na.rm=TRUE); return(x)}
dataFrame <- as.data.frame(sapply(dataFrame, poorMansImpute))
rownames(dataFrame) <- clinicalData$PDID
```

1.3.5 Subclonal mutations

```
copyNumbers = cbind(dataList$Cytogenetics[grep(c("minus|plus|mono"), colnames(dataList$Cytogenetics))], clinicalD
ata$gender)
copyNumbers$minus7 <- (copyNumbers$minus7 | copyNumbers$minus7q) +0
copyNumbers$minus7q <- NULL
for(i in 1:ncol(copyNumbers)){
    if(grepl("plus", colnames(copyNumbers)[i]))
        copyNumbers[,i] = copyNumbers[,i] * 3
}
copyNumbers[copyNumbers==0 | is.na(copyNumbers)] = 2
colnames(copyNumbers) = c(5,7,8,9,12,13,17,18,20,21,22,"Y",11,4,"X")
rownames(copyNumbers) <- clinicalData$PDID
copyNumbers$Y <- c(1:0)[clinicalData$gender] - mg14::na.zero(dataList$Cytogenetics$minusY)

cn = sapply(1:nrow(mutationData), function(i) {c=copyNumbers[mutationData$SAMPLE_NAME[i],match(mutationData$CHR[i
], colnames(copyNumbers))]; if(length(c)==0) 2 else c})
vaf <- as.numeric(as.character(mutationData$X._MUT_IN_TUM))
```

```
## Warning: NAs introduced by coercion
```

```
depth <- as.numeric(as.character(mutationData$TUM_DEPTH))
```

```
## Warning: NAs introduced by coercion
```

```
dataFLT3_ITD <- read.table("../data/AMLSG_FLT3ITD.txt", sep="\t", header=TRUE)
dataFLT3_ITD$Sample <- sub("WGA_","", dataFLT3_ITD$Sample)

mcf <- vaf/100*cn ## Approx mutant cell fraction, assuming mutations on only one copy
mcf[which(mcf > 1.25)] <- vaf[which(mcf > 1.25)] ## Probably over adjusted
mcf[mcf > 1] <- 1 ## Random fluctuations
genesClonal <- dataFrame[groups=="Genetics"]
precedence <- matrix(0, nrow=ncol(genesClonal), ncol = ncol(genesClonal) , dimnames=list(colnames(genesClonal), c
olnames(genesClonal)))
plist <- list()
lesions <- as.character(mutationData$GENE)
lesions[mutationData$GENE=='IDH2' & grepl("172", mutationData$AA_CHANGE)] <- "IDH2_p172"
lesions[mutationData$GENE=='IDH2' & grepl("140", mutationData$AA_CHANGE)] <- "IDH2_p140"
lesions[mutationData$GENE=='FLT3' & grepl(paste(835:841, collapse="|"), mutationData$AA_CHANGE)] <- "FLT3_TKD"
lesions[mutationData$GENE=='FLT3' & grepl("ITD", mutationData$AA_CHANGE)] <- "FLT3_ITD"
lesions[lesions=="FLT3"] <- "FLT3_other"

# Add FLT3_ITD VAF, not the most accurate presumably, due to mapping problems for ITDs..
i <- lesions == "FLT3_ITD"
m <- match(mutationData$SAMPLE_NAME[i], dataFLT3_ITD$Sample)
mcf[i] <- as.numeric(as.character(dataFLT3_ITD$Read_count[m]))/dataFLT3_ITD$Coverage[m]
```

```
## Warning: NAs introduced by coercion
```

```
depth[i] <- dataFLT3_ITD$Coverage[m]

ix= lesions %in% colnames(precedence) & mutationData$Result %in% c("ONCOGENIC","POSSIBLE")
for(s in clinicalData$PDID){
    l <- list()
    for(i in which(mutationData$SAMPLE_NAME==s & ix))
        for(j in which(mutationData$SAMPLE_NAME==s & ix)){
            if(!is.na(cn[i]) & !is.na(cn[j]) & i!=j){
                m <- round(matrix(c(
                                        mcf[i]*depth[i],
                                        depth[i]-mcf[i]*depth[i],
                                        mcf[j]*depth[j],
                                        depth[j]-mcf[j]*depth[j]),
                                ncol=2))
                f <- try(fisher.test(m, alternative="greater")$p.value< 0.01 , silent=TRUE) ## Fisher test
                if(class(f)!="try-error")
                    if(f & mcf[i] >= 1 - mcf[j]){ ## Pidgeonhole
                        precedence[as.character(lesions[i]),as.character(lesions[j])] <- precedence[as.character(
lesions[i]),as.character(lesions[j])] + 1
                        l <- c(l, list(c(as.character(lesions[i]),as.character(lesions[j]))))
                        genesClonal[s, as.character(lesions[i])] <- 2
                        genesClonal[s, as.character(lesions[j])] <- 3
                    }

            }
        }
    plist[[s]] <- l
}
```

```
t <- table(sapply(plist, length)>0)
pie(t, labels=paste(t, c("clonal/NA","polyclonal")), col=set1[2:1])
```

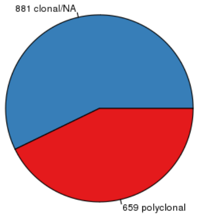

1.3.5.1 Bradley-Terry Model

```
makeDesign <- function(I) {
    w <- which(lower.tri(I), arr.ind=TRUE)
    x <- matrix(0, nrow(w), nrow(I))
    for(i in 1:nrow(w)){
        x[i,w[i,1]] <- 1
        x[i,w[i,2]] <- -1
    }
    return(x)
}

btModel <- function(I){
    y <- cbind(I[lower.tri(I)], t(I)[lower.tri(I)])
    x <- makeDesign(I = I)
    glm.fit(x=x[,-1],y=y, family=binomial())
}

nCasesGene <- table(factor(unlist(sapply(plist, function(x) unique(unlist(x)))), levels=colnames(precedence)))
w <- which(nCasesGene > 5)

fit <- btModel(precedence[w,w]+.01)
```

```
## Warning: non-integer counts in a binomial glm!
```

```
c <- c(0,coef(fit))
names(c) <- colnames(precedence)[w]
o <- rank(c)
v <- pmin(2,sqrt(c(0,diag(chol2inv(fit$qr$qr)))))
```

```
l <- names(c)
m <- paste("n=",nCasesGene[w], sep="")
plot(-c, o, xlab="Relative time", yaxt="n", pch=19, col="grey", ylab="", xlim=range(-c+3*c(-v,v)))
segments(-c-v, o,-c+v,o, col="grey")
text(-c-v ,o,l, font=3, pos=2)
text(-c+v ,o,m, font=1, pos=4)
```

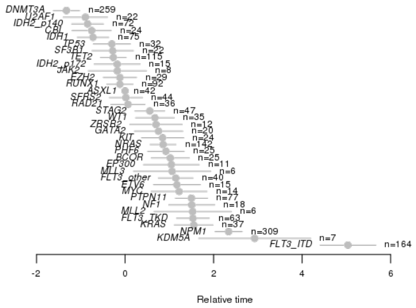

1.3.5.2 Supplemenary Figure S8

' Here we generate a panel overview of all genetic lesions and their impact on outcome, split by clonal and subclonal status.

```
par(mfrow=c(8,8), mar=c(1.5,2.5,1.5,0.5), mgp=c(2,0.5,0), bty="L", xpd=TRUE, las=1, tcl=-0.2, cex.axis=1.25)
for(g in colnames(genesClonal)){
    p <- try(pchisq(survdiff(osYr ~ genesClonal[,g] == 3, subset=genesClonal[,g]>0)$chisq,1,lower.tail=FALSE))
    plot(survfit(osYr ~ factor(genesClonal[,g], levels=0:3)), col=set1[c(9,c(4,2,1))], mark=NA, xlim=c(0,5))
    mtext(side=3, paste0(g, ifelse(class(p)!="try-error",mg14::sig2star(p),"")), line=0, font=4)
}
plot.new(); par(xpd=NA)
legend("topleft", col=set1[c(9,c(2,4,1))], lty=1, c("wt","clonal","indetermined","subclonal"), cex=1.5, bty="n")
plot.new(); par(xpd=NA)
legend("topleft", c(".","*","**","***", "P (0.05, 0.1]", "P (0.01, 0.05]", "P (0.001, 0.01]", "P < 0.001"), ncol=
2, cex=1.5, bty="n", text.width= 0.1)
```

par(mfrow=c(1,1)) # to escape the 8x8 plotting matrix

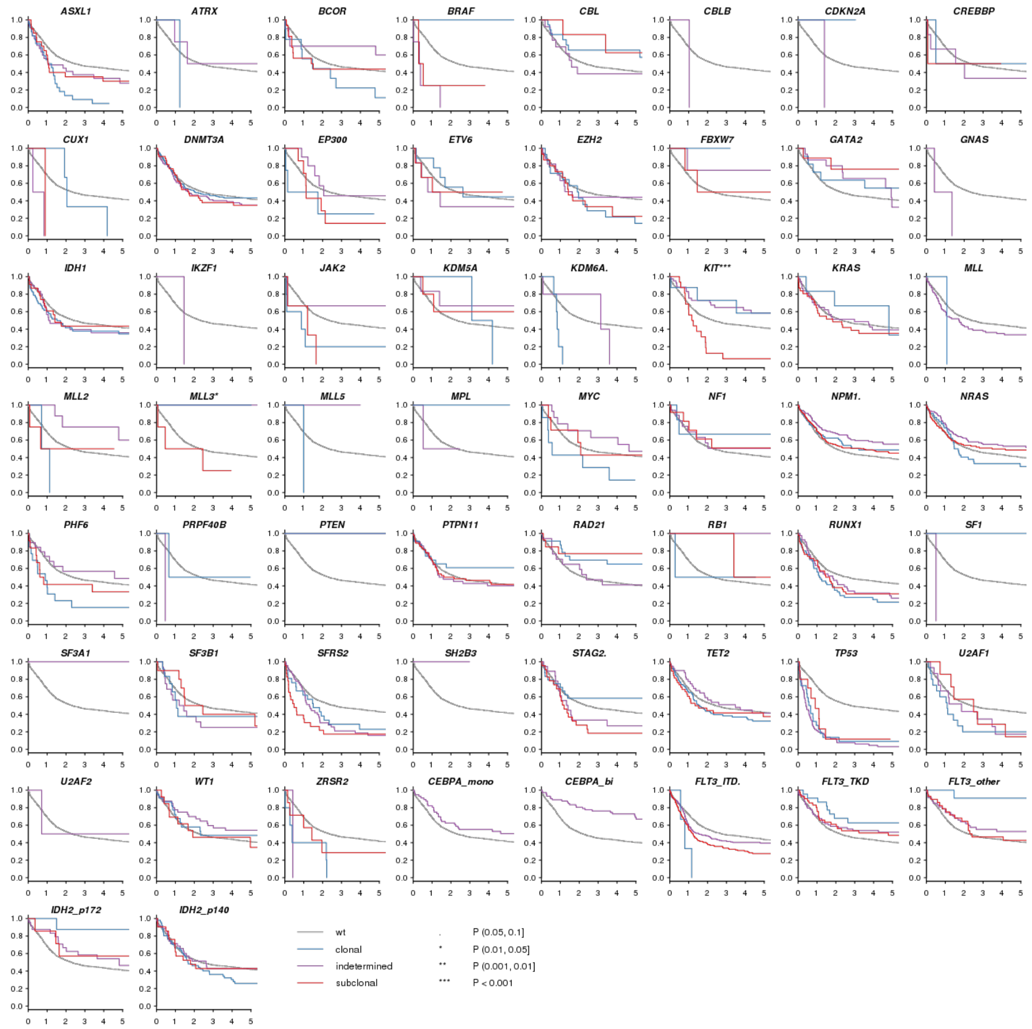

2 Models for overall survival

We use overall survival, measured from date of diagnosis, as the endpoint.

2.1 Random effects modelling

We implemented sparse random effects for the Cox proportional hazards model in the `CoxHD` R package available at [http://github.com/mg14/CoxHD (http://github.com/mg14/CoxHD)]. This implementation can handle constant covariate and time-dependent models. The latter is important to quantify the effects of allografts, which are typically administered well after diagnosis. `CoxHD::CoxRFX()`

Let the hazard be:

$$\lambda = \lambda_0(t)\exp(u^T Z) \tag{1}$$

Define $h = u^T Z$ as the log hazard. $\lambda_0(t)$ is the normal baseline hazard in a `coxph` model.

The random effects model used here is an example of a hierarchical model with an additional assumption about the distribution of the parameters u. We assume that these follow a normal distributions. This additional assumption leads to a ridge-type regularisation of the log-likelihood.

Let there be p covariates and $\{g\}$ be a partitioning of the p variables into $|g|$ groups. For each group assume that the parameters u_j are iid Normally distributed in each group:

$$\forall j \in g : u_j \sim \mathrm{N}(\mu_g; \sigma_g^2) \qquad iid. \tag{2}$$

The shared means μ_g are motivated by the observation that the effect of oncogenic lesions is, on average, deleterious.

We use the convention that variables without indexes refer to the set of variables. In particular $u = \{u_j : j = 1, \ldots, p\}$, $u_g = \{u_j : j \in g\}$.

The full logarithmic likelihood reads:

$$\ell(u, \sigma^2, \mu; Z) = \ell_0(u; Z) - \sum_g \frac{\sum_{j \in g}(u_j - \mu_g)^2}{\sigma_g^2} \tag{3}$$

$$= \ell_0(u; Z) + \ell_2(u, \mu, \sigma^2). \tag{4}$$

The term $\ell_0(u)$ is the likelihood of an unpenalised coxph model. The second term is a sum of ridge penalties resulting from the constraints imposed by the normal distribution of u, which penalises large values of $u_j - \mu_g$ with strength $1/\sigma_g$.

Note that the likelihood can be reparametrised by introducing the auxiliary variables $z_g = \sum_{j \in g} Z_j$ and the centred effects $u_j = u_j - \mu_g$:

$$\ell(u, \sigma^2, \mu; Z) = \ell_0(u, \mu; Z, z) + \ell_2(u, \sigma^2) =: \ell(u, \mu, \sigma^2; Z) \tag{5}$$

2.1.1 Implementation

All of the following steps are implemented in the `CoxHD R` package, available at [http://www.github.com/mg14/CoxHD (http://www.github.com/mg14/CoxHD)] It can be installed using the `devtools::install_github("mg14/CoxHD/CoxHD")`. The implementation makes heavy use of the `survival` package (T. Therneau 2014). The implementation is about 100x faster than the `coxme R` package for mixed effects Cox models by T. Therneau (2012), as it exploits that u are iid.

2.1.2 Parameter estimation

We use an EM algorithm as suggested by Perperoglou (2014) for Cox models, based on the work by Schall (1991). The algorithm iteratively estimates the following quantities:

1. Given $\hat{\sigma}^2$, jointly estimate

   1.1. the **shared means** $\hat{\mu}_g$ as the effect of the auxiliary variables z_g.

   1.2. the **centred variables** $\hat{u}$ as a ridge estimate,

$$\hat{\mu}, \hat{u} = \arg\max \ell(u, \mu, \hat{\sigma}^2; Z) \tag{6}$$

2. Given $\hat{\mu}$ and $\hat{u}$ the **variances** are estimated as:

$$\hat{\sigma}_g^2 = \sum_{j \in g} \hat{u}_j^2 / df_g, \qquad df_g = \mathrm{tr}[\mathcal{I}_g g H_{gg}^{-1}], \tag{7}$$

   where H is the Hessian matrix of the penalised model and $\mathcal{I}$ the observed Fisher information of the unpenalised model (each evaluated for variables of group g).

Iterate until convergence of parameters and penalised likelihood.

The final parameters are given by uncentering $\hat{u}_j = \hat{u}_j + \hat{\mu}_g$.

Note: There estimates $\hat{u}$ are maximum a posteriori (MAP) from a Bayesian interpretation with $\hat{\sigma}^2$ and $\hat{\mu}$ being empirical Bayes estimates.

2.1.3 Semiparametric bootstrap

To assess the sampling distributions of our estimates, e.g., to assess the their variances, we use the following semi-parametric bootstrap approach:

For i=1:100 simulate n semiparametric survial times `Y` (see [#survival]):

- Using MAP estimates $\hat{u}$
- Using full covariate set Z

This allows to assess the distribution of all estimates in a semi-parametric way.

2.1.4 Analytical confidence intervals of individual parameters

Two estimates exists for the covariance matrices of the parameters $\hat{u}$ and μ(T. M. Therneau, Grambsch, and Pankratz 2003):

1. $\hat{V}_1 = H^{-1}$
2. $\hat{V}_2 = H^{-1}\mathcal{I}H^{-1}$, where H is the Hessian matrix of the penalised model and $\mathcal{I}$ the observed Fisher information of the unpenalised model. Semi-parametric bootstrap simulations show that V_2 is more accurate in our context.

The estimates $\hat{V}_.$ have dimension $(p + |g|) \times (p + |g|)$.

The uncentered variance estimates of the parameter $u_j = u_j + \mu_g$ are given by

$$\hat{V}_.[u_j] = \hat{V}_.[u_j, u_j] + \hat{V}_.[\mu_g, \mu_g] + 2\hat{V}_.[u_j, \mu_g], \tag{8}$$

thus accounting for the correlation of u_j and μ_g.

2.1.5 Wald test of individual parameters

Using variance estimate $\hat{V}_2$, allows for computing a Wald-type test with one degree of freedom.

$$z = \hat{u}^2 / \hat{V}_2[u] \tag{9}$$
$$\tag{10}$$
$$Z \sim \chi_1^2 \tag{11}$$

This is implemented as `CoxHD::WaldTest()`

P-values of each test are corrected for multiple testing. Due to dependence imposed by the shared distribution we use the Benjamini-Yektuieli method for controlling the false discovery rate (Q < FDR), implemented as `p.adjust(x,method="BY")`.

Note: There exists a lively debate about how, and if at all, random effects shall be tested or not, see [http://glmm.wikidot.com/faq (http://glmm.wikidot.com/faq)] or [https://stat.ethz.ch/pipermail/r-sig-mixed-models/2008q2/000743.html (https://stat.ethz.ch/pipermail/r-sig-mixed-models/2008q2/000743.html)]. Here we use an approach outlined by Gray (1992), T. M. Therneau, Grambsch, and Pankratz (2003) and Wood (2013). However, it is important to check that the variances are correctly specified using a parametric bootstrap approach.

2.1.6 Variance components

2.1.6.1 Partial log hazard

In an additive model the linear predictor of the log hazard h is given by:

$$h = u^T Z = \sum_g \sum_{j \in g} u_j^T Z_j = \sum_g h_g \quad (12)$$

Where the set of g is partitioning of all covariates. We define h_g as the partial logarithmic hazard contributed by group g.

2.1.6.2 Variance components

The variance of the logarithmic hazard is given by:

$$Var[h] = \sum_{g,h} Cov(h_g, h_h) \quad (13)$$

Taking just the diagonal elements of $Cov(h_g, h_h)$ guarantees positive values, which do not necessarily add to the total variance. Using $V_g = \sum_h Cov(h_h, h_g)$ yields additive variance components, albeit at the cost of being negative in cases with strong collinearity of the components.

Variance components are implemented as `CoxHD::VarianceComponents()`.

Note: Unlike a classical mixed model V is not computed by marginalising the random effects, but by the MAP estimates. This can be seen as a first order approximation.

The standard deviation $\sqrt{Var[h]}$ determines the average difference between any two patients in logarithmic hazard.

2.1.6.3 Relation to concordance

For a normally distributed hazard, the variance σ_h^2 of the log hazard is related to the concordance metric (Gönen and Heller 2005)

$$C = \int \frac{1}{1 + \exp(-|x|)} f(x; 0, \sigma_h^2) dx \quad (14)$$

where $f(x, \mu, \sigma_2)$ is the density of normal distribution. There exists no analytical solution to the above equation, but it may be computed numerically. For a variance of 1, the concordance is 72.5%.

Note: For a Cox proportional hazards model even perfect knowledge of the hazard does not guaratuee perfect concordance (i.e. C=1) due to the sampling of the survival times. The limit $Var[h] \to \infty$, in which the hazard ratio between any two patients is infinite, yields a deterministic behaviour with $C = 1$.

2.1.7 Prediction error

The prediction error of a the log hazard for patient i is given by

$$\hat{V}[h_i] = V[\hat{u}^T Z_{i\cdot}] = Z_{i\cdot}^T \hat{V}[u] Z_{i\cdot} \quad (15)$$

where $V[\hat{u}]$ is the covariance matrix of the parameters defined in Analytical confidence intervals.

Note: In a linear model, the lhs corresponds to the the residual r_i of observation i and the identity $\hat{V} = Z^T Z \times RSS/n$ holds. In our case V is derived from the Fisher information, but it can be intuitive to think about the average prediction error $\sum_i \hat{V}[h_i]/n$ as a pseudo residual variance.

2.1.8 Covariance-based imputation

To predict the log-hazard in the presence of missing variables, we can use the following imputation, leveraging the covariance in the training set:

Suppose that $Z = (Z_o, Z_m)$, where Z_o are observed and Z_m missing parts of the data set. Suppose we know the means μ and covariance Σ. Then

$$E[Z_m] = \mu_m + \Sigma_{m,o}\Sigma_{o,o}^{-1}(Z_o - \mu_o) \quad (16)$$

$$V[Z_m] = \Sigma_{mm} - \Sigma_{mo}\Sigma_{oo}^{-1}\Sigma_{om} \quad (17)$$

2.1.8.1 Prediction error

The uncertainty in Z_m adds another term to the prediction error:

$$\hat{V}[h_i] = Z_{io}^T \hat{V}[u]_{oo} Z_{io} + u_m^T V[Z_{im}] u_m \quad (18)$$

2.2 Other survival models

2.2.1 Stepwise variable selection

Coxph + AIC or BIC forward and backward selection beginning from empty model. The implementation in the `survival` `R` package (T. Therneau 2014) handles constant covariate and time-dependent models.

2.2.2 Complementary pairs stability selection

Complementary pairs stability selection (CPSS) is an extension of the stability selection protocol, which combines subsampling and LASSO-regularised regression to obtain a robust subset of predictor variables (Meinshausen and Bühlmann 2010). Using complimentary pairs subsamples Shah and Samworth (2013) derived a tighter bound for error control.

We have recently used CPSS to analyse the association of genomic predictors and outcome in Myelodysplastic syndromes (Papaemmanuil et al. 2013). To this end, we have implemented CPSS in the `CoxHD` `R` package. Our implementation fits the CPSS model using the `glmnet` algorithm (J. Friedman, Hastie, and Tibshirani 2010, Simon et al. (2011)).

The algorithm `CoxHD::CoxCPSS()` uses the following parameters:

- Parameters
  - 50 pairs
  - Selection probability 80%
  - Penalty range chosen to conform FDR < 10%

- Refit `coxph()` with selected variables for predictions

Note that the `glmnet` algorithm cannot handle time-dependent covariates.

2.2.3 Random survival forests

Random survival forests are an intrinsically non-linear alternative to Cox proportional hazards based regression (Ishwaran et al. 2008). The idea is to fit an ensemble of regression trees based on subsampling of patients and/or covariates. The resulting predictions are averaged across the forest of regression trees. We used version 1.6 of the `randomForestSRC` package and default options for `randomForestSRC::rfsrc()`. Note that the model can only handle constant covariates.

2.3 Code

2.3.1 Number of oncogenic mutations

Construct data.frame for OS, replicating patients (rows) before and after allograft.

```
dataFrameOsTD <- dataFrame[tplSplitOs,]
dataFrameOsTD[which(tplIndexOs), grep("TPL", colnames(dataFrameOsTD), value=TRUE)] <- 0 ## Set pre-tpl variables
to zero
```

Define some indexes relating to subsets of variables used by the random effects model.

```
mainGroups <- grep("[A-Z][a-z]+[A-Z]",levels(groups), invert=TRUE, value=TRUE)
mainGroups
```

```
## [1] "CNA"           "Clinical"     "Demographics" "Fusions"      "Genetics"     "Nuisance"     "Treatment"
```

```
mainIdx <- groups %in% mainGroups
osIdx <- !grepl("TPL", colnames(dataFrame)) ## Exclude TPL from OS analyses..
whichRFXOs <- which((colSums(dataFrame)>=8 | mainIdx) & osIdx) # ie, > 0.5%
mainIdxOs <- mainIdx & osIdx
osTDIdx <- !grepl("TPL_efs", colnames(dataFrame))
whichRFXOsTD <- which((colSums(dataFrame)>=8 | mainIdx) & osTDIdx) # ie, > 0.5%
mainIdxOsTD <- mainIdx & osTDIdx
whichRFXOsGG <- which((colSums(dataFrame)>=8 | mainIdxOs) & osIdx & groups %in% c(mainGroups,"GeneGene")) # ie, >
0.5%
```

There is no variable in ***dataFrame*** containing ***"TPL_efs"*** as in the 5th code line; this has a bearing on three commands later which call the variable ***osTDIdx*** that is defined with ***"TPL_efs"***

This chunk creates column indices to subset ***DataFrame***, including ***mainIdx, mainIdxOs, mainIdxOsTD, osIdx, osTDIdx, whichRFXOs, whichRFXOsTD,*** and ***whichRFXOsGG***

However, on the one hand, these subsets seem not clearly indicated by the indices names; on the other, the Boolean combinations could have been simplified, for instance, the last code line combines ***mainIdxOs, osIdx,*** and ***mainGroups***, while ***mainIdxOs*** itself has already contained information of ***OsIdx*** and ***mainGroups***

Compute the number of oncogenics, excluding complex karyotype.

```
NONC <- rowSums(cbind(dataList$Cytogenetics[names(dataList$Cytogenetics)!="complex"], dataList$Genetics), na.rm=T
RUE)
```

2.3.1.1 Number of oncogenics

```
NONCs <- factor(ceiling(pmin(NONC,7)/2), labels=c("0","1-2","3-4","5-6","7+"))
c <- set1[c(3,2,4,1,5)]
f <- survfit(osYr ~ NONCs)
s <- summary(f)
plot(f, col=c, xlim=c(0,10),xlab="Years", ylab="Survival",mark="|", cex=.5)
legend('topright', bty='n', col=c, legend=paste0(levels(NONCs)," (n=",table(NONCs),")"), lty=1)
```

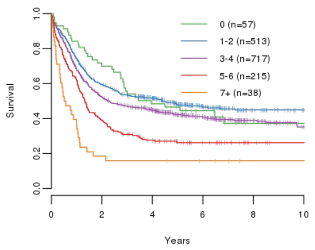

2.3.1.1.1 Linearity of continuous variables

Fit a spline through continuous covariates

```
set.seed(42)
trainIdx <- sample(c(TRUE,FALSE), nrow(dataFrame), replace=TRUE, prob=c(0.66,0.34))
trainIdxOsTD <- trainIdx[tplSplitOs]
par(mfrow=c(3,3))
clinicalSpline <- as.data.frame(sapply(dataFrame[groups %in% c("Clinical","Demographics")], function(x){
                    if(all(x[1:5] %in% 0:10)) return(x)
                    y <- log(x+min(x)+1e-3)
                    fit <- coxph(os ~ pspline(y, df=3), subset=trainIdx)
                    predict(fit, newdata=data.frame(y=y))
                })
for(n in names(clinicalSpline)) if(!all(dataFrame[1:5,n] %in% 0:10))
        plot(dataFrame[,n], clinicalSpline[,n], log='x', xlab=paste(n, '[observed]'), ylab = paste(n, '[spline]')
)
```

We suspect this should be ***log(x-min(x)+1e-3)*** to make the value in the parentheses>0

par(mfrow=c(1,1)) # to escape the 3x3 plotting matrix

```
## Warning in xy.coords(x, y, xlabel, ylabel, log): 5 x values <= 0 omitted from logarithmic plot
```

```
## Warning in xy.coords(x, y, xlabel, ylabel, log): 110 x values <= 0 omitted from logarithmic plot
```

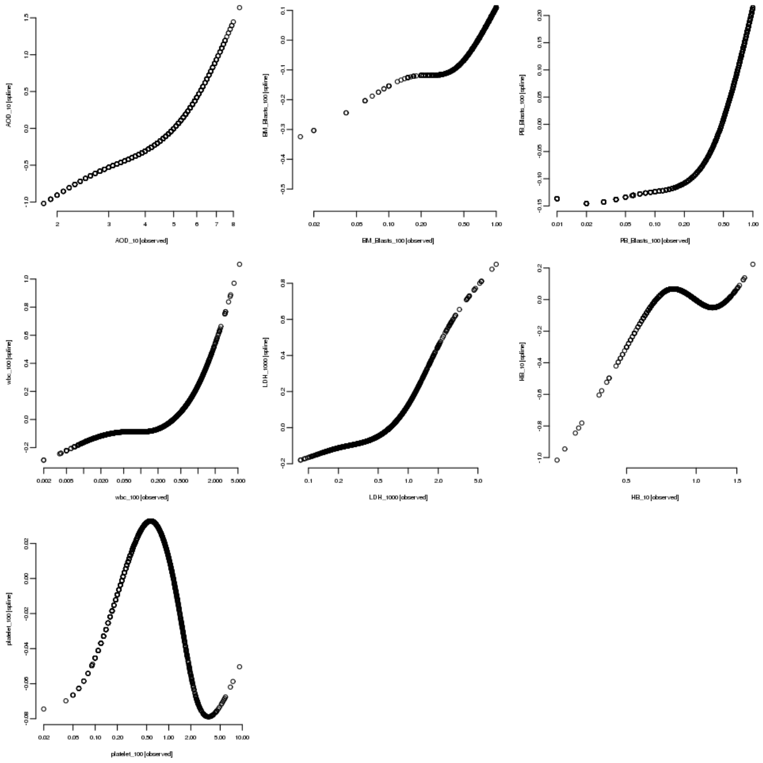

We rerun the R script in this Note twice: one in RStudio on a standard PC (Mac, R v.4.1.1) and one in RStudio Server Pro (Linux, R v.4.0.4)
Warnings, errors, and results with deviations in our rerunning on the PC are attached hereafter for comparison purposes. We note that in our two rerunning attempts, the same warnings and errors occur at the same locations, while slight deviations (fewer than those as compared to this Note) in the results between the two rerunning appear
We suspect the deviations (in numbers and figures) are due to coding errors and the difference between computing environments, and results will be again different if the R script is rerun in another environment

```
> summary(coxph(os ~ ., data=clinicalSpline, subset=trainI
dx))$concordance
         C       se(C)
0.65368150 0.01102915
> summary(coxph(os ~ ., data=dataFrame[groups %in% c("Clin
ical","Demographics")]), subset=trainIdx)$concordance
          C        se(C)
0.642972366 0.009139043
```

[outputs of the rerun (2021)]

Training set - accuracy

```
summary(coxph(os ~ ., data=clinicalSpline, subset=trainIdx))$concordance
```

```
## concordance.concordant             se.std(c-d)
##             0.65368150              0.01218587
```

```
summary(coxph(os ~ ., data=dataFrame[groups %in% c("Clinical","Demographics")]), subset=trainIdx)$concordance
```

```
## concordance.concordant             se.std(c-d)
##             0.64297237              0.01003199
```

Test set - accuracy

```
survConcordance(os[!trainIdx] ~ predict(coxph(os ~ ., data=clinicalSpline, subset=trainIdx), newdata = clinicalSp
line[!trainIdx,]))
```

```
## Call:
## survConcordance(formula = os[!trainIdx] ~ predict(coxph(os ~ 
##     ., data = clinicalSpline, subset = trainIdx), newdata = clinicalSpline[!trainIdx, 
##     ]))
## 
##   n= 507 
## Concordance= 0.6308385 se= 0.01772148
## concordant discordant  tied.risk  tied.time   std(c-d) 
##  63897.000  37392.000      0.000     34.000   3589.983
```

```
survConcordance(os[!trainIdx] ~ predict(coxph(os ~ ., data=dataFrame[groups %in% c("Clinical","Demographics")], s
ubset=trainIdx), newdata = dataFrame[!trainIdx,]))
```

```
## Call:
## survConcordance(formula = os[!trainIdx] ~ predict(coxph(os ~ 
##     ., data = dataFrame[groups %in% c("Clinical", "Demographics")], 
##     subset = trainIdx), newdata = dataFrame[!trainIdx, ]))
## 
##   n= 507 
## Concordance= 0.6296044 se= 0.01772148
## concordant discordant  tied.risk  tied.time   std(c-d) 
##  63772.000  37517.000      0.000     34.000   3589.983
```

No measurable improvement over (scaled) linear terms thus.

2.3.2 Random effects models

Here we fit the random effects model using our implementation in the `CoxHD` package. First for main effects only.

```
coxRFXFitOsTDMain <- CoxRFX(dataFrameOsTD[,mainIdxOsTD], osTD, groups[mainIdxOsTD])
```

Now including gene:gene interaction terms (min. recurrence = 8)   # Another command calling the incorrectly created variable ***osTDIdx***

```
whichRFXOsTDGG <- which((colSums(dataFrame)>=8 | mainIdxOsTD) & osTDIdx & groups %in% c(mainGroups,"GeneGene")) #
ie, > 0.5%
coxRFXFitOsTDGGc <- CoxRFX(dataFrameOsTD[,whichRFXOsTDGG], osTD, groups[whichRFXOsTDGG], which.mu=mainGroups) ##
allow only the main groups to have mean different from zero..
```

Compute Harrel's concordance index

```
survConcordance(osTD~coxRFXFitOsTDGGc$linear.predictors)
```

```
## Call:
## survConcordance(formula = osTD ~ coxRFXFitOsTDGGc$linear.predictors)
##
##   n= 1880
## Concordance= 0.7479676 se= 0.01003199
## concordant discordant  tied.risk  tied.time   std(c-d)
##  710819.00  239515.00       0.00     432.00   19067.49
```

```
> survConcordance(osTD~coxRFXFitOsTDGGc$linear.predictors)
$concordance
concordant
 0.7584512

$stats
concordant discordant  tied.risk  tied.time   std(c-d)
 720782.00  229552.00       0.00     432.00   19067.49

$n
[1] 1880

$std.err
  std(c-d)
0.01003199
```

[outputs of the rerun (2021)]

2.3.2.1 Variance components

Here we compute the variance components.

```
colGroups <- c(brewer.pal(12, "Paired")[c(10)],brewer.pal(12, "Paired")[c(6,4,3,5,12,9,1,2,7)],"#999999", brewer.
pal(12, "Paired")[c(8)])
colGroups <- colGroups[c(2:6,1,7:14)]
names(colGroups) <- levels(groups)[order(toupper(levels(groups)))]
PlotVarianceComponents(coxRFXFitOsTDGGc, col=colGroups)
title("Risk contributions OS (time-dep)")
```

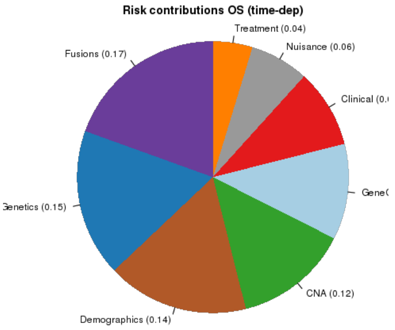

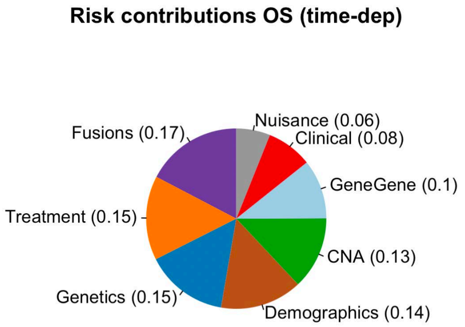

[outputs of the rerun (2021)]

2.3.3 Confidence intervals and significance tests

Estimate confidence intervals by parametric bootstrap and compare with Wald Test. Note that the usual sample with replacement yields inconsistencies for the interaction terms due to the overdispersed correlations. The theoretical description of the survival time simulation is given in section survival.

```
set.seed(42)
risk <- as.matrix(dataFrame[whichRFXOsTDGG]) %*% coxRFXFitOsTDGGc$coefficients
risk <- risk - mean(risk)
parBoot <- mclapply(1:100, function(i) {
            s <- SimSurvNonp(risk, os)
            c <- try(CoxRFX(dataFrame[whichRFXOsTDGG], s, groups=groups[whichRFXOsTDGG], sigma0=0.1, nu=0))
            if(class(c)=="try-error")
                return(s)
            c$Z <- NULL # set X to zero to save mem
            return(c)
        }, mc.cores=10)
```

Distributions of mean, sigma and df

```
boxplot(t(sapply(parBoot, `[[`, "sigma2")), border=colGroups[names(parBoot[[1]]$sigma2)], lty=1, pch=16, staplewe
x=0, ylab="sigma2", las=2, log="y", ylim=c(1e-3,1))
abline(h=0, lty=3)
points(coxRFXFitOsTDGGc$sigma2,  pch=19)
```

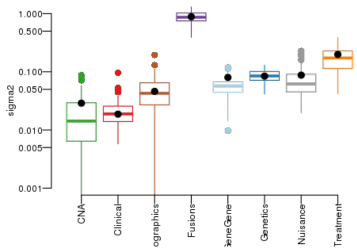

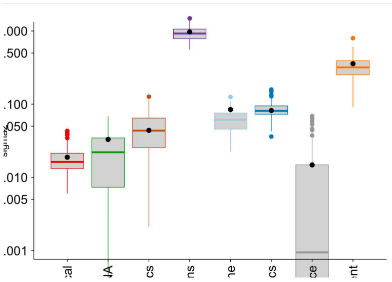

[outputs of the rerun (2021)]

```
boxplot(t(sapply(parBoot, `[[`, "mu")), border=colGroups[names(parBoot[[1]]$mu)], lty=1, pch=16, staplewex=0, yla
b="mu", las=2)
abline(h=0, lty=3)
points(coxRFXFitOsTDGGc$mu,  pch=19)
```

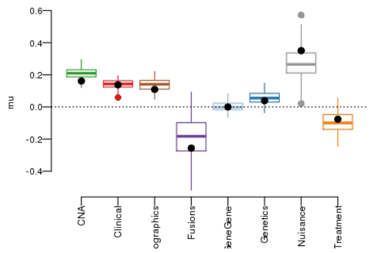

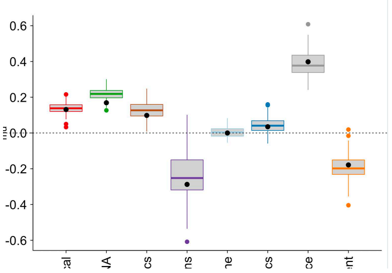

[outputs of the rerun (2021)]

```
boxplot(t(sapply(parBoot, `[[`, "df")), border=colGroups[names(parBoot[[1]]$mu)], lty=1, pch=16, staplewex=0, yla
b="df", las=2)
abline(h=0, lty=3)
points(coxRFXFitOsTDGGc$df,  pch=19)
```

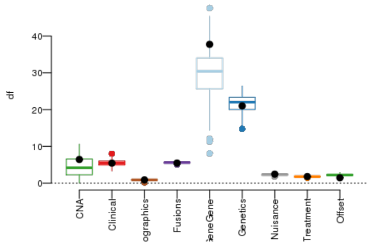

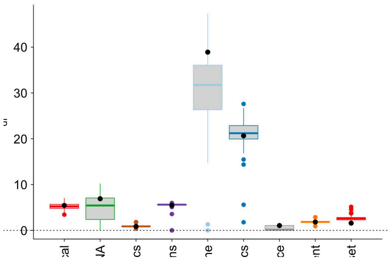

[outputs of the rerun (2021)]

Coefficients

```
v <- apply(sapply(parBoot, `[[`, "coefficients"), 1, var, na.rm=TRUE)
w <- diag(coxRFXFitOsTDGGc$var) ## H^{-1}
w2 <- diag(coxRFXFitOsTDGGc$var2) ## H^{-1} I H^{-1}
c <- coef(coxRFXFitOsTDGGc)
plot(c^2/v, c^2/w, log="xy", xlab="Chi2 (bootstrap)", ylab="Chi2 (analyt.)", cex=.66)
par(xpd=NA)
points(c^2/v, c^2/w2, pch=16, cex=.7)
arrows(c^2/v, c^2/w, c^2/v,c^2/w2, length=0.05)
abline(0,1)
abline(h=qchisq(c(0.95,0.99,0.999), 1, lower.tail=TRUE), lty=c(1,2,3))
```

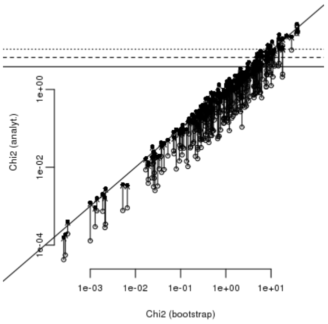

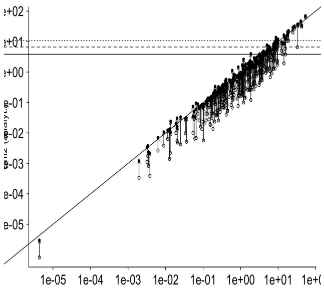

[outputs of the rerun (2021)]

The plot indicates a good agreement of the variance estimate `var2` , see section 2.1.4.. Knowing the distribution of the variance allows us to compute a Wald test of the coefficients. #### Supplementary Table S1 Table with significance

```
library(DT)
library(htmlwidgets)
pBoot <- pchisq(c^2/v,1, lower.tail=FALSE)
pVar2 <- pchisq(c^2/w2,1, lower.tail=FALSE)
pVar <- pchisq(c^2/w,1, lower.tail=FALSE)
waldOut <- data.frame(group = groups[whichRFXOsTDGG],
        `beta (log-hazard)`= c,
        `hazard exp(beta)` = exp(c),
        n = ifelse(groups[whichRFXOsTDGG] %in% c("CNA","Fusions","Genetics","GeneGene"), colSums(dataRaw[sub("_10
*$","",names(whichRFXOsTDGG))], na.rm=TRUE), NA),
        sd = sqrt(w2),
        `sd (bootstrap)` = sqrt(v),
        `sd (var)`= sqrt(w),
        `P-value`=pVar2,
        `Q (Benjamini-Yekutieli)` = p.adjust(pVar2, "BY"),
        `Q (Benjamini-Hochberg)` = p.adjust(pVar2, "BH"),
        check.names=FALSE
)
datatable(as.data.frame(lapply(waldOut, function(x) if(class(x)=="numeric") round(x,4) else x), check.names=FALSE
, row.names=row.names(waldOut)))
```

Show 10 **entries** # All tables generated from our rerun (see *Supplementary file 4*) deviate from this Note with only slight inconsistencies **Search:**

	group	beta..log.hazard.	hazard.exp.beta.	n	sd	sd..bootstrap.	sd..var.	P.value	Q..Benjamini.Yekutiel
ASXL1	Genetics	0.2398	1.271	70	0.1164	0.1184	0.1622	0.0395	
ATRX	Genetics	0.1181	1.1254	5	0.1067	0.1122	0.273	0.2682	
BCOR	Genetics	-0.0054	0.9946	35	0.1346	0.1439	0.1949	0.9681	
BRAF	Genetics	0.3249	1.3839	9	0.1247	0.1478	0.2603	0.0091	0.38
CBL	Genetics	-0.0858	0.9178	37	0.1367	0.1556	0.1953	0.5306	
CBLB	Genetics	0.0858	1.0896	1	0.0635	0.0786	0.2904	0.1765	
CDKN2A	Genetics	0.0269	1.0272	2	0.0878	0.0951	0.283	0.7595	
CREBBP	Genetics	0.0428	1.0437	10	0.1396	0.1365	0.244	0.7591	
CUX1	Genetics	0.1025	1.108	6	0.1317	0.1638	0.2496	0.4363	
DNMT3A	Genetics	0.0526	1.0541	357	0.0979	0.0901	0.1358	0.5908	

Showing 1 to 10 of 230 entries Previous 1 2 3 4 5 … 23 Next

```
library(xlsx)
wb <- createWorkbook("xlsx")
sheet  <- createSheet(wb, sheetName="Overall survival")
addDataFrame(waldOut,
        sheet,
        colnamesStyle = CellStyle(wb) + Font(wb, isBold=TRUE) + Border(),
        rownamesStyle = CellStyle(wb) + Font(wb, isBold=TRUE)
)
```

Volcano plot

```
par(mar=c(3,3,1,1)+.1,  bty="n", mgp=c(2,.5,0))
i <- coxRFXFitOsTDGGc$groups %in% c("Genetics", "CNA","Fusions","GeneGene","Treatment")#apply(coxRFXFitOsTDGGc$Z,
2,min) == 0 & apply(coxRFXFitOsTDGGc$Z,2,max) == 1
p <- pVar2 ## pvalues coxRFX
plot(c, 1/p, log='y', col=paste(colGroups[as.character(coxRFXFitOsTDGGc$groups)],"BB", sep=""), pch=ifelse(i,16,1
6), ylab="P-value",xlab="log hazard", cex=ifelse(i, sqrt(colMeans(coxRFXFitOsTDGGc$Z[!rev(duplicated(rev(tplSplit
Os))),])*50),1), xlim=range(c*1.2))
```

```
## Warning in sqrt(colMeans(coxRFXFitOsTDGGc$Z[!rev(duplicated(rev(tplSplitOs))), : NaNs produced
```

```
#abline(h=qchisq(c(0.95,0.99,0.999), 1, lower.tail=TRUE), lty=c(1,2,3))
w <- which(p.adjust(p,"BY") < 0.1)
points(c[w], 1/p[w],  pch=1, cex=ifelse(i[w], sqrt(colMeans(coxRFXFitOsTDGGc$Z[!rev(duplicated(rev(tplSplitOs))),
w])*50),1))
w <- which(p.adjust(p,"bonf") < 0.05)
par(xpd=NA)
text(c[w], 1/p[w], names(c[w]), pos=3)
u <- par("usr")
f <- c(0.01,0.05,0.1,0.2,0.5)
s <- sqrt(f*50)
legend("topright",legend=f, pch=16, pt.cex=s, bty='n', col=paste("#88888888"))
par(xpd=FALSE)
abline(h=1/0.05, lty=2)
abline(h=1/max(p[which(p.adjust(p,"BY") < 0.1)]), lty=3)
```

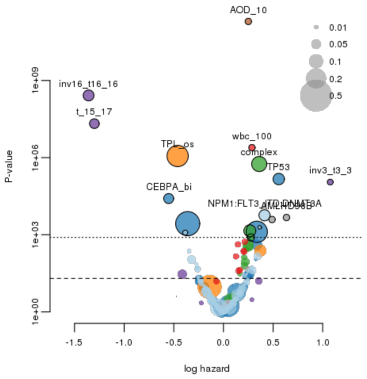

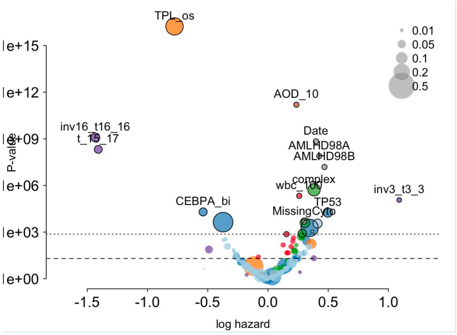

[outputs of the rerun (2021)]

P-values and random model

```
set.seed(42)
Z <- apply(coxRFXFitOsTDGGc$Z, 2,sample)[1:nrow(dataFrame),] ## random covariates
coxRFXFitOsRain <- CoxRFX(Z, os, groups=coxRFXFitOsTDGGc$groups, nu=1) ## model
w2 <- diag(coxRFXFitOsRain$var2)
c <- coef(coxRFXFitOsRain)
p2 <- pVar2
plot(seq(0,1,l=length(p2)+1)[-1],sort(p2), xlab="P-value (expected)", ylab="P-value (observed)", pch=16, col="gre
y")
abline(0,1)
points(seq(0,1,l=length(p)+1)[-1],sort(p), pch=16)
legend("topleft",bty="n", c("observed","randomised"), pch=16, col=c("black","grey"))
```

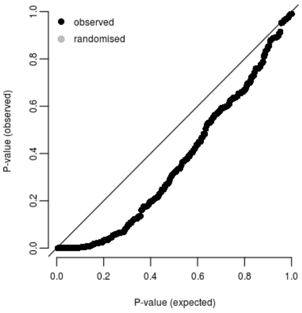

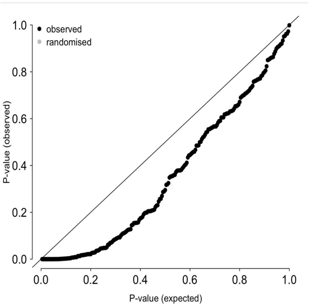

[outputs of the rerun (2021)]

Distribution of the variance components

```
v <- t(sapply(parBoot, function(x) {t <- try(VarianceComponents(x, newZ=dataFrame[whichRFXOsTDGG])); if(class(t)=
="try-error") rep(NA, nlevels(x$groups)+1) else t}))
boxplot(v, border=colGroups[colnames(v)], lty=1, pch=16, staplewex=0, ylab="variance comp.", las=2)
abline(h=0, lty=3)
points(VarianceComponents(coxRFXFitOsTDGGc),  pch=19)
```

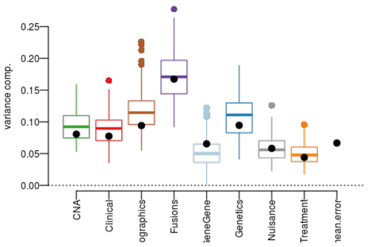

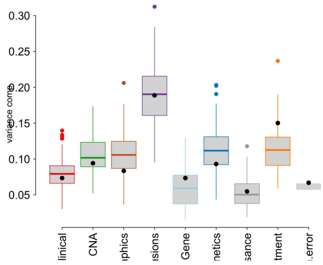

[outputs of the rerun (2021)]

```
rm(parBoot)
```

2.3.4 Risk constellation plots

Plot of log hazard v outcome

```
par(mar=c(3,3,3,1), mgp=c(2,.5,0))
t <- os
s <- survfit(os~1)
q <- quantile(t[,1], seq(0,1,.1))# q <- splinefun( s$surv, s$time,"monoH.FC")(seq(1,min(s$surv),l=10))
c <- cut(t[,1], q, na.rm=TRUE)
h <- coxRFXFitOsTDGGc$linear.predictors[rev(!duplicated(rev(tplSplitOs)))][order(tplSplitOs[rev(!duplicated(rev(t
plSplitOs)))])]
o <- order(h)
plot(h[o], col= (brewer.pal(10,'RdBu'))[c[o]], type='h', xaxt="n", xlab='Patient', las=2, ylab="log hazard")
u <- par("usr")
q <- pmin(q,365*12)
image(x=q/max(q)*500, y=c(u[4]-(u[4]-u[3])/20, u[4]), matrix(1:10), col= (brewer.pal(10,'RdBu')), add=TRUE)
#axis(side=3, at=seq(1,500,l=11), labels=seq(0,1,.1))
axis(side=3, at=pretty(q/365)/max(q)*365*500, labels=pretty(q/365))
lines(ksmooth(seq_along(o),t[o,2]==0, bandwidth=50))
```

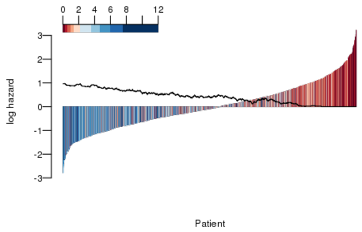

2.3.4.1 Figure S1A

Risk constellation plots using the `stars()` function

```
set.seed(42)
library(HilbertVis)
nStars <- 32
s <- sample(nrow(dataFrame),nStars^2) #1:(nStars^2)
l <- "coxRFXFitOsTDGGc"
t <- os#get(l)$surv
p <- PartialRisk(get(l),  newZ=dataFrame[, colnames(get(l)$Z)])
p <- p[,colnames(p)!="Nuisance"]
locations <- 1.5*hilbertCurve(log2(nStars)) #2*expand.grid(1:nStars,1:nStars)
h <- hclust(dist(p[s,]))
x <- p - rep(colMeans(p), each=nrow(p))
x <- x/(2*sd(x)) + 1
c <- cut(t[s,1][h$order], quantile(t[,1], seq(0,1,0.1), na.rm=TRUE))
if(l=="coxRFXFitOsTDGGc")
    x <- x[,c("Demographics","Treatment","Fusions","CNA","Genetics","GeneGene","Clinical")]
mg14:::stars(x[s,][h$order,]/2, scale=FALSE, locations=locations, key.loc=c(0,-3), col.lines=ifelse(t[s,2][h$orde
r],1,NA), col.stars = (brewer.pal(11,'RdBu'))[c], density=ifelse(t[s,2][h$order],NA,NA))
symbols(locations[,1], locations[,2], circles=rep(.5,(nStars^2)), inches=FALSE, fg="grey", add=TRUE, lty=1)
title(main=l)
```

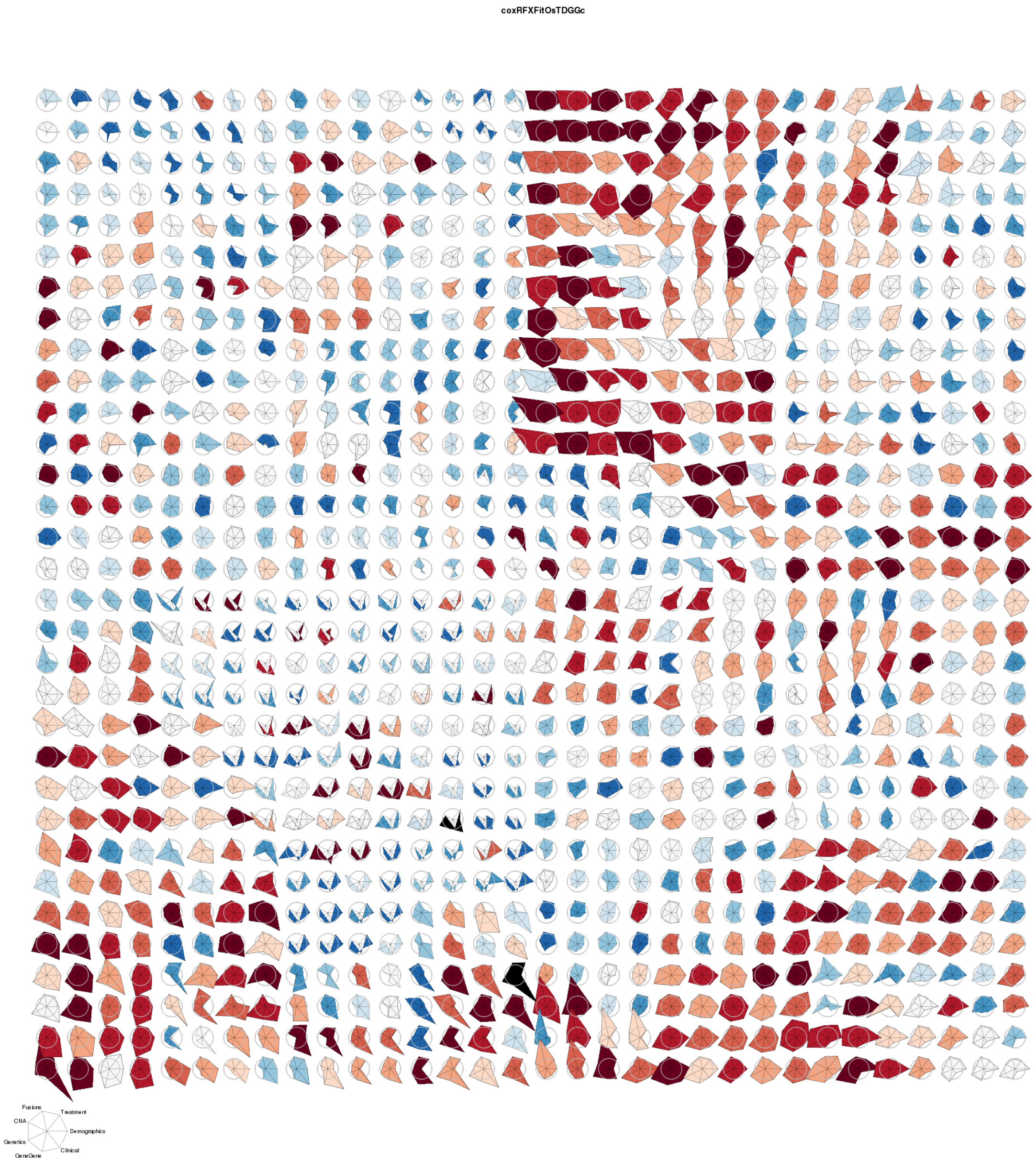

2.3.4.2 Figure S1B

Risk constellation examples

```
patients <- match(c("PD11104a", "PD8314a","PD10941a","PD10828a","PD10844a","PD10829a","PD10996a","PD10840a"), row
names(dataFrame))
genotype <- apply(dataFrame[groups %in% c("Fusions","CNA","Genetics")]==1, 1,function(x) paste(names(which(x)), c
ollapse=";"))

t <- os
p <- PartialRisk(coxRFXFitOsTDGGc, newZ=dataFrame[, whichRFXOsTDGG])
p <- p[,colnames(p)!="Nuisance"]
x <- p - rep(colMeans(p), each=nrow(p))
x <- x/(2*sd(x)) + 1
c <- cut(t[patients,1], quantile(t[,1], seq(0,1,0.1), na.rm=TRUE))
x <- x[patients,c("Demographics","Treatment","Fusions","CNA","Genetics","GeneGene","Clinical")]
locations <- expand.grid(seq_along(patients)* 1.5, 1)
mg14:::stars(x/2, scale=FALSE, locations=locations, key.loc=NA, col.lines=ifelse(t[patients,2],1,NA), col.stars =
(brewer.pal(11,'RdBu'))[c])
symbols(locations[,1], locations[,2], circles=rep(.5,length(patients)), inches=FALSE, fg="grey", add=TRUE, lty=1)
text(locations[,1], locations[,2]-1,labels=clinicalData$PDID[patients], pos=1)
l <- apply(dataFrame[patients,c("gender","AOD_10","TPL_os","wbc_100")], 1,paste, collapse=";")
par(xpd=NA)
text(locations[,1], locations[,2]+1,labels=paste(gsub(";","\n",genotype[patients]),l, paste(round(os[patients,1],
2), osYr[patients,2]), sep="\n"), pos=3)
```

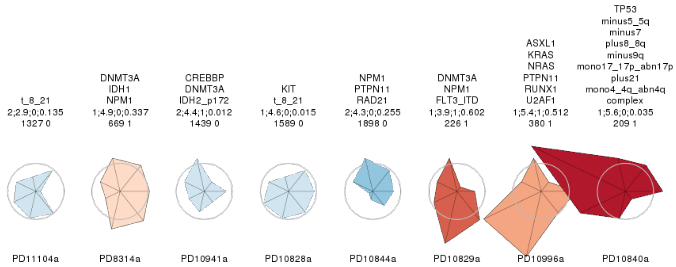

3 Multistage modelling

3.1 Definitions

3.1.1 Nomenclature

We use the following nomenclature: $f(T = t) = f(t)$ denotes a probability density, $F(T = t) = P(T < t) = F(t)$ the corresponding cumulative distribution function. $S(T = t) = 1 - F(t)$ is the survivor function, the name being motivated by the situation that t is a death time. In cases where it is clear to which variable a (cumulative) density refers to, we may drop the stochastic variable and simply use its value as the argument, $f(t) = f(T = t)$. We use the convention of lower case variables t to denote the values of the corresponding upper case stochastic variable $T = t$, $U = u$ and so on. For a categorical stochastic process X_t, $t \in \mathbb{R}^+$ we use the symbol $P(X_t)$ to denote the probability distribution at time t. The symbol Z denotes the covariates.

3.1.2 Transitions

We use a hierarchical multistage model to quantify the rates at which a patient progresses from one disease/treatment stage to another (Figure 2A). After learning the marginal time-dependent transition probabilities for each event, we can combine these into a time-dependent joint probability.

In particular, we model the following transition times:

- Time of complete remission (complete remission CR, T_{CR})
- Time of non-remission death (Non-complete remission death NCD, T_{NCD})
- Time from CR to relapse (Relapse R, T_R)
- Time from CR to non-relapse death (Non-relapse death NRD, T_{NRD})
- Time from relapse to post-relapse death (Post-relapse mortality PRD, T_{PRD})

CR and CIR and midpoints, allowing for further events, NCD, NCD and PRD are endpoints. Due to the hierarchical nature of the model, only one endpoint can ever occur and midpoints are transient.

3.1.3 States

The probability to be in a given state is then given by the combination of event times, such that

- the transition to a given stage has happened before the other competing transitions
- no subsequent transition has occurred yet
- no other endpoint has been reached yet

To be alive in CR at time t, for example, requires that CR occurred before t, CR was achieved before NCD, and neither relapse nor NCD have occurred yet. Overall, a patient can only be in one of the following six states at time t, each corresponding to a particular ordering of event times:

Table 2. Stages

Stage	Abbreviation	Ordering of times	Symbol
Alive in induction	AI	$t < T_{CR}, T_{NCD}$	$\mathcal{I}_{AI}(t)$
Death without complete remission	NCM	$T_{NCD} < t; T_{NCD} < T_{CR}$	$\mathcal{I}_{NCM}(t)$
Alive in complete remission	ACR	$T_{CR} < T_{NCD}; t < T_R, T_{NCD}$	$\mathcal{I}_{ACR}(t)$
Death without relapse	NRM	$T_{CR} < T_{NCD}; T_{NRD} < t < T_R$	$\mathcal{I}_{NRM}(t)$
Alive after relapse	AR	$T_{CR} < T_{NCD}; T_R < T_{NRD}; t < T_{PRD}$	$\mathcal{I}_{AAR}(t)$
Death after relapse	PRM	$T_{CR} < T_{NCD}; T_R < T_{NRD}\ ; T_{PRD} < t$	$\mathcal{I}_{PRM}(t)$

This defines a stochastic process X_t on the given set of six states, $X_t \in \{AI, ACR, AR, NCM, NRM, PRM\}$. Initially, all patients will be alive in induction, $X_0 = AI$.

3.1.4 Factorisation of the joint probability

The hierarchical nature of the model implies that the joint probability of event time factorises

$$f(T_{CR}, T_{NCD}, T_R, T_{NRD}, T_{PRD}) = f(T_{CR}) \times f(T_{NCD}) \times f(T_R \mid T_{CR}) \times f(T_{NRD} \mid T_{CR}) \times f(T_{PRD} \mid T_R). \quad (19)$$

The above factorisation lays out a strategy in which each of the 5 factors may be estimated separately. The probability of each state $P(X_t)$, defined in section states, are then computed by integrating the joint density f, Eq.(19) over the simplexes $\mathcal{I}_{\cdot}(t)$ defining a particular ordering of transitions detailed in table 2:

$$P(X_t = x) = \iiiint\!\!\!\int_{\mathcal{I}_x(t)} f(t_{CR}, t_{NCD}, t_R, t_{NRD}, t_{PRD})\, dt_{CR}\, dt_{NCD}\, dt_R\, dt_{NRD}\, dt_{PRD}. \tag{20}$$

The integral can be successively evaluated as described below.

3.2 Static multistage models

To estimate the population average transition probabilities and absolute incidence of each each individual stage we use the `msSurv` R package (Ferguson, Datta, and Brock 2012). The resulting time-dependent joint distribution $P(X_t)$ is shown in Figure 2B.

3.3 Multistage random effects modelling

To estimate how each transition T depends on the set of variables Z introduced in section variables (transitions), we use a random effects model for each transition to obtain $f(T \mid Z)$. Competing events are considered to be censored. We apply a separate random effects model to estimate all five terms in Eq.(19).

3.3.1 Unconditional densities

The estimation of the unconditional densities $f(T = t \mid Z)$ is straightforward. The random effects model yield the marginal survivor function $S(t \mid Z) = S_0(t)^{\exp(uZ)}$, quantifying the hypothetical scenario that there were no competing events T'. From $S(t \mid Z)$ we can derive the marginal densities $f(t \mid Z) = -dS(t)/dt = -\exp(uZ)S_0(t)^{\exp(uZ)-1}dS_0(t)/dt$ for each transition T given the covariates Z. The Kaplan-Meier estimate of $S_0(t)$ is a step function, so we may compute $f(t \mid Z)$ via a numerical differentiation.

3.3.2 Conditional densities

To estimate the conditional densities of the type $f(U = u \mid Z, T)$, we use the following approach:

$$S(u \mid Z, T = t) = S_0(u - t \mid Z)^{\exp(g(t))} = S_0(u - t)^{\exp(uZ + g(t))}. \tag{21}$$

This allows us to estimate the incidence of each event from the beginning of each stage $S_0(u - t \mid Z)$ and express the time-dependence as a smooth function $g(t)$. For example, the duration of CR1 is a prognostic factor for post-relapse mortality, e.g. (Burnett et al. 2013).

The above corresponds to a Cox proportional hazards model with a time-dependent smooth covariate g(t).

Here we estimate $g(t)$ with a spline term with 10 degrees of freedom. We estimate $S_0(u - t)$ and u using a random effects model and subsequently estimate $g(t)$ using uZ as an intercept:

```
fit_u_minus_t_given_Z <- CoxRFX(Z, Surv(U-T, event))
beta <- coef(fit_u_minus_t_given_Z)
fit_u_given_Z_t <- coxph(Surv(U-T, event) ~ I(beta %*% Z) + spline(T, df=10))
```

We may thus obtain $S(u \mid T = t, Z) = \left(S_0(u - t)^{\exp(uZ)}\right)^{\exp(g(t))}$ by offsetting the baseline hazard $S_0(u - t)$ and exponentiating for the effect of covariates uZ and exponentiating for the effect of time-dependence.

The absolute probability to be in state U is given by integrating over the conditional probabilities $S(u \mid T = t, Z)$, weighted by the probability of the preceding event probabilities $f(T = t \mid Z)$:

$$P(U < u \mid Z) = \int_0^t f(T = t \mid Z) \int_t^u f(U = v \mid T = t, Z)dtdv = \int_0^u f(T = t \mid Z)F(u \mid T = t, Z)dt \tag{22}$$

With $F(uT=t, Z) = 1-S(uT=t, Z) $, we can use the above definition to numerically solve the above integral.

The pseudo code for this is given by:

```
S0_given_Z <- S0 ^ exp(beta %*% Z)
gt <- predict(fit_u_given_t, data=data.frame(T=1:length(u)))
ft <- -diff(St)
for(t in 1:length(u)){
   Fu_given_Zt <- 1-S0_given_Z[-(1:t)] ^ exp(gt)
   Pu <- cumsum(ft * Fu_given_Zt)
}
```

3.3.3 Competing risk adjustment

In cases of competing events (CR and NCD; NCD and CIR), we use a competing risk adjustment between two event times T, U, to obtain

$$S(T = t \mid Z, T < U) = \int_t^v \int_v^\infty f(T = t' \mid Z)f(U = u' \mid Z)dt'du' = \int_0^t f(T = t' \mid Z)S(U = t' \mid Z)dv.$$ [Correction] $\mathbf{1} - \int_0^t \boldsymbol{f(T = t'|Z)S(U = t'|Z)dt'}$

In practical terms, $S(t \mid Z) = S_0(t)^{\exp(uZ)}$ denotes the survivor function estimated by the Kaplan-Meyer estimate $S_0(t)$, exponentiated by the hazard $\exp(uZ)$. The differential $f(t \mid Z)$ is obtained by evaluating the difference of $S(t + 1 \mid Z) - S(t \mid Z)$ at intervals of length 1 day, pseudo code

`S_t_cr <- cumsum(diff(S_t) * S_u)`   [Correction] S_t_cr <- cumsum(c(1, diff(S_t)) * S_u)

3.3.4 Encoding of events

Error: diff() returns negative values and N_{diff}=n−1, hence we add 1 to diff(S_t)

Table 3.

Endpoint	Censored	Model	Competing	Interval	Time-dependency
Complete remission	Non-remission death	CoxRFX	Non-remission death	From ER	
Non-remission death	Complete remission	CoxRFX	Complete remission	From ER	
Relapse	Non-relapse death	CoxRFX	Non-relapse death	From CR1	Time to CR1
Non-relapse death	Relapse	CoxRFX	Relapse	From CR1	Time to CR1
Post-relapse death	Last follow up	CoxRFX	-	From relapse	Duration of CR1

3.3.5 Probabilities of each state

As the density (19) factorises we can successively evaluate each term, beginning with the first transition. The probability to be in a given state is then computed according to the rules outlined in the previous two subsections.

3.3.5.1 Death without complete remission

The probability is given by a simple competing risk adjustment between T_{CR} and T_{NCD}:

$$P(X_t = NCM) \mid Z) = P(T_{NCD} < t, T_{NCD} < T_{CR} \mid Z) = 1 - \int_0^t f(T_{NCD} = u \mid Z) F(T_{CR} = u \mid Z) du. \tag{24}$$

3.3.5.2 Complete remission

We first compute the probability that CR is achieved, irrespective of the subsequent events, using a competing risk adjustment with T_{NCD}:

$$P(X_t = CR \mid Z) = P(T_{CR} < T_{NCD}, T_{CR} < t \mid Z) = 1 - \int_0^t f(T_{CR} = u \mid Z) F(T_{NCD} = u \mid Z) du, \tag{25}$$

where $CR = \{ACR \cup NRD \cup AAR \cup PRD\}$, which can then be further subdivided according to the possible subsequent events. To be alive, neither relapse nor non-relapse death may have occurred.

3.3.5.3 Alive in induction

The probability to be alive in induction is given by

$$P(X_t = AAI \mid Z) = 1 - P(X_t = CR \mid Z) - P(X_t = NRD \mid Z). \tag{26}$$

3.3.5.4 Non-relapse deaths

The probability of non-relapse deaths $P(X_t = NRM \mid Z)$ is computed in the following way. We first estimate transition rates for non-relapse deaths and relapses, $f(T_{NRM} \mid Z, T_{CR})$
and $f(T_{NRM} \mid Z, T_{CR})$, as outlined in conditional-densities. Instead of the marginal density $f(T_{CR} \mid Z)$ we use the differential of $dP(X_t = CR \mid Z)/dt$ in Eq.(22).

As only one of the two events can ever occur we then use a competing risk adjustment to obtain the absolute probability probability of $P(X_t = NRD \mid Z)$ and $P(X_t = R \mid Z)$, respectively, where $R = \{AAR \cup PRM\}$ denotes a relapse.

3.3.5.5 Alive in complete remission

The probability to be alive in first complete remission equals the probability of neither dying nor relapsing:

$$P(X_t = ACR) = 1 - P(X_t = NRD) - P(X_t = R). \tag{27}$$

3.3.5.6 Post-relapse death

The probability of post-relapse deaths $P(X_t = PRM \mid Z)$ is computed as described in conditional-densities. We first estimate the rate for post-relapse deaths and relapses, $f(T_{PRM} \mid Z, T_R)$, with the derivative of $dP(X_t = R \mid Z)/dt$ in Eq.(22).

3.3.5.7 Alive after relapse

Finally, the probability to be alive after relapse is given by

$$P(X_t = AAR \mid Z) = P(R \mid Z) - P(PRM \mid Z). \tag{28}$$

3.3.6 Comments

In the absence of an established estimator of the joint density Eq.(19), we assumed that each factor of the density may be separately estimated using a random effects model. We note that the interdependence of observed events could in general introduce a bias as the censoring is not independent. The precise magnitude of this effect still needs to be investigated.

We observed, however, a good consistency of the average predictions and static multistage probabilities, indicating that those biases, on average, tend to cancel. Moreover cross-validation of our methodology ascertained a very good predictive performance despite all potential shortcomings.

3.4 Confidence intervals

3.4.1 Marginal probabilities

For each predicted variable we can derive 95% confidence intervals from the prediction error of the log hazard, $(h_{0.025}, h_{0.975}) \approx h + (-2, 2) \times \hat{V}[h \mid Z]$, with $V[h \mid Z]$. This translates to the survival function as follows using the log-log approach:

$$S_{0.025}(t \mid Z) = S_0(t)^{\exp(h_{0.025})} \tag{29}$$

$$S_{0.975}(t \mid Z) = S_0(t)^{\exp(h_{0.975})}. \tag{30}$$

Note that this does not model the error of the baseline survival estimate $S_0(t)$.

3.4.2 Survival after remission

Let the symbol PCS denote post remission survival. In the following sections all quantities are conditional on the data Z.

3.4.2.1 Analytical confidence intervals

Analytical confidence intervals can be calculated using a the propagation of errors based on a Taylor expansion of the PCS probability:

$$V[h_{PCS}] \approx \sum_i \left(\frac{\partial h_{PCS}}{\partial h_i}\right)^2 V[h_i] \tag{31}$$

$$h_{PCS} = \log\log P_{PCS} + \log\log P_0(t) \tag{32}$$

$$\frac{\partial h_{PCS}}{\partial h_i} = \frac{\partial \log\log P_{PCS}}{\partial h_i} \tag{33}$$

$$= \frac{1}{P_{PCS}\log(P_{PCS})}\frac{\partial P_{PCS}}{\partial h_i} \tag{34}$$

To facilitate an efficient computation of the derivatives of P_{PCS}, which is given by the integrals above, we use the pointwise approximation:

$$P_{PCS} \approx S_{NRD}(1-(1-S_R)(1-S_{PRD})) = S_{PCS} \tag{35}$$

where $S_.$ denote the Kaplan-Meyer estimates of the survival probabilities.

The partial derivative of the loglog is given by

$$\frac{\partial \log\log S}{\partial x} = \frac{1}{\log(S)}\frac{1}{S}\frac{\partial S}{\partial x}. \tag{36}$$

So the variance of the loglog overall survival reads:

$$V[h_{PCS}] \approx \frac{1}{(S_{PCS}\log S_{PCS})^2}\left(V[S_{NRD}](1-(1-S_R)(1-S_{PRD}))^2 + V[S_R]S_{NRD}^2(1-S_{PRD})^2 + V[S_{PRD}]S_{NRD}^2(1-S_R)^2\right) \tag{37}$$

with

$$V[S_i] = \left(\frac{\partial S_0(t)^{\exp(h)}}{\partial h}\right)^2 V[h] = (S_i \log S_i)^2 V[h]. \tag{38}$$

This allows to define 95% confidence intervals of h_{PCS} as:

$$h_{PCS}^{0.975} = h_{PCS} + 2V[h_{PCS}]. \tag{39}$$

This translates to an overall survival:

$$P_{PCS}^{0.975} = \exp\exp(h_{PCS}^{0.975}) = \exp(\exp(h_{PCS})\exp(2V[h_{PCS}])) = P_{PCS}^{\exp(2V[h_{PCS}])} \tag{40}$$

Note that in the last step uses the competing risk and time-adjusted estimate P_{PCS} again

3.4.2.2 Simulated

A more accurate account comes from simulations of errors in the predicted log hazard. The cumulative survival functions are given by

$$S_.(t) = S_{0.}(t)^{\exp(h_.+\epsilon_.)} \tag{41}$$

So drawing

$$\epsilon_. \sim N(0, \hat{V}[h_.]) \tag{42}$$

for each event type and repeating the computations outlined in [combined-os] yields an empirical distribution of the survival distribution of $S_{PCS}(t)$.

We use `i=200` simulations to compute the empirical confidence intervals.

Note: In all cases the prediction errors are assumed to be independent.

3.4.3 Differential survival

Confidence intervals for differential survival, e.g. with and without allograft are computed as in the previous section. A complication arises as the errors are correlated. We hence sample errors for all common variables and then sample those variable that differ. This approach allows to assess the uncertainty resulting from a subset of variables, and on the background of the joint variation in the set of common features.

3.4.4 Overall survival from diagnosis

We use a numerical approach similar to the one outlined above to compute the confidence intervals for overall survival measured from diagnosis. Note that it is in principle also possible to derive analytical confidence intervals analogous to section 3.4.2.1.

3.5 Measures of absolute prediction errors

As mentioned above, one of the key advantages of the multistage model is that it calculates absolute probabilities at a particular time rather than relative risks (hazards). While the canonical measure for assessing the predictive performance for survival models is the concordance C, which measures the agreement of the hazard and the survival times analogous to Kendall's τ, it therefore is useful to also define measures of absolute prediction errors.

The basic idea is to quantify the difference between a prediction at time t, $P(X_t = 1|Z_i)$ and the observed status ($I(t < t_i) \in 0, 1$ here fore simplicity) at this time for each patient i. In a perfect model $P(X_t = 1|Z_i)$ would change from 0 to 1 at exactly the time of the event t_i for patient i. In reality, of course $P(X_t = 1|Z_i)$ will be a probability in the interval $[0, 1]$ and we would expect that this probability accurately describes the distribution across patients.

We can define different measures $D(P(X_t = 1|Z_i), I(t < t_i))$ for the agreement of probabilities and outcome. Choices are absolute error $D_1(x, y) = |x - y|$, squared error D_2(x,y)=$(x - y)^2$ (equivalent to the Brier score), Bayes error $D_B(x, y) = I((x > 0.5) = y)$ and entropy $D_E(x, y) = y \log_2(y/x) + (1 - y) \log_2((1 - y)/(1 - x))$. A measure for the overall absolute prediction accuracy is then the average

$$A_t = 1/n \sum_i D(P(X_t = 1|Z_i), I(t < t_i)). \tag{43}$$

One challenge arises due to censoring of some observations. In this case we extrapolate the status from the state at which a patient was last seen using the population survival distribution $S(t) = 1 - P(X_t = 1)$, $\hat{I}_t = S(t)/S(t_i)$ for $t > t_i$ and 1 otherwise. Here we choose the Kaplan-Meier estimator for $S(t)$.

The above absolute prediction error measure are implemented in `CoxHD::ape()` .

The squared error D_2 can be used to define a measure analogous to the fraction of explained variance,

$$R^2 = 1 - \frac{\sum_i D_2(P(X_t = 1|Z_i), I(t < t_i))}{D_2(P(X_t = 1), I(t < t_i))}, \tag{44}$$

where $P(X_t = 1) = 1 - S(t)$ in the denominator is given by the Kaplan-Meier estimator and hence independent of the covariates Z and identical for each patient i. The measure R^2 quantifies how much the squared error is reduced compared to a constant survival prediction.

3.6 Code

3.6.1 Static multistage model

3.6.1.1 Figure 2B

Multi-state using msSurv (Ferguson, Datta, and Brock 2012).

```
library(msSurv)
d <- sapply(1:nrow(clinicalData), function(i){
            i <<- i
            t <- c(as.numeric(clinicalData[i,c("CR_date","Recurrence_date","Date_LF")]) - as.numeric(clinicalData
$ERDate[i]))
            o <- order(t, na.last=NA)
            stages <- c(1:3,0)
            r <- stages[c(1, o+1)]
            if(clinicalData$Status[i])
                r[length(r)] <- r[length(r)-1] +3
            tt <- c(0,t[o])
            if(length(o)==0)
                return(c(rep(NA,7),i))
            s <- cbind(id=i, stop=tt[-1], start.stage=r[-length(r)], end.stage=r[-1])[diff(tt)!=0,]
            #s <- cbind(time1 = tt[-length(tt)], time2=tt[-1], death=c(rep(0, length(o)-1), clinicalData$Status[i
]), outer(0:(length(o)-1), r[-3], `>=`)+0, i=i)[diff(tt)!=0,]
            return(s)
        })
d <- as.data.frame(do.call("rbind",d))
nodes <- as.character(1:6)
edges <- list(`1`=list(edges=c("2","4")), `2`=list(edges=c("3","5")), `3`=list(edges="6"), `4`=list(edges=NULL),
`5`=list(edges=NULL),`6`=list(edges=NULL))
struct <-  new("graphNEL", nodes = nodes, edgeL = edges, edgemode = "directed")
msurv <- msSurv(d, struct, bs = FALSE)
```

```
##
## Entry distributions calculated for states 2 3 4 5 6 .
##
## Exit distributions calculated for states 1 2 3 .
```

```
y <- t(apply(cbind(1,-msurv@ps[,c(4:6, 3:1)]),1,cumsum))
par(mar=c(3,3,1,1), bty="n", mgp=c(2,.5,0), las=1)
plot(msurv@et/365.25, y[,1], ylim=c(0,1), type="s",lty=0, xlab="Time after diagnosis", ylab="Fraction of patients
", xlim=c(0,10), xaxs="i", yaxs="i")
steps <- function(x, type="s") rep(x, each=2)[if(type=="s") -1 else -2*length(x)]
x <- steps(msurv@et/365.25, type="S")
for(i in 1:6)
    polygon(c(x, rev(x)), c(steps(y[,i]), rev(steps(y[,i+1])) ), col=c(brewer.pal(5,"Pastel1")[c(1:3,5,4)],"#DDDD
DD")[i], border=NA)
abline(h=seq(0,1,.2), col='white', lty=3)
abline(v=seq(0,10,1), col='white', lty=3)
lines(x, steps(y[,4]), lwd=2)
w <- which.min(abs(msurv@et/365.25-10))
text(x=par("usr")[2], y= y[w,-7]+diff(y[w,])/2, labels=c("early death","death in CR","death after relapse","alive
with relapse","alive in remission","induction/LOF"), pos=2)
```

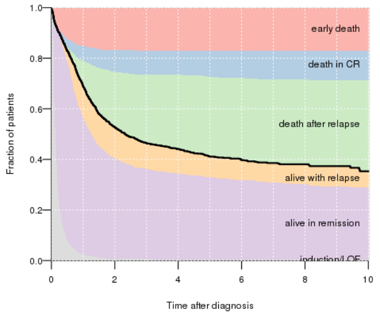

3.6.2 Prepare covariates

Times for allografts pre and post relapse, after 1CR only

```
alloIdx <- clinicalData$TPL_type %in% c("ALLO","FREMD") # only allografts
alloTimeCR1 <- clinicalData$Time_1CR_TPL + .5 # +.5 to make > 0
alloTimeCR1[!alloIdx | (clinicalData$TPL_date < clinicalData$Recurrence_date & !clinicalData$TPL_Phase %in% c("CR
1","RD"))] <- NA
```

Error: There is no *"RD"* but *"RD1"* in *clinicalData$TPL_Phase*

```
> table(clinicalData$TPL_Phase)

 CR1  CR2  CR3  CR4  PR1  RD1 Rez1 Rez2
 432   91    4    1   23  137  129    8
```

Create data frames for each phase

```
whichRFXRel <- whichRFXOsTDGG[grep("TPL",names(whichRFXOsTDGG), invert=TRUE)] #mainIdx & !grepl("TPL", names(data
Frame)) & groups!="Nuisance"
```

whichRFXRel denotes the same as *whichRFXOsGG*, which has been created earlier; the comment is incorrect, as Nuisance variables were in fact included in *whichRFXRel*

```
> table(names(dataFrame[whichRFXRel])%in%names(groups[groups=="Nuisance"]))

FALSE  TRUE
  225     4
```

```
t <- clinicalData$Recurrence_date
t[is.na(t)] <- as.Date(1e6, origin="2000-01-01")
relData <- MakeTimeDependent(dataFrame[whichRFXRel], timeEvent=alloTimeCR1, timeStop=as.numeric(pmin(t, clinicalD
ata$Date_LF) - clinicalData$CR_date), status=!is.na(clinicalData$Recurrence_date)+0)
relData$transplantCR1 <- relData$event
relData$event <- NULL
relData$transplantRel <- 0
nrdData <- MakeTimeDependent(dataFrame[whichRFXRel], timeEvent=alloTimeCR1, timeStop=as.numeric(pmin(t, clinicalD
ata$Date_LF) - clinicalData$CR_date), status=is.na(clinicalData$Recurrence_date) & clinicalData$Status)
nrdData$transplantCR1 <- nrdData$event
nrdData$event <- NULL
nrdData$transplantRel <- 0
alloTimeRel <- clinicalData$TPL_date - clinicalData$Recurrence_date + .5 # +.5 to make > 0
alloTimeRel[!alloIdx | (clinicalData$TPL_date < clinicalData$Recurrence_date & !clinicalData$TPL_Phase %in% c("CR
1","RD"))] <- NA
i <- !is.na(clinicalData$Recurrence_date)
prdData <- MakeTimeDependent(dataFrame[i,whichRFXRel], timeEvent=alloTimeRel[i], timeStop=as.numeric(clinicalData
$Date_LF- clinicalData$Recurrence_date)[i], status=clinicalData$Status[i])
prdData$transplantCR1 <- rep(0,nrow(prdData))
w <- sub("\\.1","",rownames(relData))[relData$status==1 & relData$transplantCR1==1]
prdData$transplantCR1[sub("\\.1","",rownames(prdData)) %in% w] <- 1
prdData$transplantRel <- prdData$event
prdData$event <- NULL
w <- which(prdData$time1 == prdData$time2) ## 5 cases with LF=Rec
prdData$time2[w] <- prdData$time2[w] + .5
prdData$time0 <- as.numeric(clinicalData$Recurrence_date-clinicalData$CR_date)[prdData$index]
```

```
> table(is.na(prdData$time0))

FALSE  TRUE
  338   494
```

Error: This command creates many NAs incorrectly
prdData$time0 : time from CR1 to relapse
prdData$index was created by function CoxHD::MakeTimeDepedent() for *prdData*, and could not locate entries correctly in *clinicalData*
[Correction] *prdData$time0 <- as.numeric(clinicalData$Recurrence_date[i]-clinicalData$CR_date[i])[prdData$index]*

3.6.3 RFX fit of transitions

```
crGroups <- c(as.character(groups[whichRFXRel]), "Treatment","Treatment")
names(crGroups) <- c(names(dataFrame)[whichRFXRel],"transplantCR1","transplantRel")
coxRFXNrdTD <- CoxRFX(nrdData[names(crGroups)], Surv(nrdData$time1, nrdData$time2, nrdData$status), groups=crGrou
ps, which.mu = intersect(mainGroups, unique(crGroups)))
coxRFXNrdTD$coefficients["transplantRel"] <- 0
#prsData$time1[!is.na(prsData$time1)] <- 0
coxRFXPrdTD <-  CoxRFX(prdData[names(crGroups)], Surv(prdData$time1, prdData$time2, prdData$status), groups=crGro
ups, nu=1, which.mu = intersect(mainGroups, unique(crGroups)))
coxRFXRelTD <-  CoxRFX(relData[names(crGroups)], Surv(relData$time1, relData$time2, relData$status), groups=crGro
ups, which.mu = intersect(mainGroups, unique(crGroups)))
coxRFXRelTD$coefficients["transplantRel"] <- 0
```

3.6.3.1 OS

```
osData <- MakeTimeDependent(dataFrame[whichRFXRel], timeEvent=alloTimeCR1, timeStop=as.numeric(clinicalData$Date_
LF- clinicalData$CR_date), status=clinicalData$Status)
osData$transplantCR1 <- osData$event
osData$transplantRel <- osData$event
w <- which(clinicalData$TPL_date > clinicalData$Recurrence_date | clinicalData$TPL_Phase != "CR1")
osData$transplantCR1[osData$index %in% w] <- 0
osData$transplantRel[!osData$index %in% w] <- 0
data <- osData[rev(!duplicated(rev(osData$index))),colnames(coxRFXRelTD$Z)]
osData$transplantRel <- 0 # Note: confounded by relapse
rownames(data) <- sub("\\.1$","", rownames(data))
data <- data[rownames(dataFrame),]

coxRFXOsCR <- CoxRFX(osData[names(crGroups)], Surv(osData$time1, osData$time2, osData$status), groups=crGroups, w
hich.mu = intersect(mainGroups, unique(crGroups)))
```

3.6.3.2 Early deaths

```
table(CR=!is.na(clinicalData$CR_date), os[,2])
```

```
##
## CR         0   1
##   FALSE    7 264
##   TRUE   629 640
```

```
c <- as.numeric(clinicalData$CR_date - clinicalData$ERDate)
c[is.na(c)] <- clinicalData$OS[is.na(c)]
cr <- Surv(c, factor(pmin(2 * (!is.na(clinicalData$CR_date))+os[,2],2), levels=0:2, labels=c("cens","ED","CR")),
type="mstate")

coxRFXCrTD <- CoxRFX(osData[1:1540, names(crGroups)], Surv(cr[,1], cr[,2]==2), groups=crGroups, which.mu = inters
ect(mainGroups, unique(crGroups)))
coxRFXNcdTD <- CoxRFX(osData[1:1540, names(crGroups)], Surv(cr[,1], cr[,2]==1), groups=crGroups, which.mu = NULL)
```

3.6.4 Variance components

```
par(mfrow=c(3,2), xpd=FALSE)
o <- c(1,4,6,5,2,3,7,8)
PlotVarianceComponents(coxRFXNcdTD, col=colGroups, order=o)
title(main="Early deaths")
PlotVarianceComponents(coxRFXCrTD, col=colGroups, order=o)
title(main="Remission")
PlotVarianceComponents(coxRFXRelTD, col=colGroups, order=o)
title(main="Relapse")
PlotVarianceComponents(coxRFXNrdTD, col=colGroups, order=o)
title(main="Non-relapse deaths")
PlotVarianceComponents(coxRFXPrdTD, col=colGroups, order=o)
title(main="Post-relapse deaths")
```

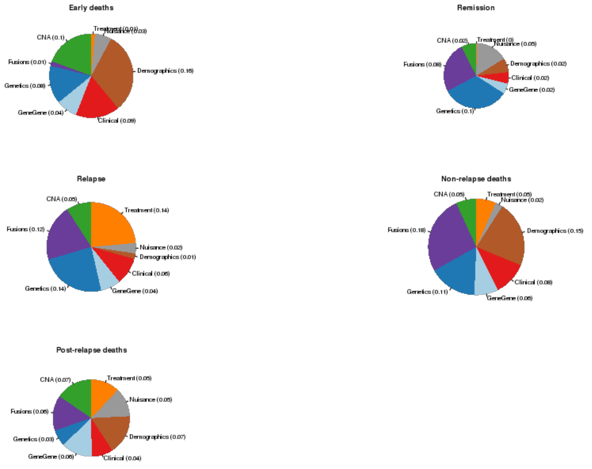

3.6.4.1 Figure 2F

As barplot

```
par(mar=c(4,3,1,5))
allVarComp <- sapply(c("NcdTD","CrTD","NrdTD","RelTD","PrdTD"), function(x){
            m <- get(paste0("coxRFX",x))
            Z <- get(sub("\\[.+","",as.character(m$call["data"])))
            i <- if(x%in%c("CrTD","EsTD")) 1:1540 else Z$index
            VarianceComponents(m, newZ=Z[!rev(duplicated(rev(i))),colnames(m$Z)])})
colnames(allVarComp) <- c("Early deaths","Remission","Non-relapse d.","Relapse","Post-relapse d.")
w <- c("CNA","Fusions","Genetics","GeneGene","Clinical","Demographics","Treatment","Nuisance")
z <- allVarComp[w,]#/rep(colSums(allVarComp[-9,]), each=8)
b <- barplot(z, col=colGroups[w], ylab="Variance [log hazard]", names.arg=rep("",ncol(z)))
rotatedLabel(x0=b, labels=colnames(z))
Z <- rbind(0,apply(z,2,cumsum))
n <- ncol(z)
segments(b[-n]+.5,t(Z[,-n]),b[-1]-.5 ,t(Z[,-1]))
```

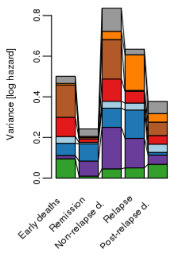


```
z <- allVarComp[w,]/rep(colSums(allVarComp[-9,]), each=8)
b <- barplot(z, col=colGroups[w], ylab="Relative importance", names.arg=rep("",ncol(z)))
rotatedLabel(x0=b, labels=colnames(z))
Z <- rbind(0,apply(z,2,cumsum))
n <- ncol(z)
segments(b[-n]+.5,t(Z[,-n]),b[-1]-.5 ,t(Z[,-1]))
mtext(side=4, at=Z[-1,n] - diff(Z[,n])/2, text=rownames(Z)[-1], las=2)
```

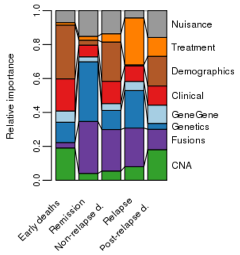


```
v <- c(1,3,5,4,2)
z <- allVarComp[w,v]/rep(colSums(allVarComp[-9,v]), each=8)
b <- barplot(z, col=colGroups[w], ylab="Relative importance", names.arg=rep("",ncol(z)))
rotatedLabel(x0=b, labels=colnames(z))
Z <- rbind(0,apply(z,2,cumsum))
n <- ncol(z)
segments(b[-n]+.5,t(Z[,-n]),b[-1]-.5 ,t(Z[,-1]))
mtext(side=4, at=Z[-1,n] - diff(Z[,n])/2, text=rownames(Z)[-1], las=2)
```

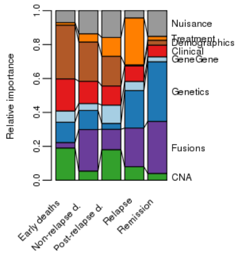

Pairwise scatter plots of the log hazard for each transition

```
allStagesRisk <- as.data.frame(sapply(c("NcdTD","CrTD","NrdTD","RelTD","PrdTD"), function(x){
                    m <- get(paste0("coxRFX",x))
                    #Z <- get(sub("\\[.+","",as.character(m$call["data"])))
                    #i <- if(x=="Cr") 1:1540 else Z$index
                    Z <- if(x=="Cr") dataFrame else data[rownames(dataFrame),]
                    predict(m, newdata=as.data.frame(Z))}))
f <- function(x,y,...) {points(x,y, col=densCols(x,y),...); lines(lowess(x,y), col='red')}
pairs(allStagesRisk, panel=f, pch=19)
```

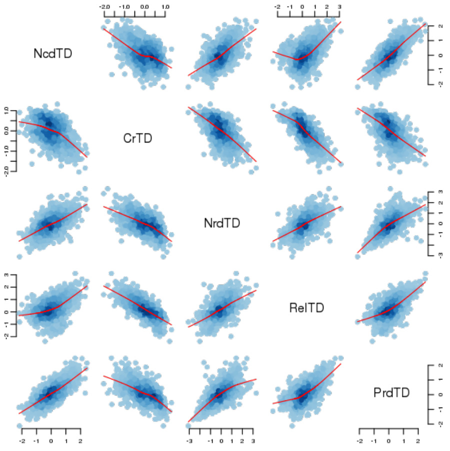

3.6.4.2 Supplementary Tables S2-6

Non-complete remission deaths

```
w <- WaldTest(coxRFXNcdTD)
w$Q.BH <- p.adjust(w$p.value, "BH")
w$Q.BY <- p.adjust(w$p.value, "BY")
datatable(w)
```

Show 10 **entries** **Search:**

	coef	sd	z	df	p.value	sig	Q.BH	
ASXL1	0.149428868672861	0.127268622337853	1.17412183716565	1	0.240346227858036		0.73385714905987	
ATRX	-0.0663396211357851	0.0628872626884677	-1.05489757861492	1	0.291472169812209		0.776129382406928	
BCOR	0.327111094194517	0.124496590537148	2.62747030085865	1	0.00860223506200936	**	0.179082893563649	
BRAF	0.16157037934428	0.0938874286054279	1.7208947112962	1	0.0852699314665005	.	0.513951559021519	
CBL	-0.0618736378188613	0.117536166816816	-0.526422117502721	1	0.598594951744326		0.867583822464877	
CBLB	-0.00672367750006618	0.0235365952690093	-0.285669079287745	1	0.775131589345255		0.948050011859948	
CDKN2A	-0.0170863395852059	0.0371222064414098	-0.460272737617938	1	0.645320467016772		0.915785349003242	
CREBBP	0.190415862391324	0.0706199744385933	2.69634567139213	1	0.00701048745300197	**	0.160540162673745	0.9
CUX1	-0.0755470242229227	0.105491375805407	-0.716144079514888	1	0.473902388240956		0.867583822464877	
DNMT3A	0.00956783245119924	0.114026387137819	0.0839089327598795	1	0.9331288372947		0.986268498977235	

Showing 1 to 10 of 231 entries Previous 1 2 3 4 5 … 24 Next

```
sheet  <- createSheet(wb, sheetName="Non-complete remission deaths")
addDataFrame(w,
        sheet,
        colnamesStyle = CellStyle(wb) + Font(wb, isBold=TRUE) + Border(),
        rownamesStyle = CellStyle(wb) + Font(wb, isBold=TRUE)
)
```

Complete remission

```
w <- WaldTest(coxRFXCrTD)
w$Q.BH <- p.adjust(w$p.value, "BH")
w$Q.BY <- p.adjust(w$p.value, "BY")
datatable(w)
```

Show 10 **entries** **Search:**

	coef	sd	z	df	p.value	sig	Q.BH	Q.
ASXL1	-0.196270437359024	0.10413528844876	-1.88476394777165	1	0.0594617144011408	.	0.406888914543051	
ATRX	-0.0738350207099324	0.0978389774230221	-0.754658548716174	1	0.450453883856731		0.806626722255076	
BCOR	-0.0408774410539759	0.114737658322759	-0.356268740808593	1	0.721639316738194		0.945657872919182	
BRAF	-0.0791746133480469	0.1014905619211	-0.780117991755704	1	0.435321427233093		0.806626722255076	

CBL	0.13731816084248	0.113025966789521	1.21492577982718	1	0.224394400309522	0.664552647070507
CBLB	-0.00267717325878776	0.0550169615392016	-0.0486608708276297	1	0.961189559468744	0.985765443554509
CDKN2A	0.00955077642794739	0.0693602552869643	0.137698115274129	1	0.89047900900447	0.97155385652999
CREBBP	0.0278312141393008	0.106332280284443	0.261738148235429	1	0.793523327445363	0.955684924796355
CUX1	-0.0501369718292377	0.0987003759814735	-0.507971437096133	1	0.61147337444613	0.899309211266298
DNMT3A	-0.105953146766679	0.0729567330716955	-1.45227372862979	1	0.146425509284254	0.554496600732176

Showing 1 to 10 of 231 entries Previous 1 2 3 4 5 … 24 Next

```
sheet  <- createSheet(wb, sheetName="Complete remission")
addDataFrame(w,
        sheet,
        colnamesStyle = CellStyle(wb) + Font(wb, isBold=TRUE) + Border(),
        rownamesStyle = CellStyle(wb) + Font(wb, isBold=TRUE)
)
```

Relapses

```
w <- WaldTest(coxRFXRelTD)
w$Q.BH <- p.adjust(w$p.value, "BH")
w$Q.BY <- p.adjust(w$p.value, "BY")
datatable(w)
```

Show 10 **entries** **Search:**

	coef	sd	z	df	p.value	sig	Q.BH	Q.BY
ASXL1	0.0845472128388816	0.149412337306159	0.565865003942993	1	0.571485553175704		0.836364726797809	1
ATRX	0.0937614312248909	0.125126899569313	0.749330731821995	1	0.453657889305742		0.748535517354474	1
BCOR	-0.276750893414432	0.165019606947334	-1.67707885465244	1	0.0935270630960872	.	0.415475991830695	1
BRAF	0.203035544377205	0.144282568190155	1.40720772387151	1	0.159365801968815		0.511298614649948	1
CBL	0.165611892336198	0.162982958298507	1.01613011608782	1	0.309567429488282		0.683131890695904	1
CBLB	0.0740913128328101	0.0875935534676397	0.845853489208908	1	0.397634481674517		0.742722109033578	1
CDKN2A	0.0759843799850096	0.0881048892293128	0.86243091217382	1	0.388450436040498		0.742722109033578	1
CREBBP	-0.249188135258821	0.16659751491564	-1.49574941369925	1	0.134718968199458		0.474553677367213	1
CUX1	0.251190494223937	0.139566404941591	1.79979196518719	1	0.0718934931395074	.	0.359449305819758	1
DNMT3A	0.199352339087902	0.106326717101432	1.87490354750375	1	0.0608059939464561	.	0.334432966705509	1

Showing 1 to 10 of 231 entries Previous 1 2 3 4 5 … 24 Next

```
sheet  <- createSheet(wb, sheetName="Relapse")
addDataFrame(w,
        sheet,
        colnamesStyle = CellStyle(wb) + Font(wb, isBold=TRUE) + Border(),
        rownamesStyle = CellStyle(wb) + Font(wb, isBold=TRUE)
)
```

Post-relapse survival

```
w <- WaldTest(coxRFXPrdTD)
w$Q.BH <- p.adjust(w$p.value, "BH")
w$Q.BY <- p.adjust(w$p.value, "BY")
datatable(w)
```

Show 10 **entries** **Search:**

	coef	sd	z	df	p.value	sig	Q.BH	Q.B
ASXL1	-0.00808571311340011	0.08636205974982	-0.0936257557638551	1	0.925406449336391		0.98623330713485	
ATRX	-0.00130194634273612	0.0544973841286402	-0.0238900703869179	1	0.980940294707315		0.98623330713485	
BCOR	-0.130989032947434	0.0807506457277952	-1.62214223510966	1	0.104772896712343		0.432188198938416	
BRAF	0.0440814042261358	0.0549646664141732	0.801995301744776	1	0.422555675983617		0.827206450442504	
CBL	-0.0230077988281729	0.0880739429370845	-0.261232755806203	1	0.793913019386202		0.98623330713485	

CBLB	0.0108898083497193	0.0422357881205187	0.257833672208165	1	0.796535276181069		0.98623330713485
CDKN2A	-0.00967610848999246	0.0493147923608831	-0.196211076367983	1	0.844444958877298		0.98623330713485
CREBBP	-0.0104050094171033	0.0502287884680506	-0.207152307161891	1	0.835890912730135		0.98623330713485
CUX1	-0.00563889271845611	0.0689496342763187	-0.0817827792364787	1	0.934819450374058		0.98623330713485
DNMT3A	-0.032589030207706	0.0840318556610655	-0.387817571697461	1	0.698151040959182		0.965705930907611

Showing 1 to 10 of 231 entries

Previous 1 2 3 4 5 … 24 Next

```
sheet  <- createSheet(wb, sheetName="Post-relapse deaths")
addDataFrame(w,
        sheet,
        colnamesStyle = CellStyle(wb) + Font(wb, isBold=TRUE) + Border(),
        rownamesStyle = CellStyle(wb) + Font(wb, isBold=TRUE)
)
```

Non-relapse deaths

```
w <- WaldTest(coxRFXNrdTD)
w$Q.BH <- p.adjust(w$p.value, "BH")
w$Q.BY <- p.adjust(w$p.value, "BY")
datatable(w)
```

Show 10 **entries** **Search:**

	coef	sd	z	df	p.value	sig	Q.BH	
ASXL1	0.399141174327162	0.154012332428057	2.59161826870982	1	0.00955257010537045	**	0.1268558207453	0.763[illegible]
ATRX	0.121756751856336	0.102466541808015	1.18825862284358	1	0.234731533958758		0.643643860293093	
BCOR	0.0178427620375905	0.151324600922574	0.117910517713639	1	0.906138559455887		0.953905834986118	
BRAF	0.0608134409278527	0.0847618472835341	0.717462430053319	1	0.473088810243276		0.769515122604177	
CBL	-0.16290957771924	0.154277129791742	-1.05595416468502	1	0.290989156087731		0.659647333919374	
CBLB	0.0789795731679614	0.0735321920632828	1.07408158184636	1	0.282786118667348		0.653722521412034	
CDKN2A	0.0474432585290746	0.0905314676420082	0.524052683169582	1	0.600241897578797		0.810853089711708	
CREBBP	0.164522854415739	0.123925140957182	1.32759868695718	1	0.184310718558164		0.574988569927507	
CUX1	0.0497877221794363	0.0908489641613481	0.548027406135453	1	0.583673084793499		0.810853089711708	
DNMT3A	-0.156630000639269	0.137948924761574	-1.13542023549645	1	0.256199285126322		0.643643860293093	

Showing 1 to 10 of 231 entries

Previous 1 2 3 4 5 … 24 Next

```
sheet  <- createSheet(wb, sheetName="Non-relapse deaths")
addDataFrame(w,
        sheet,
        colnamesStyle = CellStyle(wb) + Font(wb, isBold=TRUE) + Border(),
        rownamesStyle = CellStyle(wb) + Font(wb, isBold=TRUE)
)


saveWorkbook(wb, file="SupplementaryTables.xlsx")
```

3.6.5 Predicting outcome from diagnosis

The following function fits a 5-stage model. Note that we use a single smooth function g(t) to model the association between time of CR and all subsequent events. It is implemented in C++ for efficiency using the `Rcpp` package (Eddelbuettel and Francois 2011).

```
library(Rcpp)
MultiRFX5 <- function(coxRFXNcdTD, coxRFXCrTD, coxRFXNrdTD, coxRFXRelTD, coxRFXPrdTD, data, x =365, tdPrmBaseline
= rep(1, ceiling(max(x))+1), tdOsBaseline = rep(1, ceiling(max(x))+1), ciType="analytical"){
    cppFunction('NumericVector computeHierarchicalSurvival(NumericVector x, NumericVector diffS0, NumericVector S
1Static, NumericVector haz1TimeDep) {
                    int xLen = x.size();
                    double h;
                    NumericVector overallSurvival(xLen);
                    for(int i = 0; i < xLen; ++i) overallSurvival[i] = 1;
                    for(int j = 1; j < xLen; ++j){
                    h = haz1TimeDep[j-1];
                    for(int i = j; i < xLen; ++i){
                    overallSurvival[i] += diffS0[j-1] * (1-pow(S1Static[i-j], h));
                    }
                    }
                    return overallSurvival;
                    }')



    ## Step 1: Compute KM survival curves and log hazard
    getS <- function(coxRFX, data, max.x=5000) {
        if(!is.null(coxRFX$na.action)) coxRFX$Z <- coxRFX$Z[-coxRFX$na.action,]
        data <- as.matrix(data[,match(colnames(coxRFX$Z),colnames(data)), drop=FALSE])
        r <- PredictRiskMissing(coxRFX, data, var="var2")
        H0 <- basehaz(coxRFX, centered = FALSE)
        hazardDist <- splinefun(H0$time, H0$hazard, method="monoH.FC")
        x <- c(0:ceiling(max.x))
        S <- exp(-hazardDist(x))
        return(list(S=S, r=r, x=x, hazardDist=hazardDist, r0 = coxRFX$means %*% coef(coxRFX)))
    }
    kmCr <- getS(coxRFX = coxRFXCrTD, data = data, max.x=max(x))
    kmEs <- getS(coxRFX = coxRFXNcdTD, data = data, max.x=max(x))
    kmCir <- getS(coxRFX = coxRFXRelTD, data = data, max.x=max(x))
    kmNrm <- getS(coxRFX = coxRFXNrdTD, data = data, max.x=max(x))
    kmPrs <- getS(coxRFX = coxRFXPrdTD, data = data, max.x=max(x))

    xx <- 0:ceiling(max(x))

    sapply(1:nrow(data), function(i){
                ## Step 2: Adjust curves for competing risks, accounting for hazard
                crAbs <-  cumsum(c(1,diff(kmCr$S^exp(kmCr$r[i,1])))) * kmEs$S ^ exp(kmEs$r[i,1]))
                esAbs  <- cumsum(c(1,diff(kmEs$S^exp(kmEs$r[i,1])))) * kmCr$S ^ exp(kmCr$r[i,1])) ## array times x
nrow(data)
                cirCrAbs <- cumsum(c(1,diff(kmCir$S^exp(kmCir$r[i,1])))) * kmNrm$S ^ exp(kmNrm$r[i,1]))
                nrsCrAbs <- cumsum(c(1,diff(kmNrm$S^exp(kmNrm$r[i,1])))) * kmCir$S ^ exp(kmCir$r[i,1])) ## array t
imes x nrow(data)

                ## Step 3: Compute hierarchical survival
                ### Prs
                rsCrAbs <- computeHierarchicalSurvival(x = xx, diffS0 = diff(cirCrAbs), S1Static = kmPrs$S, haz1T
imeDep = tdPrmBaseline * exp(kmPrs$r[i,1]))

                ## Confidence intervals (loglog)
                PlogP2 <- function(x) {(x * log(x))^2}
                errOs <- kmNrm$r[i,2] * PlogP2(kmNrm$S^exp(kmNrm$r[i,1])) * (1-(1-kmCir$S ^ exp(kmCir$r[i,1]))) *
(1-kmPrs$S ^ exp(kmPrs$r[i,1]))^2 + kmCir$r[i,2] * PlogP2(kmCir$S ^ exp(kmCir$r[i,1])) * (1-kmPrs$S ^ exp(kmPrs$r
[i,1]))^2 * (kmNrm$S ^ exp(kmNrm$r[i,1]))^2 +  kmPrs$r[i,2] * PlogP2(kmPrs$S ^ exp(kmPrs$r[i,1])) * (1-kmCir$S ^
exp(kmCir$r[i,1]))^2 * (kmNrm$S ^ exp(kmNrm$r[i,1]))^2
                sdOsCr <- sqrt(errOs / PlogP2(1-(1-nrsCrAbs)-(1-rsCrAbs)))


                ### Overall survival from enrollment
                nrsEr <- computeHierarchicalSurvival(x = xx, diffS0 = diff(crAbs), S1Static = nrsCrAbs, haz1TimeD
ep = tdOsBaseline)
                rsEr <- computeHierarchicalSurvival(x = xx, diffS0 = diff(crAbs), S1Static = rsCrAbs, haz1TimeDep
= tdOsBaseline)
                cirEr <- computeHierarchicalSurvival(x = xx, diffS0 = diff(crAbs), S1Static = cirCrAbs, haz1TimeD
ep = tdOsBaseline)
                cbind(deathInErFromEr=1-esAbs, deathInCrFromEr=1-nrsEr, deathInRelFromEr=1-rsEr, aliveInRelFromEr
=1-cirEr-(1-rsEr), aliveInCrFromEr=1-crAbs - (1-cirEr) - (1-nrsEr),
                        deathInCrFromCr = 1-nrsCrAbs, deathInRelapseFromCr=(1-rsCrAbs), aliveInRelapseFromCr = (1
-cirCrAbs) - (1-rsCrAbs), osInCrFromCrSd=sdOsCr
                )
            }, simplify='array')
}
```

PRS baseline with spline-based dep on CR length)

```
xmax <- 2000
xx <- 0:ceiling(xmax)
coxphPrs <- coxph(Surv(time1, time2, status)~ pspline(time0, df=10), data=data.frame(prdData, time0=as.numeric(cl
inicalData$Recurrence_date-clinicalData$CR_date)[prdData$index]))
tdPrmBaseline <- exp(predict(coxphPrs, newdata=data.frame(time0=xx[-1]))) ## Hazard (function of CR length)

coxphOs <- coxph(Surv(time1,time2, status)~ pspline(time0, df=10), data=data.frame(osData, time0=pmin(500,cr[osDa
ta$index,1])))
tdOsBaseline <- exp(predict(coxphOs, newdata=data.frame(time0=xx[-1])))  ## Hazard (function of induction length)
, only for OS (could do CIR,NRM,PRS seperately)

fiveStagePredicted <- MultiRFX5(coxRFXNcdTD, coxRFXCrTD, coxRFXNrdTD, coxRFXRelTD, coxRFXPrdTD, data, tdPrmBaseli
ne = tdPrmBaseline, tdOsBaseline = tdOsBaseline, x=xmax)
```

Function to plot stages

```
sedimentPlot <- function(Y, x=1:nrow(Y), y0=0, y1=NULL, col=1:ncol(Y), ...){
    Z <- cbind(t(apply(cbind(y0,Y),1,cumsum)),y1)
    plot(x,Z[,1], xlim=range(x), ylim=range(Z), lty=0, pch=NA,...)
    for(i in 2:ncol(Z))
        polygon(c(x,rev(x)), c(Z[,i-1],rev(Z[,i])), border=NA, col=col[i-1])
}

lineStage <- function(CR_date, Recurrence_date, Date_LF, ERDate, Status, y=0, col=1:5, pch.trans=19, pch.end=19,
...){
    xpd <- par("xpd")
    par(xpd=NA)
    t <- as.numeric(c(CR_date, Recurrence_date, Date_LF) - ERDate )
    w <- !is.na(t)
    o <- order(t)
    to <- pmin(t[o], par("usr")[2])
    l <- length(to)
    segments(c(0,to[-l]), rep(y,l), to, rep(y,l), col=col, lend=1, ...)
    status <- if(Status == 1) 3 else 0
    if(is.na(Recurrence_date))
        status <- status - 1
    if(is.na(CR_date))
        status <- status - 1
    x <- ifelse(t <= par("usr")[2], t, NA)
    points(x, rep(y, length(t)), pch=c(pch.trans,pch.trans, if(Status) pch.end else NA), col=col[c(2:3,status+3)]
)
    par(xpd=xpd)
}
```

Average of all multistage predictions, note the precise agreement with overall survival.

```
pastel1 <- brewer.pal(9, "Pastel1")
par(mfrow=c(1,1), mar=c(3,3,1,1), cex=1)
sedimentPlot(-rowMeans(fiveStagePredicted[,1:5,], dims=2), y0=1, y1=0,  col=c(pastel1[c(1:3,5,4)], "#DDDDDD"))
lines(survfit(Surv(OS, Status) ~ 1, data=clinicalData))
```

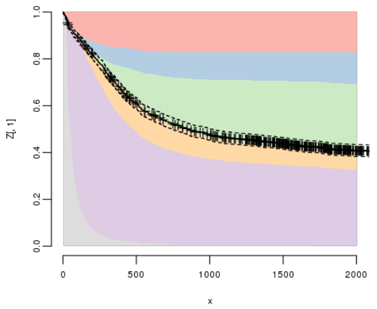

Multistage predictions v overall survival

```
for(i in 1:5)
    plot(summary(survfit(coxRFXFitOsTDGGc), i*365)$surv^ exp(coxRFXFitOsTDGGc$linear.predictors[1:1540]), 1-rowSu
ms(aperm(fiveStagePredicted[,1:3,], c(3,1,2)), dim=2)[,365*3],
            xlab="Survival RFX OS", ylab="Survival RFX Multistage", main=paste(i, "years"))
```

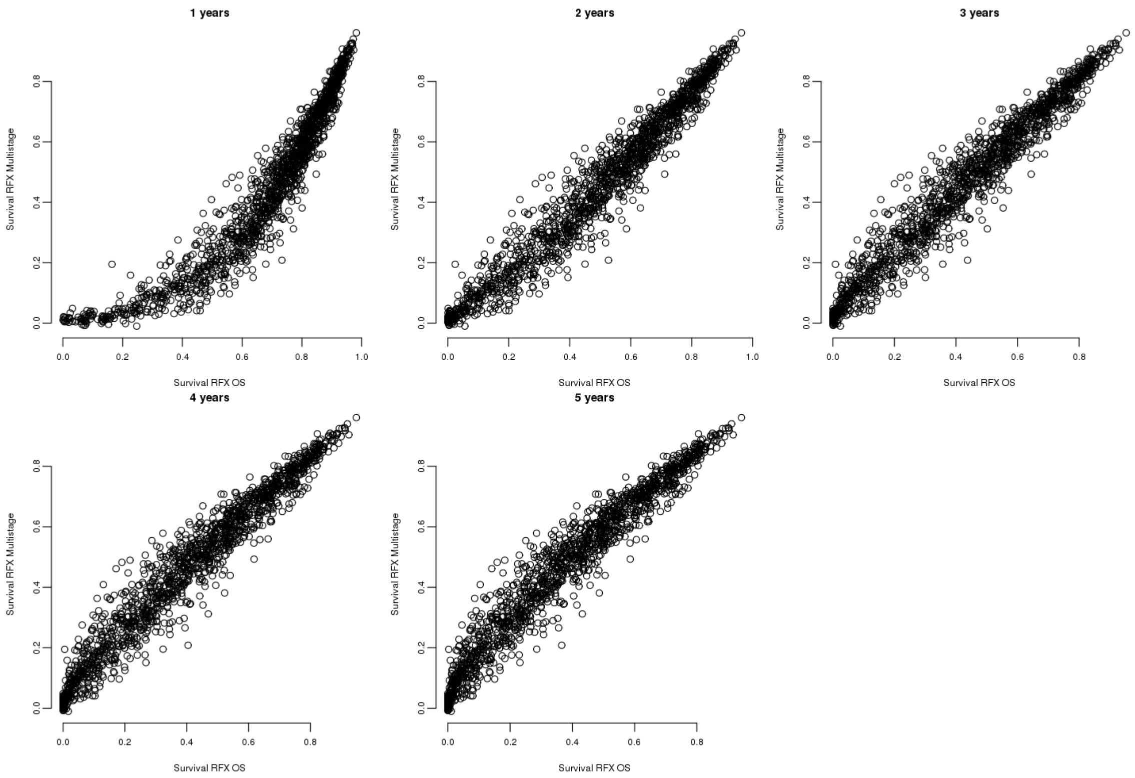

3.6.5.1 Leave-one-out cross-validation

Codes in sections 3.6.5.1–3.6.5.9 rely on an LSF environment for parallel computing purposes. We were not able to tailor the entire R script to our own environments, as the computations are very intensive, and hence the modifications are prone to errors.

The following code is run on the cluster

```
read_chunk('../code/leaveOneOut.R', labels="leaveOneOut")
```

```
load("loo.RData")
library(mg14)
library(CoxHD)
library(Rcpp)

#save(dataFrame, nrdData, crGroups, mainGroups, prdData, relData, prdData, osData, cr, dataFrameOsTD, osTD, tplSp
litOs, groups, data, whichRFXOsTDGG, clinicalData, MultiRFX5,  file="../../code/loo.RData")

jobIndex <- as.numeric(Sys.getenv("LSB_JOBINDEX"))

cvIdx <- 1:nrow(dataFrame)
whichTrain <- which(cvIdx != jobIndex)
rfxNrs <- CoxRFX(nrdData[nrdData$index %in% whichTrain, names(crGroups)], Surv(nrdData$time1, nrdData$time2, nrdD
ata$status)[nrdData$index %in% whichTrain], groups=crGroups, which.mu = intersect(mainGroups, unique(crGroups)))
rfxNrs$coefficients["transplantRel"] <- 0
#prsData$time1[!is.na(prsData$time1)] <- 0
rfxPrs <-  CoxRFX(prdData[prdData$index %in% whichTrain, names(crGroups)], Surv(prdData$time1, prdData$time2, prd
Data$status)[prdData$index %in% whichTrain], groups=crGroups, nu=1, which.mu = intersect(mainGroups, unique(crGro
ups)))
rfxRel <-  CoxRFX(relData[relData$index %in% whichTrain, names(crGroups)], Surv(relData$time1, relData$time2, rel
Data$status)[relData$index %in% whichTrain], groups=crGroups, which.mu = intersect(mainGroups, unique(crGroups)))
rfxRel$coefficients["transplantRel"] <- 0
rfxCr <- CoxRFX(osData[whichTrain, names(crGroups)], Surv(cr[,1], cr[,2]==2)[whichTrain], groups=crGroups, which.
mu = intersect(mainGroups, unique(crGroups)))
rfxEs <- CoxRFX(osData[whichTrain, names(crGroups)], Surv(cr[,1], cr[,2]==1)[whichTrain], groups=crGroups, which.
mu = NULL)

ix <- tplSplitOs %in% whichTrain
rfxOs <- CoxRFX(dataFrameOsTD[ix,whichRFXOsTDGG], osTD[ix], groups[whichRFXOsTDGG], which.mu=mainGroups) ## allow
only the main groups to have mean different from zero..

xx <- 0:2000
coxphPrs <- coxph(Surv(time1, time2, status)~ pspline(time0, df=10), data=data.frame(prdData, time0=as.numeric(cl
inicalData$Recurrence_date-clinicalData$CR_date)[prdData$index])[prdData$index %in% whichTrain,])
tdPrmBaseline <- exp(predict(coxphPrs, newdata=data.frame(time0=xx[-1])))

coxphOs <- coxph(Surv(time1, time2, status)~ pspline(time0, df=10), data=data.frame(osData, time0=pmin(500,cr[osD
ata$index,1]))[osData$index %in% whichTrain,])
tdOsBaseline <- exp(pmin(predict(coxphOs, newdata=data.frame(time0=500)),predict(coxphOs, newdata=data.frame(time
0=xx[-1])))) ## cap predictions at induction length 500 days.
multiRfx5 <- MultiRFX5(rfxEs, rfxCr, rfxNrs, rfxRel, rfxPrs, data[cvIdx == jobIndex,,drop=FALSE], tdPrmBaseline =
tdPrmBaseline, tdOsBaseline = tdOsBaseline, x=2000)

save(rfxEs, rfxCr, rfxEs, rfxNrs, rfxPrs, rfxRel, rfxOs, multiRfx5, file=paste0("loo/",jobIndex,".RData"))
```

Multistage model

```
times <- round(seq(0,5,0.05)*365)
multiRfx5Loo <- sapply(mclapply(1:nrow(data), function(i){
                    e <- new.env()
                    t <- try(load(paste0("../code/loo/",i,".RData"), env=e))
                    if(class(t)=="try-error") rep(NA, length(times))
                    else e$multiRfx5[times+1,,1]
                }, mc.cores=6), I, simplify="array")
```

Error OS

```
survConcordance(os ~ colSums(multiRfx5Loo[times == 3*365,1:3,]))
```

```
## Call:
## survConcordance(formula = os ~ colSums(multiRfx5Loo[times ==
##     3 * 365, 1:3, ]))
##
##   n= 1540
## Concordance= 0.7237308 se= 0.01003199
## concordant discordant  tied.risk  tied.time   std(c-d)
##  687786.00  262548.00       0.00     432.00   19067.49
```

```
ape(1-colSums(multiRfx5Loo[times == 3*365,1:3,]), os, 3*365)
```

```
##       abs     brier      log2     bayes
## 0.3684970 0.1836208 0.7614532 0.2818182
```

3.6.5.2 Figure 3

We plot all predictions as sediments plots, laid out in the same way as the risk constellation plot, Supplementary Figure S2C

```
set.seed(42)
s <- sample(nrow(dataFrame),nStars^2) #1:(nStars^2)
library(HilbertVis)
nStars <- 32
l <- "coxRFXFitOsTDGGc"
t <- os#get(l)$surv
p <- PartialRisk(get(l),  newZ=dataFrame[, colnames(get(l)$Z)])
p <- p[,colnames(p)!="Nuisance"]
locations <- hilbertCurve(log2(nStars))+1
mat <- matrix(order(locations[,1], locations[,2]), ncol=nStars)
h <- hclust(dist(p[s,]))
layout(mat[nStars:1,])
par(mar=c(0,0,0,0),+.5, bty="n")
for(i in 1:nStars^2){ # Fitted predictions
    sedimentPlot(-fiveStagePredicted[seq(1,2001,200),1:5,s[h$order[i]]], x=seq(1,2001,200),y0=1, y1=0,  col=c(pas
tel1[c(1:3,5,4)], "#DDDDDD"), xlab="time",ylab="fraction", xaxt="n", yaxt="n")
    lines(x=seq(1,2001,200), y=1-rowSums(fiveStagePredicted[seq(1,2001,200),1:3,s[h$order[i]]]), lwd=2)
    i <- s[h$order[i]]
    lineStage(clinicalData$CR_date[i], clinicalData$Recurrence_date[i], clinicalData$Date_LF[i], clinicalData$ERD
ate[i], clinicalData$Status[i], col=c(brewer.pal(8,"Dark2")[8], set1[c(4:5,1:3)]), lwd=2, pch.trans=NA, y=0.05)
}
```

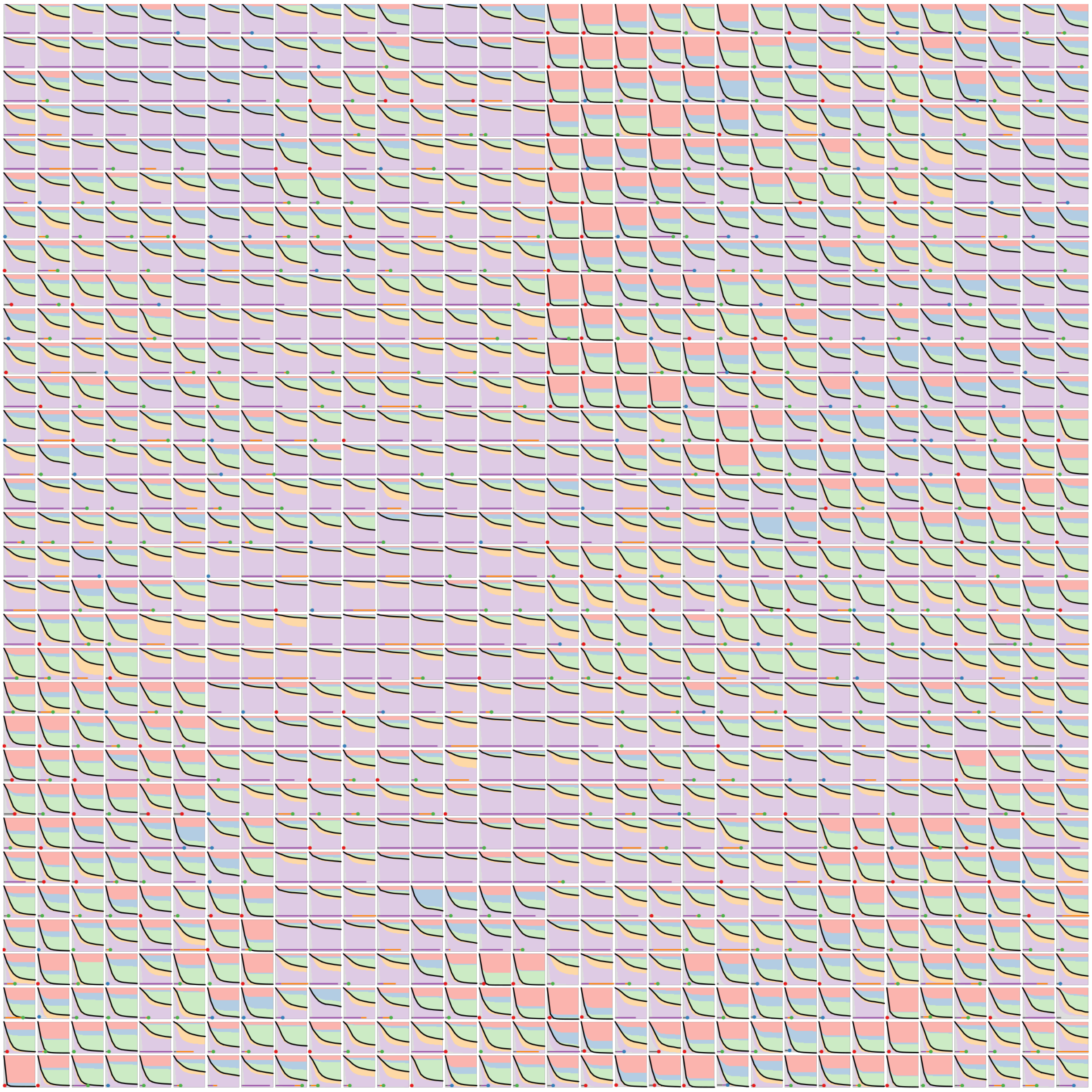

```
for(i in 1:nStars^2){ # Leave-one-out predictions
    sedimentPlot(-multiRfx5Loo[seq(1,length(times),5),1:5,s[h$order[i]]], x=times[seq(1,length(times),5)],y0=1, y
1=0,  col=c(pastel1[c(1:3,5,4)], "#DDDDDD"), xlab="time",ylab="fraction", xaxt="n", yaxt="n")
    lines(x=times[seq(1,length(times),5)], y=1-rowSums(multiRfx5Loo[seq(1,length(times),5),1:3,s[h$order[i]]]), l
wd=2)
    i <- s[h$order[i]]
    lineStage(clinicalData$CR_date[i], clinicalData$Recurrence_date[i], clinicalData$Date_LF[i], clinicalData$ERD
ate[i], clinicalData$Status[i], col=c(brewer.pal(8,"Dark2")[8], set1[c(4:5,1:3)]), lwd=2, pch.trans=NA, y=0.05)
}
```

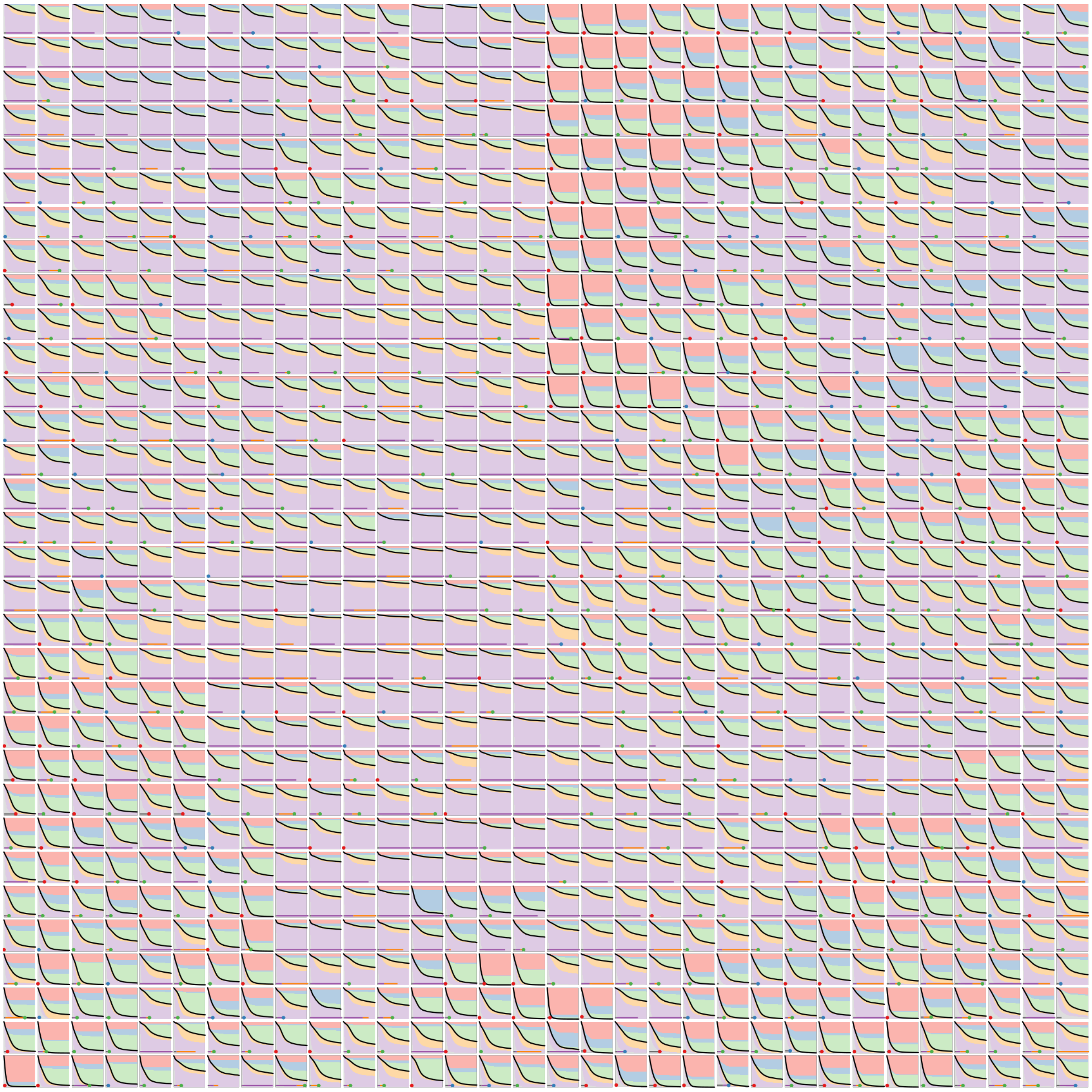

3.6.5.3 Comparison with RFX

```
rfx5Loo <- sapply(mclapply(1:nrow(data), function(i){
                    e <- new.env()
                    t <- try(load(paste0("../code/loo/",i,".RData"), env=e))
                    if(class(t)=="try-error") rep(NA, length(times))
                    else {
                        cvIdx <- 1:nrow(dataFrame)
                        whichTrain <<- which(cvIdx != i)
                        pNrs <- predict(e$rfxNrs, newdata=data[cvIdx==i,])
                        pRel <- predict(e$rfxRel, newdata=data[cvIdx==i,])
                        pPrs <- predict(e$rfxPrs, newdata=data[cvIdx==i,])
                        pCr <- predict(e$rfxCr, newdata=data[cvIdx==i,])
                        pEs <- predict(e$rfxEs, newdata=data[cvIdx==i,])
                        pOs <- predict(e$rfxOs, newdata=dataFrame[cvIdx==i,])
                        c(pCr, pEs, pNrs, pRel, pPrs, pOs)
                    }
                }, mc.cores=6), I, simplify="array")

colnames(rfx5Loo) <- rownames(data)
survConcordance(Surv(nrdData$time1, nrdData$time2, nrdData$status) ~ rfx5Loo[3,nrdData$index])
```

```
## Call:
## survConcordance(formula = Surv(nrdData$time1, nrdData$time2,
##     nrdData$status) ~ rfx5Loo[3, nrdData$index])
##
##   n=1609 (271 observations deleted due to missingness)
## Concordance= 0.6652892 se= 0.02393788
## concordant discordant  tied.risk  tied.time   std(c-d)
##  96115.000  48356.000      0.000     16.000   6916.659
```

```
survConcordance(Surv(prdData$time1, prdData$time2, prdData$status) ~ rfx5Loo[5,rownames(prdData)[prdData$index]])
```

```
## Call:
## survConcordance(formula = Surv(prdData$time1, prdData$time2,
##     prdData$status) ~ rfx5Loo[5, rownames(prdData)[prdData$index]])
##
##   n= 832
## Concordance= 0.6768913 se= 0.01433183
## concordant discordant  tied.risk  tied.time   std(c-d)
## 117928.000  56292.000      0.000    259.000   4993.785
```

```
survConcordance(Surv(relData$time1, relData$time2, relData$status) ~ rfx5Loo[4,relData$index])
```

```
## Call:
## survConcordance(formula = Surv(relData$time1, relData$time2,
##     relData$status) ~ rfx5Loo[4, relData$index])
##
##   n=1609 (271 observations deleted due to missingness)
## Concordance= 0.6437934 se= 0.01212035
## concordant discordant  tied.risk  tied.time   std(c-d)
##  336890.00  186399.00       0.00     272.00   12684.89
```

```
survConcordance(Surv(cr[,1], cr[,2]==2) ~ rfx5Loo[1,])
```

```
## Call:
## survConcordance(formula = Surv(cr[, 1], cr[, 2] == 2) ~ rfx5Loo[1,
##     ])
##
##   n= 1540
## Concordance= 0.637342 se= 0.009305283
## concordant discordant  tied.risk  tied.time   std(c-d)
##  642298.00  365478.00       0.00   11815.00   18755.28
```

```
survConcordance(Surv(cr[,1], cr[,2]==1) ~ rfx5Loo[2,])
```

```
## Call:
## survConcordance(formula = Surv(cr[, 1], cr[, 2] == 1) ~ rfx5Loo[2,
##     ])
##
##   n= 1540
## Concordance= 0.689559 se= 0.02411522
## concordant discordant  tied.risk  tied.time   std(c-d)
## 114222.000  51423.000      0.000    151.000   7989.131
```

```
survConcordance(os ~ rfx5Loo[6,])
```

```
## Call:
## survConcordance(formula = os ~ rfx5Loo[6, ])
##
##   n= 1540
## Concordance= 0.7238402 se= 0.01003199
## concordant discordant  tied.risk  tied.time   std(c-d)
##  687890.00  262444.00       0.00     432.00   19067.49
```

3.6.5.4 Figure 2C

Model schematic with heatmaps of all coefficients, all data and resulting logfc transition rates

```
layout(matrix(c(1,2,4,3), nrow=2), width=c(3,1),height=c(1,4))
par(bty="n", mar=c(0,3,3,2), mgp=c(2,.5,0), tcl=-.25)

allCoef <- sapply(c("NcdTD","CrTD","NrdTD","RelTD","PrdTD"), function(x){
            m <- get(paste0("coxRFX",x))$coef})

s <- c(Fusions=1, CNA=2, Genetics=3, GeneGene=4, Clinical=5, Demographics=6, Treatment=7, Nuisance=8)
o <- order(s[crGroups], 1/apply(allCoef,1,var)/apply(data,2,var))
image(y=1:5, x=1:nrow(allCoef), z=as.matrix(allCoef[o,]), useRaster=TRUE, col=colorRampPalette(rev(brewer.pal(9,"
RdBu")))(100), xaxt="n", ylab="transition")
t <- table(crGroups)[names(s)]
par(xpd=NA)
segments(x0=cumsum(c(0.5, t))[-length(t)-1] , x1=cumsum(t)+.5, col=colGroups[names(s)], y0=6, y1=6, lend=1, lwd=2
)

colRamp <- sapply(colGroups[names(s)], function(x) c(colorRampPalette(c("white",x))(11)[-1]))

par(bty="L", mar=c(3,3,1,2))
times <- round(seq(0,5,0.05)*365)
p <- order(colSums(multiRfx5Loo[times == 3*365,1:3,]))
Z <- data[p,o]
Z <- t(Z) + apply(Z,2,min)
Z <- Z/apply(Z,1,max)

image(x=1:nrow(Z),y=1:ncol(Z),Z*.9 + as.numeric(s[crGroups][o])-1 + 1e-5, useRaster=TRUE,
        col=colRamp,
        breaks=seq(0,length(unique(crGroups)), 0.1),  ylab="Patients",xlab="Variable", xlim=c(0,nrow(Z)), ylim=c(
0,ncol(Z)))

par(bty="n", mar=c(3,0,1,2))
image(x=1:5, z=t(as.matrix(allStagesRisk[p,]) - rep(colMeans(allStagesRisk), each=ncol(allStagesRisk))), useRaste
r=TRUE, col=rev(brewer.pal(9,"RdBu")), yaxt="n", xlab="rate (logFC)")
```

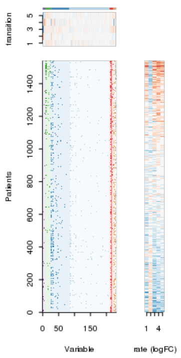

3.6.5.5 Figure 2D

Plot of absolute risk at 3yr, leave-one-out cross validated v outcome

```
par(mar=c(3,3,2,1), mgp=c(1.5,.5,0), bty="n")
t <- os
q <- quantile(t[,1], seq(0,1,.1))# q <- splinefun( s$surv, s$time,"monoH.FC")(seq(1,min(s$surv),l=10))
c <- cut(t[,1], q, na.rm=TRUE)
h <- colSums(multiRfx5Loo[times == 3*365,1:3,])
o <- order(h)
plot(1-h[o], col= (brewer.pal(10,'RdBu'))[c[o]], type='h', xaxt="n", xlab='', las=2, ylab="Survival at 3 years")
mtext(side=1, line=1, "Patient")
u <- par("usr")
q <- pmin(q,365*12)
image(x=q/max(q)*500, y=c(u[4]-(u[4]-u[3])/20, u[4]), matrix(1:10), col= (brewer.pal(10,'RdBu')), add=TRUE)
#axis(side=3, at=seq(1,500,l=11), labels=seq(0,1,.1))
axis(side=3, at=pretty(q/365)/max(q)*365*500, labels=pretty(q/365))
lines(ksmooth(seq_along(o),t[o,2]==0, bandwidth=50))
```

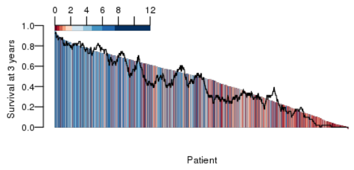

3.6.5.6 Figure 2E

```
par(mar=c(3,5,3,1), mgp=c(2,.5,0), las=2)
s <- 1-colSums(multiRfx5Loo[times == 3*365,1:3,])
surv365Quantiles <- cut(s, seq(0,1,0.25), include.lowest=TRUE, labels=c("0-25%","25-50%","50-75%","75-100%"))
eln <- factor( paste(clinicalData$M_Risk))
t <- table(`ELN risk group`=eln,`3-year survival`=surv365Quantiles)[5:1,4:1]
mosaicplot(t, col=RColorBrewer::brewer.pal(9,"Set1")[c(3,2,4,1,9)], dir=c("v","h"), main="")
```

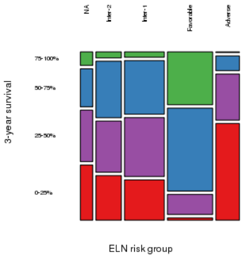

3.6.5.7 Figure 1B

Plots of concordance and absolute prediction errors for baseline error, ELN, RFX and multistage models.

```
multiRfx5C <- sapply(seq_along(times), function(i) survConcordance(os ~ colSums(multiRfx5Loo[i,1:3,]))$concordanc
e[1])

plot(times/365.25, multiRfx5C, type='l', xlab="Time", ylab="Concordance", ylim=c(0.5, 0.73), col=set1[1])
abline(h=survConcordance(os ~ rfx5Loo[6,])$concordance, col=set1[2], lwd=1)
abline(h=survConcordance(os ~ predict(coxph(os ~ eln)))$concordance, col=set1[3], lwd=1)

legend("bottomright",c("ELN","RFX OS","RFX Multistage"), col=set1[c(3:1)], lty=1, bty="n")
```

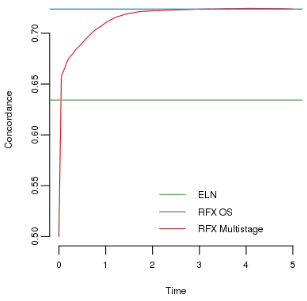


```
ss <- sapply(levels(eln),function(e) summary(survfit(os ~ 1, subset=eln==e), times=times)$surv)
ee <- sapply(times, function(t) ape(ss[times==t,eln], os, t))

a <- sapply(times, function(t) ape(1-colSums(multiRfx5Loo[times == t,1:3,]), os, t))
s <- summary(survfit(coxRFXFitOsTDGGc), times=times)
b <- sapply(times, function(t) ape(s$surv[times==t]^exp(rfx5Loo[6,]), os, t))
e <- sapply(times, function(t) ape(s$surv[times==t], os, t))
for(i in 1:4){
    plot(times/365.25, e[i,], type='l', xlab="Time (yr)", ylab=rownames(a)[i], col=set1[9])
    lines(times/365.25, a[i,], col=set1[1])
    lines(times/365.25, b[i,], col=set1[2])
    lines(times/365.25, ee[i,], col=set1[3])
    legend("bottomright",c("Kaplan-Meier","ELN","Multistage","RFX OS"), col=set1[c(9,3,1:2)], lty=1, bty="n")
}
```

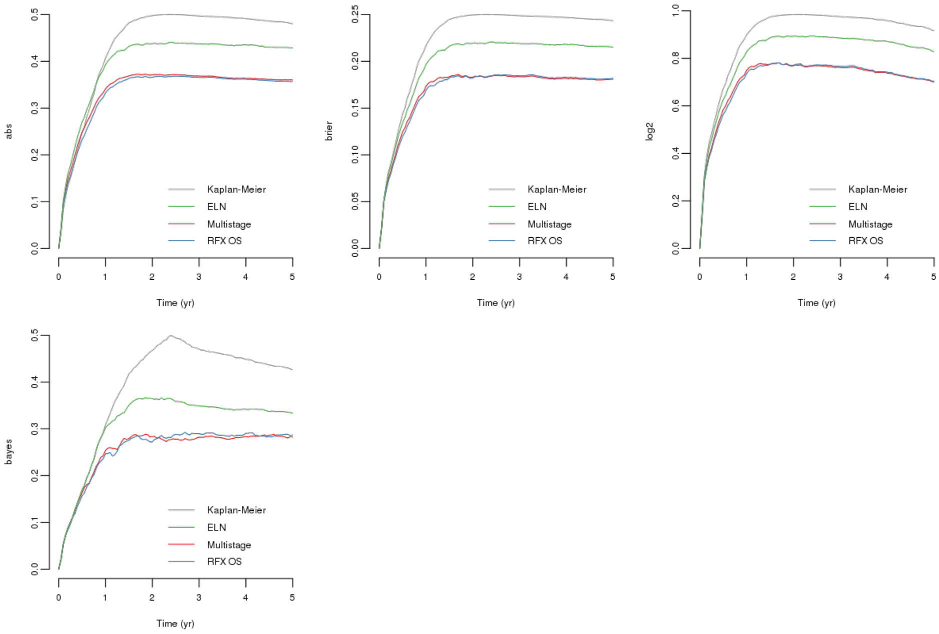

```
i <- 2; ## R2
plot(times/365.25, 1 - a[i,]/e[i,], type='l', xlab="Time (yr)", ylab="R2", col=set1[1], ylim=c(0,.3))
lines(times/365.25,  1 - b[i,]/e[i,], col=set1[2])
lines(times/365.25,  1 - ee[i,]/e[i,], col=set1[3])
legend("bottomright",c("ELN","Multistage","RFX OS"), col=set1[c(3,1:2)], lty=1, bty="n")
```

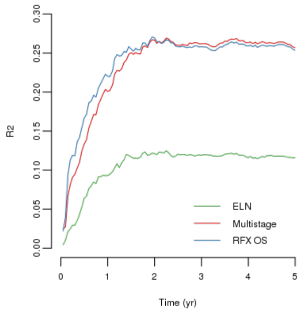

Figure of predicted survival for 100 patients, comparing multistage and OS predictions

```
plot(s$surv^exp(rfx5Loo[6,1]), 1-rowSums(multiRfx5Loo[,1:3,1]), type='l', xlim=c(0,1), ylim=c(0,1), col='grey', x
lab="Predicted survival RFX", ylab="Pedicted survival Multistage")
for(i in 2:100)
    lines(s$surv^exp(rfx5Loo[6,i]), 1-rowSums(multiRfx5Loo[,1:3,i]), col='grey')
```

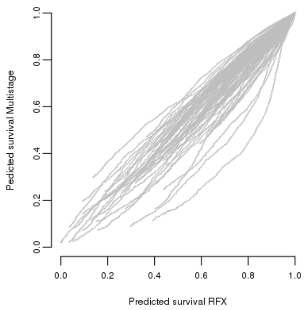

Data with and without allograft

```
allDataTpl <- osData[rep(1:nrow(dataFrame), each=3),]
allDataTpl$transplantCR1 <- rep(c(0,1,0), nrow(dataFrame))
allDataTpl$transplantRel <- rep(c(0,0,1), nrow(dataFrame))
```

3.6.5.8 Figure 4

Here we show survival predictions for three selected patients

```r
xmax=2000
patients <- c("PD11104a","PD8314a","PD10941a")
layout(matrix(1:(3*length(patients)), byrow=TRUE, ncol=3), width=c(0.5,1,1))
par(mar=c(3,3,1,1), bty="n", mgp=c(2,.5,0), cex=1)
for(pd in patients){
    e <- new.env()
    i <- which(rownames(dataFrame)==pd)
    load(paste0("../code/loo/",i,".RData"), env=e)

    cvIdx <- 1:nrow(dataFrame)
    whichTrain <<- which(cvIdx != i)
    xx <- 0:2000
    coxphPrs <- coxph(Surv(time1, time2, status)~ pspline(time0, df=10), data=data.frame(prdData, time0=as.numeri
c(clinicalData$Recurrence_date-clinicalData$CR_date)[prdData$index])[prdData$index %in% whichTrain,])
    tdPrmBaseline <- exp(predict(coxphPrs, newdata=data.frame(time0=xx[-1])))

    coxphOs <- coxph(Surv(time1, time2, status)~ pspline(time0, df=10), data=data.frame(osData, time0=pmin(500,cr
[osData$index,1]))[osData$index %in% whichTrain,])
    tdOsBaseline <- exp(pmin(predict(coxphOs, newdata=data.frame(time0=500)),predict(coxphOs, newdata=data.frame(
time0=xx[-1])))) ## cap predictions at induction length 500 days.
    newdata <- allDataTpl[grep(pd, rownames(allDataTpl)),]
    m <- MultiRFX5(e$rfxEs, e$rfxCr, e$rfxNrs, e$rfxRel, e$rfxPrs, newdata, tdPrmBaseline = tdPrmBaseline, tdOsBa
seline = tdOsBaseline, x=2000)

    w <- seq(1,2001,10)
    at <- ceiling(1:5 * 365.5)
    x <- (w-1)/365.25
    plot.new()
    p <- which(rownames(dataFrame)==pd)
    mtext(side=2, paste0(c(pd, clinicalData$AOD[p], c("male","female")[clinicalData$gender[p]], gsub(";","\n",gen
otype[p]), paste("ELN", clinicalData$M_Risk[p])), collapse="\n"), las=1, adj=0)

    for(i in c(3,2)){
        sedimentPlot(-m[w,6:8,i],x=x, y0=1, y1=0,  col=pastel1[c(2:3,5,4)], xlab="Years from CR",ylab="Probabilit
y", xaxs='i', yaxs='i')
        o <- 1-rowSums(m[w,6:7,i])
        abline(v=c(1:5), col="white", lty=3)
        abline(h=seq(0.2,0.8,0.2), col="white", lty=3)
        lines(x,o, lwd=2)
        lines(x,o ^ exp(qnorm(0.975) * m[w,9,i]))
        lines(x,o ^ exp(-qnorm(0.975) * m[w,9,i]))
        text(x=rep(0,3), c(0.1,0.2,0.3), c("AAR", "RD", "NRD") )
        text(x=1:5, y=rep(0.3, 5), round(m[at,6,i],2))
        text(x=1:5, y=rep(0.2, 5), round(m[at,7,i],2))
        text(x=1:5, y=rep(0.1, 5), round(m[at,8,i],2))
        #text(x=at, y=rep(0.1, 5), round(fiveStagePredictedTpl[w,6,i],2))
        lineStage(CR_date=0, as.numeric(clinicalData$Recurrence_date[p]-clinicalData$CR_date[p])/365.25, as.numer
ic(clinicalData$Date_LF[p]-clinicalData$CR_date[p])/365.25, ERDate=0, clinicalData$Status[p], col=c(brewer.pal(8,
"Dark2")[8], set1[c(4:5,1:3)]), lwd=4, pch.trans=NA, y=0.05, cex=4)
        points(x=as.numeric(clinicalData$TPL_date[p]-clinicalData$CR_date[p])/365.25, y=0.05, pch=4, cex=1.5, lwd
=1)

    }}
```

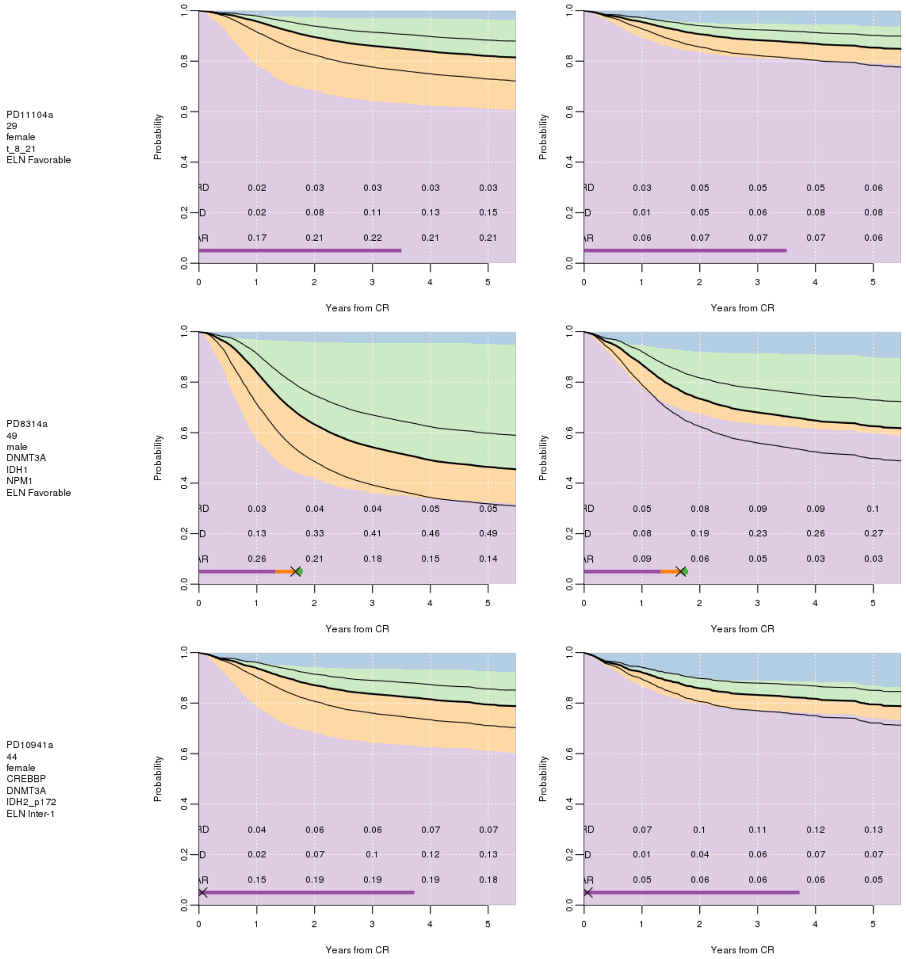

3.6.5.9 Supplementary Figure S2

Here we show results for an additional 4 patients, the first three being chosen as counterparts to the patients shown in Figure 4. The fourth patient is a patients with ASXL1 mutations.

```
xmax=2000
patients <- c("PD10828a","PD10844a","PD10829a","PD10996a")
layout(matrix(1:(3*length(patients)), byrow=TRUE, ncol=3), width=c(0.5,1,1))
par(mar=c(3,3,1,1), bty="n", mgp=c(2,.5,0), cex=1)
for(pd in patients){
    e <- new.env()
    i <- which(rownames(dataFrame)==pd)
    load(paste0("../code/loo/",i,".RData"), env=e)

    cvIdx <- 1:nrow(dataFrame)
    whichTrain <<- which(cvIdx != i)
    xx <- 0:2000
    coxphPrs <- coxph(Surv(time1, time2, status)~ pspline(time0, df=10), data=data.frame(prdData, time0=as.numeri
c(clinicalData$Recurrence_date-clinicalData$CR_date)[prdData$index])[prdData$index %in% whichTrain,])
    tdPrmBaseline <- exp(predict(coxphPrs, newdata=data.frame(time0=xx[-1])))

    coxphOs <- coxph(Surv(time1, time2, status)~ pspline(time0, df=10), data=data.frame(osData, time0=pmin(500,cr
[osData$index,1]))[osData$index %in% whichTrain,])
    tdOsBaseline <- exp(pmin(predict(coxphOs, newdata=data.frame(time0=500)),predict(coxphOs, newdata=data.frame(
time0=xx[-1])))) ## cap predictions at induction length 500 days.
    newdata <- allDataTpl[grep(pd, rownames(allDataTpl)),]
    m <- MultiRFX5(e$rfxEs, e$rfxCr, e$rfxNrs, e$rfxRel, e$rfxPrs, newdata, tdPrmBaseline = tdPrmBaseline, tdOsBa
seline = tdOsBaseline, x=2000)

    w <- seq(1,2001,10)
    at <- ceiling(1:5 * 365.5)
    x <- (w-1)/365.25
    plot.new()
    p <- which(rownames(dataFrame)==pd)
    mtext(side=2, paste0(c(pd, clinicalData$AOD[p], c("male","female")[clinicalData$gender[p]], gsub(";","\n",gen
otype[p]), paste("ELN", clinicalData$M_Risk[p])), collapse="\n"), las=1, adj=0)

    for(i in c(3,2)){
        sedimentPlot(-m[w,6:8,i],x=x, y0=1, y1=0,  col=pastel1[c(2:3,5,4)], xlab="Years from CR",ylab="Probabilit
y", xaxs='i', yaxs='i')
        o <- 1-rowSums(m[w,6:7,i])
        abline(v=c(1:5), col="white", lty=3)
        abline(h=seq(0.2,0.8,0.2), col="white", lty=3)
        lines(x,o, lwd=2)
        lines(x,o ^ exp(qnorm(0.975) * m[w,9,i]))
        lines(x,o ^ exp(-qnorm(0.975) * m[w,9,i]))
        text(x=rep(0,3), c(0.1,0.2,0.3), c("AAR", "RD", "NRD") )
        text(x=1:5, y=rep(0.3, 5), round(m[at,6,i],2))
        text(x=1:5, y=rep(0.2, 5), round(m[at,7,i],2))
        text(x=1:5, y=rep(0.1, 5), round(m[at,8,i],2))
        #text(x=at, y=rep(0.1, 5), round(fiveStagePredictedTpl[w,6,i],2))
        lineStage(CR_date=0, as.numeric(clinicalData$Recurrence_date[p]-clinicalData$CR_date[p])/365.25, as.numer
ic(clinicalData$Date_LF[p]-clinicalData$CR_date[p])/365.25, ERDate=0, clinicalData$Status[p], col=c(brewer.pal(8,
"Dark2")[8], set1[c(4:5,1:3)]), lwd=4, pch.trans=NA, y=0.05, cex=4)
        points(x=as.numeric(clinicalData$TPL_date[p]-clinicalData$CR_date[p])/365.25, y=0.05, pch=4, cex=1.5, lwd
=1)

    }}
```

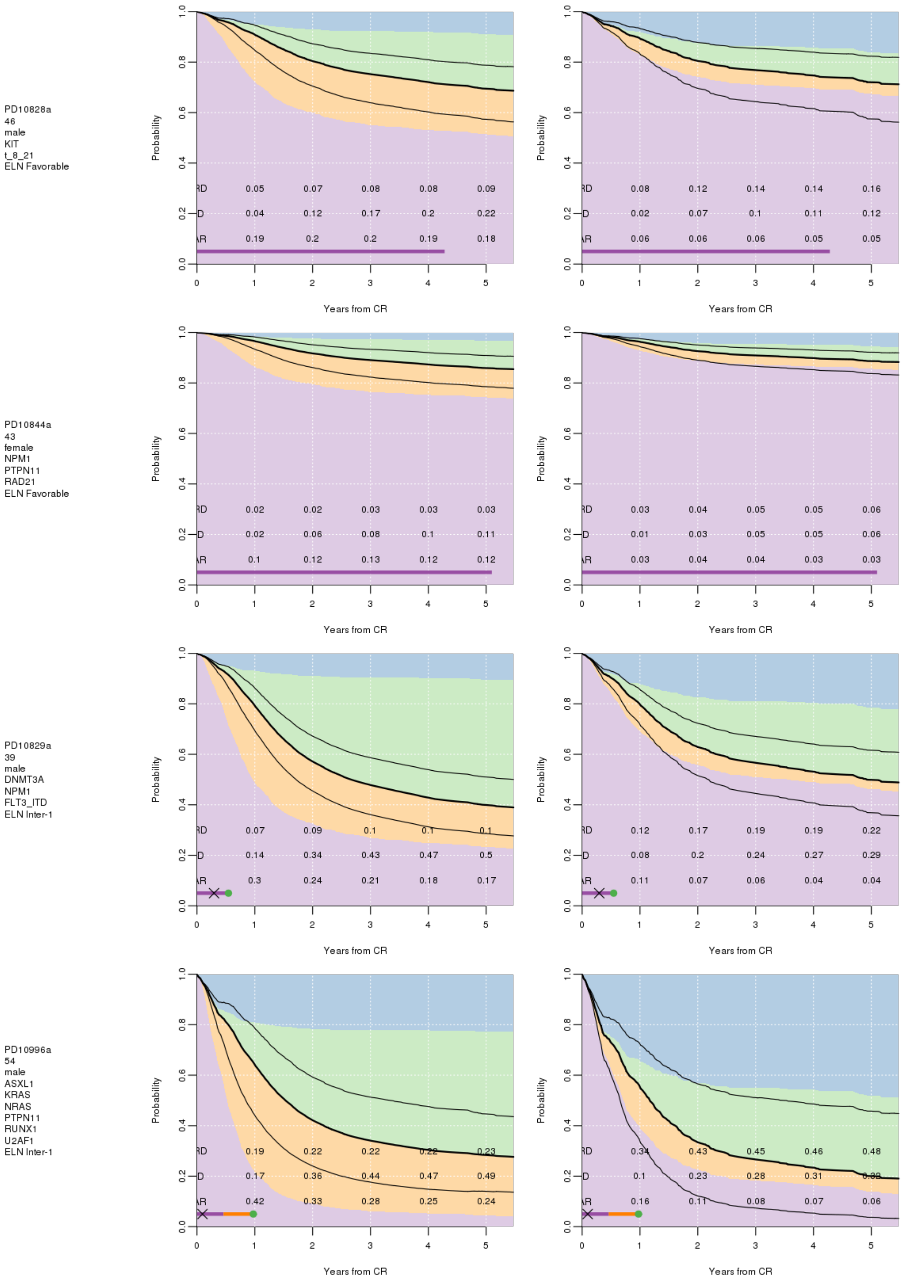

3.6.6 Predicting outcome after CR

We use the following function to compute the hierarchical adjustment for two subsequent stages.

```
cppFunction('NumericVector computeTotalPrsC(NumericVector x, NumericVector diffCir, NumericVector prsP, NumericVe
ctor tdPrmBaseline, double risk) {
                int xLen = x.size();
                double hj;
                double r = exp(risk);
                NumericVector rs(xLen);
                for(int i = 0; i < xLen; ++i) rs[i] = 1;
                for(int j = 1; j < xLen; ++j){
                hj = tdPrmBaseline[j-1] * r;
                for(int i = j; i < xLen; ++i){
                rs[i] += diffCir[j-1] * (1-pow(prsP[i-j], hj));
                }
                }
                return rs;
                }', rebuild=TRUE)
```

Function to predict OS from Relapse, PRS and NRM, as described in Section 4.3.5. It is slightly more efficient than MultiRFX5, as it doesn't require evaluating the chances of reaching CR.

```
MultiRFX3 <- function(coxRFXNrdTD, coxRFXRelTD, coxRFXPrdTD, data, x =365, ciType="analytical", prdData){
    ## Step 1: Compute KM survival curves and log hazard
    getS <- function(coxRFX, data, max.x=5000) {
        if(!is.null(coxRFX$na.action)) coxRFX$Z <- coxRFX$Z[-coxRFX$na.action,]
        data <- as.matrix(data[,match(colnames(coxRFX$Z),colnames(data))])
        r <- PredictRiskMissing(coxRFX, data, var="var2")
        H0 <- basehaz(coxRFX, centered = FALSE)
        hazardDist <- splinefun(H0$time, H0$hazard, method="monoH.FC")
```

```
        x <- c(0:max.x,max.x)
        S <- exp(-hazardDist(x))
        return(list(S=S, r=r, x=x, hazardDist=hazardDist, r0 = coxRFX$means %*% coef(coxRFX)))
    }
    kmRel <- getS(coxRFX = coxRFXRelTD, data = data, max.x=max(x))
    kmNrd <- getS(coxRFX = coxRFXNrdTD, data = data, max.x=max(x))
    kmPrd <- getS(coxRFX = coxRFXPrdTD, data = data, max.x=max(x))
```

KmRel, kmNrd, kmPrd were previously represented by ***kmCir, kmNrm***, and ***kmPrs***

```
    ## Step 2: Adjust CIR and NRM curve for competing risks, accounting for hazard
    kmRel$Sadj <- sapply(1:nrow(data), function(i) cumsum(c(1,diff(kmRel$S^exp(kmRel$r[i,1]))) * kmNrd$S ^ exp(km
Nrd$r[i,1])))
    kmNrd$Sadj <- sapply(1:nrow(data), function(i) cumsum(c(1,diff(kmNrd$S^exp(kmNrd$r[i,1]))) * kmRel$S ^ exp(km
Rel$r[i,1]))) ## array times x nrow(data)

    stopifnot(length(x)==1 | length(x) == nrow(data))
    if(length(x)==nrow(data))
        w <- match(x,kmRel$x)
    else if(length(x)==1)
        w <- rep(match(x, kmRel$x), nrow(data))
    y <- mapply(function(i,j) kmNrd$Sadj[i,j], w,1:length(w) ) # select time for each sample
    nrs <- y
    nrsUp <- y^exp(2*sqrt(kmNrd$r[,2]))
    nrsLo <- y^exp(- 2*sqrt(kmNrd$r[,2]))

    y <- mapply(function(i,j) kmRel$Sadj[i,j], w,1:length(w) ) # select time for each sample
    cir <- y
    cirLo <- y^exp( 2*sqrt(kmRel$r[,2]))
    cirUp <- y^exp( - 2*sqrt(kmRel$r[,2]))
```

CIR seems to be short for ***Cumulative Incidence of Relapse***; here it is in fact ***1-Cumulative Incidence of Relapse***

```
    ## Step 3: Compute post-relapse survival
    survPredict <- function(surv){
        s <- survfit(surv~1)
        splinefun(s$time, s$surv, method="monoH.FC")
    }
    xx <- 0:max(x)
    # Baseline Prs (measured from relapse)
    kmPrs0 <- survPredict(Surv(prdData$time1, prdData$time2, prdData$status))(xx)
    # PRS baseline with spline-based dep on CR length)

    coxphPrs <- coxph(Surv(time1, time2, status)~ pspline(time0, df=10), data=prdData )
    tdPrmBaseline <- exp(predict(coxphPrs, newdata=data.frame(time0=xx[-1])))
    rs <- sapply(1:nrow(data), function(i){
                ### Different approach
                xLen <- 1+floor(x)
                cir <- kmRel$Sadj[1:xLen,i]
                rs <- computeTotalPrsC(x = xx, diffCir = diff(cir), prsP = kmPrs0, tdPrmBaseline = tdPrmBaseline,
risk = kmPrd$r[i,1]-kmPrd$r0)
                rs[xLen]
            })

    ## Step 4: Combine into overall survival
    if(any(1-(1-rs)-(1-nrs)<0)) warning("OS < 0 occured.")
    os <- pmax(pmin(1-(1-rs)-(1-nrs),1),0)

    ## Step 5: Confidence intervals for OS
    osCi <- sapply(1:nrow(data), function(i){
                if("analytical" == ciType){
                    ## Confidence intervals
                    PlogP2 <- function(x) {(x * log(x))^2}
                    errOs <- kmNrd$r[i,2] * PlogP2(kmNrd$S[w[i]]) * (1-kmRel$S[w[i]] * kmPrd$S[w[i]])^2 + kmRel$r
[i,2]  * (1-kmNrd$S[w[i]])^2* kmPrd$S[w[i]]^2 * PlogP2(kmRel$S[w[i]]) +  kmPrd$r[i,2]  * (1-kmNrd$S[w[i]])^2* kmR
el$S[w[i]]^2 * PlogP2(kmPrd$S[w[i]])
                    errOs <- errOs / PlogP2(1-(1-kmNrd$S[w[i]])*(1-kmRel$S[w[i]]*kmPrd$S[w[i]]))
                    return(c(osUp=os[i] ^ exp(-2* errOs), osLo= os[i] ^ exp(+2*errOs)))
                } else if("simulated" == ciType){
                    ## Simulate CI
                    nSim <- 200
                    osCiMc <- sapply(1:nSim, function(foo){
                                H <- exp(rnorm(3,c(kmRel$r[i,1],kmNrd$r[i,1],kmPrd$r[i,1]),sqrt(c(kmRel$r[i,2],km
Nrd$r[i,2],kmPrd$r[i,2]))))
                                nrs <- cumsum(c(1,diff(kmNrd$S^H[2]) * kmRel$S[-1]^H[1])) ## Correct KM estimate
for competing risk
                                diffCir <- diff(kmRel$inc^H[1]) * kmNrd$inc[-1]^H[2] ## Correct KM estimate for c
ompeting risk
                                rs <- computeTotalPrsC(x = x, diffCir = diffCir, prsP = kmPrs0, tdPrmBaseline = t
dPrmBaseline, risk = -kmPrd$r0+log(H[3]))
                                return((1-(1-nrs)-(1-rs))[w[i]])
                            })
                    osCiMcQ <- quantile(osCiMc, c(0.025,0.975))
                    return(c(osUp = osCiMcQ[2], osLo = osCiMcQ[1]))
                }
            })

    return(data.frame(os=os, osLo = osCi[2,], osUp = osCi[1,],  cir=cir, cirLo=cirLo, cirUp=cirUp, nrs=nrs, nrsLo
=nrsLo, nrsUp=nrsUp, rs=rs ))
}
```

Create a data.frame with all data in cr

```
allData <- MakeTimeDependent(dataFrame[whichRFXRel], timeEvent=alloTimeCR1, timeStop=as.numeric(clinicalData$Date
_LF- clinicalData$CR_date), status=clinicalData$Status)
allData$transplantCR1 <- allData$event
allData$transplantRel <- allData$event
w <- which(clinicalData$TPL_date > clinicalData$Recurrence_date)
allData$transplantCR1[allData$index %in% w] <- 0
allData$transplantRel[!allData$index %in% w] <- 0

multiRFX3 <-  MultiRFX3(coxRFXNrdTD = coxRFXNrdTD, coxRFXPrdTD = coxRFXPrdTD, coxRFXRelTD = coxRFXRelTD, data=all
Data, x=3*365, prdData=prdData)
```

3.6.6.1 Model assessment

3.6.6.1.1 Random cross-validation

```
replicates <- 100 ## number of replicates
concordanceCIRcv <- lapply(list(crGroups[crGroups %in% mainGroups], crGroups), function(g){
            mclapply(1:replicates, function(foo){
                        set.seed(foo)
                        trainIdx <- sample(1:nrow(dataFrame)%%5 +1 )!=1 ## sample 4/5
                        dNrm <- nrdData[nrdData$index %in% which(trainIdx),names(g)]
                        sNrm <- Surv(nrdData$time1, nrdData$time2, nrdData$status)[nrdData$index %in% which(train
Idx)]
                        coxRFXNrdTD <- CoxRFX(dNrm, sNrm, groups=g, nu=1, which.mu = mainGroups)
                        coxRFXNrdTD$coefficients["transplantRel"] <- 0
                        dPrs <- prdData[prdData$index %in% which(trainIdx), c(names(g),"time0","time1","time2","s
tatus")]
                        sPrs <- Surv(prdData$time1, prdData$time2, prdData$status)[prdData$index %in% which(train
Idx)]
                        coxRFXPrdTD <-  CoxRFX(dPrs, sPrs, groups=g, nu=1, which.mu = mainGroups)
                        dCir <- relData[relData$index %in% which(trainIdx), names(g)]
                        sCir <- Surv(relData$time1, relData$time2, relData$status)[relData$index %in% which(train
Idx)]
                        coxRFXRelTD <-  CoxRFX(dCir, sCir, groups=g, which.mu = mainGroups)
                        coxRFXRelTD$coefficients["transplantRel"] <- 0
                        dOs <- osData[osData$index %in% which(trainIdx), names(g)]
                        sOs <- Surv(osData$time1, osData$time2, osData$status)[osData$index %in% which(trainIdx)]
                        coxRFXOsCR <- CoxRFX(dOs, sOs, groups=g, which.mu = mainGroups)

                        allRisk365 <- MultiRFX3(coxRFXNrdTD = coxRFXNrdTD, coxRFXPrdTD = coxRFXPrdTD, coxRFXRelTD
= coxRFXRelTD, data=allData, x=365, prdData=dPrs)
                        allRisk1000 <- MultiRFX3(coxRFXNrdTD = coxRFXNrdTD, coxRFXPrdTD = coxRFXPrdTD, coxRFXRelT
D = coxRFXRelTD, data=allData, x=1000, prdData=dPrs)

                        p365 <- -allRisk365[,1]
                        p1000 <-  -allRisk1000[,1]
                        pCIR <- as.matrix(relData[names(g)]) %*% coef(coxRFXRelTD)
                        pPRS <- as.matrix(prdData[names(g)]) %*% coef(coxRFXPrdTD)
                        pNRM <- as.matrix(nrdData[names(g)]) %*% coef(coxRFXNrdTD)
                        pOS <- as.matrix(osData[names(g)]) %*% coef(coxRFXOsCR)

                        C <- c(
                                CIRrfx = survConcordance(Surv(time1, time2, status)~ pCIR, data=relData, subset =
relData$index %in% which(!trainIdx) )$concordance,
                                PRSrfx = survConcordance(Surv(time1, time2, status) ~ pPRS, data=prdData, subset=
prdData$index %in% which(!trainIdx) )$concordance,
                                NRMrfx = survConcordance(Surv(time1, time2, status)~  pNRM, data=nrdData, subset=
nrdData$index %in% which(!trainIdx) )$concordance,
                                OSrfx = survConcordance(Surv(time1, time2, status) ~ pOS, data=osData, subset=osD
ata$index %in% which(!trainIdx) )$concordance,
                                OS365 = survConcordance(Surv(time1, time2, status) ~ p365, data=osData, subset=os
Data$index %in% which(!trainIdx) )$concordance,
                                OS1000 = survConcordance(Surv(time1,time2, status) ~ p1000, data=osData, subset=o
sData$index %in% which(!trainIdx) )$concordance
                        )

                        coef <- cbind(CIRrfx=coef(coxRFXRelTD), PRSrfx=coef(coxRFXPrdTD), NRMrfx=coef(coxRFXNrdTD
),  OSrfx=coef(coxRFXOsCR))

                        return(list(C=C, coef=coef, allRisk365=allRisk365, allRisk1000=allRisk1000))
                    }, mc.cores=10)
        })

apply(apply(-sapply(concordanceCIRcv[[1]], `[[` , "C")[4:6,],2,rank),1,function(x) table(factor(x, levels=1:3)))
```

```
##   OSrfx.concordant OS365.concordant OS1000.concordant
## 1               70                9                20
## 2                9               25                65
## 3               21               65                14
```

```
> apply(apply(-sapply(concordanceCIRcv[[1]], `[[` , "C")[4:6,],2,rank),1,
function(x) table(factor(x, levels=1:3)))
  OSrfx.concordant OS365.concordant OS1000.concordant
1               64               19                17
2               12               41                47
3               24               40                36
```

[outputs of the rerun (2021)]

```
apply(apply(-sapply(concordanceCIRcv[[2]], `[[` , "C")[4:6,],2,rank),1,function(x) table(factor(x, levels=1:3)))
```

```
##   OSrfx.concordant OS365.concordant OS1000.concordant
## 1               39                7                53
## 2               23               31                45
## 3               37               61                 2
```

```
> apply(apply(-sapply(concordanceCIRcv[[2]], `[[` , "C")[4:6,],2,rank),1,
function(x) table(factor(x, levels=1:3)))
  OSrfx.concordant OS365.concordant OS1000.concordant
1               39               24                36
2                9               33                56
3               51               42                 6
```

[outputs of the rerun (2021)]

Test and train errors

```
i <- 0
concordanceCIRcvTrain <- lapply(list(crGroups[crGroups %in% mainGroups], crGroups), function(g){
            i <- i+1
            sapply(1:replicates, function(foo){
                        set.seed(foo)
                        trainIdx <- sample(1:nrow(dataFrame)%%5 +1 )!=1 ## sample 4/5
                        coef <- concordanceCIRcv[[i]][[foo]][["coef"]]
                        pCIR <- as.matrix(relData[names(coef[,"CIRrfx"])]) %*% coef[,"CIRrfx"]
                        pPRS <- as.matrix(prdData[names(coef[,"PRSrfx"])]) %*% coef[,"PRSrfx"]
                        pNRM <- as.matrix(nrdData[names(coef[,"NRMrfx"])]) %*% coef[,"NRMrfx"]
                        pOS <- as.matrix(osData[names(coef[,"OSrfx"])]) %*% coef[,"OSrfx"]
                        p365 <- -concordanceCIRcv[[i]][[foo]][["allRisk365"]]$os
                        p1000 <- -concordanceCIRcv[[i]][[foo]][["allRisk1000"]]$os
                        C <- sapply(list(train=which(trainIdx), test=which(!trainIdx)), function(w)
                                    c(
                                            CIRrfx = survConcordance(Surv(time1, time2, status)~ pCIR, data=relDa
ta, subset = relData$index %in% w )$concordance,
                                            PRSrfx = survConcordance(Surv(time1, time2, status) ~ pPRS, data=prdD
ata, subset=prdData$index %in% w )$concordance,
                                            NRMrfx = survConcordance(Surv(time1, time2, status)~  pNRM, data=nrdD
ata, subset=nrdData$index %in% w )$concordance,
                                            OSrfx = survConcordance(Surv(time1, time2, status) ~ pOS, data=osData
, subset=osData$index %in% w )$concordance,
                                            OS365 = survConcordance(Surv(time1, time2, status) ~ p365, data=osDat
a, subset=osData$index %in% w )$concordance,
                                            OS1000 = survConcordance(Surv(time1,time2, status) ~ p1000, data=osDa
ta, subset=osData$index %in% w )$concordance
                                    ))
                        return(C)
                    }, simplify='array')
        })
```

Plot test and training errors

```
for(i in 1:4){
    plot(t(concordanceCIRcvTrain[[2]][i,,] ), main=rownames(concordanceCIRcvTrain[[2]])[i])
    abline(0,1)
}
```

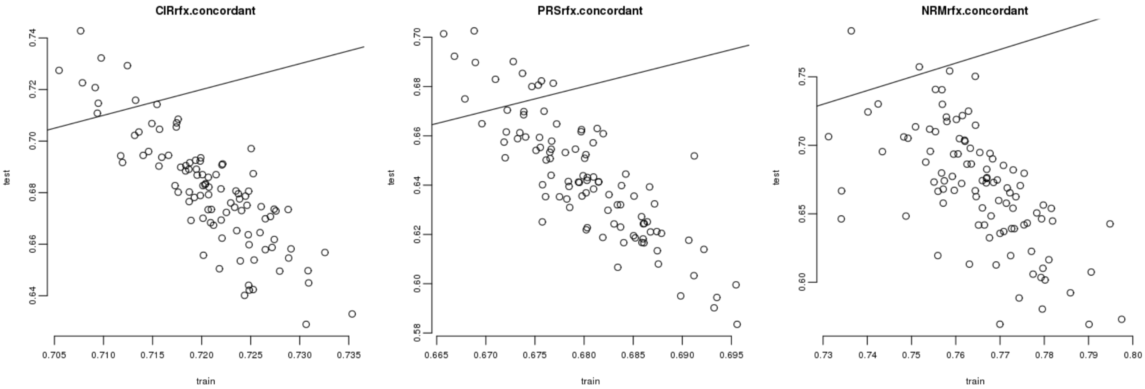

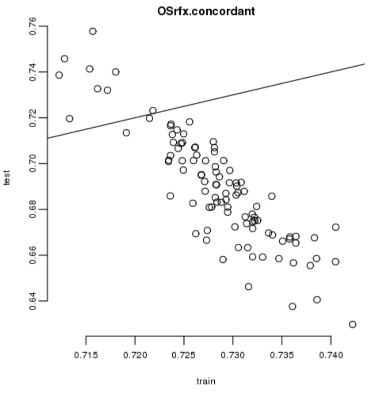

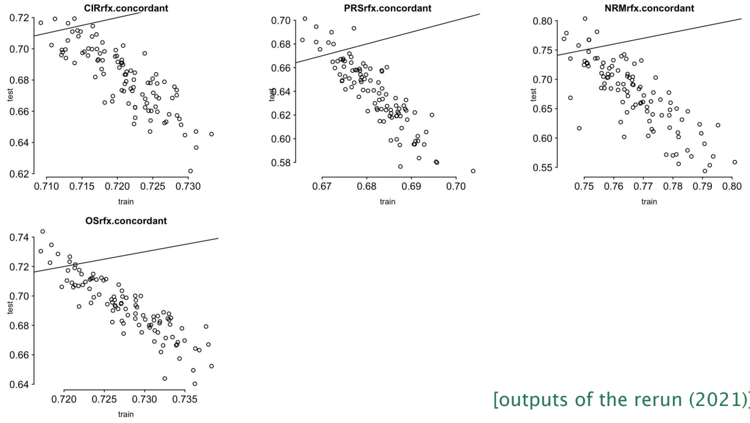

[outputs of the rerun (2021)]

Plot coefficients v mean of subsampled coef

```
r <- rowMeans(sapply(concordanceCIRcv[[2]], `[[` , "coef", simplify="array"), dim=2)
plot(r[,1], coef(coxRFXRelTD)); abline(0,1)
```

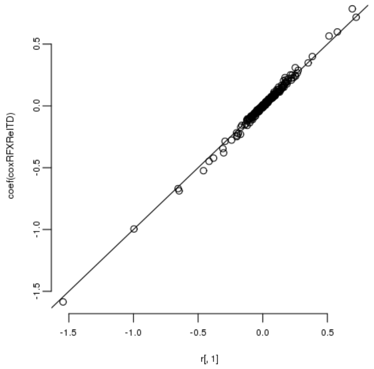


```
plot(r[,2], coef(coxRFXPrdTD)); abline(0,1)
```

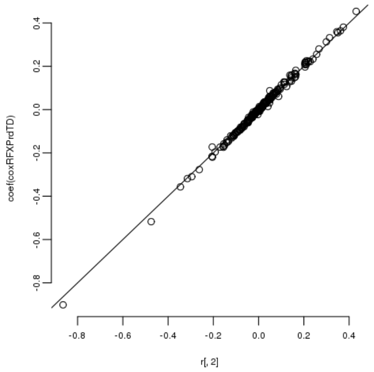


```
plot(r[,3], coef(coxRFXNrdTD)); abline(0,1)
```

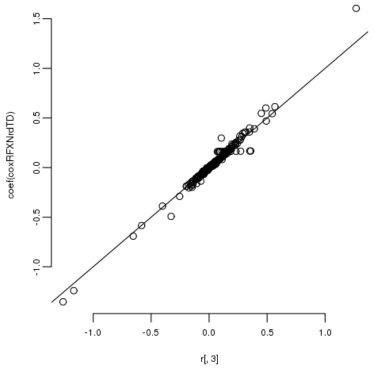


```
plot(r[,4], coef(coxRFXOsCR)); abline(0,1)
```

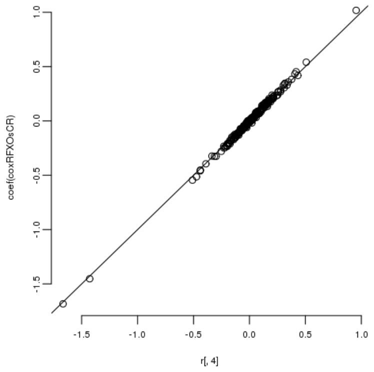

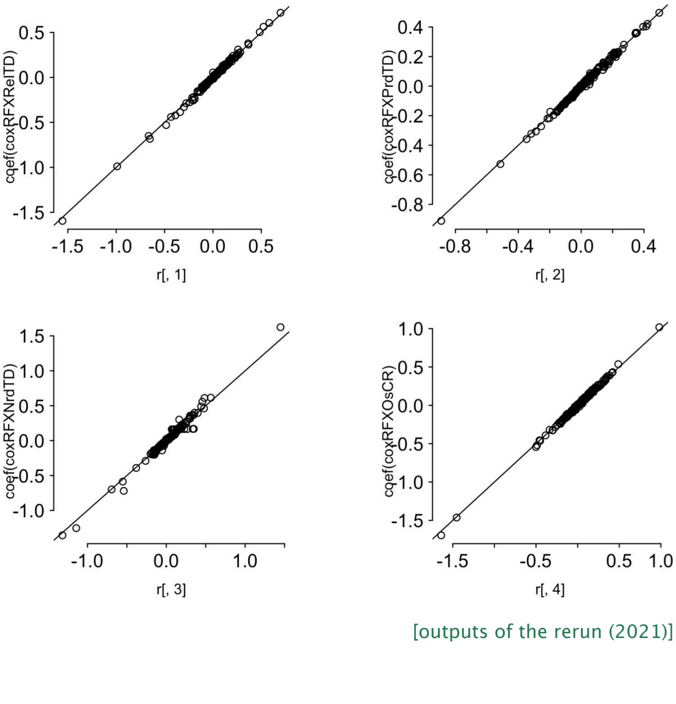

[outputs of the rerun (2021)]

Variance-based concordance estimate

```
i <- 0
concordanceCIRcvVar <- lapply(list(crGroups[crGroups %in% mainGroups], crGroups), function(g){
            i <- i+1
            sapply(1:replicates, function(foo){
                        set.seed(foo)
                        trainIdx <- sample(1:nrow(dataFrame)%%5 +1 )!=1 ## sample 4/5
                        coef <- concordanceCIRcv[[i]][[foo]][["coef"]]
                        pCIR <- as.matrix(relData[names(coef[,"CIRrfx"])]) %*% coef[,"CIRrfx"]
                        pPRS <- as.matrix(prdData[names(coef[,"PRSrfx"])]) %*% coef[,"PRSrfx"]
                        pNRM <- as.matrix(nrdData[names(coef[,"NRMrfx"])]) %*% coef[,"NRMrfx"]
                        pOS <- as.matrix(osData[names(coef[,"OSrfx"])]) %*% coef[,"OSrfx"]
                        C <- sapply(list(train=which(trainIdx), test=which(!trainIdx)), function(w){
                                    sapply(ls(sys.frame(-3),pattern='^p[A-Z]+'), function(x)
                                                CoxHD::ConcordanceFromVariance(var(get(x)[w], na.rm=TRUE)))[c(1,
4,2,3)]
                                })
                    }, simplify="array")})

for(i in 1:4)
{cat(rownames(concordanceCIRcvTrain[[2]])[i],"\n"); print(summary(data.frame(harrel=t(concordanceCIRcvTrain[[2]][
i,1:2,]) , var=t(concordanceCIRcvVar[[2]][i,1:2,])))))}
```

```
## CIRrfx.concordant
##   harrel.train      harrel.test       var.train         var.test
##  Min.   :0.7055   Min.   :0.6289   Min.   :0.6609   Min.   :0.6546
##  1st Qu.:0.7176   1st Qu.:0.6681   1st Qu.:0.6711   1st Qu.:0.6691
##  Median :0.7207   Median :0.6802   Median :0.6760   Median :0.6759
##  Mean   :0.7208   Mean   :0.6807   Mean   :0.6759   Mean   :0.6755
##  3rd Qu.:0.7248   3rd Qu.:0.6923   3rd Qu.:0.6800   3rd Qu.:0.6813
##  Max.   :0.7353   Max.   :0.7428   Max.   :0.6972   Max.   :0.6937
## PRSrfx.concordant
##   harrel.train      harrel.test       var.train         var.test
##  Min.   :0.6744   Min.   :0.5938   Min.   :0.6491   Min.   :0.6344
##  1st Qu.:0.6828   1st Qu.:0.6335   1st Qu.:0.6577   1st Qu.:0.6525
##  Median :0.6870   Median :0.6528   Median :0.6603   Median :0.6587
##  Mean   :0.6873   Mean   :0.6516   Mean   :0.6612   Mean   :0.6596
##  3rd Qu.:0.6913   3rd Qu.:0.6679   3rd Qu.:0.6645   3rd Qu.:0.6656
##  Max.   :0.7023   Max.   :0.7141   Max.   :0.6772   Max.   :0.6878
## NRMrfx.concordant
##   harrel.train      harrel.test       var.train         var.test
##  Min.   :0.7312   Min.   :0.5693   Min.   :0.6617   Min.   :0.6601
##  1st Qu.:0.7573   1st Qu.:0.6424   1st Qu.:0.6840   1st Qu.:0.6835
##  Median :0.7657   Median :0.6714   Median :0.6932   Median :0.6902
##  Mean   :0.7652   Mean   :0.6691   Mean   :0.6920   Mean   :0.6912
##  3rd Qu.:0.7729   3rd Qu.:0.6962   3rd Qu.:0.6985   3rd Qu.:0.7000
##  Max.   :0.7975   Max.   :0.7836   Max.   :0.7194   Max.   :0.7231
## OSrfx.concordant
##   harrel.train      harrel.test       var.train         var.test
##  Min.   :0.7123   Min.   :0.6298   Min.   :0.6804   Min.   :0.6805
##  1st Qu.:0.7250   1st Qu.:0.6714   1st Qu.:0.6967   1st Qu.:0.6941
##  Median :0.7283   Median :0.6876   Median :0.7000   Median :0.7000
##  Mean   :0.7284   Mean   :0.6892   Mean   :0.7000   Mean   :0.6999
##  3rd Qu.:0.7320   3rd Qu.:0.7055   3rd Qu.:0.7038   3rd Qu.:0.7058
##  Max.   :0.7422   Max.   :0.7577   Max.   :0.7175   Max.   :0.7232
```

3.6.6.1.2 Cross-validation across trials

```
> for(i in 1:4)
+ {cat(rownames(concordanceCIRcvTrain[[2]])[i],"\n"); print(summary(data.frame(harrel=t(concordanceCIRcvTrain[[2]][i,1:2,]) , var=t(concordance
CIRcvVar[[2]][i,1:2,])))))}
CIRrfx.concordant
  harrel.train     harrel.test     var.train.Length  var.train.Class  var.train.Mode var.test.Length  var.test.Class  var.test.Mode
 Min.   :0.7092   Min.   :0.6217   0       -none-   NULL                           0       -none-   NULL
 1st Qu.:0.7171   1st Qu.:0.6661   0       -none-   NULL                           0       -none-   NULL
 Median :0.7211   Median :0.6827   0       -none-   NULL                           0       -none-   NULL
 Mean   :0.7209   Mean   :0.6812   0       -none-   NULL                           0       -none-   NULL
 3rd Qu.:0.7247   3rd Qu.:0.6971   0       -none-   NULL                           0       -none-   NULL
 Max.   :0.7333   Max.   :0.7193   0       -none-   NULL                           0       -none-   NULL
PRSrfx.concordant
  harrel.train     harrel.test     var.train.Length  var.train.Class  var.train.Mode var.test.Length  var.test.Class  var.test.Mode
 Min.   :0.6656   Min.   :0.5727   0       -none-   NULL                           0       -none-   NULL
 1st Qu.:0.6766   1st Qu.:0.6211   0       -none-   NULL                           0       -none-   NULL
 Median :0.6823   Median :0.6406   0       -none-   NULL                           0       -none-   NULL
 Mean   :0.6818   Mean   :0.6382   0       -none-   NULL                           0       -none-   NULL
 3rd Qu.:0.6868   3rd Qu.:0.6583   0       -none-   NULL                           0       -none-   NULL
 Max.   :0.7038   Max.   :0.7014   0       -none-   NULL                           0       -none-   NULL
NRMrfx.concordant
  harrel.train     harrel.test     var.train.Length  var.train.Class  var.train.Mode var.test.Length  var.test.Class  var.test.Mode
 Min.   :0.7432   Min.   :0.5437   0       -none-   NULL                           0       -none-   NULL
 1st Qu.:0.7576   1st Qu.:0.6368   0       -none-   NULL                           0       -none-   NULL
 Median :0.7658   Median :0.6823   0       -none-   NULL                           0       -none-   NULL
 Mean   :0.7671   Mean   :0.6737   0       -none-   NULL                           0       -none-   NULL
 3rd Qu.:0.7759   3rd Qu.:0.7193   0       -none-   NULL                           0       -none-   NULL
 Max.   :0.8009   Max.   :0.8034   0       -none-   NULL                           0       -none-   NULL
OSrfx.concordant
  harrel.train     harrel.test     var.train.Length  var.train.Class  var.train.Mode var.test.Length  var.test.Class  var.test.Mode
 Min.   :0.7171   Min.   :0.6403   0       -none-   NULL                           0       -none-   NULL
 1st Qu.:0.7235   1st Qu.:0.6802   0       -none-   NULL                           0       -none-   NULL
 Median :0.7274   Median :0.6926   0       -none-   NULL                           0       -none-   NULL
 Mean   :0.7277   Mean   :0.6921   0       -none-   NULL                           0       -none-   NULL
 3rd Qu.:0.7319   3rd Qu.:0.7074   0       -none-   NULL                           0       -none-   NULL
 Max.   :0.7383   Max.   :0.7438   0       -none-   NULL                           0       -none-   NULL
```

[outputs of the rerun (2021)]

```
concordanceCIRcvTrial <- mclapply(list(crGroups[crGroups %in% mainGroups], crGroups), function(g){
            mclapply(levels(clinicalData$Study), function(study){
                        trainIdx <- clinicalData$Study != study
                        g <- g[colSums(allData[allData$index %in% which(trainIdx), names(g)])>0]
                        if(study == "AMLSG0704") g <- g[names(g) != "AMLHD98B"] # avoid collinearity
                        dNrm <- nrdData[nrdData$index %in% which(trainIdx),names(g)]
                        sNrm <- Surv(nrdData$time1, nrdData$time2, nrdData$status)[nrdData$index %in% which(train
Idx)]
                        coxRFXNrdTD <- CoxRFX(dNrm, sNrm, groups=g, nu=1, which.mu = mainGroups)
                        coxRFXNrdTD$coefficients["transplantRel"] <- 0
                        dPrs <- prdData[prdData$index %in% which(trainIdx), c(names(g),"time0","time1","time2","s
tatus")]
                        sPrs <- Surv(prdData$time1, prdData$time2, prdData$status)[prdData$index %in% which(train
Idx)]
                        coxRFXPrdTD <-  CoxRFX(dPrs, sPrs, groups=g, nu=1, which.mu = mainGroups)
                        dCir <- relData[relData$index %in% which(trainIdx), names(g)]
                        sCir <- Surv(relData$time1, relData$time2, relData$status)[relData$index %in% which(train
Idx)]
                        coxRFXRelTD <-  CoxRFX(dCir, sCir, groups=g, which.mu = mainGroups)
                        coxRFXRelTD$coefficients["transplantRel"] <- 0
                        dOs <- osData[osData$index %in% which(trainIdx), names(g)]
                        sOs <- Surv(osData$time1, osData$time2, osData$status)[osData$index %in% which(trainIdx)]
                        coxRFXOsCR <- CoxRFX(dOs, sOs, groups=g, which.mu = mainGroups)

                        allRisk365 <- MultiRFX3(coxRFXNrdTD = coxRFXNrdTD, coxRFXPrdTD = coxRFXPrdTD, coxRFXRelTD
= coxRFXRelTD, data=allData, x=365, prdData=dPrs)
                        allRisk1000 <- MultiRFX3(coxRFXNrdTD = coxRFXNrdTD, coxRFXPrdTD = coxRFXPrdTD, coxRFXRelT
D = coxRFXRelTD, data=allData, x=1000, prdData=dPrs)

                        p365 <- -allRisk365[,1]
                        p1000 <-  -allRisk1000[,1]
                        pCIR <- as.matrix(relData[names(g)]) %*% coef(coxRFXRelTD)
                        pPRS <- as.matrix(prdData[names(g)]) %*% coef(coxRFXPrdTD)
                        pNRM <- as.matrix(nrdData[names(g)]) %*% coef(coxRFXNrdTD)
                        pOS <- as.matrix(osData[names(g)]) %*% coef(coxRFXOsCR)

                        C <- c(
                                CIRrfx = survConcordance(Surv(time1, time2, status)~ pCIR, data=relData, subset =
relData$index %in% which(!trainIdx) )$concordance,
                                PRSrfx = survConcordance(Surv(time2 - time1, status) ~ pPRS, data=prdData, subset
=prdData$index %in% which(!trainIdx) )$concordance,
                                NRMrfx = survConcordance(Surv(time1, time2, status)~  pNRM, data=nrdData, subset=
nrdData$index %in% which(!trainIdx) )$concordance,
                                OSrfx = survConcordance(Surv(time1, time2, status) ~ pOS, data=osData, subset=osD
ata$index %in% which(!trainIdx) )$concordance,
                                OS365 = survConcordance(Surv(time1, time2, status) ~ p365, data=osData, subset=os
Data$index %in% which(!trainIdx) )$concordance,
                                OS1000 = survConcordance(Surv(time1,time2, status) ~ p1000, data=osData, subset=o
sData$index %in% which(!trainIdx) )$concordance
                        )

                        coef <- cbind(RELrfx=coef(coxRFXRelTD), PRSrfx=coef(coxRFXPrdTD), NRSrfx=coef(coxRFXNrdTD
),  OSrfx=coef(coxRFXOsCR))

                        return(list(C=C, coef=coef, allRisk365=allRisk365, allRisk1000=allRisk1000))
                    }, mc.cores=3)
        }, mc.cores=2)
```

```
dotplot(sapply(concordanceCIRcvTrial[[1]], `[[` , "C")[4:6,])
```

Error in FUN(X[[i]], ...) : subscript out of bounds

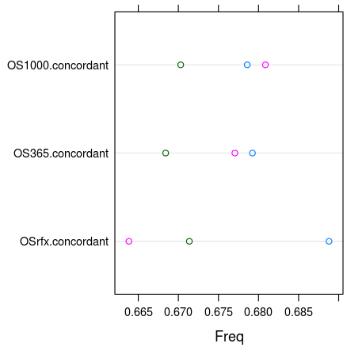


```
dotplot(sapply(concordanceCIRcvTrial[[2]], `[[` , "C")[4:6,])
```

Error in FUN(X[[i]], ...) : subscript out of bounds

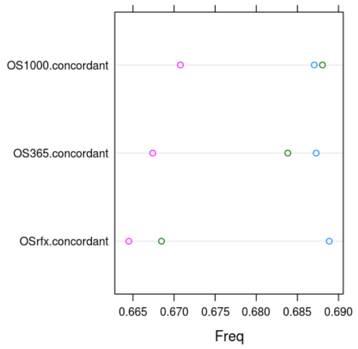

3.6.6.1.3 Test for TPL:Age interactions

```
# CIR
c <- coxph(Surv(time1,time2,status)~transplantCR1*AOD_10, data=relData)
print(c)
```

```
## Call:
## coxph(formula = Surv(time1, time2, status) ~ transplantCR1 *
##     AOD_10, data = relData)
##
##
##                          coef exp(coef) se(coef)     z       p
## transplantCR1         0.514     1.671   0.4349  1.18 2.4e-01
## AOD_10                0.193     1.213   0.0385  5.01 5.4e-07
## transplantCR1:AOD_10 -0.255     0.775   0.0948 -2.69 7.2e-03
##
## Likelihood ratio test=71.2  on 3 df, p=2.33e-15  n= 1609, number of events= 615
##    (271 observations deleted due to missingness)
```

```
anova(c)
```

```
## Analysis of Deviance Table
##  Cox model: response is Surv(time1, time2, status)
## Terms added sequentially (first to last)
##
##                       loglik   Chisq Df Pr(>|Chi|)
## NULL                 -4115.7
## transplantCR1        -4093.3 44.9037  1  2.070e-11 ***
## AOD_10               -4083.6 19.3132  1  1.109e-05 ***
## transplantCR1:AOD_10 -4080.1  7.0165  1   0.008076 **
## ---
## Signif. codes:  0 '***' 0.001 '**' 0.01 '*' 0.05 '.' 0.1 ' ' 1
```

```
#NRM
c <- coxph(Surv(time1,time2,status)~transplantCR1*AOD_10, data=nrdData)
print(c)
```

```
## Call:
## coxph(formula = Surv(time1, time2, status) ~ transplantCR1 *
##     AOD_10, data = nrdData)
##
##
##                          coef exp(coef) se(coef)     z      p
## transplantCR1        0.4899      1.63   0.7724 0.634 0.5300
## AOD_10               0.2847      1.33   0.0885 3.217 0.0013
## transplantCR1:AOD_10 0.0676      1.07   0.1545 0.438 0.6600
##
## Likelihood ratio test=36.7  on 3 df, p=5.36e-08  n= 1609, number of events= 165
##    (271 observations deleted due to missingness)
```

```
anova(c)
```

```
## Analysis of Deviance Table
##  Cox model: response is Surv(time1, time2, status)
## Terms added sequentially (first to last)
##
##                       loglik   Chisq Df Pr(>|Chi|)
## NULL                 -1097.4
## transplantCR1        -1088.7 17.4353  1  2.973e-05 ***
## AOD_10               -1079.1 19.0581  1  1.268e-05 ***
## transplantCR1:AOD_10 -1079.0  0.1927  1     0.6607
## ---
## Signif. codes:  0 '***' 0.001 '**' 0.01 '*' 0.05 '.' 0.1 ' ' 1
```

```
#PRS
c <- coxph(Surv(time1,time2,status)~ transplantRel*AOD_10, data=prdData)
print(c)
```

```
## Call:
## coxph(formula = Surv(time1, time2, status) ~ transplantRel *
##     AOD_10, data = prdData)
##
##
##                          coef exp(coef) se(coef)      z       p
## transplantRel         0.187     1.205   0.4500  0.415 6.8e-01
## AOD_10                0.205     1.228   0.0426  4.814 1.5e-06
## transplantRel:AOD_10 -0.147     0.863   0.0929 -1.585 1.1e-01
##
## Likelihood ratio test=49.1  on 3 df, p=1.23e-10  n= 832, number of events= 479
```

```
anova(c)
```

```
## Analysis of Deviance Table
##  Cox model: response is Surv(time1, time2, status)
## Terms added sequentially (first to last)
##
##                         loglik   Chisq Df Pr(>|Chi|)
## NULL                   -2772.3
## transplantRel          -2760.0 24.6720  1  6.797e-07 ***
## AOD_10                 -2749.0 21.9728  1  2.765e-06 ***
## transplantRel:AOD_10 -2747.8  2.4687  1     0.1161
## ---
## Signif. codes:  0 '***' 0.001 '**' 0.01 '*' 0.05 '.' 0.1 ' ' 1
```

3.6.6.2 Absolute survival probabilities

This function computes the average accuracy of multiple absolute survival predictions at a given point in time by subdividing them into equally sized bins and computing the weighted average absolute difference of the KM estimated survival probability and predicted.

```
EvalAbsolutePred <- function(prediction, surv, time, bins=seq(0,1,0.05)){
    c <- cut(prediction, bins)
    f <- survfit(surv ~ c)
    e <- summary(f, time)
    x <- sapply(strsplit(gsub("[a-z\\=\\(]|]","",e$strata),","), function(x) mean(as.numeric(x)));
    #w <- 1/(e$std.err+.Machine$double.eps)^2
    w <- e$n[e$strata]
    std.err = 1/sum(w, na.rm=TRUE)
    mean.error = sum(abs(e$surv-x)*w, na.rm=TRUE)*std.err
    return(list(mean.error=mean.error, std.err=std.err, survfit=e, x=x))
}
```

Absolute prediction error

```
absPredError <- EvalAbsolutePred(multiRFX3$os, Surv(allData$time1, allData$time2, allData$status), time=3*365)
Error in findrow(fit[i], times, extend) : no points selected for one or more curves, consider using the extend argument
plot(absPredError$x, absPredError$survfit$surv, xlim=c(0,1), ylim=c(0,1), xlab="Predicted probability", ylab="Observed", main="Prediction tool")
segments(absPredError$x, absPredError$survfit$lower,absPredError$x, absPredError$survfit$upper)
abline(0,1)
```

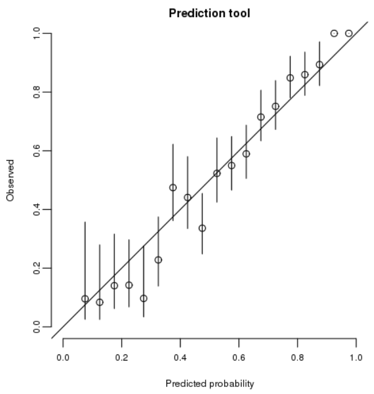

After adding ***extend=T*** to ***e <- summary(f, time)*** in function ***EvalAbsolutePred*** as suggested in the error message above, we have:

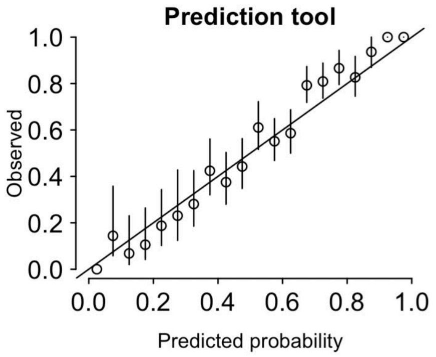

[outputs of the rerun (2021)]

```
PredictAbsoluteCoxph <- function(coxRFXOsCR, allData, time) {
    s <- survfit(coxRFXOsCR)
    q <- s$surv[which.min(abs(s$time-time))] ^ exp(predict(coxRFXOsCR, newdata=allData))
}
q <- PredictAbsoluteCoxph(coxRFXOsCR = coxRFXOsCR, allData = allData, time=365)

absPredErrorOs <- EvalAbsolutePred(q, Surv(allData$time1, allData$time2, allData$status), time=365)
plot(absPredErrorOs$x, absPredErrorOs$survfit$surv, xlim=c(0,1), ylim=c(0,1), xlab="Predicted probability", ylab=
"Observed", main="RFX on OS")
segments(absPredErrorOs$x, absPredErrorOs$survfit$lower,absPredErrorOs$x, absPredErrorOs$survfit$upper)
abline(0,1)
```

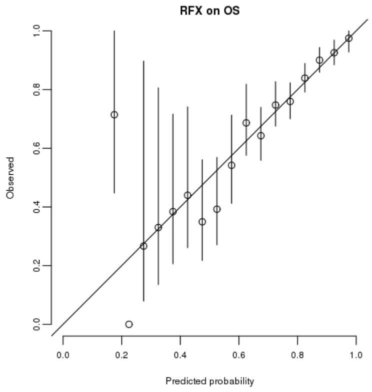

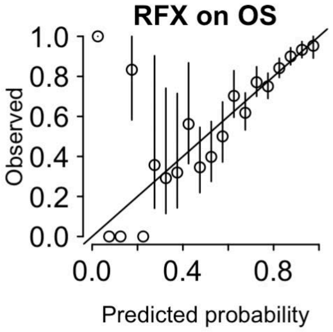

[outputs of the rerun (2021)]

Eval cross-validated samples

```
i <- 0
absoluteErrorsCIRcv <- lapply(list(crGroups[crGroups %in% mainGroups], crGroups), function(g){
            i <- i+1
            sapply(1:replicates, function(foo){
                        set.seed(foo)
                        time <- 365
                        trainIdx <- sample(1:nrow(dataFrame)%%5 +1 )!=1 ## sample 4/5
                        coef <- concordanceCIRcv[[i]][[foo]][["coef"]]

                        lpCIR <- as.matrix(relData[names(coef[,"CIRrfx"])]) %*% coef[,"CIRrfx"]
                        s <- survfit(Surv(time1, time2, status)~1, data=relData, subset=relData$index %in% which(
trainIdx))
                        pCIR <- s$surv[which.min(abs(s$time-time))] ^ exp(lpCIR-mean(lpCIR[relData$index %in%whic
h(trainIdx)]))

                        lpPRS <- as.matrix(prdData[names(coef[,"PRSrfx"])]) %*% coef[,"PRSrfx"]
                        s <- survfit(Surv(time2- time1, status)~1, data=prdData, subset=prdData$index %in% which(
trainIdx))
                        pPRS <- s$surv[which.min(abs(s$time-time))] ^ exp(lpPRS-mean(lpPRS[prdData$index %in% whi
ch(trainIdx)]))

                        lpNRM <- as.matrix(nrdData[names(coef[,"NRMrfx"])]) %*% coef[,"NRMrfx"]
                        s <- survfit(Surv(time1, time2, status)~1, data=nrdData, subset=nrdData$index %in% which(
trainIdx))
                        pNRM <- s$surv[which.min(abs(s$time-time))] ^ exp(lpNRM-mean(lpNRM[nrdData$index %in% whi
ch(trainIdx)]))

                        lpOS <- as.matrix(osData[names(coef[,"OSrfx"])]) %*% coef[,"OSrfx"]
                        s <- survfit(Surv(time1, time2, status)~1, data=osData, subset=osData$index %in% which(tr
ainIdx))
                        pOS <- s$surv[which.min(abs(s$time-time))] ^ exp(lpOS-mean(lpOS[osData$index %in% which(t
rainIdx)]))

                        p365 <- concordanceCIRcv[[i]][[foo]][["allRisk365"]]$os
                        p1000 <- concordanceCIRcv[[i]][[foo]][["allRisk1000"]]$os
                        err <- sapply(list(train=which(trainIdx), test=which(!trainIdx)), function(w)
                                    c(
                                                CIRrfx = EvalAbsolutePred(pCIR[relData$index %in% w ], Surv(relData$t
ime1, relData$time2, relData$status)[relData$index %in% w ], time=365)$mean.error,
                                                PRSrfx =  EvalAbsolutePred(pPRS[prdData$index %in% w ], Surv(prdData$
time1, prdData$time2, prdData$status)[prdData$index %in% w ], time=365)$mean.error,
                                                NRMrfx =  EvalAbsolutePred(pNRM[nrdData$index %in% w ], Surv(nrdData$
time1, nrdData$time2, nrdData$status)[nrdData$index %in% w ], time=365)$mean.error,
                                                OSrfx =  EvalAbsolutePred(pOS[osData$index %in% w ], Surv(osData$time
1, osData$time2, osData$status)[osData$index %in% w ], time=365)$mean.error,
                                                OS365 =  EvalAbsolutePred(p365[osData$index %in% w ], Surv(osData$tim
e1, osData$time2, osData$status)[osData$index %in% w ], time=365)$mean.error,
                                                OS1000 =  EvalAbsolutePred(p1000[osData$index %in% w ], Surv(osData$t
ime1, osData$time2, osData$status)[osData$index %in% w ], time=1000)$mean.error
                                    ))
                        return(err)
                  }, simplify='array')
        })

summary(t(absoluteErrorsCIRcv[[2]][,1,]))
```

```
##      CIRrfx            PRSrfx            NRMrfx            OSrfx             OS365             OS1000
##  Min.   :0.03669   Min.   :0.03836   Min.   :0.01515   Min.   :0.03073   Min.   :0.02095   Min.   :0.03204
##  1st Qu.:0.04551   1st Qu.:0.05974   1st Qu.:0.02360   1st Qu.:0.04013   1st Qu.:0.03136   1st Qu.:0.04235
##  Median :0.04949   Median :0.06497   Median :0.02768   Median :0.04354   Median :0.03477   Median :0.04800
##  Mean   :0.05081   Mean   :0.06524   Mean   :0.02781   Mean   :0.04377   Mean   :0.03576   Mean   :0.04801
##  3rd Qu.:0.05629   3rd Qu.:0.07118   3rd Qu.:0.03175   3rd Qu.:0.04767   3rd Qu.:0.03938   3rd Qu.:0.05295
##  Max.   :0.06849   Max.   :0.08820   Max.   :0.04339   Max.   :0.05674   Max.   :0.05117   Max.   :0.07093
```

```
boxplot(t(absoluteErrorsCIRcv[[2]][,1,]), main="Training")
```

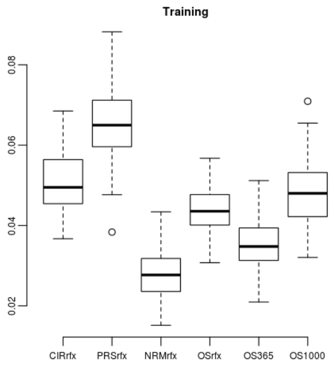


```
> summary(t(absoluteErrorsCIRcv[[2]][,1,]))
     CIRrfx            PRSrfx            NRMrfx            OSrfx             OS365             OS1000
 Min.   :0.03208   Min.   :0.04022   Min.   :0.01849   Min.   :0.03119   Min.   :0.03542   Min.   :0.04322
 1st Qu.:0.04793   1st Qu.:0.05496   1st Qu.:0.02640   1st Qu.:0.03983   1st Qu.:0.04992   1st Qu.:0.05336
 Median :0.05282   Median :0.06485   Median :0.02960   Median :0.04475   Median :0.05400   Median :0.05726
 Mean   :0.05241   Mean   :0.06442   Mean   :0.03012   Mean   :0.04408   Mean   :0.05465   Mean   :0.05825
 3rd Qu.:0.05756   3rd Qu.:0.07089   3rd Qu.:0.03357   3rd Qu.:0.04815   3rd Qu.:0.05963   3rd Qu.:0.06243
 Max.   :0.07806   Max.   :0.09203   Max.   :0.04413   Max.   :0.05574   Max.   :0.06898   Max.   :0.08067
```

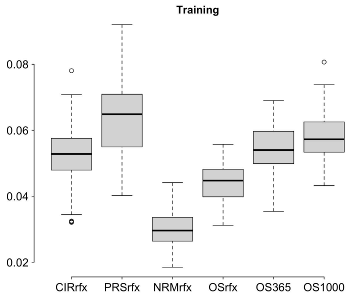

[outputs of the rerun (2021)]

```
summary(t(absoluteErrorsCIRcv[[2]][,2,]))
```

```
##      CIRrfx             PRSrfx             NRMrfx             OSrfx              OS365              OS1000       
##  Min.   :0.04373   Min.   :0.07699   Min.   :0.01895   Min.   :0.04045   Min.   :0.02840   Min.   :0.05755  
##  1st Qu.:0.07425   1st Qu.:0.10945   1st Qu.:0.03944   1st Qu.:0.06770   1st Qu.:0.05942   1st Qu.:0.08178  
##  Median :0.08979   Median :0.12459   Median :0.04902   Median :0.08073   Median :0.07175   Median :0.09328  
##  Mean   :0.08967   Mean   :0.12564   Mean   :0.04901   Mean   :0.07998   Mean   :0.07166   Mean   :0.09584  
##  3rd Qu.:0.10427   3rd Qu.:0.13970   3rd Qu.:0.05801   3rd Qu.:0.09100   3rd Qu.:0.08469   3rd Qu.:0.10879  
##  Max.   :0.15110   Max.   :0.19326   Max.   :0.08479   Max.   :0.12246   Max.   :0.12315   Max.   :0.14889  
```

```
boxplot(t(absoluteErrorsCIRcv[[2]][,2,]), main="Test")
```

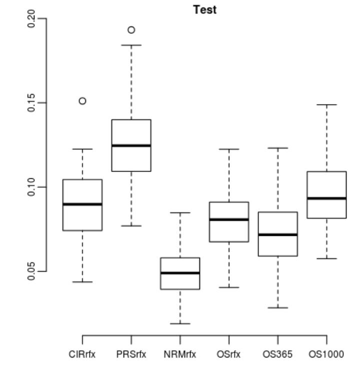


```
> summary(t(absoluteErrorsCIRcv[[2]][,2,]))
     CIRrfx            PRSrfx            NRMrfx            OSrfx             OS365             OS1000       
 Min.   :0.05608   Min.   :0.07695   Min.   :0.02053   Min.   :0.04135   Min.   :0.05629   Min.   :0.06787  
 1st Qu.:0.08284   1st Qu.:0.10982   1st Qu.:0.04467   1st Qu.:0.07204   1st Qu.:0.07690   1st Qu.:0.09048  
 Median :0.09424   Median :0.12672   Median :0.05426   Median :0.08200   Median :0.09139   Median :0.10611  
 Mean   :0.09522   Mean   :0.12914   Mean   :0.05520   Mean   :0.08332   Mean   :0.09191   Mean   :0.10701  
 3rd Qu.:0.10803   3rd Qu.:0.14608   3rd Qu.:0.06544   3rd Qu.:0.09308   3rd Qu.:0.10417   3rd Qu.:0.12042  
 Max.   :0.14856   Max.   :0.18744   Max.   :0.11361   Max.   :0.12532   Max.   :0.13317   Max.   :0.16488  
```

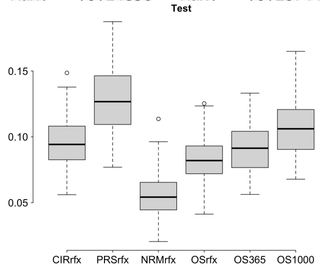

[outputs of the rerun (2021)]

3.6.6.2.1 KM curves after remission

```
riskCol=set1[c(1,3,4,2)]
names(riskCol) <- levels(clinicalData$M_Risk)
```

```
i <- 1
rsStatus <- osData$status
rsStatus[osData$index %in% nrdData$index[nrdData$status==1]] <- 0
par(mfrow=c(2,2), mar=c(3,3,1,1), bty="n", mgp=c(2,.5,0))
for(l in levels(clinicalData$M_Risk)[c(2,4,3,1)]){
    plot(NA,NA, ylim=c(0,1),  xlab="Years", ylab="Mortality", xlim=c(0,10), yaxs='i', xaxs='i')
    abline(h=seq(0.2,0.8,0.2),lty=1, col='lightgrey')
    #abline(v=seq(1,9), col='lightgrey')
    lines(survfit(Surv(time1/365, time2/365, status) ~ clinicalData$M_Risk[osData$index], data=osData, subset=cli
nicalData$M_Risk[osData$index]==l), col=riskCol[l],fun=function(x) 1-x ,mark=NA, lty=1, conf.int=FALSE)
    rsKM <- survfit(Surv(time1/365, time2/365, rsStatus) ~ 1, data=osData, subset=  clinicalData$M_Risk[osData$in
dex]==l)
    nrsKM <- survfit(Surv(time1/365, time2/365, status) ~ 1, data=nrdData, subset=  clinicalData$M_Risk[nrdData$i
ndex]==l)

    rsCR <- cumsum(c(1,diff(rsKM$surv)) * splinefun(nrsKM$time, nrsKM$surv, method="monoH.FC")(rsKM$time))
    nrsCR <- cumsum(c(1,diff(nrsKM$surv)) * splinefun(rsKM$time, rsKM$surv, method="monoH.FC")(nrsKM$time))


    lines(rsKM$time, 1-rsCR, col=riskCol[l], lty=2, type='s')
    lines(nrsKM$time, 1-nrsCR, col=riskCol[l], lty=3, type='s')
    if(i ==1)
        legend(ifelse(i<=3,"topleft","bottomright"), c("total","relapse","non-rel"), lty=c(1,2,3), col="black", b
ox.lty = 0, bg="white")
    i <- i+1
    mtext(l, side=3, font=2)
}
```

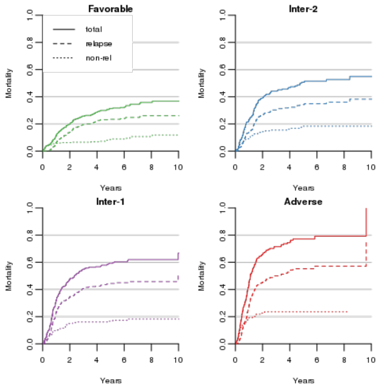

```
f <- function(x) 1-x
plot(survfit(Surv(time1/365, time2/365, status) ~ clinicalData$M_Risk[relData$index], data=relData), col=riskCol,
ylab="CIR", xlab="Time after CR", main="Molecular risk groups, all cases", fun=f , ylim=c(0,1))
legend("bottomright", lty=1, bty="n", paste(levels(clinicalData$M_Risk), table(clinicalData$M_Risk[!is.na(c)])),
col=riskCol)
```

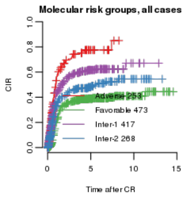

Incidence of relapse v risk tercile

```
par(mfrow=c(2,2), mar=c(3,3,1,1), bty="n", mgp=c(2,.5,0))
riskCirTD <- coxRFXRelTD$Z %*% coef(coxRFXRelTD) - relData$transplantCR1 * coef(coxRFXRelTD)["transplantCR1"]
quantileRiskCirTD <- numeric(nrow(relData))
for(l in levels(clinicalData$M_Risk)[c(2,4,3,1)]){
    w <- which(clinicalData$M_Risk[relData$index]==l)
    q <- cut(riskCirTD[w], quantile(riskCirTD[w], seq(0,1,.33)), include.lowest=TRUE, labels=c("T1","T2","T3"))
    quantileRiskCirTD[w] <- q
    plot(NA,NA,  ylab="CIR", main=paste(l, "terciles"),  xlab="Years after CR", ylim=c(0,1), xlim=c(0,10), xaxs="
i", yaxs="i")
    #abline(h=seq(0.2,0.8,0.2),lty=1, col='lightgrey')
    fit <- survfit(Surv(time1/365, time2/365, status) ~ q + transplantCR1, data=relData[w,])
    ## adjust for competing risk (NRM)
    i <- c(0,diff(fit$surv))
    s <- split(fit$surv, cumsum(i>0)) # split into strata
    u <- split(fit$upper, cumsum(i>0)) # split into strata
    v <- split(fit$lower, cumsum(i>0)) # split into strata

    t <- split(fit$time, cumsum(i>0))
    nrsKM <- survfit(Surv(time1/365, time2/365, status) ~ 1, data=nrdData, subset=  clinicalData$M_Risk[nrdData$i
ndex]==l)

    fit$surv <- unlist(sapply(seq_along(s), function(i) cumsum(c(1,diff(s[[i]])) * splinefun(nrsKM$time, nrsKM$su
rv, method="monoH.FC")(t[[i]])))) #adjust increments by nrs KM est
    fit$lower <- unlist(sapply(seq_along(s), function(i) cumsum(c(1,diff(v[[i]])) * splinefun(nrsKM$time, nrsKM$s
urv, method="monoH.FC")(t[[i]])))) #adjust increments by nrs KM est
    fit$upper <- unlist(sapply(seq_along(s), function(i) cumsum(c(1,diff(u[[i]])) * splinefun(nrsKM$time, nrsKM$s
urv, method="monoH.FC")(t[[i]])))) #adjust increments by nrs KM est
    lines(fit, col=rep(sapply(2:0,function(x) colTrans(riskCol[l],x)), each=2), lty=c(1,0), mark=NA, xlab="Time a
fter CR", fun=f)
    #legend("bottomright", lty=c(1,3), bty="n", c("no TPL","TPL"), col=riskCol[l])
}
```

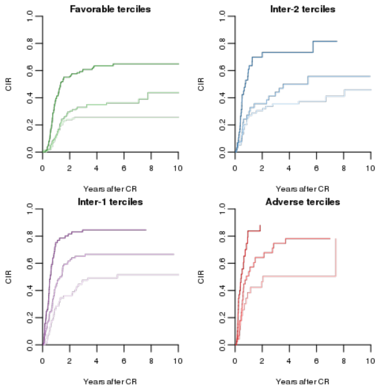

We use the `survival` package to compute the following mstate fits of CIR and NRM

```
t <- clinicalData$Recurrence_date
t[is.na(t)] <-  clinicalData$Date_LF[is.na(t)]
time <- as.numeric(pmin(t, clinicalData$Date_LF) - clinicalData$CR_date)
status <- factor(ifelse(!is.na(clinicalData$Recurrence_date), "relapse", ifelse(clinicalData$Status==1,"dead","al
ive"  )))
status[is.na(clinicalData$CR_date)] <- NA
alloCR1 <- 1:1540 %in% osData$index[osData$transplantCR1==1]
mSurv <- Surv(time/365.25, status, type="mstate")

f <- function(x) 1-x
```

```
par(mfrow=c(2,2), mar=c(3,3,1,1), bty="n", mgp=c(2,.5,0))
riskCir <- (coxRFXRelTD$Z %*% coef(coxRFXRelTD) - relData$transplantCR1 * coef(coxRFXRelTD)["transplantCR1"])[1:1
540] # Risk w/o allograft
qtl <- numeric(nrow(dataFrame))
for(l in levels(clinicalData$M_Risk)[c(2,4,3,1)]){
    w <- which(clinicalData$M_Risk==l)
    q <- cut(riskCir[w], quantile(riskCir[w], seq(0,1,.33)), include.lowest=TRUE, labels=c("T1","T2","T3"))
    qtl[w] <- q
    plot(NA,NA,  ylab="Fraction relapsed", main=paste0(l,", n=",sum(clinicalData$M_Risk[!is.na(mSurv)]==l, na.rm=
TRUE)),  xlab="Years after CR", ylim=c(0,1), xlim=c(0,5), xaxs="i", yaxs="i", font.main=1)
    #abline(h=seq(0.2,0.8,0.2),lty=1, col='lightgrey')
    fit <- survfit(mSurv~ qtl, subset= clinicalData$M_Risk==l)

    lines(fit, col=sapply(2:0, function(x) c(colTrans(set1[2],x), colTrans(set1[5],x))), lty=c(1,1), mark=NA, xla
b="Time after CR", fun=f)
    #legend("bottomright", lty=c(1,3), bty="n", c("no TPL","TPL"), col=riskCol[l])
}
```

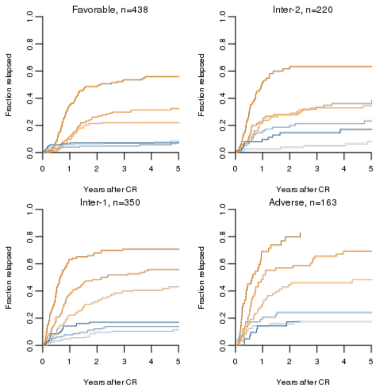

Overall survival after remission v risk tercile

```
par(mfrow=c(2,2), mar=c(3,3,1,1), bty="n", mgp=c(2,.5,0))
riskOsCR <- coxRFXOsCR$Z %*% coef(coxRFXOsCR) - osData$transplantCR1 * coef(coxRFXOsCR)["transplantCR1"]
quantileRiskOsCR <- numeric(nrow(osData))
for(l in levels(clinicalData$M_Risk)[c(2,4,3,1)]){
    w <- which(clinicalData$M_Risk[osData$index]==l)
    q <- cut(riskOsCR[w], quantile(riskOsCR[w], seq(0,1,.33)), include.lowest=TRUE, labels=c("T1","T2","T3"))
    quantileRiskOsCR[w] <- q
    plot(NA,NA,  ylab="OS", main=paste(l, "terciles"),  xlab="Years after CR", ylim=c(0,1), xlim=c(0,10), xaxs="i
", yaxs="i")
    abline(h=seq(0.2,0.8,0.2),lty=1, col='lightgrey')
    fit <- survfit(Surv(time1/365, time2/365, status) ~ q + transplantCR1, data=osData[w,])
    lines(fit, col=rep(sapply(2:0,function(x) colTrans(riskCol[l],x)), each=2), lty=c(1,3), mark=NA, xlab="Time a
fter CR")
    legend("bottomright", lty=c(1,3), bty="n", c("no TPL","TPL"), col=riskCol[l])
}
```

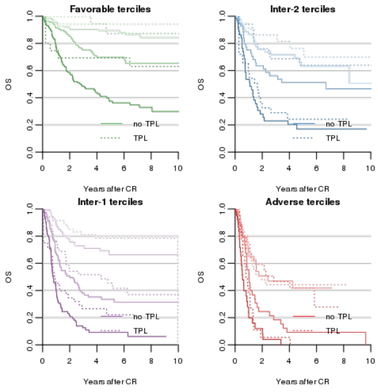

3.6.6.2.2 Risk factors of relapse and survival

```
p <- lapply(levels(clinicalData$M_Risk), function(l) {
            w <- which(clinicalData$M_Risk==l)
            q <- cut(riskOsCR[w], quantile(riskOsCR[w], seq(0,1,.33)), include.lowest=TRUE, labels=c("T1","T2","T
3"))
            sapply(split(relData[w, names(crGroups)], q), colMeans)
        })
names(p) <- levels(clinicalData$M_Risk)
```

```
par(mfrow=c(4,1), xpd=NA)
for(l in levels(clinicalData$M_Risk)[c(2,4,3,1)]){
    t <- t((p[[l]]) * coef(coxRFXRelTD))[,crGroups != "Treatment"]
    z <- (coef(coxRFXRelTD)/sqrt(diag(coxRFXRelTD$var2)))[crGroups != "Treatment"]
    o <- order(z)
    w <- c(1:15,ncol(t)-14:0)
    b <- barplot(t[,o][,w], las=2, col=sapply(2:0,function(x) colTrans(riskCol[l],x)), beside=TRUE, ylim=c(-.5,.5
), names.arg=rep("", length(w)))
    rotatedLabel(b[2,],pmin(0,apply(t,2,min)[o][w]), colnames(t)[o][w])
    s <- matrix(rep(sqrt(diag(coxRFXRelTD$var2)[crGroups != "Treatment"]), each=3) * t/rep(coef(coxRFXRelTD)[crGr
oups != "Treatment"], each=3), nrow=3)[,o][,w]
    segments(b[1,], (colMeans(coxRFXRelTD$Z)*coef(coxRFXRelTD))[crGroups != "Treatment"][o][w] ,b[3,], (colMeans(
coxRFXRelTD$Z)*coef(coxRFXRelTD))[crGroups != "Treatment"][o][w])
    segments(b,t[,o][,w]-s, b,t[,o][,w]+s)
}
```

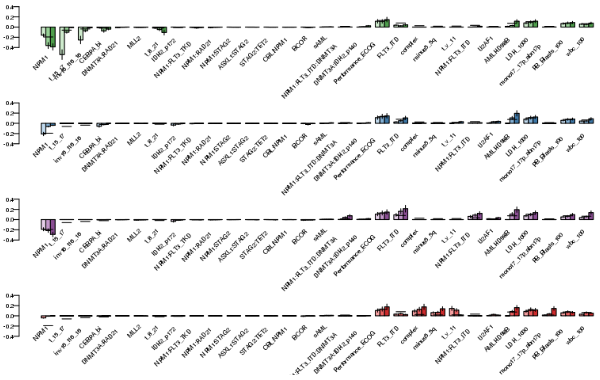

```
#p <- as.data.frame(PartialRisk(coxRFXRelTD)[1:nrow(clinicalData),])
partialRiskOsCR <- as.data.frame(PartialRisk(coxRFXOsCR)[1:nrow(clinicalData),])
s <- do.call("rbind",lapply(levels(clinicalData$M_Risk)[c(2,4,3,1)], function(l) {
                    w <- which(clinicalData$M_Risk==l)
                    q <- cut(riskOsCR[w], quantile(riskOsCR[w], seq(0,1,.33)), include.lowest=TRUE, labels=c("T1"
,"T2","T3"))
                    t(sapply(split(partialRiskOsCR[w, ], q), colMeans) +.5 - colMeans(partialRiskOsCR))
                }))
```

Risk constellation for OS after remission

```
c <- sapply(2:0, function(t) sapply(riskCol[c(2,4,3,1)], function(c) colTrans(c,t)))
g <- expand.grid(1:3,1:4-1)*3
stars(2*s[,c("Clinical","Demographics","Genetics","GeneGene","CNA","Fusions","Treatment")], scale=FALSE, col.star
s=t(c), key.loc = c(13,0), locations=g, labels=NA)
symbols(g[,1], g[,2], circles=rep(1,12), inches=FALSE, add=TRUE)
text(1, 0:3*3, names(riskCol[c(2,4,3,1)]), pos=2)
text(1:3*3, 11, c("Best","Intermediate","Worst"), pos=3)
```

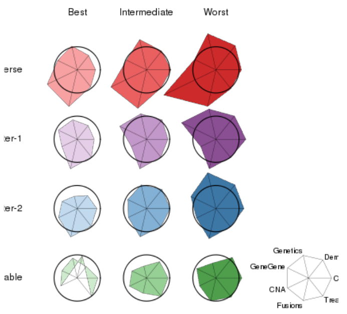

Prototypical risk constellations

```
prototypes <- sapply(levels(clinicalData$M_Risk)[c(2,4,3,1)], function(l) sapply(1:3, function(i){
                        #d <- dist(as.data.frame(coxRFXRelTD$Z[which(clinicalData$M_Risk[cirData$index]==l & quantileRiskCirTD==i &! is.na(clinicalData$CR_date[cirData$index])), ]))
                        w <- which(clinicalData$M_Risk[osData$index]==l & quantileRiskOsCR==i &! is.na(clinicalData$CR_date[osData$index]))
                        d <- dist(t(t(coxRFXOsCR$Z[w, ]) ))
                        osData$index[w][which.min(rowMeans(as.matrix(d), na.rm=TRUE))]
                    }))

c <- sapply(2:0, function(t) sapply(riskCol[c(2,4,3,1)], function(c) colTrans(c,t)))
g <- expand.grid(1:3,1:4-1)*3
stars(2*t(t(partialRiskOsCR[prototypes,])- colMeans(partialRiskOsCR))[,c("Clinical","Demographics","Genetics","CNA","Fusions","Treatment")] +1, scale=FALSE, col.stars=t(c), key.loc = c(13,0), locations=g, labels=NA)
symbols(g[,1], g[,2], circles=rep(1,12), inches=FALSE, add=TRUE)
text(1, 0:3*3, names(riskCol[c(2,4,3,1)]), pos=2)
text(1:3*3, 11, c("Low","Intermediate","High"), pos=3)
```

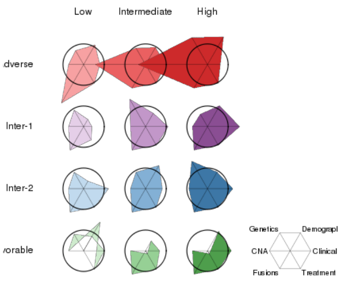

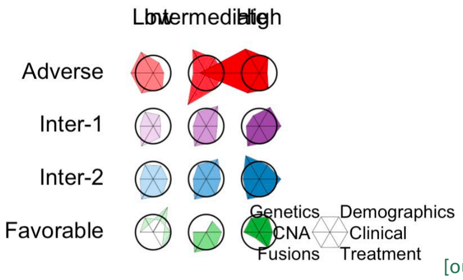

[outputs of the rerun (2021)]

```
s <- partialRiskOsCR - rep(colMeans(partialRiskOsCR), each=nrow(partialRiskOsCR))
w <- sapply(split(1:1540, paste(clinicalData$M_Risk, quantileRiskOsCR[1:1540])), `[`, 1:12)
w <- w[,!grepl("NA", colnames(w))][,c(4:6,10:12,7:9,1:3)]
l <- stars(s[w,c("Demographics","Treatment","Fusions","CNA","Genetics","GeneGene","Clinical")] + .5, scale=FALSE, col.stars = mapply(function(i,j) {t <- try(c[i,j]); if(class(t)=="try-error") NA else t}, as.character(clinicalData$M_Risk[w]),quantileRiskCirTD[w]), labels="")
symbols(l[,1],l[,2], circles=rep(0.5, nrow(l)), inches=FALSE,add=TRUE)
```

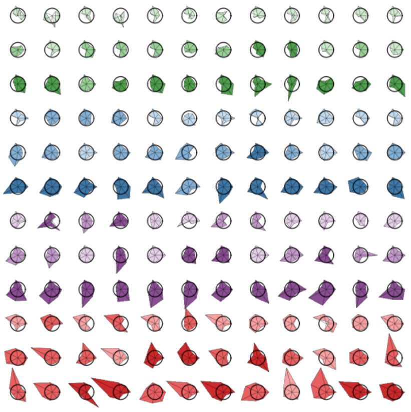

```
layout(matrix(c(1:4), ncol=2),heights = c(10,1), widths = c(10,1))
partialRiskCirTD <- as.data.frame(PartialRisk(coxRFXRelTD))
s <- partialRiskCirTD[1:nrow(clinicalData),] - rep(colMeans(partialRiskCirTD), each=nrow(clinicalData))
u <- unique(relData$index[!is.na(relData$time2)])
w <- sapply(split(u, paste(clinicalData$M_Risk, quantileRiskCirTD[1:1540])[u]), `[`, 1:12)
w <- w[,!grepl("NA", colnames(w))][,c(4:6,10:12,7:9,1:3)]
i <- which(rev(!duplicated(rev(relData$index))))
m <- i[order(relData$index[i])]
c <- cut(relData$time2, quantile(relData$time2[m], seq(0,1,0.1), na.rm=TRUE))
l <- mg14:::stars(s[w,c("Demographics","Treatment","Fusions","CNA","Genetics","GeneGene","Clinical")] + .5, scale=FALSE, col.stars = brewer.pal(11,"RdBu")[-6][c[w]], labels="", density=ifelse(relData$status[m][w]==1,NA,48),  col.lines=rep(1,(12^2)))
symbols(l[,1],l[,2], circles=rep(0.5, nrow(l)), inches=FALSE,add=TRUE, fg='lightgrey')
par(mar=c(2,2,0,2))
barplot(matrix(diff(quantile(relData$time2[m], na.rm=T, seq(0,1,0.1))), ncol=1)/365.25, col=brewer.pal(11,"RdBu")[-6], horiz=TRUE, border=NA, xlim=c(0,20))
```

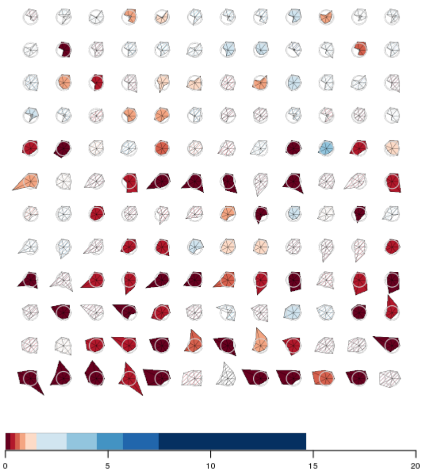

3.6.6.3 Allogeneic hematopoietic stem cell transplants

Create a data.frame with all possibilities for allografts - none, CR1, after relapse.

```
w <- sort(unique(osData$index[which(quantileRiskOsCR==3 & clinicalData$M_Risk[osData$index]=="Favorable")]))
multiRFX3Tpl <- MultiRFX3(coxRFXNrdTD, coxRFXRelTD, coxRFXPrdTD, data=allDataTpl, x=3*365, prdData=prdData)
multiRFX3Tpl <- as.data.frame(matrix(multiRFX3Tpl$os, ncol=3, byrow=TRUE, dimnames=list(NULL, c("None","CR1","Relapse"))), row.names=rownames(dataFrame))
survivalTpl <- data.frame(multiRFX3Tpl, os=osYr, age=clinicalData$AOD, ELN=clinicalData$M_Risk, tercile=quantileRiskOsCR[1:nrow(multiRFX3Tpl)])
```

```
datatable(format(survivalTpl[order(survivalTpl$CR1 -survivalTpl$Relapse),], digits=4))
```

Show 10 **entries** **Search:**

	None	CR1	Relapse	os	age	ELN	tercile
PD11027a	0.278584	0.166553	0.343992	0.216438356	56	Inter-1	3
PD8237a	0.538942	0.414715	0.559086	0.369863014	50	Adverse	1
PD11062a	0.496207	0.407844	0.536002	1.556164384	27	Adverse	2
PD10891a	0.405365	0.317522	0.439537	5.243835616+	47	Favorable	3
PD8068a	0.324768	0.230607	0.350785	0.172602740	60	Inter-1	3
PD11040a	0.353202	0.285691	0.400263	0.484931507	55	Inter-2	2
PD8255a	0.513728	0.431842	0.545903	7.367123288+	34	Adverse	2
PD8014a	0.597127	0.517558	0.629677	5.227397260+	39	Inter-2	1
PD10927a	0.434004	0.392176	0.498444	1.687671233	28	Adverse	2
PD7739a	0.409750	0.323282	0.428542	0.200000000	57	Favorable	3

Showing 1 to 10 of 1,540 entries Previous 1 2 3 4 5 … 154 Next

```
datatable(multiRFX3Tpl[patients,])
```

Show 10 **entries** **Search:**

	None	CR1	Relapse
PD10828a	0.728326141287733	0.775881095510976	0.761343700364516
PD10844a	0.877500850156228	0.911470802687657	0.894725652526159
PD10829a	0.416222049802171	0.560709606542194	0.476399131001701
PD10996a	0.27118369500072	0.271664243539793	0.351294646170179

Showing 1 to 4 of 4 entries Previous 1 Next

Function to predict OS from Relapse, PRS and NRM. This one also computes confidence intervals for each type of allograft and the predicted differences in outcome between allograft types.

```
MultiRFX3TplCi <- function(coxRFXNrdTD, coxRFXRelTD, coxRFXPrdTD, data, x =365, prdData, ciType="simulated", nSim = 200, mc.cores=10){
```

```
    ## Step 1: Compute KM survival curves and log hazard
    getS <- function(coxRFX, data, max.x=5000) {
        if(!is.null(coxRFX$na.action)) coxRFX$Z <- coxRFX$Z[-coxRFX$na.action,]
        data <- as.matrix(data[,match(colnames(coxRFX$Z),colnames(data))])
        r <- PredictRiskMissing(coxRFX, data, var="var2")
        H0 <- basehaz(coxRFX, centered = FALSE)
        hazardDist <- splinefun(H0$time, H0$hazard, method="monoH.FC")
        x <- c(0:max.x,max.x)
        S <- exp(-hazardDist(x))
        return(list(S=S, r=r, x=x, hazardDist=hazardDist, r0 = coxRFX$means %*% coef(coxRFX)))
    }

    data$transplantCR1 <- 0
    data$transplantRel <- 0

    kmCir <- getS(coxRFX = coxRFXRelTD, data = data, max.x=max(x))
    kmNrs <- getS(coxRFX = coxRFXNrdTD, data = data, max.x=max(x))
    kmPrs <- getS(coxRFX = coxRFXPrdTD, data = data, max.x=max(x))

    survPredict <- function(surv){
        s <- survfit(surv~1)
        splinefun(s$time, s$surv, method="monoH.FC")
    }
    xx <- 0:max(x)

    # Baseline Prs (measured from relapse)
    kmPrs0 <- survPredict(Surv(prdData$time1, prdData$time2, prdData$status))(xx)

    # PRS baseline with spline-based dep on CR length)
    coxphPrs <- coxph(Surv(time1, time2, status)~ pspline(time0, df=10), data=prdData)
    tdPrmBaseline <- exp(predict(coxphPrs, newdata=data.frame(time0=xx[-1])))

    stopifnot(length(x)==1 | length(x) == nrow(data))
    if(length(x)==nrow(data))
        w <- match(x,kmCir$x)
    else if(length(x)==1)
        w <- rep(match(x, kmCir$x), nrow(data))

    survival <- sapply(c("None","Rel","CR1"), function(type){
                if(type=="None"){
                    data$transplantCR1 <- 0
                    data$transplantRel <- 0
                }else if(type=="Rel"){
                    data$transplantCR1 <- 0
                    data$transplantRel <- 1
                }else if(type=="CR1"){
                    data$transplantCR1 <- 1
                    data$transplantRel <- 0
                }


                kmCir <- getS(coxRFX = coxRFXRelTD, data = data, max.x=max(x))
                kmNrm <- getS(coxRFX = coxRFXNrdTD, data = data, max.x=max(x))
                kmPrs <- getS(coxRFX = coxRFXPrdTD, data = data, max.x=max(x))

                ## Step 2: Adjust CIR and NRM curve for competing risks, accounting for hazard
                kmCir$Sadj <- sapply(1:nrow(data), function(i) cumsum(c(1,diff(kmCir$S^exp(kmCir$r[i,1]))) * kmNr
m$S ^ exp(kmNrm$r[i,1])))
                kmNrm$Sadj <- sapply(1:nrow(data), function(i) cumsum(c(1,diff(kmNrm$S^exp(kmNrm$r[i,1]))) * kmCi
r$S ^ exp(kmCir$r[i,1]))) ## array times x nrow(data)

                y <- mapply(function(i,j) kmNrm$Sadj[i,j], w,1:length(w) ) # select time for each sample
                nrs <- y
                nrsUp <- y^exp(2*sqrt(kmNrm$r[,2]))
                nrsLo <- y^exp(- 2*sqrt(kmNrm$r[,2]))

                y <- mapply(function(i,j) kmCir$Sadj[i,j], w,1:length(w) ) # select time for each sample
                cir <- y
                cirLo <- y^exp( 2*sqrt(kmCir$r[,2]))
                cirUp <- y^exp( - 2*sqrt(kmCir$r[,2]))

                ## Step 3: Compute post-relapse survival
                rs <- sapply(1:nrow(data), function(i){
                            ### Different approach
                            xLen <- 1+floor(x)
                            cir <- kmCir$Sadj[1:xLen,i]
                            rs <- computeTotalPrsC(x = xx, diffCir = diff(cir), prsP = kmPrs0, tdPrmBaseline = td
PrmBaseline, risk = kmPrs$r[i,1]-kmPrs$r0)
                            rs[xLen]
                        })

                ## Step 4: Combine into overall survival
                if(any(1-(1-rs)-(1-nrs)<0)) warning("OS < 0 occured.")
                os <- pmax(pmin(1-(1-rs)-(1-nrs),1),0)
                cbind(os, rs, nrs, aar=rs-cir)
            }, simplify='array')
```

```
    ## Step 5: Confidence intervals for OS
    osCi <- sapply(mclapply(1:nrow(data), function(i){
                        {
                            ## Simulate CI
                            osCiMc <- sapply(1:nSim, function(foo){
                                        r0 <- rnorm(3,c(kmCir$r[i,1],kmNrs$r[i,1],kmPrs$r[i,1]),sqrt(c(kmCir$r[i,
2],kmNrs$r[i,2],kmPrs$r[i,2])))
                                        H0 <- exp(r0)
                                        nrs0 <- cumsum(c(1,diff(kmNrs$S^H0[2])) * kmCir$S^H0[1]) ## Correct KM es
timate for competing risk
                                        diffCir <- diff(c(1,kmCir$S^H0[1])) * kmNrs$S^H0[2] ## Correct KM estimat
e for competing risk
                                        cir0 <- 1+cumsum(diffCir)
                                        rs0 <- computeTotalPrsC(x = xx, diffCir = diffCir, prsP = kmPrs0, tdPrmBa
seline = tdPrmBaseline, risk = -kmPrs$r0+log(H0[3]))
                                        aar0 <- rs0[1:w[i]]-cir0[1:w[i]]

                                        Hcr1 <- exp(r0 + rnorm(3,c(coxRFXRelTD$coefficients["transplantCR1"],coxR
FXNrdTD$coefficients["transplantCR1"],coxRFXPrdTD$coefficients["transplantCR1"]),
                                                sqrt(c(coxRFXRelTD$var2["transplantCR1","transplantCR1"],
coxRFXNrdTD$var2["transplantCR1","transplantCR1"],coxRFXPrdTD$var2["transplantCR1","transplantCR1"]))))
                                        nrsCr1 <- cumsum(c(1,diff(kmNrs$S^Hcr1[2])) * kmCir$S^Hcr1[1]) ## Correct
KM estimate for competing risk
                                        diffCir <- diff(c(1,kmCir$S^Hcr1[1])) * kmNrs$S^Hcr1[2] ## Correct KM est
imate for competing risk
                                        cirCr1 <- 1+cumsum(diffCir)
                                        rsCr1 <- computeTotalPrsC(x = xx, diffCir = diffCir, prsP = kmPrs0, tdPrm
Baseline = tdPrmBaseline, risk = -kmPrs$r0+log(Hcr1[3]))
                                        aarCr1 <- rsCr1[1:w[i]]-cirCr1[1:w[i]]


                                        Hrel <- exp(r0 + rnorm(3,c(coxRFXRelTD$coefficients["transplantRel"],coxR
FXNrdTD$coefficients["transplantRel"],coxRFXPrdTD$coefficients["transplantRel"]),
                                                sqrt(c(coxRFXRelTD$var2["transplantRel","transplantRel"],
coxRFXNrdTD$var2["transplantRel","transplantRel"],coxRFXPrdTD$var2["transplantRel","transplantRel"]))))
                                        nrsRel <- cumsum(c(1,diff(kmNrs$S^Hrel[2])) * kmCir$S^Hrel[1]) ## Correct
KM estimate for competing risk
                                        diffCir <- diff(c(1,kmCir$S^Hrel[1])) * kmNrs$S^Hrel[2] ## Correct KM est
imate for competing risk
                                        cirRel <- 1+cumsum(diffCir)
                                        rsRel <- computeTotalPrsC(x = xx, diffCir = diffCir, prsP = kmPrs0, tdPrm
Baseline = tdPrmBaseline, risk = -kmPrs$r0+log(Hrel[3]))
                                        aarRel <- rsRel[1:w[i]]-cirRel[1:w[i]]


                                        os0 <- (1-(1-nrs0[1:w[i]])-(1-rs0))[w[i]]
                                        osCr1 <- (1-(1-nrsCr1[1:w[i]])-(1-rsCr1))[w[i]]
                                        osRel <- (1-(1-nrsRel[1:w[i]])-(1-rsRel))[w[i]]
                                        return(cbind(os=c(none=os0, cr1=osCr1, rel=osRel, dCr1=osCr1-os0, dRel=os
Rel-os0, dCr1Rel=osCr1-osRel),
                                                rs=c(none=rs0[w[i]], cr1=rsCr1[w[i]], rel=rsRel[w[i]], dC
r1=rsCr1[w[i]]-rs0[w[i]], dRel=rsRel[w[i]]-rs0[w[i]], dCr1Rel=rsCr1[w[i]]-rsRel[w[i]]),
                                                nrs=c(none=nrs0[w[i]], cr1=nrsCr1[w[i]], rel=nrsRel[w[i]]
, dCr1=nrsCr1[w[i]]-nrs0[w[i]], dRel=nrsRel[w[i]]-nrs0[w[i]], dCr1Rel=nrsCr1[w[i]]-nrsRel[w[i]]),
                                                aar=c(none=aar0[w[i]], cr1=aarCr1[w[i]], rel=aarRel[w[i]]
, dCr1=aarCr1[w[i]]-aar0[w[i]], dRel=aarRel[w[i]]-aar0[w[i]], dCr1Rel=aarCr1[w[i]]-aarRel[w[i]])))
                                    }, simplify='array')
                            osCiMcQ <- apply(osCiMc,1:2,quantile, c(0.025,0.5,0.975))
                            return(sapply(c("os","rs","nrs","aar"), function(t)
                                            cbind(hat = c(survival[i,t,1], survival[i,t,3], survival[i,t,2],
survival[i,t,3]-survival[i,t,1], survival[i,t,2]-survival[i,t,1], survival[i,t,3]-survival[i,t,2]),
                                                median = osCiMcQ[2,,t], lower = osCiMcQ[1,,t], upper = os
CiMcQ[3,,t]), simplify="array"))
                        }
                    }, mc.cores=mc.cores), I, simplify="array")
    #cat(os, "\n")
    return(osCi)
}
```

```
set.seed(42)
d <- osData[1:nrow(dataFrame),]
d$transplantCR1 <- 0
d$transplantRel <- 0
p <- grep("PD11104a|PD8314a|PD10941a",rownames(dataFrame))
predict3 <- MultiRFX3TplCi(coxRFXNrdTD, coxRFXRelTD, coxRFXPrdTD, data=d[p,colnames(coxRFXNrdTD$Z)], x=3*365, nSi
m=1000, prdData=prdData) ## selected with 1000
dimnames(predict3)[[4]] <- rownames(dataFrame)[p]
predict3
```

```
## , , os, PD10941a
##
##                hat      median       lower      upper
## none     0.818663673 0.813758093 0.735781209 0.87846475
## cr1      0.837567294 0.833837913 0.728221120 0.89613925
```

```
## rel      0.841335270  0.837729406  0.758152717 0.89684242
## dCr1     0.018903622  0.016563073 -0.043991177 0.06893157
## dRel     0.022671597  0.021987031 -0.006177209 0.05224429
## dCr1Rel -0.003767975 -0.005622502 -0.067644137 0.04502109
##
## , , rs, PD10941a
##
##                hat     median        lower      upper
## none    0.88114586 0.88071728 0.8028936741 0.93390699
## cr1     0.94463616 0.94507091 0.8999107697 0.97099932
## rel     0.90381746 0.90559639 0.8318637923 0.95154789
## dCr1    0.06349030 0.06276975 0.0330569278 0.10630881
## dRel    0.02267160 0.02226157 0.0002740095 0.05254428
## dCr1Rel 0.04081871 0.03892768 0.0112503418 0.08418140
##
## , , nrs, PD10941a
##
##                 hat        median       lower       upper
## none     0.93751781  0.9377259143  0.87950077  0.96807394
## cr1      0.89293113  0.8921954102  0.79052095  0.94496498
## rel      0.93751781  0.9369624377  0.87673751  0.96928132
## dCr1    -0.04458668 -0.0447622293 -0.10283944 -0.01614686
## dRel     0.00000000 -0.0003878613 -0.01799561  0.01482152
## dCr1Rel -0.04458668 -0.0439414652 -0.10433321 -0.01148903
##
## , , aar, PD10941a
##
##                 hat      median        lower       upper
## none     0.16778126  0.16602329  0.098569191  0.26404124
## cr1      0.05952954  0.05832456  0.032368604  0.10513500
## rel      0.19045286  0.18806412  0.114633337  0.30149402
## dCr1    -0.10825172 -0.10691238 -0.163826806 -0.06454859
## dRel     0.02267160  0.02286218 -0.004982565  0.05963474
## dCr1Rel -0.13092332 -0.12981706 -0.207085090 -0.07409337
##
## , , os, PD11104a
##
##                hat     median        lower      upper
## none    0.84056298 0.83639387  0.752890309 0.89841989
## cr1     0.88621933 0.88221026  0.800876178 0.93160969
## rel     0.86553736 0.86095632  0.779960110 0.91781550
## dCr1    0.04565635 0.04427444 -0.005897815 0.09407140
## dRel    0.02497437 0.02388986 -0.002510175 0.05296898
## dCr1Rel 0.02068197 0.01937872 -0.029100606 0.07237330
##
## , , rs, PD11104a
##
##                hat     median         lower      upper
## none    0.87009314 0.87005765  0.7878001759 0.92237900
## cr1     0.93795534 0.93869458  0.8918465140 0.96652262
## rel     0.89506752 0.89493997  0.8132439573 0.94137070
## dCr1    0.06786219 0.06762730  0.0365378460 0.11655265
## dRel    0.02497437 0.02414719 -0.0007901814 0.05285589
## dCr1Rel 0.04288782 0.04308201  0.0133882399 0.09190801
##
## , , nrs, PD11104a
##
##                 hat        median       lower        upper
## none     0.97046984  0.9698761766  0.92402463  0.988322758
## cr1      0.94826399  0.9474848018  0.86036996  0.979862354
## rel      0.97046984  0.9690673487  0.91721959  0.988712274
## dCr1    -0.02220585 -0.0224939417 -0.06600414 -0.006582919
## dRel     0.00000000 -0.0001226097 -0.01089082  0.009718243
## dCr1Rel -0.02220585 -0.0216192196 -0.06747818 -0.005121411
##
## , , aar, PD11104a
##
##                 hat      median        lower       upper
## none     0.19080344  0.18803262  0.117200743  0.28859824
## cr1      0.06981669  0.06730478  0.036730974  0.12009207
## rel      0.21577782  0.21368379  0.136124880  0.32761145
## dCr1    -0.12098675 -0.11886572 -0.178730740 -0.07408171
## dRel     0.02497437  0.02483472 -0.004621345  0.06238499
## dCr1Rel -0.14596112 -0.14404519 -0.219428528 -0.09029031
##
## , , os, PD8314a
##
##                hat    median        lower     upper
## none    0.48820503 0.4881795  0.346459914 0.6095472
## cr1     0.67849561 0.6772427  0.551822750 0.7668568
## rel     0.54208498 0.5424903  0.395004579 0.6651239
## dCr1    0.19029058 0.1856732  0.101625028 0.2656564
## dRel    0.05387995 0.0522543 -0.006530565 0.1162451
## dCr1Rel 0.13641063 0.1321234  0.036290833 0.2415983
##
## , , rs, PD8314a
```

```
> predict3
, , os, PD10941a

               hat     median       lower      upper
none    0.79997794 0.79580536  0.69668429 0.86546735
cr1     0.83411592 0.83062446  0.72397488 0.89235699
rel     0.82262688 0.81942109  0.72341163 0.88221860
dCr1    0.03413798 0.03231478 -0.03913475 0.09561705
dRel    0.02264894 0.02229687 -0.00757331 0.05349902
dCr1Rel 0.01148904 0.00957548 -0.06046428 0.06991335

, , rs, PD10941a

               hat     median        lower      upper
none    0.86069487 0.86071484  0.762300046 0.92103990
cr1     0.93712931 0.93826666  0.886666582 0.96708969
rel     0.88334381 0.88470591  0.791470654 0.93772420
dCr1    0.07643444 0.07488964  0.042761092 0.12842914
dRel    0.02264894 0.02159045 -0.004073383 0.05143396
dCr1Rel 0.05378550 0.05309494  0.020168256 0.10587895

, , nrs, PD10941a

                hat        median       lower       upper
none     0.93928307  0.9391554081  0.88207064  0.96800479
cr1      0.89698661  0.8973082823  0.79305947  0.94715160
rel      0.93928307  0.9397034579  0.88123144  0.96947877
dCr1    -0.04229646 -0.0415580731 -0.10807820 -0.01486833
dRel     0.00000000  0.0002080128 -0.01732593  0.01475103
dCr1Rel -0.04229646 -0.0418451563 -0.10828802 -0.01006861

, , aar, PD10941a

                hat      median        lower       upper
none     0.14019726  0.13782269  0.084520504  0.21929982
cr1      0.04969161  0.04795044  0.025160214  0.09243314
rel      0.16284620  0.15959254  0.094922667  0.25910535
dCr1    -0.09050565 -0.08865005 -0.133815470 -0.05502451
dRel     0.02264894  0.02181524 -0.001973287  0.05370493
dCr1Rel -0.11315459 -0.11050083 -0.173053080 -0.06384424

, , os, PD11104a

               hat     median        lower      upper
none    0.82265695 0.82200115  0.734091245 0.88609251
cr1     0.88245205 0.87958717  0.797816045 0.92964026
rel     0.84706742 0.84642135  0.758797802 0.90630982
dCr1    0.05979511 0.05697213  0.006996882 0.11233419
dRel    0.02441048 0.02356702 -0.004032907 0.05270372
dCr1Rel 0.03538463 0.03243228 -0.018347285 0.08758233

, , rs, PD11104a

               hat     median        lower      upper
none    0.85110632 0.85293199  0.770133657 0.91482589
cr1     0.93158868 0.93301389  0.885930917 0.96191582
rel     0.87551680 0.87879099  0.796759475 0.93094553
dCr1    0.08048236 0.07885156  0.042094959 0.12837655
dRel    0.02441048 0.02346990 -0.002580453 0.05148218
dCr1Rel 0.05607188 0.05414821  0.019235456 0.10624974

, , nrs, PD11104a

                hat        median       lower        upper
none     0.97155062  0.9725298776  0.93467801  0.988177611
cr1      0.95086337  0.9513720013  0.87592593  0.981061134
rel      0.97155062  0.9724396866  0.93137471  0.989074256
dCr1    -0.02068725 -0.0199698409 -0.05926447 -0.005498843
dRel     0.00000000  0.0001295742 -0.01019666  0.008572254
dCr1Rel -0.02068725 -0.0200329583 -0.06182988 -0.004042374

, , aar, PD11104a

                hat      median       lower       upper
none     0.15411573  0.15240565  0.09665206  0.23181202
cr1      0.05612391  0.05584734  0.03026617  0.09882656
rel      0.17852621  0.17738098  0.10893126  0.27056164
dCr1    -0.09799182 -0.09655186 -0.14617357 -0.06187148
dRel     0.02441048  0.02303449 -0.00254950  0.05587120
dCr1Rel -0.12240230 -0.12024467 -0.18031613 -0.07516525

, , os, PD8314a

               hat     median       lower      upper
none    0.44271008 0.44088270  0.31243758 0.58132423
cr1     0.66194728 0.65732203  0.53630493 0.75815362
rel     0.48290475 0.48277812  0.33010365 0.62326972
dCr1    0.21923720 0.21323111  0.13103406 0.28548157
dRel    0.04019467 0.03940165 -0.01999899 0.09733149
dCr1Rel 0.17904253 0.17289487  0.07291901 0.26789095

, , rs, PD8314a

               hat     median       lower      upper
none    0.48570730 0.48654378  0.35500779 0.62360719
cr1     0.74635599 0.74692937  0.63379290 0.83890479
rel     0.52590197 0.53081277  0.37331054 0.67050040
dCr1    0.26064869 0.25592810  0.18732443 0.32668085
dRel    0.04019467 0.04008989 -0.02095379 0.09812361
dCr1Rel 0.22045402 0.21284279  0.12473996 0.31232770

, , nrs, PD8314a

               hat        median       lower       upper
none     0.9570028  0.9573853414  0.91587222  0.97927163
cr1      0.9155913  0.9167951196  0.82845830  0.96055621
rel      0.9570028  0.9570458681  0.90990956  0.98105739
dCr1    -0.0414115 -0.0395097400 -0.09353489 -0.01575280
dRel     0.0000000 -0.0002405708 -0.01477132  0.01177916
dCr1Rel -0.0414115 -0.0392744978 -0.09509183 -0.01400980

, , aar, PD8314a

                hat      median        lower       upper
none     0.10281055  0.10213752  0.054042258  0.17062886
cr1      0.04371070  0.04354594  0.019622075  0.08655167
rel      0.14300522  0.14263501  0.078542831  0.23456024
dCr1    -0.05909986 -0.05676395 -0.098854951 -0.02644740
dRel     0.04019467  0.03922021  0.004179576  0.08289283
dCr1Rel -0.09929452 -0.09678776 -0.165300483 -0.04818205
```

[outputs of the rerun (2021)]

```
##
##                hat     median        lower     upper
## none    0.53198035 0.53396623  0.391145882 0.6595976
## cr1     0.76445697 0.76880775  0.658094276 0.8472067
## rel     0.58586030 0.58956092  0.437185335 0.7242483
## dCr1    0.23247662 0.22964120  0.159395090 0.3104094
## dRel    0.05387995 0.05361889 -0.007811998 0.1136675
## dCr1Rel 0.17859667 0.17710282  0.086782790 0.2753473
##
## , , nrs, PD8314a
##
##                 hat        median       lower       upper
## none     0.95622468  0.9564742160  0.91389070  0.97815004
## cr1      0.91403863  0.9143142553  0.82517813  0.95636212
## rel      0.95622468  0.9559808647  0.91109939  0.97855350
## dCr1    -0.04218604 -0.0418897124 -0.09626402 -0.01503247
## dRel     0.00000000 -0.0001838208 -0.01385361  0.01141869
## dCr1Rel -0.04218604 -0.0416301733 -0.09778504 -0.01323211
##
## , , aar, PD8314a
##
##                 hat      median        lower       upper
## none     0.13298991  0.13096528  0.061187235  0.22304131
## cr1      0.04916117  0.04820070  0.020633949  0.10185429
## rel      0.18686985  0.18489281  0.089794282  0.30353042
## dCr1    -0.08382874 -0.08014001 -0.141322138 -0.03631045
## dRel     0.05387995  0.05032049  0.008146389  0.10664127
## dCr1Rel -0.13770869 -0.13275091 -0.218742006 -0.06246168
```

```
set.seed(42)
multiRFX3TplCi <- MultiRFX3TplCi(coxRFXNrdTD, coxRFXRelTD, coxRFXPrdTD, data=d[,colnames(coxRFXNrdTD$Z)], x=3*365
, nSim=200, prdData=prdData) ## others with 200
dimnames(multiRFX3TplCi)[[4]] <- rownames(dataFrame)
```

The following shows boxplots of the mortality reduction v the risk terciles.

```
par(mar=c(7,5,1,1))
f <- factor(clinicalData$M_Risk, levels=levels(clinicalData$M_Risk)[c(2,4,3,1)])
boxplot(multiRFX3Tpl$CR1 - multiRFX3Tpl$None ~ quantileRiskOsCR[1:1540]  + f, las=2, col=t(outer(riskCol[c(2,4,3,
1)], 2:0, colTrans)), ylab="Survival gain TPL CR1 at 3yr")
```

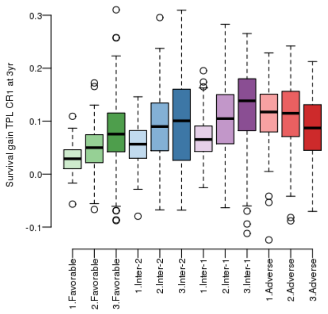

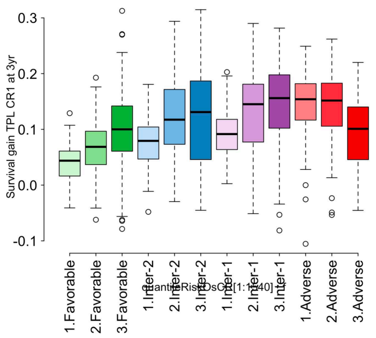

[outputs of the rerun (2021)]

```
boxplot(multiRFX3Tpl$Relapse - multiRFX3Tpl$None ~ quantileRiskOsCR[1:1540]  + f, las=2, col=t(outer(riskCol[c(2,
4,3,1)], 2:0, colTrans)), ylab="Survival gain TPL Relapse at 3yr")
```

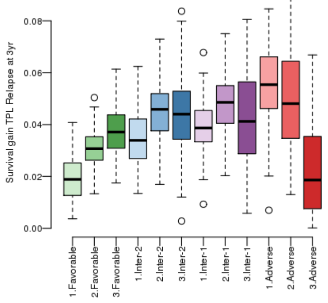

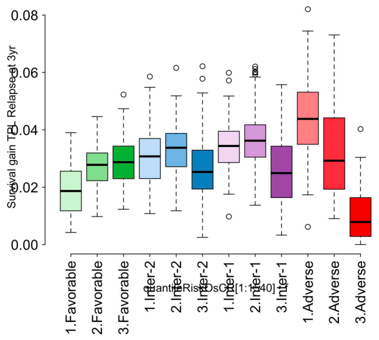

[outputs of the rerun (2021)]

```
boxplot(multiRFX3Tpl$CR1 - multiRFX3Tpl$Relapse ~ quantileRiskOsCR[1:1540] + f, las=2, col=t(outer(riskCol[c(2,4,
3,1)], 2:0, colTrans)), ylab="Survival gain TPL in CR1 over salvage at 3yr")
abline(h=0)
```

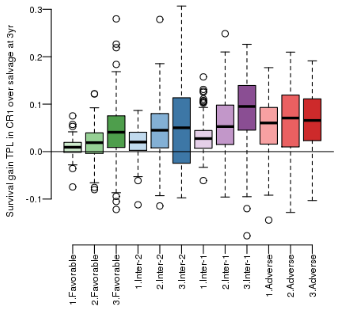

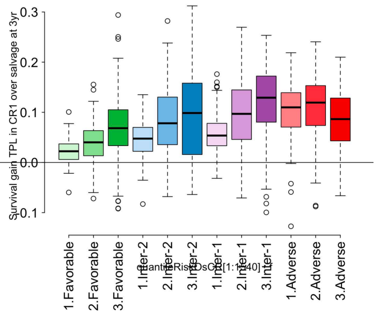

[outputs of the rerun (2021)]

Mortality reduction v age

```
par(c(3,3,1,1))
```

```
## NULL
```

```
y <- multiRFX3Tpl$CR1 - multiRFX3Tpl$None
x <- dataFrame$AOD_10*10
plot(y ~ x)
lines(lowess(x[x<60], y[x<60]), col="green")
```

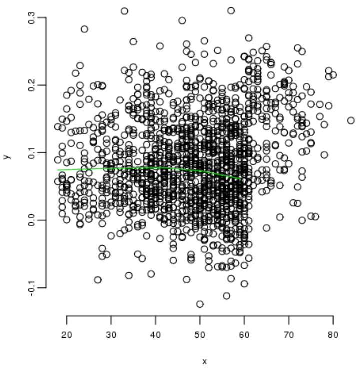

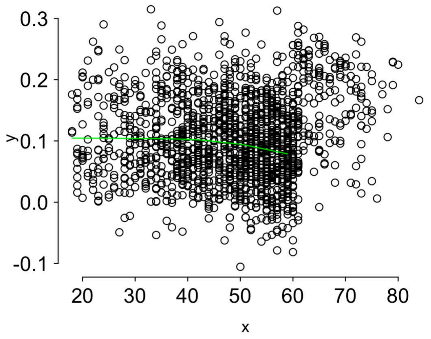

[outputs of the rerun (2021)]

Note: The jump after 60 arises from patients after 60 in AMLHD98B not having received allografts. Based on the trial stratum they are hence (incorrectly) predicted to have very low non-relapse mortality upon allograft. However, this doesn't affect novel patients.

```
plot(multiRFX3Tpl$CR1 - multiRFX3Tpl$None ~ predict(coxRFXOsCR, newdata=osData[1:1540,]), xlab="Risk", ylab="Surv
ival gain TPL CR1 at 1000d")
lines(lowess(predict(coxRFXOsCR, newdata=osData[1:1540,]), multiRFX3Tpl$CR1 - multiRFX3Tpl$None), col='green')
```

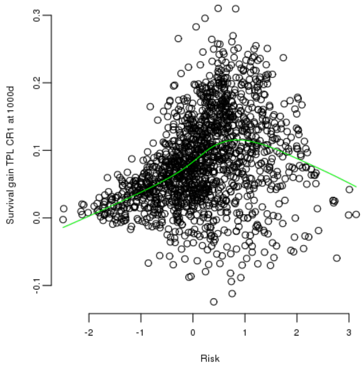

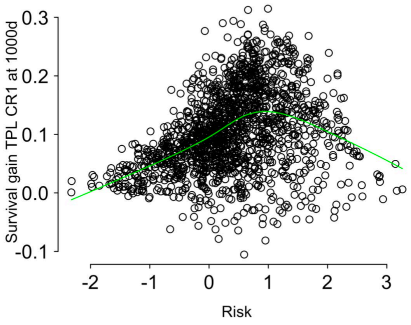

[outputs of the rerun (2021)]

3.6.6.4 Leave one out cross-validation

Codes in the following sections are subject to an LSF environment for parallel computing purposes. We tailor the codes in order to proceed (as shown below). However, since the computations are very intensive, our modifications are liable to errors.

3.6.6.4.1 Three state model

We compute LOO out-of-sample predictions for the survival gain by allograft in CR1 v relapse by training 1540 models on 1539 patients each.

```
multiRFX3TplCiLoo <- sapply(mclapply(rownames(dataFrame), function(pd){
            e <- new.env()
            i <- which(rownames(dataFrame)==pd)
            whichTrain <<- which(rownames(dataFrame)!=pd)
            load(paste0("../code/loo/",i,".RData"), env=e)
            multiRFX3TplCi <- MultiRFX3TplCi(e$rfxNrs, e$rfxRel, e$rfxPrs, data=data[i,colnames(e$rfxPrs$Z), drop
=FALSE], x=3*365, nSim=200, prdData=prdData[prdData$index!=i,], mc.cores=1)
        }, mc.cores=10), I, simplify="array")[,,,1,]
```

This we compare to in-sample predictions of the model trained on all 1540 patients.

```
plot(multiRFX3TplCiLoo["dCr1Rel","hat","os",],multiRFX3TplCi["dCr1Rel","hat","os",] )
```

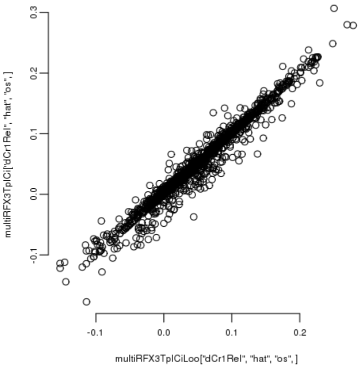


```
# [Modification] multiRFX3TplCiLoo <- sapply(mclapply(rownames(dataFrame), function(pd){
  i <- which(rownames(dataFrame)==pd)
  whichTrain <<- which(rownames(dataFrame)!=pd)
  rfxNrs <- CoxRFX(nrdData[nrdData$index %in% whichTrain, names(crGroups)], Surv(nrdData$time1, nrdData$time2, nrdData$status)[nrdData$index %in% whichTrain], groups=crGroups, which.mu = intersect(mainGroups, unique(crGroups)))
  rfxNrs$coefficients["transplantRel"] <- 0
  rfxPrs <-  CoxRFX(prdData[prdData$index %in% whichTrain, names(crGroups)], Surv(prdData$time1, prdData$time2, prdData$status)[prdData$index %in% whichTrain], groups=crGroups, nu=1, which.mu = intersect(mainGroups, unique(crGroups)))
  rfxRel <-  CoxRFX(relData[relData$index %in% whichTrain, names(crGroups)], Surv(relData$time1, relData$time2, relData$status)[relData$index %in% whichTrain], groups=crGroups, which.mu = intersect(mainGroups, unique(crGroups)))
  rfxRel$coefficients["transplantRel"] <- 0
  multiRFX3TplCi <- MultiRFX3TplCi(rfxNrs, rfxRel, rfxPrs, data=data[i,colnames(rfxPrs$Z), drop=FALSE], x=3*365, nSim=200, prdData=prdData[prdData$index!=i,], mc.cores=1)
}, mc.cores=10), I, simplify="array")[,,,1,]
```

```
cor(multiRFX3TplCiLoo["dCr1Rel","hat","os",], multiRFX3TplCi["dCr1Rel","hat","os",] )
```

```
## [1] 0.9815685
```

```
multiRFX3TplLoo <- t(multiRFX3TplCiLoo[1:3,"hat","os",])
```

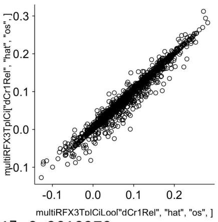


```
[1] 0.9810978
```

[outputs of the rerun (2021)]

The correlation of in-sample and out-of-sample predictions is very high, but there are some differences. We now assess the accuracy of our predictions by comparing the observed survival with the out-of-sample prediction. To this end, we split out the quarter of patients predicted to benefit the most. In both subsets we compare the observed 3yr survial between patients with and without allograft in CR1 and compute the difference. CIs by boostrapping.

```
d <- multiRFX3TplLoo[,2]-multiRFX3TplLoo[,3]
w <- which(clinicalData$AOD < 60)
q <- c(min(d), 0.1, max(d))
c <- cut(d, breaks=q, include.lowest=TRUE)# , paste0("[",names(q)[-length(q)],",",names(q)[-1],")"))
e <- sapply(levels(c),
        function(cc) {
            t <- try(survfit(Surv(time1, time2, status) ~ transplantCR1, data=osData, subset=c[osData$index]==cc & osData$index %in% w));
            if(class(t)=="try-error")
                rep(NA,2)
            else {
                s <- summary(t, time=3*365)
                if(length(s$surv)==2) {
                    ci <- sapply(1:200, function(foo){
                                set.seed(foo)
                                b <- sample(1:nrow(dataFrame), replace=TRUE)
                                diff(summary(survfit(Surv(time1, time2, status) ~ transplantCR1, data=osData, subset=c[osData$index]==cc & osData$index %in% w & osData$index %in% b), time=3*365)$surv)
                            })
                    r <- c(diff(s$surv), quantile(ci, c(0.025, 0.975)))
                    names(r) <- c("delta", "lower",'upper')
                    r
                }
                else rep(NA,3)
            }})
x <- sapply(split(d[w],c[w]),median)
par(xpd=NA, bty="L")
plot(x,e[1,], pch=19, xlim=c(-.05,.2), ylim=c(-.05,.2), xlab = "Predicted survival benefit", ylab="Observed survival benefit (leave-one-out CV)")
h <- density(d[w])
y <- h$y/diff(range(h$y))*.05 + par("usr")[3]
v <- h$x <= q[2]
par(xpd=FALSE)
polygon(c(h$x[v], h$x[which(v)[length(which(v))]]), c(y[v],par("usr")[3]), border=NA, col=set1[1])
polygon(c(h$x[which(!v)[1]], h$x[!v]), c(par("usr")[3],y[!v]), border=NA, col=set1[3])
lines(h$x, y)
segments(x,e[2,],x,e[3,])
#rug(d, col="#00000022")
abline(0,1, lty=3)
```

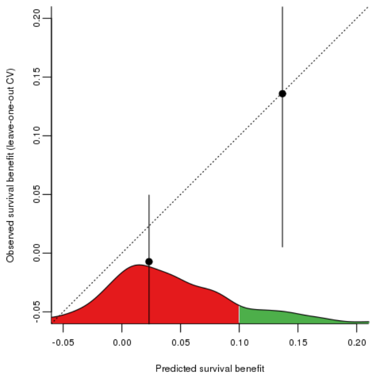

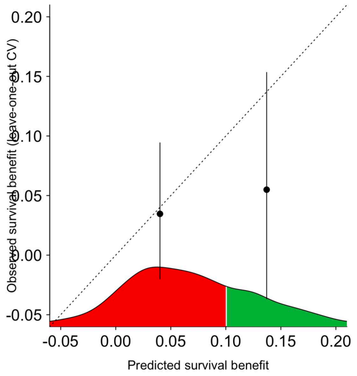

[outputs of the rerun (2021)]

3.6.6.5 Best treatment options

We can explore the the hypothetical survival gain if each patient had received the optimal treatment strategy. First, observed outcome for patients < 60yr with CR

```
summary(survfit(Surv(time1, time2, status) ~ 1, data=osData, subset=AOD_10 < 6), time=3*365)
```

```
## Call: survfit(formula = Surv(time1, time2, status) ~ 1, data = osData,
##     subset = AOD_10 < 6)
##
## 176 observations deleted due to missingness
##  time n.risk n.event survival std.err lower 95% CI upper 95% CI
##  1095    605     455    0.584  0.0149        0.555        0.614
```

Compared to predictions, as treated

```
mean(sapply((1:nrow(data))[!is.na(clinicalData$CR_date) & clinicalData$AOD < 60], function(i) multiRFX3TplLoo[i,
1+data[i,"transplantCR1"] + 2*data[i, "transplantRel"] ]))
```

```
## [1] 0.5865632
```

The observed outcome and predictions are consistent. Now explore what would have been the best option for each patient:

```
apply(apply(-multiRFX3TplLoo[!is.na(clinicalData$CR_date) & clinicalData$AOD < 60,],1,rank),1,function(x) table(f
actor(x, levels=1:3)))
```

```
##   none cr1 rel
## 1    0 861 248
## 2   92 156 861
## 3 1017  92   0
```

The same as above, split by ELN risk categories:

```
table(clinicalData$M_Risk[!is.na(clinicalData$CR_date) & clinicalData$AOD < 60], factor(apply(multiRFX3TplLoo[!is
.na(clinicalData$CR_date) & clinicalData$AOD < 60,], 1, which.ma
)[c(2,4,3,1),]
```

```
##
##              none cr1 rel
##   Favorable     0 276 120
##   Inter-2       0 137  49
##   Inter-1       0 245  51
##   Adverse       0 121  24
```

Split by ELN risk, requiring TPL in CR1 to offer 5% advantage over salvage

```
table(clinicalData$M_Risk, apply(multiRFX3TplLoo, 1, function(x)
```

```
##
##              FALSE TRUE
##   Favorable    376   97
##   Inter-2      167  101
##   Inter-1      210  207
##   Adverse      101  152
```

Compute the extrapolated survival under each of the different scenarios:

```
colMeans(multiRFX3TplLoo[!is.na(clinicalData$CR_date) & clinical
```

```
##      none       cr1       rel
## 0.5546881 0.6305105 0.5920412
```

Best possible - everyone had received the optimal strategy:

```
> mean(sapply((1:nrow(data))[!is.na(clinicalData$CR_date
  1+data[i,"transplantCR1"] + 2*data[i, "transplantRel"]
[1] 0.561496
> # The observed outcome and predictions are consistent.
  patient:
> apply(apply(-multiRFX3TplLoo[!is.na(clinicalData$CR_da
actor(x, levels=1:3)))
  none cr1 rel
1    0 998 111
2   47  64 998
3 1062  47   0
> # The same as above, split by ELN risk categories:
> table(clinicalData$M_Risk[!is.na(clinicalData$CR_date)
s.na(clinicalData$CR_date) & clinicalData$AOD < 60,], 1,
[c(2,4,3,1),]

               none cr1 rel
  Favorable    0 336  60
  Inter-2      0 164  22
  Inter-1      0 277  19
  Adverse      0 137   8
> # Split by ELN risk, requiring TPL in CR1 to offer 5%
> table(clinicalData$M_Risk, apply(multiRFX3TplLoo, 1, f

               FALSE TRUE
  Favorable     287  186
  Inter-2       103  165
  Inter-1       118  299
  Adverse        57  196
> # Compute the extrapolated survival under each of the
> colMeans(multiRFX3TplLoo[!is.na(clinicalData$CR_date)
     none       cr1       rel
0.5238456 0.6214604 0.5532709
> # Best possible - everyone had received the optimal s
> mean(apply(multiRFX3TplLoo[!is.na(clinicalData$CR_date
[1] 0.6240944
```

[outputs of the rerun (2021)]

```
mean(apply(multiRFX3TplLoo[!is.na(clinicalData$CR_date) & clinicalData$AOD < 60,],1,max))
```

```
## [1] 0.6368276
```

3.6.6.6 Three patients with numerical CI's and LOO

```
patients <- c("PD11104a","PD8314a","PD10941a")
threePatientTplCiLoo <- sapply(patients, function(pd){
            e <- new.env()
            i <- which(rownames(dataFrame)==pd)
            whichTrain <<- which(rownames(dataFrame)!=pd)
            load(paste0("../code/loo/",i,".RData"), env=e)
            multiRFX3TplCi <- MultiRFX3TplCi(e$rfxNrs, e$rfxRel, e$rfxPrs, data=data[i,colnames(e$rfxPrs$Z), drop
=FALSE], x=3*365, nSim=1000, prdData=prdData[prdData$index!=i,], mc.cores=5)
        }, simplify="array")
```

We modify this function the same way (with the same commands altered as in section 3.6.6.4.1) in order to proceed

3.6.6.7 LOO predictions of HSCT with CI's accounting for correlation

The following code is run on the cluster

Section 3.6.6.7 calculates the confidence interval for predictions, which was originally run in parallel on an LSF platform. We decided to skip this section as the computations are very intensive, and thus the modifications are prone to errors

```
nSim <- 200
read_chunk('../code/ciCor.R', labels="ciCor")
```

```
load("ciCor.RData")

library(mg14)
library(CoxHD)
library(Rcpp)

#save(allDataTpl, coxRFXNrdTD, coxRFXPrdTD, coxRFXRelTD, MultiRFX3, prdData, relData, nrdData, crGroups, nSim, fi
le="../code/ciCor.RData")

cppFunction('NumericVector computeTotalPrsC(NumericVector x, NumericVector diffCir, NumericVector prsP, NumericVe
ctor tdPrmBaseline, double risk) {
                int xLen = x.size();
                double hj;
                double r = exp(risk);
                NumericVector rs(xLen);
                for(int i = 0; i < xLen; ++i) rs[i] = 1;
                for(int j = 1; j < xLen; ++j){
                hj = tdPrmBaseline[j-1] * r;
                for(int i = j; i < xLen; ++i){
                rs[i] += diffCir[j-1] * (1-pow(prsP[i-j], hj));
                }
                }
                return rs;
                }', rebuild=TRUE)

jobIndex <- as.numeric(Sys.getenv("LSB_JOBINDEX"))

load(paste0("loo/",jobIndex,".RData"))

cvIdx <- 1:nrow(dataFrame)
whichTrain <- which(cvIdx != jobIndex)

multiRFX3TplCiCorLoo <- sapply(1:nSim, function(foo){
            set.seed(foo)
            cNrd <- rfxNrs
            cNrd$coefficients <- mvtnorm::rmvnorm(1, mean=cNrd$coefficients, sigma=coxRFXNrdTD$var2, method="chol
")[1,]
            cRel <- rfxRel
            cRel$coefficients <- mvtnorm::rmvnorm(1, mean=cRel$coefficients, sigma=coxRFXRelTD$var2, method="chol
")[1,]
            cPrd <- rfxPrs
            cPrd$coefficients <- mvtnorm::rmvnorm(1, mean=cPrd$coefficients, sigma=coxRFXPrdTD$var2, method="chol
")[1,]
            multiRFX3Tpl <- as.matrix(MultiRFX3(cNrd, cRel, cPrd, data=allDataTpl[3*jobIndex + (-2:0),], x=3*365,
prdData=prdData[prdData$index %in% whichTrain,])[,c("os","cir","nrs","rs")])
            rownames(multiRFX3Tpl) <- c("None","CR1","Relapse")
            return(multiRFX3Tpl)
        }, simplify='array')

save(multiRFX3TplCiCorLoo, file=paste0("ciCorLoo/",jobIndex,".RData"))
```

Collect the results

```
multiRFX3TplCiCorLoo <- simplify2array(mclapply(1:nrow(dataFrame), function(foo) try({
                                e <- new.env()
                                load(paste0("../code/ciCorLoo/",foo,".RData"), envir=e)
                                return(e$multiRFX3TplCiCorLoo)
                            }), mc.cores=4))
```

3.6.6.8 Figure 5A

The figure shows the mortality reduction of allograft CR1 v none, allograft in Rel v none, and CR1 v Relapse, for LOO predictions similar to above.

```
par(mar=c(3,3,1,3), las=2, mgp=c(2,.5,0), bty="n")
benefit <- multiRFX3TplLoo[,2]-multiRFX3TplLoo[,3]
benefitGroup <- factor(benefit > 0.1, labels=c("Low","High"))
absrisk <- multiRFX3TplLoo[,1]
names(absrisk) <- names(benefit) <- rownames(dataFrame)
s <- clinicalData$AOD < 60 & !is.na(clinicalData$CR_date) &! clinicalData$TPL_Phase %in% c("RD1","PR1")
x <- 1-absrisk
y <- benefit
plot(x[s], y[s], pch=NA, ylab="Mortality reduction from allograft", xlab="3yr mortality with standard chemo", col
=riskCol[clinicalData$M_Risk], cex=.8, las=1, ylim=range(benefit))
abline(h=seq(-.1,.3,.1), col='grey', lty=3)
abline(v=seq(.2,.9,0.2), col='grey', lty=3)
points(x[s], y[s], pch=16,  col=riskCol[clinicalData$M_Risk[s]], cex=.8)
segments(1-threePatientTplCiLoo["none","lower","os",1,patients], y[patients],1-threePatientTplCiLoo["none","upper
","os",1,patients],y[patients])
segments(x[patients], threePatientTplCiLoo["dCr1Rel","lower","os",1,patients],x[patients], threePatientTplCiLoo["
dCr1Rel","upper","os",1,patients])
# Add loess fit, accounting for correlations of errors
xn <- seq(0.01,0.99,0.01)
fit <- sapply(1:nSim, function(i){
            benefit <- multiRFX3TplCiCorLoo[2,"os",i,]-multiRFX3TplCiCorLoo[3,"os",i,]
            absrisk <- multiRFX3TplCiCorLoo[1,"os",i,]
            s <- clinicalData$AOD < 60 & !is.na(clinicalData$CR_date) &! clinicalData$TPL_Phase %in% c("RD1","PR1
")
            x <- 1-absrisk
            y <- benefit
            p <- predict(loess(y~x, data=data.frame(x=x[s], y=y[s])), newdata=data.frame(x=xn), se=TRUE)
            yn <- c(p$fit + 2*p$se.fit,rev(p$fit - 2*p$se.fit))
            p$fit
        })
q <- apply(fit, 1, quantile, c(0.025, 0.5, 0.975), na.rm=TRUE)
polygon(c(xn, rev(xn)), c(q[1,], rev(q[3,])), border=NA, col="#00000044", lwd=1)
lines(xn, rowMeans(fit, na.rm=TRUE))
legend("topleft", pch=c(16,16,16,16,NA),lty=c(NA,NA,NA,NA,1), col=c(riskCol[c(2,4,3,1)],1),fill=c(NA,NA,NA,NA,"gr
ey"), border=NA, c(names(riskCol)[c(2,4,3,1)],"loess average"), box.lty=0)
n <- c(100,50,20,10,5,4,3)
axis(side=4, at=1/n, labels=n, las=1)
mtext("Number needed to treat", side=4, at=.2, line=2, las=0)
axis(side=4, at=-1/n, labels=n, las=1)
mtext("Number needed to harm", side=4, at=-.1, line=2, las=0)
```

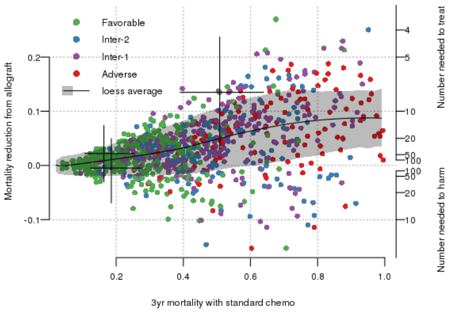

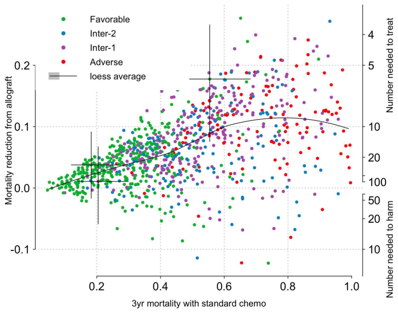

Confidence interval (grey area) is not shown here, as the computations on the cluster have been skipped [outputs of the rerun (2021)]

3.6.6.9 Figure 5B

Mosaic plot of benefit v eln risk category

```
benefit4 <- cut(benefit, c(-Inf, 0, .05, .1, Inf), labels=c("<0%", "0-5%","5-10%",">10%"))
e <- factor(paste(clinicalData$M_Risk), levels=rev(c("NA","Adverse","Inter-1","Inter-2","Favorable")))
w <- clinicalData$AOD < 60 & !is.na(clinicalData$CR_date) & !clinicalData$TPL_Phase %in% c("PR1","RD1")
mosaicplot(table(`ELN risk group`=e[w],`Mortality Reduction`=benefit4[w])[5:1,4:1], col=RColorBrewer::brewer.pal(
7,"RdBu")[6:3], main="")
```

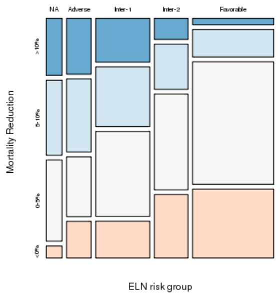

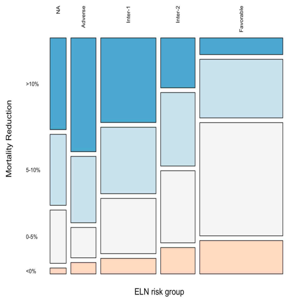

[outputs of the rerun (2021)]

Violins plot of the predicted survival gain

```
par(mar=c(3,3,1,1), mgp=c(2,0.5,0), bty="n")
h <- density(benefit[s])
y <- h$y/diff(range(h$y))*.05 + par("usr")[3]
par(xpd=FALSE)
xx <- c(h$x, rev(h$x))
yy <- c(h$y, -rev(h$y))
v <- xx <= 0.1 #q[2]
plot(yy,xx, pch=NA, ylab="Predicted benefit", xlab="", xaxt="n", ylim=range(benefit))
polygon(yy[v], xx[v], border=NA, col=set1[1])
polygon(yy[!v], xx[!v], border=NA, col=set1[2])
lines(yy, xx)
```

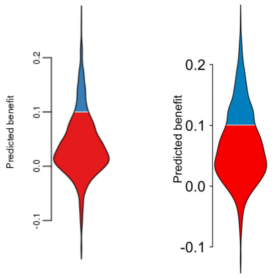

[outputs of the rerun (2021)]

3.6.6.10 Figure 5C

KM plot of the high v low benefit groups

```
par(mar=c(3,3,1,1), mgp=c(2,0.5,0), bty="L")
f <- survfit(Surv(time1/365, time2/365, status) ~ group +  transplantCR1, data=cbind(osData, group=benefitGroup[o
sData$index]), subset=osData$index %in% which(s) & !clinicalData$M_Risk[osData$index] %in% c("Favorable"))
summary(f, time=3)
```

```
## Call: survfit(formula = Surv(time1/365, time2/365, status) ~ group +
##     transplantCR1, data = cbind(osData, group = benefitGroup[osData$index]),
##     subset = osData$index %in% which(s) & !clinicalData$M_Risk[osData$index] %in%
##         c("Favorable"))
##
##                 group=Low, transplantCR1=0
##         time       n.risk       n.event      survival      std.err lower 95% CI upper 95% CI
##        3.000      133.000       142.000         0.506        0.030        0.450        0.568
##
##                 group=Low, transplantCR1=1
##         time       n.risk       n.event      survival      std.err lower 95% CI upper 95% CI
##       3.0000     119.0000       86.0000        0.5499       0.0386       0.4792       0.6310
##
##                 group=High, transplantCR1=0
##         time       n.risk       n.event      survival      std.err lower 95% CI upper 95% CI
##       3.0000      14.0000       44.0000        0.2722       0.0594       0.1775       0.4174
##
##                 group=High, transplantCR1=1
##         time       n.risk       n.event      survival      std.err lower 95% CI upper 95% CI
##       3.0000      21.0000       32.0000        0.3955       0.0671       0.2835       0.5516
```

```
plot(f, col=rep(pastel1[1:nlevels(benefitGroup)],each=2), lty=rep(c(1,2), nlevels(benefitGroup)), xlab="TIme afte
r CR", ylab="Survival", xlim=c(0,5), cex=.5)
t <- table(which(s) %in% osData$index[osData$transplantCR1==1],benefitGroup[s],!is.na(clinicalData$CR_date[s]), !
clinicalData$M_Risk[s] %in% c("Favorable"))[,,"TRUE","TRUE"]
legend("topright", legend=paste(rep(levels(benefitGroup), each=2), rep(c("no HSCT","w. HSCT"), 2), as.numeric(t),
sep=", "), col=rep(pastel1[1:nlevels(benefitGroup)],each=2), lty=rep(c(1,2), nlevels(benefitGroup)), bty="n")
```

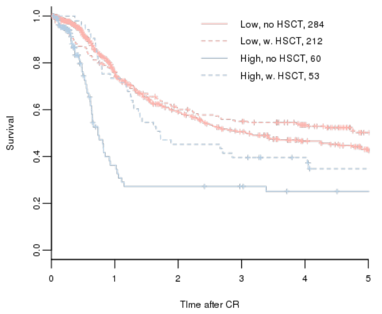


```
Call: survfit(formula = Surv(time1/365, time2/365, status) ~ group +
    transplantCR1, data = cbind(osData, group = benefitGroup[osData$index]),
    subset = osData$index %in% which(s) & !clinicalData$M_Risk[osData$index] %in%
        c("Favorable"))

                group=Low, transplantCR1=0
        time       n.risk       n.event      survival      std.err lower 95% CI upper 95% CI
      3.0000     116.0000      107.0000        0.5407       0.0331       0.4795       0.6097

                group=Low, transplantCR1=1
        time       n.risk       n.event      survival      std.err lower 95% CI upper 95% CI
      3.0000      97.0000       54.0000        0.6249       0.0404       0.5505       0.7094

                group=High, transplantCR1=0
        time       n.risk       n.event      survival      std.err lower 95% CI upper 95% CI
      3.0000      31.0000       79.0000        0.3157       0.0446       0.2393       0.4165

                group=High, transplantCR1=1
        time       n.risk       n.event      survival      std.err lower 95% CI upper 95% CI
      3.0000      43.0000       64.0000        0.3788       0.0509       0.2912       0.4929
```

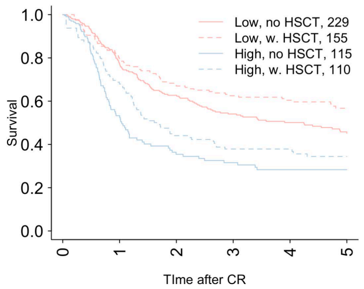

[outputs of the rerun (2021)]

3.6.6.11 Supplementary Figure S3

KM plot of the high v low benefit groups

```
par(mar=c(3,3,2,1), mgp=c(2,0.5,0), bty="L", mfrow=c(3,2), cex=1)
e <- factor(paste(clinicalData$M_Risk))
for(l in levels(e)){
    f <- survfit(Surv(time1/365, time2/365, status) ~ group +  transplantCR1, data=cbind(osData, group=benefitGro
up[osData$index]), subset=osData$index %in% which(s) & e[osData$index] == l)
    summary(f, time=3)
    plot(f, col=rep(pastel1[1:nlevels(benefitGroup)],each=2), lty=rep(c(1,2), nlevels(benefitGroup)), xlab="TIme
after CR", ylab="Survival", xlim=c(0,5), cex=.5, main=l)
    t <- table(which(s) %in% osData$index[osData$transplantCR1==1],benefitGroup[s],!is.na(clinicalData$CR_date[s]
), e[s])[,,"TRUE",l]
    legend("topright", legend=paste(rep(levels(benefitGroup), each=2), rep(c("no HSCT","w. HSCT"), 2), as.numeric
(t), sep=", "), col=rep(pastel1[1:nlevels(benefitGroup)],each=2), lty=rep(c(1,2), nlevels(benefitGroup)), bty="n"
)
}
```

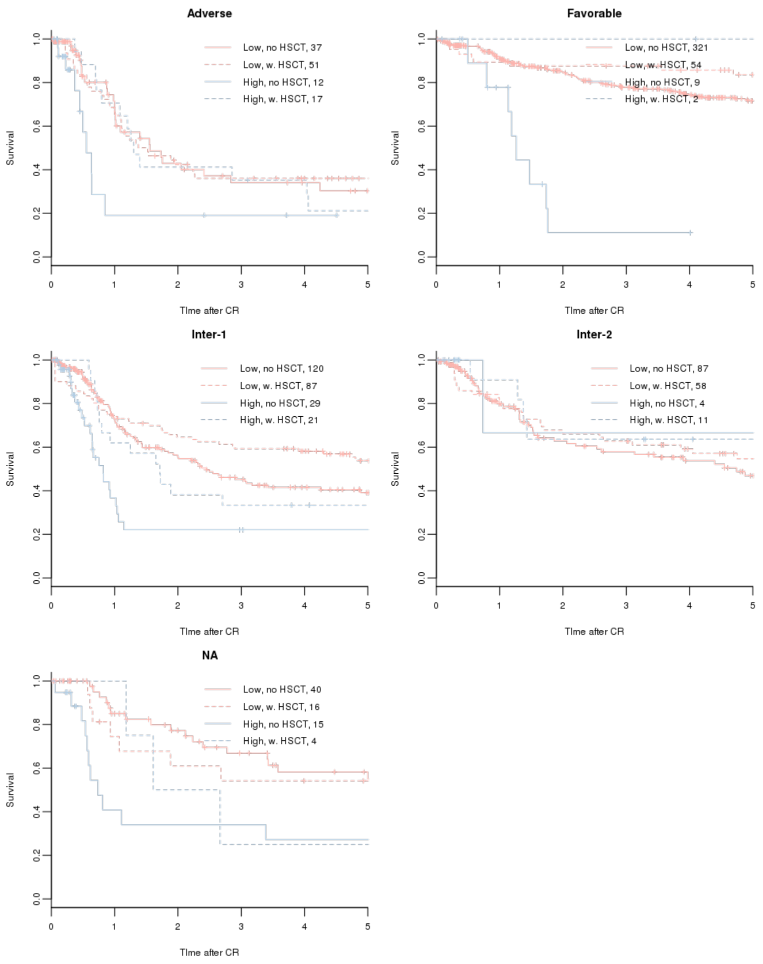

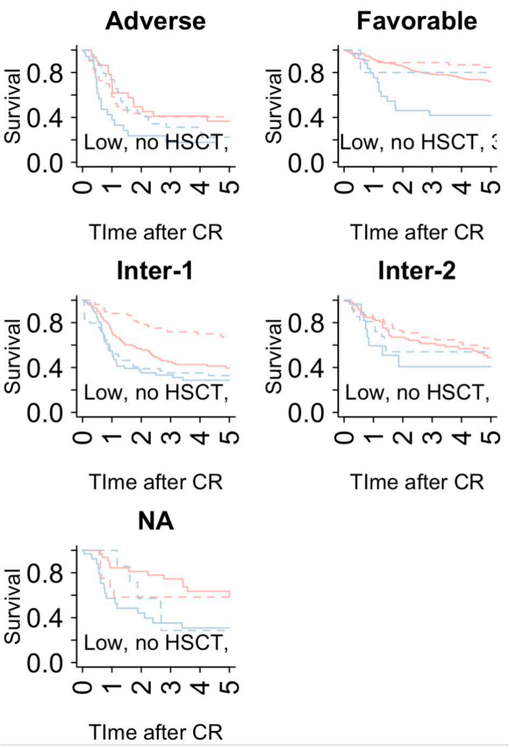

[outputs of the rerun (2021)]

3.6.6.12 Figure 5D

The following plot shows the hypothetical population-level survival gains of the knowledge bank.

```
par(bty="L")
s <- clinicalData$AOD < 60 & !is.na(clinicalData$CR_date) & !clinicalData$TPL_Phase %in% c("PR1","RD1")
fAlloRelapse <- sum(prdData$transplantRel & s[!is.na(clinicalData$Recurrence_date)][prdData$index])/sum(relData$s
tatus & !relData$transplantCR1 & s[relData$index]) # fraction of patients that have received a salvage transplant
benefitAllo <- multiRFX3TplLoo[,"cr1"] - (fAlloRelapse*multiRFX3TplLoo[,"rel"] +(1-fAlloRelapse)*multiRFX3TplLoo[
,"none"])
o <- order(-benefitAllo + ifelse(!s ,NA,0), na.last=NA)
pRelapse <- 1+multiRFX3TplCiLoo[1:2,1,"aar",] - multiRFX3TplCiLoo[1:2,1,"rs",] ## Relapse probabilities
fRelapse <- sapply(seq_along(o), function(i) mean(c(pRelapse[2,o[1:i]], pRelapse[1,o[-(1:i)]]), na.rm=TRUE)) # Pe
rsonalised

sIdeal <- sapply(seq_along(o), function(i) mean(c(multiRFX3TplLoo[o[1:i],"cr1"], (1-fAlloRelapse)*multiRFX3TplLoo
[o[-(1:i)],"none"] + fAlloRelapse*multiRFX3TplLoo[o[-(1:i)],"rel"]), na.rm=TRUE))
x <- seq_along(sIdeal)/length(sIdeal)
plot(x + (1-x)*fRelapse*fAlloRelapse,sIdeal, type='l', xlab="Total fraction of allografts", ylab="Survival of eli
gible patients 3yrs after CR", col=set1[1], xaxs="i", yaxs="i", lty=1)

p <- order(na.zero(c(1,4,2,3)[clinicalData$M_Risk])  + dataFrame$AOD_10/20 + ifelse(!s,NA,0) + ifelse(clinicalDat
a$AOD>=60,NA,0), na.last=NA)
fRelapseEln <- sapply(seq_along(p), function(i) mean(c(pRelapse[2,p[1:i]], pRelapse[1,p[-(1:i)]]), na.rm=TRUE)) #
ELN
sEln <- sapply(seq_along(p), function(i) mean(c(multiRFX3TplLoo[p[1:i],"cr1"], (1-fAlloRelapse)*multiRFX3TplLoo[p
[-(1:i)],"none"] + fAlloRelapse*multiRFX3TplLoo[p[-(1:i)],"rel"]), na.rm=TRUE))
x <- seq_along(sEln)/length(sEln)

lines(x + (1-x)*fRelapseEln*fAlloRelapse,sEln, sEln, type='l', col=set1[2])
legend("bottomright", c("Priorisation based on", "Knowledge bank", "ELN and age"),  col=set1[c(NA,1,2)],lty=c(NA,
1,1), bty="n", text.font=c(2,1,1))
```

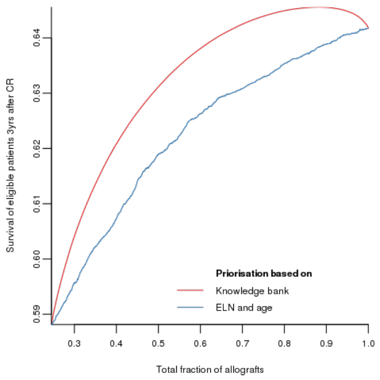

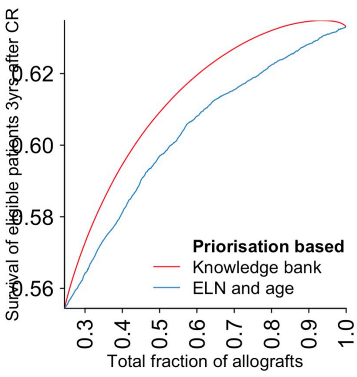

[outputs of the rerun (2021)]

Total numbers of transplants

```
fAlloCR1 <- 0.3 ## Assume 30% allografts in CR1
i <- which(x > fAlloCR1)[1] - 1
c(`Knowlege bank`=(x + (1-x)*fRelapse*fAlloRelapse)[i], ELN=(x + (1-x)*fRelapseEln*fAlloRelapse)[i])
```

```
## Knowlege bank            ELN
##     0.4358391     0.4380818
```

```
> c(`Knowlege bank`=(x + (1-x)*fl
n*fAlloRelapse)[i])
Knowlege bank           ELN
    0.4351103     0.4380676
> #' Projected survival at 3yrs
> c(ELN=sEln[i], `Knowledge bank
          ELN Knowledge bank
    0.5874657      0.6003123
> #' Achieve same survival as EL
> j <- c(`Knowledge bank`=which(
> fAlloCR1Pers <- (x + (1-x)*fRe
> names(fAlloCR1Pers) <- names(j
> fAlloCR1Pers
Knowledge bank
     0.3625197
```

Projected survival at 3yrs

```
c(ELN=sEln[i], `Knowledge bank`=sIdeal[i])
```

```
##           ELN Knowledge bank
##     0.6117393      0.6250449
```

Achieve same survival as ELN with the following number of allografts

[outputs of the rerun (2021)]

```
j <- c(`Knowledge bank`=which(sIdeal >= sEln[i])[1]-1)
fAlloCR1Pers <- (x + (1-x)*fRelapse*fAlloRelapse)[j]
names(fAlloCR1Pers) <- names(j)
fAlloCR1Pers
```

```
## Knowledge bank
##      0.3392658
```

3.6.6.13 Supplementary Figure S4

As there is some uncertainty related to the overall benefit of early vs late allografts, the following plots show the benefit at the extremes of the expected distribution. Plots are shown for the 5%, 50% and 95% quantiles.

```
par(bty="L")

benefitCiLoo <- multiRFX3TplCiCorLoo[3,"os",,] - multiRFX3TplCiCorLoo[2,"os",,]
r <- rank(rowMeans(benefitCiLoo))

s <- clinicalData$AOD < 60 & !is.na(clinicalData$CR_date) & !clinicalData$TPL_Phase %in% c("PR1","RD1")

for(q in c(10, 50, 190)){
    w <- which(r==q)
    fAlloRelapse <- sum(prdData$transplantRel & s[!is.na(clinicalData$Recurrence_date)][prdData$index])/sum(relData$status & !relData$transplantCR1 & s[relData$index]) # fraction of patients that have received a salvage transplant
    o <- order(-benefitAllo + ifelse(!s,NA,0), na.last=NA)
    pRelapse <-  1-multiRFX3TplCiCorLoo[,"cir",w,] ## Relapse probabilities
    fRelapse <- sapply(seq_along(o), function(i) mean(c(pRelapse[2,o[1:i]], pRelapse[1,o[-(1:i)]]), na.rm=TRUE)) # Personalised

    sIdeal <- sapply(seq_along(o), function(i) mean(c(multiRFX3TplCiCorLoo["CR1","os",w,o[1:i]], (1-fAlloRelapse)*multiRFX3TplCiCorLoo["None","os",w,o[-(1:i)]] + fAlloRelapse*multiRFX3TplCiCorLoo["Relapse","os",w, o[-(1:i)]]), na.rm=TRUE))
    x <- seq_along(sIdeal)/length(sIdeal)
    plot(x + (1-x)*fRelapse*fAlloRelapse,sIdeal, type='l', xlab="Total fraction of allografts", ylab="Survival of eligible patients 3yrs after CR", col=set1[1], xaxs="i", yaxs="i", lty=1, ylim=c(0.59, 0.67))

    p <- order(na.zero(c(1,4,2,3)[clinicalData$M_Risk])  + dataFrame$AOD_10/20 + ifelse(!s,NA,0) + ifelse(!s,NA,0), na.last=NA)
    fRelapseEln <- sapply(seq_along(p), function(i) mean(c(pRelapse[2,p[1:i]], pRelapse[1,p[-(1:i)]]), na.rm=TRUE)) # ELN
    sEln <- sapply(seq_along(p), function(i) mean(c(multiRFX3TplCiCorLoo["CR1","os",w,p[1:i]], (1-fAlloRelapse)*multiRFX3TplCiCorLoo["None","os",w,p[-(1:i)]] + fAlloRelapse*multiRFX3TplCiCorLoo["Relapse","os",w, p[-(1:i)]]), na.rm=TRUE))

    x <- seq_along(sEln)/length(sEln)

    lines(x + (1-x)*fRelapseEln*fAlloRelapse,sEln, sEln, type='l', col=set1[2])
    legend("bottomright", c("Priorisation based on", "Knowledge bank", "ELN and age"),  col=set1[c(NA,1,2)],lty=c(NA,1,1), bty="n", text.font=c(2,1,1))
}
```

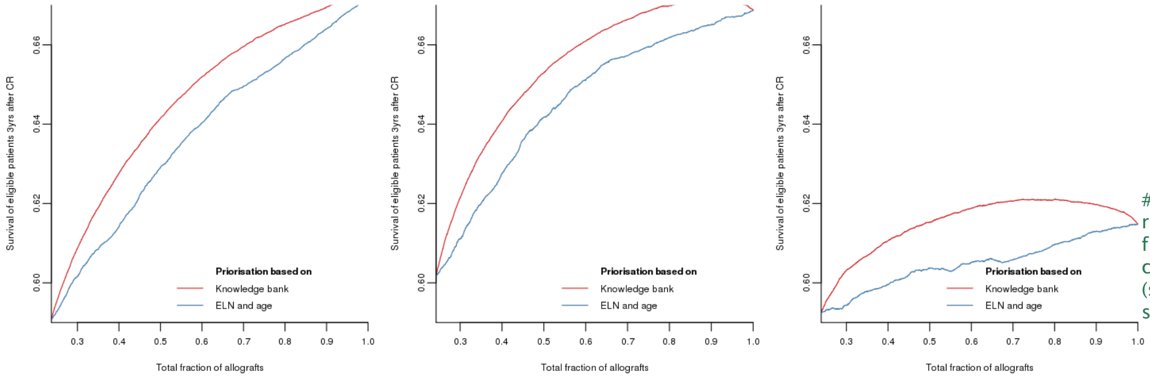

Here we cannot reproduce these three figures as the computations for CI (section 3.6.6.7) were skipped as we noted.

The bottom line is that we are able to confidently isolate a quarter of patients with high benefit of allografts (about 12% absolute benefit). The breakdown across ELN risk groups is:

```
table(benefitGroup[s], paste(clinicalData$M_Risk[s]), allograft=data$transplantCR1[s])
```

```
## , , allograft = 0
## 
##       
##        Adverse Favorable Inter-1 Inter-2  NA
##   Low       37       321     120      87  40
##   High      12         9      29       4  15
## 
## , , allograft = 1
## 
##       
##        Adverse Favorable Inter-1 Inter-2  NA
##   Low       51        54      87      58  16
##   High      17         2      21      11   4
```

```
> table(benefitGroup[s], paste(clinicalData$M_Risk[s]), allograft=data$transplantCR1[s])
, , allograft = 0

      
       Adverse Favorable Inter-1 Inter-2  NA
  Low       25       306      96      76  32
  High      24        24      53      15  23

, , allograft = 1

      
       Adverse Favorable Inter-1 Inter-2  NA
  Low       32        51      63      48  12
  High      36         5      45      21   8
```

[outputs of the rerun (2021)]

```
summary(coxph(Surv(time1/365, time2/365, status) ~ transplantCR1 + AOD_10 + clinicalData$M_Risk[osData$index] , data=osData, subset=osData$index %in% which(s) & benefitGroup[osData$index]=="Low"))
```

```
## Call:
## coxph(formula = Surv(time1/365, time2/365, status) ~ transplantCR1 +
##     AOD_10 + clinicalData$M_Risk[osData$index], data = osData,
##     subset = osData$index %in% which(s) & benefitGroup[osData$index] ==
##         "Low")
##
##   n= 1225, number of events= 342
##    (90 observations deleted due to missingness)
##
##                                               coef exp(coef) se(coef)      z Pr(>|z|)
## transplantCR1                             -0.30067   0.74032  0.12538 -2.398 0.016483 *
## AOD_10                                     0.24248   1.27440  0.05606  4.326 1.52e-05 ***
## clinicalData$M_Risk[osData$index]Favorable -1.41836   0.24211  0.17131 -8.279  < 2e-16 ***
## clinicalData$M_Risk[osData$index]Inter-1   -0.47766   0.62023  0.16191 -2.950 0.003176 **
## clinicalData$M_Risk[osData$index]Inter-2   -0.65588   0.51899  0.17808 -3.683 0.000231 ***
## ---
## Signif. codes:  0 '***' 0.001 '**' 0.01 '*' 0.05 '.' 0.1 ' ' 1
##
##                                            exp(coef) exp(-coef) lower .95 upper .95
## transplantCR1                                 0.7403     1.3508    0.5790    0.9466
## AOD_10                                        1.2744     0.7847    1.1418    1.4224
## clinicalData$M_Risk[osData$index]Favorable    0.2421     4.1303    0.1731    0.3387
## clinicalData$M_Risk[osData$index]Inter-1      0.6202     1.6123    0.4516    0.8519
## clinicalData$M_Risk[osData$index]Inter-2      0.5190     1.9268    0.3661    0.7358
##
## Concordance= 0.666  (se = 0.016 )
## Rsquare= 0.087   (max possible= 0.971 )
## Likelihood ratio test= 111.5  on 5 df,   p=0
## Wald test            = 110.2  on 5 df,   p=0
## Score (logrank) test = 118.8  on 5 df,   p=0
```

```
> summary(coxph(Surv(time1/365, time2/365, status) ~ transplantCR1 + AOD_10 + clinicalData$M_R
isk[osData$index] , data=osData, subset=osData$index %in% which(s) & benefitGroup[osData$inde
x]=="Low"))
Call:
coxph(formula = Surv(time1/365, time2/365, status) ~ transplantCR1 +
    AOD_10 + clinicalData$M_Risk[osData$index], data = osData,
    subset = osData$index %in% which(s) & benefitGroup[osData$index] ==
        "Low")

  n= 1031, number of events= 270
   (68 observations deleted due to missingness)

                                               coef exp(coef) se(coef)      z Pr(>|z|)
transplantCR1                              -0.39269   0.67524  0.14743 -2.664  0.00773 **
AOD_10                                      0.31693   1.37291  0.06585  4.813 1.49e-06 ***
clinicalData$M_Risk[osData$index]Favorable -1.28167   0.27757  0.20257 -6.327 2.50e-10 ***
clinicalData$M_Risk[osData$index]Inter-1   -0.38533   0.68022  0.20045 -1.922  0.05457 .
clinicalData$M_Risk[osData$index]Inter-2   -0.60121   0.54815  0.21398 -2.810  0.00496 **
---
Signif. codes:  0 '***' 0.001 '**' 0.01 '*' 0.05 '.' 0.1 ' ' 1

                                           exp(coef) exp(-coef) lower .95 upper .95
transplantCR1                                 0.6752     1.4810    0.5058    0.9015
AOD_10                                        1.3729     0.7284    1.2067    1.5620
clinicalData$M_Risk[osData$index]Favorable    0.2776     3.6026    0.1866    0.4129
clinicalData$M_Risk[osData$index]Inter-1      0.6802     1.4701    0.4592    1.0076
clinicalData$M_Risk[osData$index]Inter-2      0.5481     1.8243    0.3604    0.8338

Concordance= 0.673  (se = 0.017 )
Likelihood ratio test= 92.77  on 5 df,   p=<2e-16
Wald test            = 92.5  on 5 df,   p=<2e-16
Score (logrank) test = 98.33  on 5 df,   p=<2e-16
```

[outputs of the rerun (2021)]

```
summary(coxph(Surv(time1/365, time2/365, status) ~ transplantCR1 + AOD_10 + clinicalData$M_Risk[osData$index] , d
ata=osData, subset=osData$index %in% which(s) & benefitGroup[osData$index]=="High"))
```

```
## Call:
## coxph(formula = Surv(time1/365, time2/365, status) ~ transplantCR1 +
##     AOD_10 + clinicalData$M_Risk[osData$index], data = osData,
##     subset = osData$index %in% which(s) & benefitGroup[osData$index] ==
##         "High")
##
##   n= 174, number of events= 73
##    (25 observations deleted due to missingness)
##
##                                              coef exp(coef) se(coef)      z Pr(>|z|)
## transplantCR1                             -0.7061    0.4936   0.2569 -2.749  0.00599 **
## AOD_10                                     0.0705    1.0731   0.1187  0.594  0.55235
## clinicalData$M_Risk[osData$index]Favorable -0.8071    0.4461   0.4328 -1.865  0.06222 .
## clinicalData$M_Risk[osData$index]Inter-1   -0.2869    0.7506   0.2733 -1.050  0.29377
## clinicalData$M_Risk[osData$index]Inter-2   -1.3228    0.2664   0.4965 -2.664  0.00771 **
## ---
## Signif. codes:  0 '***' 0.001 '**' 0.01 '*' 0.05 '.' 0.1 ' ' 1
##
##                                            exp(coef) exp(-coef) lower .95 upper .95
## transplantCR1                                 0.4936     2.0260    0.2983    0.8166
## AOD_10                                        1.0730     0.9319    0.8504    1.3540
## clinicalData$M_Risk[osData$index]Favorable    0.4461     2.2414    0.1910    1.0421
## clinicalData$M_Risk[osData$index]Inter-1      0.7505     1.3324    0.4393    1.2824
## clinicalData$M_Risk[osData$index]Inter-2      0.2664     3.7540    0.1007    0.7049
##
## Concordance= 0.674  (se = 0.036 )
## Rsquare= 0.099   (max possible= 0.969 )
## Likelihood ratio test= 18.22  on 5 df,   p=0.002679
## Wald test            = 15.71  on 5 df,   p=0.00773
## Score (logrank) test = 16.76  on 5 df,   p=0.004988
```

```
> summary(coxph(Surv(time1/365, time2/365, status) ~ transplantCR1 + AOD_10 + clinicalData$M_R
isk[osData$index] , data=osData, subset=osData$index %in% which(s) & benefitGroup[osData$inde
x]=="High"))
Call:
coxph(formula = Surv(time1/365, time2/365, status) ~ transplantCR1 +
    AOD_10 + clinicalData$M_Risk[osData$index], data = osData,
    subset = osData$index %in% which(s) & benefitGroup[osData$index] ==
        "High")

  n= 368, number of events= 145
   (47 observations deleted due to missingness)

                                               coef exp(coef) se(coef)      z Pr(>|z|)
transplantCR1                              -0.41378   0.66115  0.17983 -2.301   0.0214 *
AOD_10                                      0.07182   1.07446  0.08099  0.887   0.3752
clinicalData$M_Risk[osData$index]Favorable -0.92663   0.39589  0.31007 -2.988   0.0028 **
clinicalData$M_Risk[osData$index]Inter-1   -0.34275   0.70982  0.19625 -1.747   0.0807 .
clinicalData$M_Risk[osData$index]Inter-2   -0.68085   0.50618  0.27900 -2.440   0.0147 *
---
Signif. codes:  0 '***' 0.001 '**' 0.01 '*' 0.05 '.' 0.1 ' ' 1

                                           exp(coef) exp(-coef) lower .95 upper .95
transplantCR1                                 0.6611     1.5125    0.4648    0.9405
AOD_10                                        1.0745     0.9307    0.9167    1.2593
clinicalData$M_Risk[osData$index]Favorable    0.3959     2.5260    0.2156    0.7270
clinicalData$M_Risk[osData$index]Inter-1      0.7098     1.4088    0.4832    1.0428
clinicalData$M_Risk[osData$index]Inter-2      0.5062     1.9756    0.2930    0.8746

Concordance= 0.599  (se = 0.026 )
Likelihood ratio test= 15.9  on 5 df,   p=0.007
Wald test            = 15.59  on 5 df,   p=0.008
Score (logrank) test = 15.83  on 5 df,   p=0.007
```

[outputs of the rerun (2021)]

Distribution of genetic variables

```
par(mar=c(6,3,1,1))
o <- order(-colSums(dataFrame[s,groups %in% c("Genetics","CNA","Fusions")]))
t <- t(sapply(split(dataFrame[s,groups %in% c("Genetics","CNA","Fusions")], benefitGroup[s]), colSums))
barplot(t[,o], col=pastel1[1:2], legend=TRUE, las=2, xaxs="i", cex.lab=0.66, args.legend=list(title="Benefit", bt
y="n", border=NA), border=NA)
```

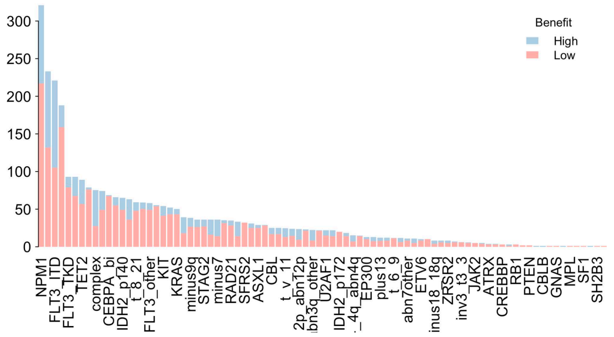

[outputs of the rerun (2021)]

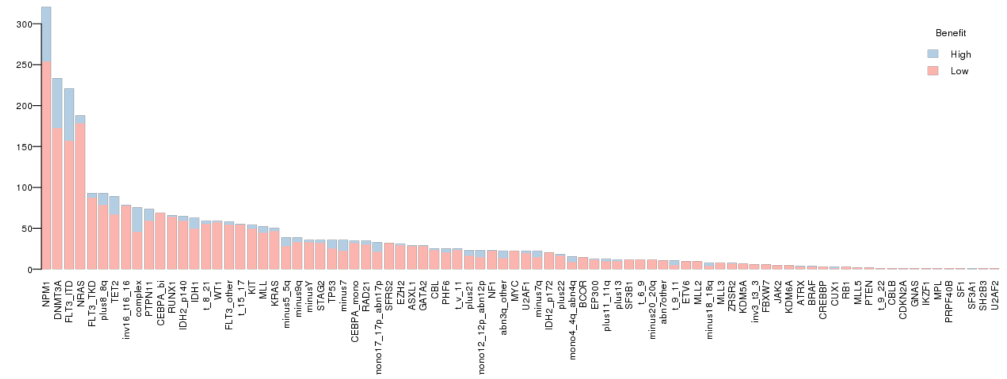

3.6.6.13.1 Leave one out cross-validation for RFX on post-CR OS

```
cvFold <- nrow(dataFrame)
cvIdx <- 1:nrow(dataFrame)
p <- Reduce("rbind", mclapply(cvIdx, function(i){
                    whichTrain <- which(cvIdx != i)
                    rfxOS <- CoxRFX(osData[osData$index %in% whichTrain, names(crGroups)], Surv(osData$time1, osD
ata$time2, osData$status)[osData$index %in% whichTrain], groups=crGroups, which.mu = intersect(mainGroups, unique
(crGroups)))
                    p <- as.data.frame(predict(rfxOS, newdata=osData[!osData$index %in% whichTrain, names(crGroup
s)], se.fit=TRUE))
                    s <- summary(survfit(Surv(osData$time1, osData$time2, osData$status)[osData$index %in% whichT
rain] ~ 1), time=3*365)$surv
                    cbind(p, surv=s^exp(p$fit))
                }, mc.cores=10))
d <- duplicated(sub(".1$","",rownames(p)))
coxRFXOsCrLOO <- rbind(p[!d,], p[d,])
rm(p,d)
```

Compare with corresponding multistage predictions

```
m <- c(multiRFX3TplLoo[,3],multiRFX3TplLoo[osData$index[osData$transplantCR1==1],2])
r <- c(coxRFXOsCrLOO$surv[1:1540],coxRFXOsCrLOO$surv[osData$transplantCR1==1])
plot(m, r)
abline(0,1)
```

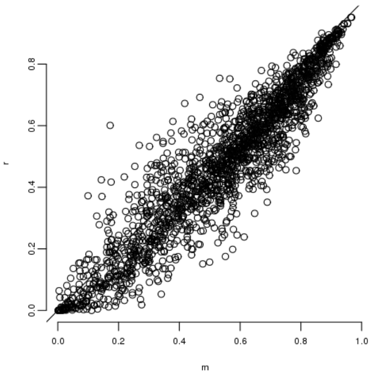

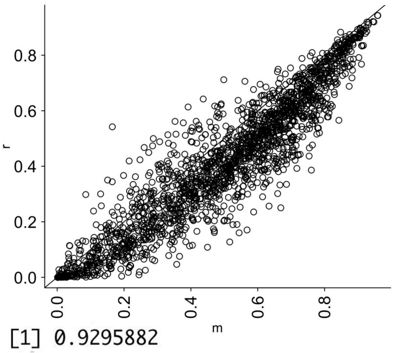

[1] 0.9295882

[outputs of the rerun (2021)]

```
cor(m, r)
```

```
## [1] 0.9314411
```

3.6.6.14 Prediction errors

3.6.6.14.1 Training error

3-state model

```
c <- Surv(as.numeric(clinicalData$Date_LF - clinicalData$CR_date), clinicalData$Status)
p <- multiRFX3TplCi[3,1,1,]
p[osData$index[osData$transplant1CR==1]] <-  multiRFX3TplCi[2,1,1,]
ape(p, c, time=3*365)
```

```
##       abs     brier      log2     bayes
## 0.3834699 0.1867695 0.7745674 0.2836879
```

```
> ape(p, c, time=3*365)
      abs     brier      log2     bayes
0.3830247 0.1850749 0.7695160 0.2758077
```

[outputs of the rerun (2021)]

RFX

```
unduplicate <- function(index) {u <- unique(index); u[which(rev(duplicated(rev(index))))] <- seq_along(index)[dup
licated(index)]; return(u)}
q <- summary(survfit(Surv(time1,time2,status) ~ 1, data=osData), time=3*365)$surv^exp(scale(predict(coxRFXOsCR, n
ewdata=osData[unduplicate(osData$index),]), scale=FALSE))
ape(q, c, time=3*365)
```

```
##       abs     brier      log2     bayes
## 0.3919441 0.1918189 0.7925326 0.2931442
```

```
> ape(q, c, time=3*365)
      abs     brier      log2     bayes
0.3919637 0.1920416 0.7933108 0.2947203
```

[outputs of the rerun (2021)]

3.6.6.14.2 LOO test error

3-state model

```
p <- multiRFX3TplLoo[,3]
p[osData$index[osData$transplantCR1==1]] <-  multiRFX3TplLoo[osData$index[osData$transplantCR1==1],2]
ape(p, c, time=3*365)
```

```
##       abs     brier      log2     bayes
## 0.3969946 0.1995807 0.8183460 0.3159968
```

```
> ape(p, c, time=3*365)
      abs     brier      log2     bayes
0.3967306 0.1973823 0.8122192 0.2962963
```

[outputs of the rerun (2021)]

RFX

```
ape(coxRFXOsCrLOO$surv[unduplicate(osData$index)], c, time=3*365)
```

```
##       abs     brier      log2     bayes
## 0.4086589 0.2070700 0.8496459 0.3167849
```

```
> ape(coxRFXOsCrLOO$surv[unduplicate(osData$
      abs     brier      log2     bayes
0.4123188 0.2141514 0.8798877 0.3372734
```

[outputs of the rerun (2021)]

3.6.7 Imputation of missing genes

Not all variables are needed to make predictions. Here we systematically assess the effect of the set of know genes for our preditive accuracy.

3.6.7.1 RFX model on OS

```
w <- WaldTest(coxRFXFitOsTDGGc)
o <- order(w$p.value[groups[whichRFXOsTDGG] %in% c("Genetics","GeneGene")])
genes <- unique(sub("_.+","",unlist(strsplit(names(whichRFXOsTDGG[groups[whichRFXOsTDGG]%in% c("Genetics","GeneGe
ne")])[o],":"))))

cvFold <- 1540
foo <- 42
set.seed(foo)
cvIdx <- 1:cvFold #sample(1:nrow(dataFrame)%% cvFold +1 ) ## sample 1/10

m <- unlist(sapply(1:cvFold, function(i) which(tplSplitOs %in% which(cvIdx==i))))
o <- order(m)

imputedRiskCv <- do.call("abind", c(mclapply(1:cvFold, function(i){
                            whichTrain <- which(cvIdx != i)
                            ix <- tplSplitOs %in% whichTrain
                            cRfx <- CoxRFX(dataFrameOsTD[ix,whichRFXOsTDGG], osTD[ix], groups[whichRFXOsTDGG], wh
ich.mu=mainGroups) ## allow only the main groups to have mean different from zero..

                            imputedRisk <- sapply(mclapply(c(0,seq_along(genes)), function(i){
                                        na.genes <- if(i==0) genes else genes[-(1:i)]
                                        if(length(na.genes)==0) na.genes <- "FOO42"
                                        d <- dataFrameOsTD[,whichRFXOsTDGG]
                                        d[grepl(paste(na.genes, collapse="|"), colnames(d))] <- NA
                                        p <- PredictRiskMissing(cRfx, d[!ix,,drop=FALSE])
                                    }, mc.cores=1), I, simplify="array")
                            dimnames(imputedRisk)[[3]] <- c("None",genes)
                            return(imputedRisk)

                        }, mc.cores=10), along=1))[o,,]
```

```
par(mar=c(3,3,3,1))
imputedCCv <- sapply(dimnames(imputedRiskCv)[[3]], function(i) as.numeric(survConcordance(osTD ~ imputedRiskCv[,1
,i])[c("concordance","std.err")]))
x <- 0:ncol(imputedCCv)-.5
plot(x, c(imputedCCv[1,], imputedCCv[1,ncol(imputedCCv)]), type="s", xaxt="n", xlab="", ylab="Concordance", ylim=
range(imputedCCv[1,]) + c(-1,1)*imputedCCv[2,1])
polygon(c(rep(x,each=2)[-c(1, 2*length(x))],rep(rev(x), each=2)[-c(1, 2*length(x))]), c(rep(imputedCCv[1,]+impute
dCCv[2,], each=2), rep(rev(imputedCCv[1,]-imputedCCv[2,]), each=2)), border=NA, col="#00000044")
mtext(dimnames(imputedRiskCv)[[3]], side=1, at=1:ncol(imputedCCv)-1, las=2, font=3, cex=.9)
abline(v=seq(0,50,10), lty=3)
abline(h=seq(0.68,0.73,0.01), lty=3)
axis(side=3)
```

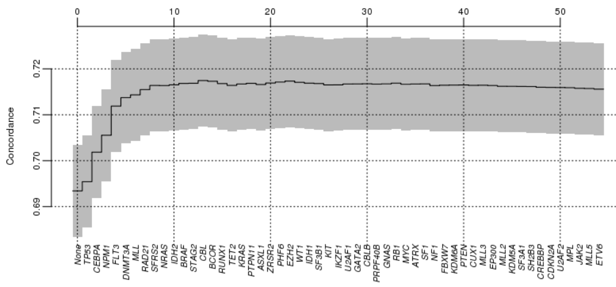

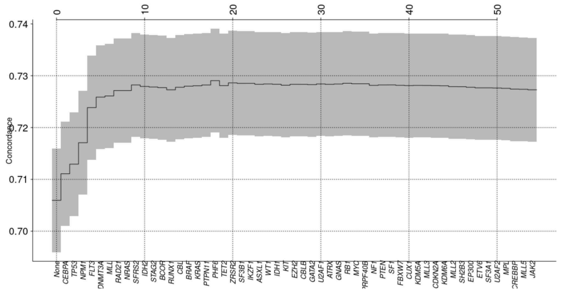

[outputs of the rerun (2021)]

3.6.7.2 Genetic imputation multi stage

As we noted, codes in sections 3.6.7.2–3.6.7.3 are also subject to an LSF environment for parallel computations. We were not able to tailor the entire R script to our own environments, as the computations are very intensive, and hence the modifications are prone to errors.

```
read_chunk('../code/imputation.R', labels="imputationMultiRfx")
```

```
#save(genes, file="genesImputation.RData")

load("loo.RData")
load("genesImputation.RData")
library(mg14)
library(CoxHD)
library(Rcpp)

i <- as.numeric(Sys.getenv("LSB_JOBINDEX"))

cvIdx <- 1:nrow(dataFrame)
whichTrain <- which(cvIdx != i)

e <- new.env()
t <- try(load(paste0("loo/",i,".RData"), env=e))
if(class(t)=="try-error"){
    stop()
}else{
    whichTrain <<- (1:nrow(data))[-i]
    dMiss <- do.call("rbind", lapply(c(0,seq_along(genes)), function(g){
                na.genes <- if(g==0) genes else genes[-(1:g)]
                if(length(na.genes)==0) na.genes <- "FOO42"
                d <- data[i,, drop=FALSE]
                d[grepl(paste(na.genes, collapse="|"), colnames(d))] <- NA
                d
            }))

    xx <- 0:2000
    coxphPrs <- coxph(Surv(time1, time2, status)~ pspline(time0, df=10), data=data.frame(prdData, time0=as.numeri
c(clinicalData$Recurrence_date-clinicalData$CR_date)[prdData$index])[prdData$index %in% whichTrain,])
    tdPrmBaseline <- exp(predict(coxphPrs, newdata=data.frame(time0=xx[-1])))

    coxphOs <- coxph(Surv(time1, time2, status)~ pspline(time0, df=10), data=data.frame(osData, time0=pmin(500,cr
[osData$index,1]))[osData$index %in% whichTrain,])
    tdOsBaseline <- exp(pmin(predict(coxphOs, newdata=data.frame(time0=500)),predict(coxphOs, newdata=data.frame(
time0=xx[-1])))) ## cap predictions at induction length 500 days.

    multiRfx5Imputed <- MultiRFX5(e$rfxEs, e$rfxCr, e$rfxNrs, e$rfxRel, e$rfxPrs, dMiss, tdPrmBaseline = tdPrmBas
eline, tdOsBaseline = tdOsBaseline, x=2000)
    save(multiRfx5Imputed, file=paste0("imputed/",i,".RData"))
}
```

Collect data

```
multiRfx5CvImputed <- sapply(mclapply(1:nrow(data), function(i){
            e <- new.env()
            t <- try(load(paste0("../code/imputed/",i,".RData"), env=e))
            if(class(t)=="try-error") return(rep(NA, length(genes)+1))
            else colSums(e$multiRfx5Imputed[3*365,1:3,])
        }, mc.cores=10), I)
```

3.6.7.3 Supplementary Figure S6B

Imputed accuracy

```
par(mar=c(3,3,3,1))
multiRfx5CvImputedC <- sapply(1:nrow(multiRfx5CvImputed), function(i) as.numeric(survConcordance(os ~ multiRfx5Cv
Imputed[i,])[c('concordance','std.err')]))
x <- 0:ncol(multiRfx5CvImputedC)-.5
plot(x, c(multiRfx5CvImputedC[1,], multiRfx5CvImputedC[1,ncol(multiRfx5CvImputedC)]), type="s", xaxt="n", xlab=""
, ylab="Concordance", ylim=range(multiRfx5CvImputedC[1,]) + c(-1,1)*multiRfx5CvImputedC[2,1])
polygon(c(rep(x,each=2)[-c(1, 2*length(x))],rep(rev(x), each=2)[-c(1, 2*length(x))]), c(rep(multiRfx5CvImputedC[1
,]+multiRfx5CvImputedC[2,], each=2), rep(rev(multiRfx5CvImputedC[1,]-multiRfx5CvImputedC[2,]), each=2)), border=N
A, col="#00000044")
mtext(dimnames(imputedRiskCv)[[3]], side=1, at=1:ncol(multiRfx5CvImputedC)-1, las=2, font=3, cex=.9)
abline(v=seq(0,50,10), lty=3)
abline(h=seq(0.68,0.73,0.01), lty=3)
axis(side=3)
```

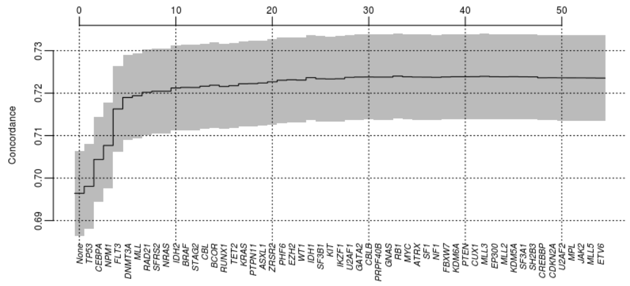


```
par(mar=c(3,3,3,1))
multiRfx5CvImputedApe <- sapply(1:nrow(multiRfx5CvImputed), function(i) ape(1-multiRfx5CvImputed[i,], os, 3*365))
x <- 0:ncol(multiRfx5CvImputedApe)-.5
for(i in 1:4){
    plot(x, c(multiRfx5CvImputedApe[i,], multiRfx5CvImputedApe[i,ncol(multiRfx5CvImputedApe)]), type="s", xaxt="n
", xlab="", ylab=rownames(multiRfx5CvImputedApe)[i], col=set1[i])
    mtext(dimnames(imputedRiskCv)[[3]], side=1, at=1:ncol(multiRfx5CvImputedApe)-1, las=2, font=3, cex=.9)
    abline(v=seq(0,50,10), lty=3)
    abline(h=axTicks(side=2), lty=3)
    axis(side=3)
}
```

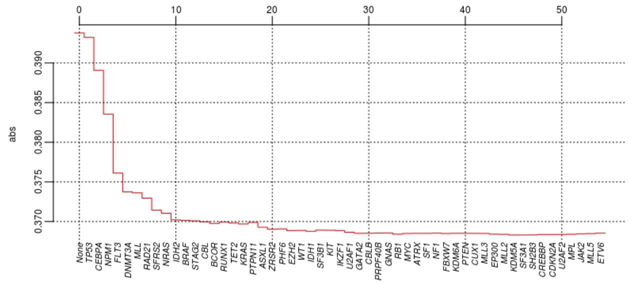

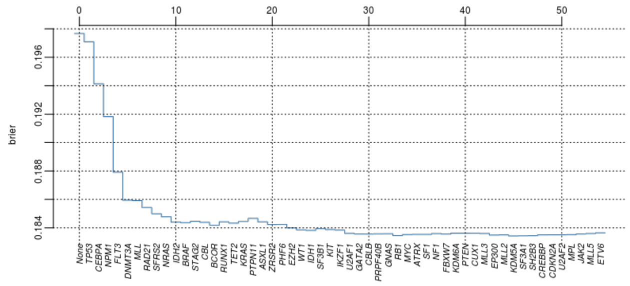

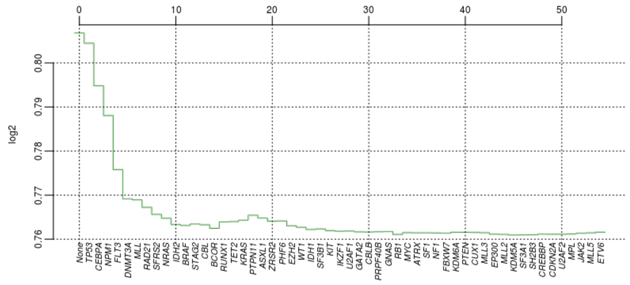

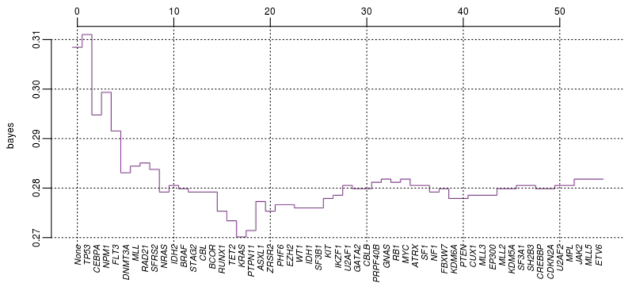

4 Model comparison

Choosing the optimal model needs to balance model complexity and statistical robustness. Simple models cannot reproduce all details of the underlying data and thus are inevitably biased. However they tend to be more robust to fit, with little variance between fits on different cohorts. More complex models have the potential to provide a more accurate fit, however ususally at the price of increased variance when applied to different data sets presenting a risk of overfitting. Both type of errors (systematic bias and random variance) impact on overall model accurcay. In statistical terms the resulting antagonism is therefore often expressed as a *bias-variance-tradeoff*, in which the overall model error is decomposed. The choice of a good model thus amounts to balance bias and variance.

A key quantity to define model complexity are the degrees of freedom. In a basic linear model the degrees of freedom are the number of parameters (variables). If more parameters are to be fit, each with a given uncertainty, the overall uncertainty of the predictions increases since the uncertainties in each parameter estimate tend to add up. Hence two strategies can be employed to reduce the total variance: First, one may reduce the total prediction variance by limiting the number of parameters (variable selection). Alternatively, one may also reduce the variance of individual estimates by imposing a penalty on large values (shrinkage). For example the note the LASSO model, underlying stability selection, achieves shrinkage (often to zero) with an L_1 -penalty. The random effects model shrinks parameters estimates using a quadratic penalty because the assumption that parameters share the same normal distribution implies that large absolute parameter values are less likely. It can be shown that this strategy also reduces the effective degrees of freedom of the model.

To assess which modeling strategy achieves the best balance between bias and variance we define different metrics for the model accuracy and employ cross-validation to evaluate the performance of different modelling strategies. The idea is to split the data into a training and a test set; the model is fitted on the training part and its prognostic accuracy evaluated on the test set. This yields more reliable estimates of the overall model error.

4.1 Random cross validation

We have randomly split the data 100 times 80% training data and 20% validation data. For each split, we evaluated the following metrics:

- Concordance, defined as the probability that the survival times of two individuals are concordant to the ranking of their risk (Harrell, Lee, and Mark 1996), implemented in `survival::survConcordance()`
- Area under the receiver-operating characteristic curve (AUC), a measure of the correct classification into dead and alive at a given time (Uno et al. 2007), implemented in `survAUC::auc.Uno()` .
- Brier score, an absolute measure of the prediction error (Gerds and Schumacher 2006), implemented in `survAUC::predErr()` .
- A generalised coefficient of explained variation R^2 (Nagelkerke 1991,O'Quigley, Xu, and Stare (2005)) , implemented as `survAUC::OXS()` .

The latter three algorithms are implemented in the `survAUC` `R` package (Potapov, Adler, and Schmid. 2012).

4.2 Inter-trial

The data comprised patients from three different trials - AMLSG07/04 (n=740), AMLHD98A (n=627), and AMLHD98B (n=173). In addition to randomly splitting the data into training and test partitions, we trained the model on all three combinations of 2 trials and evaluated the prognostic accuracy on the third trial. This situation is more challenging as there may be some systematic differences between the trials, but it can also be expected to more closely mimic the situation of predicting a novel cohort.

4.3 TCGA

As an additional and independent evaluation cohort, we downloaded data from the cancer genome atlas (TCGA) (Cancer Genome Atlas Research Network 2013). We downloaded variant calls from exome sequencing and cytogenetic data for n=200 and annotated oncogenic mutations as described in our companion paper. Overall survival was available for n=186 patients. For missing prognostic variables, we use a covariance-based imputation, with a covariance matrix derived from our original data set (n=1,540). We note that there was no data available for allografts.

4.4 Code

4.4.1 Systematic cross-validation

4.4.1.1 Static models

```
library(rpart)
library(randomForestSRC)
```

```
##
##  randomForestSRC 1.6.0
##
##  Type rfsrc.news() to see new features, changes, and bug fixes.
##
```

```r
replicates <- 100 ## number of replicates
scope <- c("Genetics","CNA","Treatment","Fusions") ## For CPSS
scopeStep <- as.formula(paste("os ~", paste(colnames(dataFrame)[mainIdxOs& osIdx], collapse="+"))) ## For AIC&BIC
allModelsCV <- mclapply(1:replicates, function(foo){
            set.seed(foo)
            trainIdx <- sample(1:nrow(dataFrame)%%5 +1 )!=1 ## sample 1/5
            c <- coxph(os[trainIdx] ~ 1, data=dataFrame[trainIdx,mainIdxOs])
            scopeStep <- as.formula(paste("os[trainIdx] ~", paste(colnames(dataFrame)[mainIdxOs], collapse="+")))
            coxBICOsTrain <- step(c, scope=scopeStep, k = log(sum(trainIdx)), trace=0)
            coxAICOsTrain <- step(coxBICOsTrain, scope=scopeStep, k = 2, trace=0)
            coxCPSSOsTrain <- CoxCPSSInteractions(dataFrame[!is.na(os) & trainIdx, mainIdxOs], na.omit(os[trainId
x]), bootstrap.samples=50, scope = which(groups %in% scope))
            coxRFXOsTrain <- CoxRFX(dataFrame[trainIdx,mainIdxOs], os[trainIdx], groups=groups[mainIdxOs])
            coxRFXOsTrain$Z <- NULL
            coxRFXOsGGc <- CoxRFX(dataFrame[trainIdx,whichRFXOsGG], os[trainIdx], groups=groups[whichRFXOsGG], wh
ich.mu=mainGroups)
            coxRFXOsGGc$Z <- NULL
            rForestOsTrain <- rfsrc(Surv(time, status) ~.,data= cbind(time = os[,1], status = os[,2], dataFrame[,
mainIdxOs])[trainIdx,], ntree=100, importance="none")
            return(list(
                            BIC=coxBICOsTrain,
                            AIC=coxAICOsTrain,
                            CPSS=coxCPSSOsTrain,
                            RFX=coxRFXOsTrain,
                            RFXgg=coxRFXOsGGc,
                            rForest=rForestOsTrain
                    ))
        }, mc.cores=10)
```

Compute predictions for all model fits

```r
predictAllModels <- function(x, newdata){
    if("rfsrc" %in% class(x)){
        predict(x, newdata, importance="none")$predicted
    }else{
        predict(x, newdata)
    }
}

allModelsCvPredictions <- mclapply(seq_along(allModelsCV), function(foo){
            set.seed(foo)
            x <- allModelsCV[[foo]]
            trainIdx <- sample(1:nrow(dataFrame)%%5 +1 )!=1 ## sample 1/5
            cbind(ELN=c(4,1,3,2)[clinicalData$M_Risk[!trainIdx]],
                    sapply(x, function(y){
                                predictAllModels(y, newdata=dataFrame[!trainIdx,])
                            }))
        }, mc.cores=10)

colModels <- c("#888888", set1[c(2,1,4,3,5,7)])
```

Harrel's C

```r
foo <- 1
allModelsCvC <- sapply(allModelsCvPredictions, function(x){
            set.seed(foo)
            trainIdx <- sample(1:nrow(dataFrame)%%5 +1 )!=1 ## sample 1/5
            foo <<- foo +1
            apply(x, 2 , function(p){
                        survConcordance(osYr[!trainIdx,] ~ p)$concordance
                    })
        })
apply(allModelsCvC,1,quantile)
```

```
##            ELN       BIC       AIC      CPSS       RFX     RFXgg   rForest
## 0%   0.5880492 0.6423902 0.6358633 0.6454865 0.6534156 0.6579039 0.6539126
## 25%  0.6281709 0.6816841 0.6760669 0.6807904 0.6894067 0.6943526 0.6800193
## 50%  0.6437515 0.6918903 0.6923465 0.6955938 0.7050271 0.7095102 0.6918675
## 75%  0.6546729 0.7048202 0.7055158 0.7048667 0.7165585 0.7202644 0.7039033
## 100% 0.6863701 0.7407616 0.7372427 0.7390396 0.7483232 0.7490528 0.7363960
```

```r
par(mar=c(3,3,1,1),bty="n", mgp=c(2,.5,0), las=2)
r <- sapply(as.data.frame(lapply(as.data.frame(t(apply(-allModelsCvC,2,rank))),factor, levels=1:7)),table)
o <- order(apply(allModelsCvC,1,median))
boxplot(t(allModelsCvC[o,]), notch=TRUE, ylab="Concordance", staplewex=0, lty=1, pch=16, xaxt="n")
rotatedLabel(1:7, rep(par("usr")[3],7), rownames(allModelsCvC)[o])
```

```
> apply(allModelsCvC,1,quantile)
           ELN       BIC       AIC      CPSS       RFX     RFXgg   rForest
0%   0.5886008 0.6603639 0.6529221 0.6624138 0.6621309 0.6652691 0.6523860
25%  0.6267595 0.6898046 0.6881246 0.6897148 0.6975971 0.6976143 0.6862839
50%  0.6384101 0.7004565 0.6951043 0.7034819 0.7070043 0.7085601 0.6973769
75%  0.6521891 0.7124851 0.7108459 0.7117075 0.7188193 0.7219131 0.7080744
100% 0.6793053 0.7452558 0.7409260 0.7614175 0.7560732 0.7618177 0.7480995
```

[outputs of the rerun (2021)]

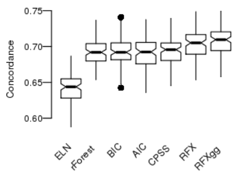

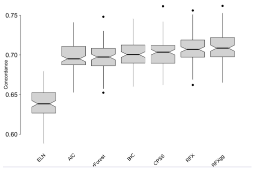

[outputs of the rerun (2021)]

```
par(mar=c(3,3,3,1), xpd=NA, las=2, mgp=c(2,.5,0))
barplot(r[,o]/replicates, col=c(set1[c(3,2,4,1,5,7)],"grey"), ylab="Fraction", names.arg=rep("",ncol(r))) -> b
rotatedLabel(b, rep(par("usr")[3],6), colnames(allModelsCvC)[o])
legend(par("usr")[1],1.5, fill=c(set1[c(3,2,4,1,5,7)],"grey"), legend=1:6, bty="n", border=NA, horiz=TRUE, title=
"Rank")
```

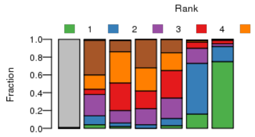

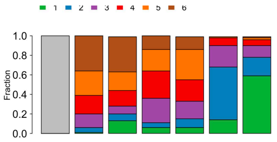

[outputs of the rerun (2021)]

Brier scores

```
library(survAUC)
foo <- 1
allModelsCvBrier<- sapply(allModelsCV, function(x){
            set.seed(foo)
            trainIdx <- sample(1:nrow(dataFrame)%%5 +1 )!=1 ## sample 1/5
            foo <<- foo +1
            sapply(x, function(y){
                        p <- predictAllModels(y, newdata=dataFrame)
                        a <- predErr(Surv.rsp = osYr[trainIdx,], Surv.rsp.new = osYr[!trainIdx,], lp=p[trainIdx],
lpnew = p[!trainIdx], times= c(90,365,1000)/365, type="brier")$error
                    })
        })
apply(allModelsCvBrier,1,quantile)
```

```
##               [,1]      [,2]      [,3]       [,4]      [,5]      [,6]       [,7]      [,8]      [,9]      [,10]
[,11]
## 0%   0.05849797 0.1526225 0.1757253 0.05944734 0.1503072 0.1760910 0.05846343 0.1511088 0.1786459 0.05605151 0
.1495037
## 25%  0.07750536 0.1753677 0.1937367 0.07789139 0.1761197 0.1978792 0.07718722 0.1741391 0.1952522 0.07664560 0
.1721626
## 50%  0.08304442 0.1835706 0.2036727 0.08438041 0.1833998 0.2071846 0.08321443 0.1819881 0.2040855 0.08271161 0
.1798175
## 75%  0.09084118 0.1898236 0.2102678 0.09188209 0.1912654 0.2164903 0.09047345 0.1882972 0.2085808 0.09088417 0
.1865091
## 100% 0.10871873 0.2126778 0.2380005 0.10799058 0.2130452 0.2498866 0.10677651 0.2135231 0.2329530 0.10806363 0
.2077360
##          [,12]      [,13]     [,14]     [,15]      [,16]     [,17]     [,18]
## 0%   0.1741597 0.05555387 0.1464211 0.1717377 0.06177217 0.2322100 0.3047124
## 25%  0.1886995 0.07639397 0.1704750 0.1870156 0.09108158 0.2752806 0.3576704
## 50%  0.1977054 0.08184680 0.1792870 0.1958929 0.10079971 0.2934602 0.3789192
## 75%  0.2050220 0.09022773 0.1849654 0.2046791 0.11054862 0.3124864 0.3938336
## 100% 0.2407017 0.10635080 0.2035862 0.2412429 0.13336421 0.3411525 0.4467354
```

```
rownames(allModelsCvBrier) <- paste(rep(names(allModelsCV[[1]]), each=3), rep(c(90,365,1000), length(allModelsCV[
[1]])))
boxplot(t(allModelsCvBrier)[,rep(0:5*3, 3) + rep(1:3, each=6)],notch=TRUE, ylab="Brier score", border=rep(colMode
ls[-1],3), las=2, lty=1, pch=16, staplewex=0)
```

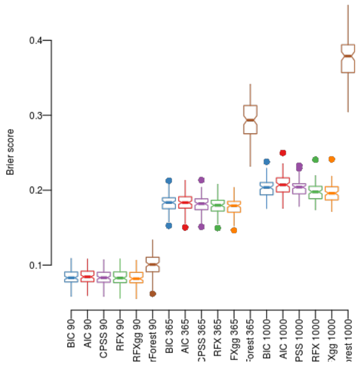

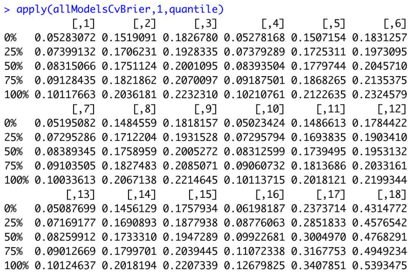
```
> apply(allModelsCvBrier,1,quantile)
           [,1]      [,2]      [,3]       [,4]      [,5]      [,6]
0%   0.05283072 0.1519091 0.1826780 0.05278168 0.1507154 0.1831257
25%  0.07399132 0.1706231 0.1928335 0.07379289 0.1725311 0.1973095
50%  0.08315066 0.1751124 0.2001095 0.08393504 0.1779744 0.2045710
75%  0.09128435 0.1821862 0.2070097 0.09187501 0.1868265 0.2135375
100% 0.10117663 0.2036181 0.2232310 0.10210761 0.2122635 0.2324579
           [,7]      [,8]      [,9]      [,10]     [,11]     [,12]
0%   0.05195082 0.1484559 0.1818157 0.05023424 0.1486613 0.1784422
25%  0.07295286 0.1712204 0.1931528 0.07295794 0.1693835 0.1903410
50%  0.08389345 0.1758959 0.2005272 0.08312599 0.1739495 0.1953132
75%  0.09103505 0.1827483 0.2085071 0.09060732 0.1813686 0.2033161
100% 0.10033613 0.2067138 0.2214645 0.10113715 0.2018121 0.2199344
          [,13]     [,14]     [,15]      [,16]     [,17]     [,18]
0%   0.05087699 0.1456129 0.1757934 0.06198187 0.2373714 0.4314772
25%  0.07169177 0.1690893 0.1877938 0.08776063 0.2851833 0.4576542
50%  0.08259912 0.1733310 0.1947289 0.09922681 0.3004970 0.4768291
75%  0.09012669 0.1799701 0.2039445 0.11072338 0.3167753 0.4949234
100% 0.10124637 0.2018194 0.2207339 0.12679825 0.3407851 0.5393475
```

GHCI

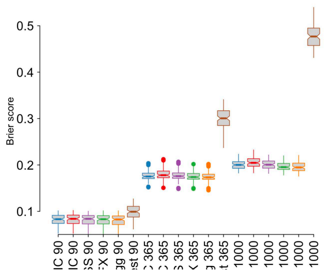

[outputs of the rerun (2021)]

```
allModelsCvGHCI<- sapply(allModelsCvPredictions, function(x){
            apply(x[,2:6], 2 , function(p){
                        p <- GHCI(lpnew = na.omit(p))
                    })
        })
apply(allModelsCvGHCI,1,quantile)
```

```
##            BIC       AIC      CPSS       RFX     RFXgg
## 0%   0.6806809 0.6982268 0.6756061 0.6867523 0.6951368
## 25%  0.6944020 0.7155827 0.6928061 0.6965384 0.7075201
## 50%  0.7008508 0.7208792 0.6976200 0.7029009 0.7117394
## 75%  0.7047188 0.7271990 0.7032465 0.7079622 0.7169094
## 100% 0.7210609 0.7407248 0.7163284 0.7179690 0.7258588
```

```
boxplot(t(allModelsCvGHCI),notch=TRUE, ylab="GH C", border=colModels[2:6], las=2, lty=1, pch=16, staplewex=0)
```

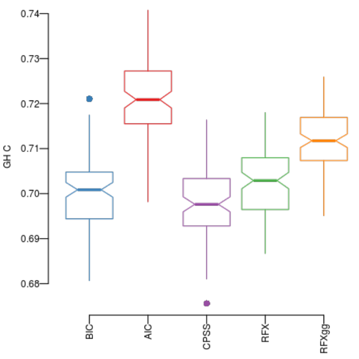

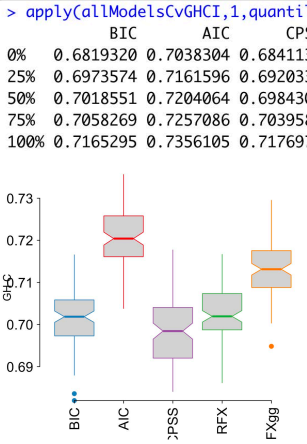

[outputs of the rerun (2021)]

OXS R2 estimates

```
foo <- 1
allModelsCvOXS <- sapply(allModelsCvPredictions, function(x){
            set.seed(foo)
            trainIdx <- sample(1:nrow(dataFrame)%%5 +1 )!=1 ## sample 1/5
            foo <<- foo +1
            apply(x[,2:6], 2 , function(p){
                        a <- OXS(osYr[!trainIdx,], p, rep(0,length(p)))
                    })
        })
apply(allModelsCvOXS,1,quantile)
```

```
##            BIC           AIC       CPSS       RFX     RFXgg
## 0%   0.1289646 -4.161681e+238 0.08704625 0.1436286 0.1531176
## 25%  0.2990897   1.889040e-01 0.30089659 0.3180463 0.3265460
## 50%  0.3431369   2.620666e-01 0.34378953 0.3746620 0.3838629
## 75%  0.3862799   3.374566e-01 0.38786964 0.4142649 0.4283471
## 100% 0.4765289   4.554361e-01 0.47522406 0.5001872 0.5180129
```

```
boxplot(t(allModelsCvOXS), ylim=c(0,0.5), notch=TRUE, ylab="OXS R2", border=colModels[2:6], las=2, lty=1, pch=16,
staplewex=0)
```

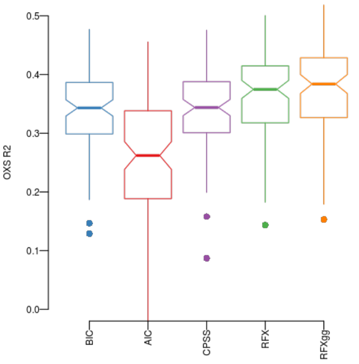

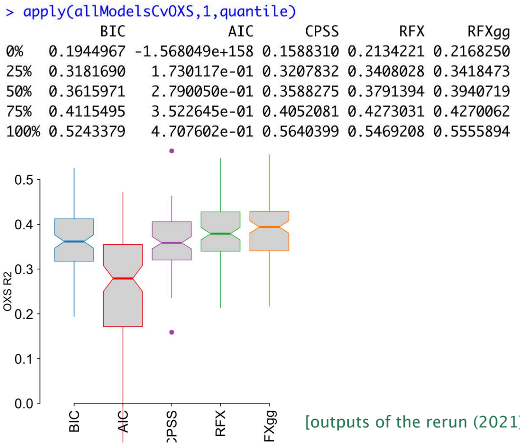

Nagelk R2 estimates

[outputs of the rerun (2021)]

```r
foo <- 1
allModelsCvNagelk <- sapply(allModelsCvPredictions, function(x){
            set.seed(foo)
            trainIdx <- sample(1:nrow(dataFrame)%%5 +1 )!=1 ## sample 1/5
            foo <<- foo +1
            apply(x[,2:6], 2 , function(p){
                        a <- Nagelk(osYr[!trainIdx,], p, rep(0,length(p)))
                    })
        })
apply(allModelsCvNagelk,1,quantile)
```

```
##              BIC           AIC       CPSS        RFX      RFXgg
## 0%   0.08257594 -1.993769e+150 0.05609423 0.08634564 0.09225612
## 25%  0.18763141   1.149669e-01 0.19279267 0.20628936 0.21301746
## 50%  0.21625945   1.628463e-01 0.21672847 0.24227620 0.24468426
## 75%  0.24690641   2.180015e-01 0.24761718 0.27236095 0.28003519
## 100% 0.31557340   2.995430e-01 0.29898599 0.32345779 0.33542100
```

```r
boxplot(t(allModelsCvNagelk), ylim=c(0,0.4), notch=TRUE, ylab="Nagelk's R2", border=colModels[2:6], las=2, lty=1,
pch=16, staplewex=0)
```

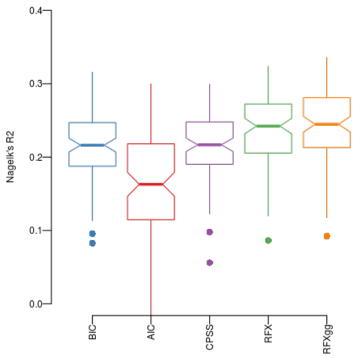


```
> apply(allModelsCvNagelk,1,quantile)
           BIC           AIC       CPSS       RFX     RFXgg
0%   0.1226315 -2.073888e+87 0.09933272 0.1351605 0.1326734
25%  0.2014981  1.080911e-01 0.20248005 0.2157883 0.2130064
50%  0.2298235  1.719372e-01 0.22729365 0.2433718 0.2484829
75%  0.2606386  2.270871e-01 0.26049516 0.2734872 0.2757005
100% 0.3197915  2.926640e-01 0.34988451 0.3367527 0.3433717
```

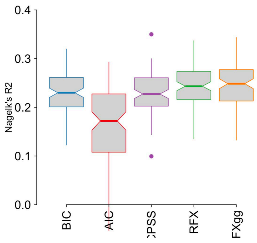

[outputs of the rerun (2021)]

UnoC

```r
foo <- 1
allModelsCvUnoC<- sapply(allModelsCvPredictions, function(x){
            set.seed(foo)
            trainIdx <- sample(1:nrow(dataFrame)%%5 +1 )!=1 ## sample 1/5
            foo <<- foo +1
            apply(x,2, function(p){
                        a <- UnoC(Surv.rsp = osYr[trainIdx,], Surv.rsp.new = osYr[!trainIdx,][!is.na(p)],  lpnew
= na.omit(p), time=5)
                    })
        })
apply(allModelsCvUnoC,1,quantile)
```

```
##            ELN       BIC       AIC      CPSS       RFX     RFXgg   rForest
## 0%   0.4512105 0.6430067 0.6334845 0.6451623 0.6509440 0.6557092 0.6498282
## 25%  0.5026052 0.6825757 0.6772569 0.6826433 0.6911294 0.6934710 0.6797299
## 50%  0.5176337 0.6926510 0.6930621 0.6956262 0.7032943 0.7085578 0.6910261
## 75%  0.5304213 0.7048022 0.7047715 0.7049114 0.7170992 0.7202833 0.7029446
## 100% 0.5736298 0.7379782 0.7371860 0.7353561 0.7506707 0.7493965 0.7393023
```

```r
boxplot(t(allModelsCvUnoC), notch=TRUE,  ylab="Uno's C", border=colModels, lty=1, pch=16, staplewex=0)
```

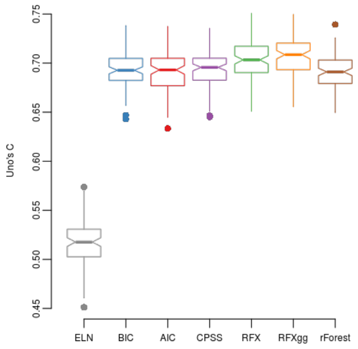


```
> apply(allModelsCvUnoC,1,quantile)
           ELN       BIC       AIC      CPSS       RFX     RFXgg   rForest
0%   0.4624404 0.6578594 0.6527207 0.6584551 0.6617980 0.6637549 0.6506456
25%  0.4999048 0.6889159 0.6864500 0.6880861 0.6976440 0.6974822 0.6850143
50%  0.5100012 0.7010170 0.6952299 0.7020001 0.7072516 0.7085411 0.6967413
75%  0.5302898 0.7114135 0.7099348 0.7107660 0.7185439 0.7207884 0.7057719
100% 0.5617483 0.7495927 0.7443142 0.7667109 0.7597657 0.7653807 0.7521385
```

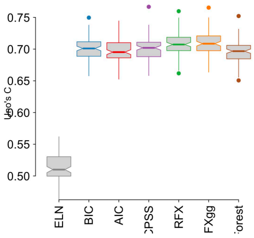

[outputs of the rerun (2021)]

AUC UNO

```
t <- seq(0.1,5,0.1) #times
allModelsCvAuc <- sapply(seq_along(allModelsCvPredictions), function(foo){
            set.seed(foo)
            trainIdx <- sample(1:nrow(dataFrame)%%5 +1 )!=1 ## sample 1/5
            apply(allModelsCvPredictions[[foo]], 2, function(p){
                        AUC.uno(osYr[trainIdx,], osYr[!trainIdx, ][!is.na(p)], scale(na.omit(p)), t)$auc
                    })
        })
allModelsCvAuc <- array(allModelsCvAuc, dim=c(length(t),ncol(allModelsCvPredictions[[1]]),length(allModelsCvPredi
ctions)))
plot(NA,NA, xlab="Years",ylab="AUC", xlim=range(t), ylim=c(0.5,0.8))
for(i in 1:dim(allModelsCvAuc)[2]){
    lines(t,rowMeans(allModelsCvAuc, dims=2)[,i], type='l', new=i==1, col=colModels[i])
}
legend("bottomright", colnames(allModelsCvPredictions[[1]]), bty="n", lty=1, col=colModels)
```

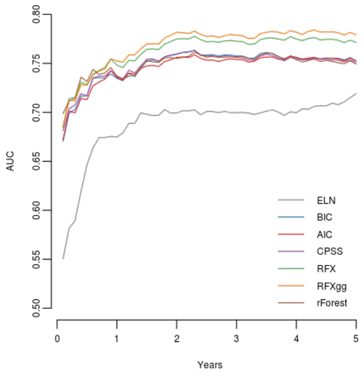

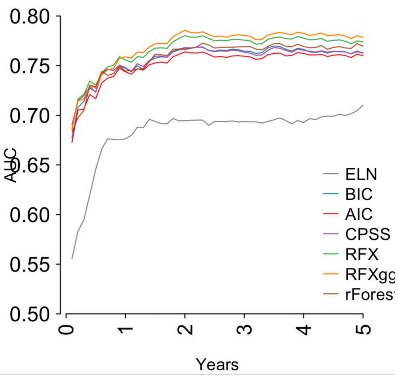

[outputs of the rerun (2021)]

Wisdom of the crowds?

```
foo <- 1
allModelsCvCCrowd <- sapply(allModelsCvPredictions, function(x){
            set.seed(foo)
            trainIdx <- sample(1:nrow(dataFrame)%%5 +1 )!=1 ## sample 1/5
            foo <<- foo +1
            r <- rowMeans(apply(x, 2 , rank))
            survConcordance(osYr[!trainIdx,] ~ r)$concordance
        })
quantile(allModelsCvCCrowd)
```

```
##        0%       25%       50%       75%      100% 
## 0.6648901 0.6949264 0.7088463 0.7206734 0.7520978
```

```
boxplot(cbind(t(allModelsCvC),allModelsCvCCrowd), notch=TRUE, ylab="Concordance", border=c(colModels,1), las=2, l
ty=1, pch=16, staplewex=0)
```

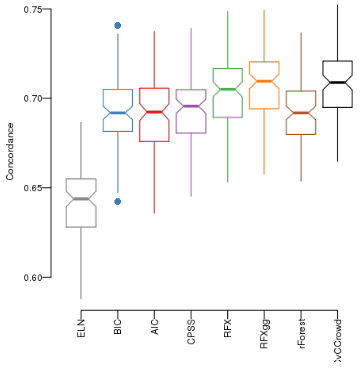


```
> quantile(allModelsCvCCrowd)
       0%       25%       50%       75%      100% 
0.6682143 0.7010209 0.7118930 0.7230097 0.7605459
```

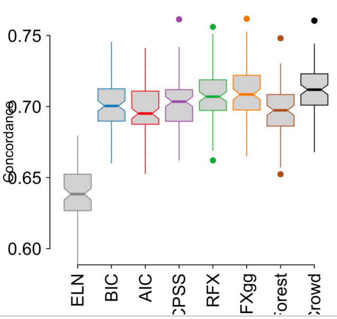

[outputs of the rerun (2021)]

```
ranks <- apply(apply(-cbind(t(allModelsCvC),kraut=allModelsCvCCrowd),1,rank, ties.method="random"),1,function(x)
table(factor(x, levels=1:8)))
ranks <- ranks[,order(1:8 %*% ranks)]
```

Clean up..

```
rm(allModelsCV)
```

4.4.1.2 Different RFX models

Here we assess RFX models with interaction terms for different variable categories.

```
replicates <- 100 ## number of replicates
allModelsCvRfxC <- do.call("rbind",mclapply(1:100, function(foo){
                    set.seed(foo)
                    trainIdx <- sample(1:nrow(dataFrameOsTD)%%5 +1 )[tplSplitOs]!=1 ## sample 1/5
                    coxRFXOsMain <- CoxRFX(dataFrameOsTD[trainIdx,mainIdxOsTD], osTD[trainIdx], groups=groups[mainIdxOsTD])
                    coxRFXOsGG <- CoxRFX(dataFrameOsTD[trainIdx,whichRFXOsTDGG], osTD[trainIdx], groups=groups[whichRFXOsTDGG])
                    coxRFXOsGGc <- CoxRFX(dataFrameOsTD[trainIdx,whichRFXOsTDGG], osTD[trainIdx], groups=groups[whichRFXOsTDGG], which.mu=mainGroups)
                    coxRFXOsAll <- CoxRFX(dataFrameOsTD[trainIdx,whichRFXOsTD], osTD[trainIdx], groups=groups[whichRFXOsTD])
                    coxRFXOsAllc <- CoxRFX(dataFrameOsTD[trainIdx,whichRFXOsTD], osTD[trainIdx], groups=groups[whichRFXOsTD], which.mu=mainGroups)
                    return(c(
                                    Main=survConcordance(osTD[!trainIdx]~as.matrix(dataFrameOsTD[!trainIdx,mainIdxOsTD]) %*% coef(coxRFXOsMain))$concordance,
                                    GeneGene=survConcordance(osTD[!trainIdx]~as.matrix(dataFrameOsTD[!trainIdx,whichRFXOsTDGG]) %*% coef(coxRFXOsGG))$concordance,
                                    GeneGeneCentred=survConcordance(osTD[!trainIdx]~as.matrix(dataFrameOsTD[!trainIdx,whichRFXOsTDGG]) %*% coef(coxRFXOsGGc))$concordance,
                                    AllInt=survConcordance(osTD[!trainIdx]~as.matrix(dataFrameOsTD[!trainIdx,whichRFXOsTD]) %*% coef(coxRFXOsAll))$concordance,
                                    AllIntCentred=survConcordance(osTD[!trainIdx]~as.matrix(dataFrameOsTD[!trainIdx,whichRFXOsTD]) %*% coef(coxRFXOsAllc))$concordance
                            ))
                }, mc.cores=10))
colnames(allModelsCvRfxC) <- sub(".concordant","",colnames(allModelsCvRfxC))
```

```
par(mar=c(3,3,1,1),bty="n", mgp=c(2,.5,0), las=2)
r <- sapply(as.data.frame(lapply(as.data.frame(round(t(apply(-allModelsCvRfxC,1,rank)))),factor, levels=1:6)),table)
o <- order(colMeans(allModelsCvRfxC))
boxplot(allModelsCvRfxC[,o], notch=TRUE, ylab="Concordance", staplewex=0, lty=1, pch=16, xaxt="n")
rotatedLabel(1:ncol(allModelsCvRfxC), rep(par("usr")[3],ncol(allModelsCvRfxC)), colnames(allModelsCvRfxC)[o])
```

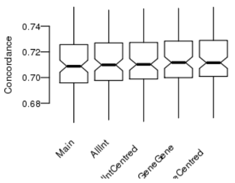

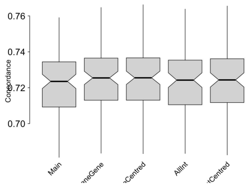

[outputs of the rerun (2021)]

```
par(mar=c(3,3,3,1), xpd=NA, las=2, mgp=c(2,.5,0))
barplot(r[,o]/replicates, col=set1[c(3,2,4,1,5,7)][1:ncol(allModelsCvRfxC)], ylab="Fraction", names.arg=rep("",ncol(r))) -> b
rotatedLabel(b, rep(par("usr")[3],ncol(allModelsCvRfxC)), colnames(allModelsCvRfxC)[o])
legend(par("usr")[1],1.5, fill=set1[c(3,2,4,1,5,7)][1:ncol(allModelsCvRfxC)], legend=1:ncol(allModelsCvRfxC), bty="n", border=NA, horiz=TRUE, title="Rank")
```

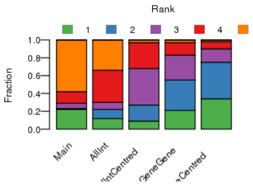

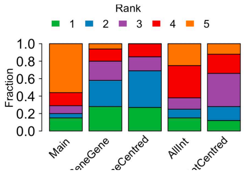

[outputs of the rerun (2021)]

4.4.1.3 Time-dependent models

The following models allow for quantifying the effect of a time-dependent covariate, such as a bone marrow transplant, which is typically administered after diagnosis. The subsequent code is executed on our LSF cluster for 100 replicates

```
read_chunk('../code/cv100.R', labels="allModelsCVTDCode")
```

As we noted, codes in sections 4.4.1.3 are also subject to an LSF environment for parallel computations. We were not able to tailor the entire R script to our own environments, as the computations are very intensive, and hence the modifications are prone to errors.

```r
#save(dataFrame, nrdData, crGroups, mainGroups, prdData, relData, prdData, osData, cr, dataFrameOsTD, dataFrame, osTD, tplSplitOs, groups, data, whichRFXOsTDGG, mainIdxOs, clinicalData, MultiRFX5, os, mainIdxOsTD, scope, which RFXOsGG, file="../../code/cv100.RData")

load("cv100.RData")
library(mg14)
library(CoxHD)
library(Rcpp)
library(randomForestSRC)


jobIndex <- as.numeric(Sys.getenv("LSB_JOBINDEX"))

set.seed(jobIndex)
splits <- sample(1:nrow(dataFrame)%%5 +1 )
trainIdx <- splits!=1 ## sample 1/5

# Static models (other)
c <- coxph(os[trainIdx] ~ 1, data=dataFrame[trainIdx,mainIdxOs])
scope <- c("Genetics","CNA","Treatment","Fusions") ## For CPSS
scopeStep <- as.formula(paste("os[trainIdx] ~", paste(colnames(dataFrame)[mainIdxOs], collapse="+")))
coxBICOs <- step(c, scope=scopeStep, k = log(sum(trainIdx)), trace=0)
coxAICOs <- step(coxBICOs, scope=scopeStep, k = 2, trace=0)
coxCPSSOs <- CoxCPSSInteractions(dataFrame[!is.na(os) & trainIdx, mainIdxOs], na.omit(os[trainIdx]), bootstrap.samples=50, scope = which(groups %in% scope))
coxRFXOs <- CoxRFX(dataFrame[trainIdx,mainIdxOs], os[trainIdx], groups=groups[mainIdxOs])
coxRFXOs$Z <- NULL
coxRFXOsGGc <- CoxRFX(dataFrame[trainIdx,whichRFXOsGG], os[trainIdx], groups=groups[whichRFXOsGG], which.mu=mainGroups)
coxRFXOsGGc$Z <- NULL
rForestOsTrain <- rfsrc(Surv(time, status) ~.,data= cbind(time = os[,1], status = os[,2], dataFrame[,mainIdxOs])[trainIdx,], ntree=100, importance="none")

# Time-dependent models
trainIdxTD <- splits[tplSplitOs]!=1 ## sample 1/5
c <- coxph(osTD[trainIdxTD] ~ 1, data=dataFrameOsTD[trainIdxTD,mainIdxOsTD])
scopeStep <- as.formula(paste("osTD[trainIdx] ~", paste(colnames(dataFrameOsTD)[mainIdxOsTD], collapse="+")))
coxBICOsTD <- step(c, scope=scopeStep, k = log(sum(trainIdxTD)), trace=0)
coxAICOsTD <- step(coxBICOsTD, scope=scopeStep, k = 2, trace=0)
coxRFXOsTD <- CoxRFX(dataFrameOsTD[trainIdxTD,mainIdxOsTD], osTD[trainIdxTD], groups=groups[mainIdxOsTD])
coxRFXOsTD$Z <- NULL
coxRFXOsTDGGc <- CoxRFX(dataFrameOsTD[trainIdxTD,whichRFXOsTDGG], osTD[trainIdxTD], groups=groups[whichRFXOsTDGG], which.mu=mainGroups)
coxRFXOsTDGGc$Z <- NULL

# Multi-stage model
whichTrain <- which(trainIdx)
rfxNrs <- CoxRFX(nrdData[nrdData$index %in% whichTrain, names(crGroups)], Surv(nrdData$time1, nrdData$time2, nrdData$status)[nrdData$index %in% whichTrain], groups=crGroups, nu=ifelse(jobIndex==45,1,0), which.mu = intersect(mainGroups, unique(crGroups))) #avoiding data singularity in split 45
rfxNrs$coefficients["transplantRel"] <- 0
rfxPrs <-  CoxRFX(prdData[prdData$index %in% whichTrain, names(crGroups)], Surv(prdData$time1, prdData$time2, prdData$status)[prdData$index %in% whichTrain], groups=crGroups, nu=1, which.mu = intersect(mainGroups, unique(crGroups)))
rfxRel <-  CoxRFX(relData[relData$index %in% whichTrain, names(crGroups)], Surv(relData$time1, relData$time2, relData$status)[relData$index %in% whichTrain], groups=crGroups, which.mu = intersect(mainGroups, unique(crGroups)))
rfxRel$coefficients["transplantRel"] <- 0
rfxCr <- CoxRFX(osData[whichTrain, names(crGroups)], Surv(cr[,1], cr[,2]==2)[whichTrain], groups=crGroups, which.mu = intersect(mainGroups, unique(crGroups)), nu=ifelse(jobIndex==55,1,0))
rfxEs <- CoxRFX(osData[whichTrain, names(crGroups)], Surv(cr[,1], cr[,2]==1)[whichTrain], groups=crGroups, which.mu = NULL)
ix <- tplSplitOs %in% whichTrain
rfxOs <- CoxRFX(dataFrameOsTD[ix,whichRFXOsTDGG], osTD[ix], groups[whichRFXOsTDGG], which.mu=mainGroups) ## allow only the main groups to have mean different from zero..
xx <- 0:2000
coxphPrs <- coxph(Surv(time1, time2, status)~ pspline(time0, df=10), data=data.frame(prdData, time0=as.numeric(clinicalData$Recurrence_date-clinicalData$CR_date)[prdData$index])[prdData$index %in% whichTrain,]) #avoiding data singularity in split 55
tdPrmBaseline <- exp(predict(coxphPrs, newdata=data.frame(time0=xx[-1])))
coxphOs <- coxph(Surv(time1, time2, status)~ pspline(time0, df=10), data=data.frame(osData, time0=pmin(500,cr[osData$index,1]))[osData$index %in% whichTrain,])
tdOsBaseline <- exp(pmin(predict(coxphOs, newdata=data.frame(time0=500)),predict(coxphOs, newdata=data.frame(time0=xx[-1])))) ## cap predictions at induction length 500 days.

dataTD <- data[tplSplitOs, ]
dataTD$transplantCR1[1:nrow(data)] <- 0
dataTD$transplantRel[1:nrow(data)] <- 0
multiRfx5 <- MultiRFX5(rfxEs, rfxCr, rfxNrs, rfxRel, rfxPrs, dataTD[!trainIdxTD,], tdPrmBaseline = tdPrmBaseline, tdOsBaseline = tdOsBaseline, x=2000)

save(rfxEs, rfxCr, rfxEs, rfxNrs, rfxPrs, rfxRel, rfxOs, multiRfx5, coxBICOs, coxBICOsTD, coxAICOs, coxAICOsTD, coxCPSSOs, coxRFXOs, coxRFXOsTD, coxRFXOsGGc, coxRFXOsTDGGc, rForestOsTrain, file=paste0("cv100/",jobIndex,".RData"))
```

Gathering results and computing predictions

```
replicates <- 100
allModelsCvTdPredictions <- mclapply(1:replicates, function(foo) try({
            e <- new.env()
            load(paste0("../code/cv100/",foo,".RData"), envir=e)
            set.seed(foo)
            x <- list(
                    BIC=e$coxBICOsTD,
                    AIC=e$coxAICOsTD,
                    RFX=e$coxRFXOsTD,
                    RFXgg=e$coxRFXOsTDGGc
            )
            trainIdx <- sample(1:nrow(dataFrame)%%5 +1 )[tplSplitOs]!=1 ## sample 1/5
            pred <- cbind(ELN=c(4,1,3,2)[clinicalData$M_Risk[tplSplitOs][!trainIdx]],
                    sapply(x, function(y){
                                predictAllModels(y, newdata=dataFrameOsTD[!trainIdx,])
                            }))
            pred <- cbind(pred, mRFX1yr=colSums(e$multiRfx5[365,1:3,]), mRFX3yr=colSums(e$multiRfx5[3*365,1:3,]),
mRFX5yr=colSums(e$multiRfx5[5*365,1:3,]))
            return(pred)
        }), mc.cores=4)
```

Harrel's C

```
allModelsCvTdC <- sapply(1:replicates, function(foo){
            x <- allModelsCvTdPredictions[[foo]]
            set.seed(foo)
            trainIdx <- sample(1:nrow(dataFrame)%%5 +1 )[tplSplitOs]!=1 ## sample 1/5
            apply(x, 2 , function(p){
                        survConcordance(osYrTD[!trainIdx,] ~ p)$concordance
                    })
        })
apply(allModelsCvTdC,1,quantile)
```

```
##            ELN       BIC       AIC       RFX     RFXgg   mRFX1yr   mRFX3yr   mRFX5yr
## 0%   0.5779764 0.6475790 0.6462563 0.6617532 0.6662437 0.6686109 0.6729061 0.6733210
## 25%  0.6199073 0.6939107 0.6910616 0.7015326 0.7070262 0.6937961 0.7050696 0.7050183
## 50%  0.6369861 0.7048814 0.7046267 0.7175752 0.7211409 0.7070192 0.7178056 0.7178882
## 75%  0.6481770 0.7196140 0.7164776 0.7291661 0.7329534 0.7215234 0.7331457 0.7326754
## 100% 0.6805547 0.7492823 0.7440624 0.7633471 0.7621882 0.7538965 0.7604133 0.7600605
```

```
par(mar=c(3,3,1,1),bty="n", mgp=c(2,.5,0), las=2)
r <- sapply(as.data.frame(lapply(as.data.frame(t(apply(-allModelsCvTdC,2,rank))),factor, levels=1:nrow(allModelsC
vTdC))),table)
o <- order(apply(allModelsCvTdC,1,median))
boxplot(t(allModelsCvTdC[o,]), notch=TRUE, ylab="Concordance", staplewex=0, lty=1, pch=16, xaxt="n")
rotatedLabel(1:nrow(allModelsCvTdC), rep(par("usr")[3],nrow(allModelsCvTdC)), rownames(allModelsCvTdC)[o])
```

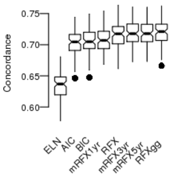


```
par(mar=c(3,3,3,1), xpd=NA, las=2, mgp=c(2,.5,0))
clr <- brewer.pal(nrow(allModelsCvTdC),"PiYG")#set1[c(3,2,4,1,5,7)]
barplot(r[,o]/replicates, col=clr[1:ncol(allModelsCvTdC)], ylab="Fraction", names.arg=rep("",ncol(r))) -> b
rotatedLabel(b, rep(par("usr")[3],ncol(allModelsCvTdC)), colnames(allModelsCvTdC)[o])
legend(par("usr")[1],1.5, fill=clr[1:nrow(allModelsCvTdC)], legend=1:nrow(allModelsCvTdC), bty="n", border=NA, ho
riz=TRUE, title="Rank")
```

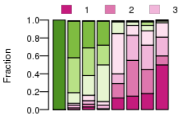

4.4.2 Inter-study CV

```r
allModelsTrial <- mclapply(levels(clinicalData$Study), function(foo){
            #set.seed(foo)
            trainIdx <- clinicalData$Study != foo
            c <- coxph(os[trainIdx] ~ 1, data=dataFrame[trainIdx,mainIdxOs])
            scopeStep <- as.formula(paste("os[trainIdx] ~", paste(colnames(dataFrame)[mainIdxOs], collapse="+")))
            coxBICOsTrain <- step(c, scope=scopeStep, k = log(sum(trainIdx)), trace=0)
            coxAICOsTrain <- step(coxBICOsTrain, scope=scopeStep, k = 2, trace=0)
            coxCPSSOsTrain <- CoxCPSSInteractions(dataFrame[!is.na(os) & trainIdx, mainIdxOs], na.omit(os[trainId
x]), bootstrap.samples=50, scope = which(groups %in% scope))
            w <- colnames(dataFrame[mainIdxOs])
            w <- setdiff(w, names(which(colSums(dataFrame[trainIdx,w])==0)))
            coxRFXOsTrain <- CoxRFX(dataFrame[trainIdx,w], os[trainIdx], groups=groups[w], nu = if(foo=="AMLSG070
4") 1 else 0) # add prior for 0704 (just one group member)
            coxRFXOsTrain$Z <- NULL
            w <- whichRFXOsGG
            w <- setdiff(w, which(colSums(dataFrame[trainIdx,w])==0))
            coxRFXOsGGc <- CoxRFX(dataFrame[trainIdx,w], os[trainIdx], groups=groups[w], which.mu=mainGroups, nu
= if(foo=="AMLSG0704") 1 else 0)
            coxRFXOsGGc$Z <- NULL
            rForestOsTrain <- rfsrc(Surv(time, status) ~.,data= cbind(time = os[,1], status = os[,2], dataFrame[,
mainIdxOs])[trainIdx,], ntree=100, importance="none")
            return(list(
                            BIC=coxBICOsTrain,
                            AIC=coxAICOsTrain,
                            CPSS=coxCPSSOsTrain,
                            RFX=coxRFXOsTrain,
                            RFXgg=coxRFXOsGGc,
                            rForest=rForestOsTrain
                    ))
        }, mc.cores=3)
names(allModelsTrial) <- levels(clinicalData$Study)

allModelsTrialPredictions <- mclapply(names(allModelsTrial), function(foo){
            x <- allModelsTrial[[foo]]
            trainIdx <- clinicalData$Study != foo
            cbind(ELN=c(4,1,3,2)[clinicalData$M_Risk[!trainIdx]],
                    sapply(x, function(y){
                                predictAllModels(y, newdata=dataFrame[!trainIdx,])
                            }))
        }, mc.cores=10)
names(allModelsTrialPredictions) <- names(allModelsTrial)

allModelsTrialC <- sapply(names(allModelsTrial), function(foo){
            trainIdx <- clinicalData$Study != foo
            apply(allModelsTrialPredictions[[foo]], 2 , function(p){
                        unlist( survConcordance(osYr[!trainIdx,] ~ p)[c("concordance","std.err")])
                    })
        }, simplify="array")

allModelsTrialC
```

```
## , , AMLHD98A
## 
##                                ELN        BIC        AIC       CPSS        RFX      RFXgg    rForest
## concordance.concordant 0.65602221 0.65776049 0.67425763 0.65551738 0.69185458 0.69107788 0.66051312
## std.err.std(c-d)       0.01533222 0.01554614 0.01554614 0.01554614 0.01554614 0.01554614 0.01554614
## 
## , , AMLHD98B
## 
##                                ELN        BIC        AIC       CPSS        RFX      RFXgg    rForest
## concordance.concordant 0.60724349 0.67239030 0.67552467 0.68635868 0.68138457 0.66578087 0.64172799
## std.err.std(c-d)       0.02607456 0.02595602 0.02595607 0.02595607 0.02595607 0.02595607 0.02595607
## 
## , , AMLSG0704
## 
##                                ELN        BIC        AIC       CPSS        RFX      RFXgg    rForest
## concordance.concordant 0.63806151 0.66111737 0.65393399 0.66226428 0.68447415 0.68294156 0.66402777
## std.err.std(c-d)       0.01552632 0.01550206 0.01550206 0.01550206 0.01550206 0.01550206 0.01550206
```

4.4.2.1 Time-dependent

```
> allModelsTrialC
, , AMLHD98A

                              ELN        BIC        AIC       CPSS        RFX      RFXgg    rForest
concordance.concordant 0.65602221 0.66979004 0.67728987 0.66017759 0.69383051 0.69283633 0.66488129
std.err.std(c-d)       0.01533222 0.01554614 0.01554614 0.01554614 0.01554614 0.01554614 0.01554614

, , AMLHD98B

                              ELN        BIC        AIC       CPSS        RFX      RFXgg    rForest
concordance.concordant 0.60724349 0.67239030 0.67552467 0.68676751 0.68056691 0.66578087 0.64636141
std.err.std(c-d)       0.02607456 0.02595602 0.02595607 0.02595607 0.02595607 0.02595607 0.02595607

, , AMLSG0704

                              ELN        BIC        AIC       CPSS        RFX      RFXgg    rForest
concordance.concordant 0.63806151 0.66111737 0.65393399 0.66226428 0.68528104 0.68320545 0.67191401
std.err.std(c-d)       0.01552632 0.01550206 0.01550206 0.01550206 0.01550206 0.01550206 0.01550206
```

[outputs of the rerun (2021)]

```
allModelsTrialTD <- mclapply(levels(clinicalData$Study), function(foo){
            #set.seed(foo)
            trainIdxTD <- clinicalData$Study[tplSplitOs] != foo
            c <- coxph(osTD[trainIdxTD] ~ 1, data=dataFrameOsTD[trainIdxTD,mainIdxOsTD])
            scopeStep <- as.formula(paste("osTD[trainIdx] ~", paste(colnames(dataFrameOsTD)[mainIdxOsTD], collaps
e="+")))
            coxBICOsTrain <- step(c, scope=scopeStep, k = log(sum(trainIdxTD)), trace=0)
            coxAICOsTrain <- step(coxBICOsTrain, scope=scopeStep, k = 2, trace=0)
            coxRFXOsTrain <- CoxRFX(dataFrameOsTD[trainIdxTD,mainIdxOsTD], osTD[trainIdxTD], groups=groups[mainId
xOsTD], nu = if(foo=="AMLSG0704") 1 else 0)
            coxRFXOsTrain$Z <- NULL
            coxRFXOsGGc <- CoxRFX(dataFrameOsTD[trainIdxTD,whichRFXOsTDGG], osTD[trainIdxTD], groups=groups[which
RFXOsTDGG], which.mu=mainGroups, nu = if(foo=="AMLSG0704") 1 else 0)
            coxRFXOsGGc$Z <- NULL

            return(list(
                            BIC=coxBICOsTrain,
                            AIC=coxAICOsTrain,
                            RFX=coxRFXOsTrain,
                            RFXgg=coxRFXOsGGc
                                                            ))
        }, mc.cores=3)
names(allModelsTrialTD) <- levels(clinicalData$Study)

allModelsTrialTdPredictions <- mclapply(names(allModelsTrialTD), function(foo){
            x <- allModelsTrialTD[[foo]]
            trainIdxTD <<- clinicalData$Study[tplSplitOs] != foo
            pred <- cbind(ELN=c(4,1,3,2)[clinicalData$M_Risk[tplSplitOs][!trainIdxTD]],
                    sapply(x, function(y){
                                predictAllModels(y, newdata=dataFrameOsTD[!trainIdxTD,])
                            }))
            whichTrain <<- which(trainIdxTD[1:nrow(dataFrame)])
            rfxNrs <- CoxRFX(nrdData[nrdData$index %in% whichTrain, names(crGroups)], Surv(nrdData$time1, nrdData
$time2, nrdData$status)[nrdData$index %in% whichTrain], groups=crGroups, which.mu = intersect(mainGroups, unique(
crGroups)))
            rfxNrs$coefficients["transplantRel"] <- 0
            rfxPrs <-  CoxRFX(prdData[prdData$index %in% whichTrain, names(crGroups)], Surv(prdData$time1, prdDat
a$time2, prdData$status)[prdData$index %in% whichTrain], groups=crGroups, nu=1, which.mu = intersect(mainGroups,
unique(crGroups)))
            rfxRel <-  CoxRFX(relData[relData$index %in% whichTrain, names(crGroups)], Surv(relData$time1, relDat
a$time2, relData$status)[relData$index %in% whichTrain], groups=crGroups, which.mu = intersect(mainGroups, unique
(crGroups)))
            rfxRel$coefficients["transplantRel"] <- 0
            rfxCr <- CoxRFX(osData[whichTrain, names(crGroups)], Surv(cr[,1], cr[,2]==2)[whichTrain], groups=crGr
oups, which.mu = NULL)#intersect(mainGroups, unique(crGroups)))
            rfxEs <- CoxRFX(osData[whichTrain, names(crGroups)], Surv(cr[,1], cr[,2]==1)[whichTrain], groups=crGr
oups, which.mu = NULL)
            ix <- tplSplitOs %in% whichTrain
            rfxOs <- CoxRFX(dataFrameOsTD[ix,whichRFXOsTDGG], osTD[ix], groups[whichRFXOsTDGG], which.mu=mainGrou
ps) ## allow only the main groups to have mean different from zero..
            xx <- 0:2000
            coxphPrs <- coxph(Surv(time1, time2, status)~ pspline(time0, df=10), data=data.frame(prdData, time0=a
s.numeric(clinicalData$Recurrence_date-clinicalData$CR_date)[prdData$index])[prdData$index %in% whichTrain,])
            tdPrmBaseline <- exp(predict(coxphPrs, newdata=data.frame(time0=xx[-1])))
            coxphOs <- coxph(Surv(time1, time2, status)~ pspline(time0, df=10), data=data.frame(osData, time0=pmi
n(500,cr[osData$index,1]))[osData$index %in% whichTrain,])
            tdOsBaseline <- exp(pmin(predict(coxphOs, newdata=data.frame(time0=500)),predict(coxphOs, newdata=dat
a.frame(time0=xx[-1])))) ## cap predictions at induction length 500 days.

            dataTD <- data[tplSplitOs, ]
            dataTD$transplantCR1[1:nrow(data)] <- 0
            dataTD$transplantRel[1:nrow(data)] <- 0
            multiRfx5 <- MultiRFX5(rfxEs, rfxCr, rfxNrs, rfxRel, rfxPrs, dataTD[!trainIdxTD,], tdPrmBaseline = td
PrmBaseline, tdOsBaseline = tdOsBaseline, x=2000)

            pred <- cbind(pred, mRFX1yr=colSums(multiRfx5[365,1:3,]), mRFX3yr=colSums(multiRfx5[3*365,1:3,]), mRF
X5yr=colSums(multiRfx5[5*365,1:3,]))
            return(pred)

        }, mc.cores=3)
names(allModelsTrialTdPredictions) <- names(allModelsTrialTD)

allModelsTrialTdC <- sapply(names(allModelsTrialTD), function(foo){
            trainIdx <- clinicalData$Study[tplSplitOs] != foo
            apply(allModelsTrialTdPredictions[[foo]], 2 , function(p){
                        unlist( survConcordance(osYrTD[!trainIdx,] ~ p)[c("concordance","std.err")])
                    })
        }, simplify="array")

allModelsTrialTdC
```

The first mclapply() returns warning message and causes errors in the following steps.
Warning message:
In mclapply(names(allModelsTrialTD), function(foo) { :
all scheduled cores encountered errors in user code

```
> allModelsTrialTdC <- sapply(names(allModelsTrialTD), function(foo){
+                       trainIdx <- clinicalData$Study[tplSplitOs] != foo
+                       apply(allModelsTrialTdPredictions[[foo]], 2 , function(p){
+                                               unlist( survConcordance(osYrTD[!trainIdx,] ~ p)[c("concordance","std.err")])
+                                       })
+               }, simplify="array")
Error in apply(allModelsTrialTdPredictions[[foo]], 2, function(p) { :
  dim(X) must have a positive length
> allModelsTrialTdC
Error: object 'allModelsTrialTdC' not found
```

```
## , , AMLHD98A
##
##                                  ELN        BIC        AIC        RFX      RFXgg    mRFX1yr    mRFX3yr    mRFX5yr
## concordance.concordant 0.64679319 0.67046248 0.68279982 0.70245030 0.70055621 0.70234158 0.70868764 0.70878492
## std.err.std(c-d)       0.01579591 0.01588897 0.01588897 0.01588897 0.01588897 0.01588897 0.01588897 0.01588897
##
## , , AMLHD98B
##
##                                  ELN        BIC        AIC        RFX      RFXgg    mRFX1yr    mRFX3yr    mRFX5yr
## concordance.concordant 0.60665156 0.68014926 0.66209102 0.68314786 0.66875458 0.67142000 0.67495169 0.67088692
## std.err.std(c-d)       0.02622793 0.02608867 0.02608872 0.02608872 0.02608872 0.02608872 0.02608872 0.02608872
##
## , , AMLSG0704
##
##                                  ELN        BIC        AIC        RFX      RFXgg    mRFX1yr    mRFX3yr    mRFX5yr
## concordance.concordant 0.63467885 0.67383609 0.66879788 0.68775965 0.68597782 0.68237127 0.69286694 0.69053722
## std.err.std(c-d)       0.01583684 0.01581224 0.01581224 0.01581224 0.01581224 0.01581224 0.01581224 0.01581224
```

4.4.3 TCGA validation

4.4.3.1 Fit models

Fit a single tree (T. Therneau, Atkinson, and Ripley 2014) and a random forest model (Ishwaran et al. 2008).

```
library(rpart)
library(randomForestSRC)
tree <- rpart(os ~ ., data=dataFrame[mainIdxOs & osIdx])
plot(tree)
text(tree)
```

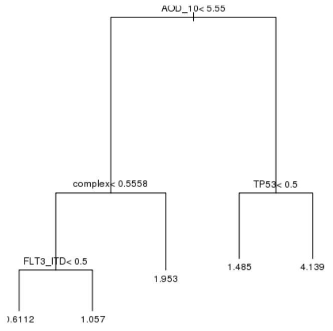


```
survConcordance(na.omit(os)~predict(tree))
```

```
## Call:
## survConcordance(formula = na.omit(os) ~ predict(tree))
##
##   n= 1540
## Concordance= 0.636895 se= 0.00915092
## concordant discordant  tied.risk  tied.time   std(c-d)
##  466728.00  206536.00  277070.00     432.00   17392.86
```

Random forest

```
rForest <- rfsrc(Surv(time, status) ~.,data= cbind(time = os[,1], status = os[,2], dataFrame[,mainIdxOs & osIdx])
, ntree=100)
boxplot(rForest$importance ~ droplevels(groups[mainIdxOs & osIdx]), border= colGroups[mainGroups], staplewex=0, p
ch=16, cex=0.75, ylab="RSF importance", lty=1, xaxt="n")
rotatedLabel(labels=mainGroups)
```

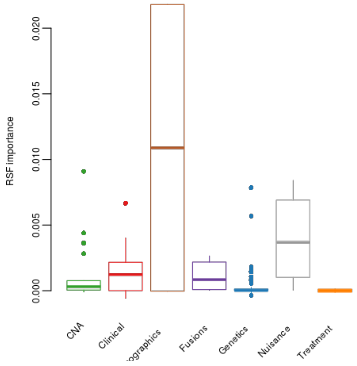

```
rForestVimp <- sapply(mainGroups, function(g) vimp(rForest, colnames(dataFrame)[which(groups==g)]))

survConcordance(na.omit(os)~predict(rForest, importance="none")$predicted)
```

```
## Call:
## survConcordance(formula = na.omit(os) ~ predict(rForest, importance = "none")$predicted)
##
##   n= 1540
## Concordance= 0.8960502 se= 0.01003199
## concordant discordant  tied.risk  tied.time   std(c-d)
##  851547.00   98787.00       0.00     432.00   19067.49
```

Complementary pairs stability selection with interaction terms

```
set.seed(42)
coxCPSSIntOs <- CoxCPSSInteractions(dataFrame[!is.na(os),groups %in% mainGroups & osIdx], na.omit(os), bootstrap.
samples=50, scope = which(groups %in% scope))
```

```
## ..................................................
## ..................................................
```

```
selectedIntOs <- names(which(coxCPSSIntOs$Pi > 0.8))
coxCPSSIntOs
```

```
##
## Stability selection:
## Variable         P[select] P-value adj. P
## NPM1             0.98      0.00121 0.0155
## TP53             0.98      0.00121 0.0155
## CEBPA_bi         0.92      0.00413 0.0327
## FLT3_ITD         1.00      0.00038 0.0099
## minus7           0.92      0.00413 0.0327
## t_15_17          0.97      0.00164 0.0188
## inv16_t16_16     0.98      0.00121 0.0155
## complex          1.00      0.00038 0.0099
## Date_1000        1.00      0.00079 0.0155
## ATRA             0.84      0.00084 0.0874
## AOD_10           1.00      0.00038 0.0099
## Performance_ECOG 0.96      0.00210 0.0216
## wbc_100          1.00      0.00038 0.0099
## LDH_1000         0.94      0.00307 0.0287
##
## Corresponding coxph:
## Call:
## coxph(formula = na.omit(os) ~ NPM1 + TP53 + CEBPA_bi + FLT3_ITD +
##     minus7 + t_15_17 + inv16_t16_16 + complex + Date_1000 + ATRA +
##     AOD_10 + Performance_ECOG + wbc_100 + LDH_1000, data = dataFrame[!is.na(os),
##     groups %in% mainGroups & osIdx])
##
##
##                     coef exp(coef) se(coef)     z       p
## NPM1             -0.4847     0.616   0.0830 -5.84 5.3e-09
## TP53              0.7601     2.139   0.1426  5.33 9.7e-08
## CEBPA_bi         -0.9735     0.378   0.2196 -4.43 9.3e-06
## FLT3_ITD          0.4619     1.587   0.0856  5.39 6.9e-08
## minus7            0.4963     1.643   0.1333  3.72 2.0e-04
## t_15_17          -1.2482     0.287   0.2466 -5.06 4.2e-07
## inv16_t16_16     -1.2180     0.296   0.2334 -5.22 1.8e-07
## complex           0.5532     1.739   0.1214  4.56 5.1e-06
## Date_1000        -0.1510     0.860   0.0311 -4.86 1.2e-06
## ATRA             -0.1936     0.824   0.0801 -2.42 1.6e-02
## AOD_10            0.3062     1.358   0.0318  9.63 0.0e+00
## Performance_ECOG  0.1115     1.118   0.0542  2.06 4.0e-02
## wbc_100           0.4852     1.625   0.0696  6.97 3.2e-12
## LDH_1000          0.0935     1.098   0.0527  1.77 7.6e-02
##
## Likelihood ratio test=509  on 14 df, p=0  n= 1540, number of events= 904
```

Stepwise model selection by BIC

```
c <- coxph(os ~ 1, data=dataFrame[,mainIdxOs & osIdx])
scopeStep <- as.formula(paste("os ~", paste(colnames(dataFrame)[mainIdxOs& osIdx], collapse="+")))
coxBICOs <- step(c, scope=scopeStep, k = log(sum(trainIdx)), trace=0)
summary(coxBICOs)
```

```
## Call:
## coxph(formula = os ~ AOD_10 + complex + wbc_100 + TP53 + inv16_t16_16 +
##     Date_1000 + t_15_17 + CEBPA_bi + NPM1 + FLT3_ITD + inv3_t3_3 +
##     SFRS2, data = dataFrame[, mainIdxOs & osIdx])
##
##   n= 1540, number of events= 904
##
##                  coef exp(coef) se(coef)      z Pr(>|z|)
## AOD_10        0.27345   1.31449  0.03075  8.892  < 2e-16 ***
## complex       0.72285   2.06029  0.11770  6.142 8.17e-10 ***
## wbc_100       0.50452   1.65618  0.06377  7.912 2.55e-15 ***
## TP53          0.82568   2.28344  0.13893  5.943 2.80e-09 ***
## inv16_t16_16 -1.16935   0.31057  0.23335 -5.011 5.41e-07 ***
## Date_1000    -0.17136   0.84252  0.02938 -5.833 5.45e-09 ***
## t_15_17      -1.20870   0.29859  0.24607 -4.912 9.01e-07 ***
## CEBPA_bi     -0.93085   0.39422  0.21984 -4.234 2.29e-05 ***
## NPM1         -0.45461   0.63470  0.08310 -5.471 4.48e-08 ***
## FLT3_ITD      0.51234   1.66919  0.08554  5.990 2.10e-09 ***
## inv3_t3_3     1.14923   3.15576  0.22911  5.016 5.27e-07 ***
## SFRS2         0.38488   1.46943  0.12731  3.023   0.0025 **
## ---
## Signif. codes:  0 '***' 0.001 '**' 0.01 '*' 0.05 '.' 0.1 ' ' 1
##
##              exp(coef) exp(-coef) lower .95 upper .95
## AOD_10          1.3145     0.7608    1.2376    1.3962
## complex         2.0603     0.4854    1.6359    2.5948
## wbc_100         1.6562     0.6038    1.4616    1.8767
## TP53            2.2834     0.4379    1.7391    2.9981
## inv16_t16_16    0.3106     3.2199    0.1966    0.4907
## Date_1000       0.8425     1.1869    0.7954    0.8925
## t_15_17         0.2986     3.3491    0.1843    0.4836
## CEBPA_bi        0.3942     2.5367    0.2562    0.6066
## NPM1            0.6347     1.5756    0.5393    0.7470
## FLT3_ITD        1.6692     0.5991    1.4115    1.9739
## inv3_t3_3       3.1558     0.3169    2.0141    4.9445
## SFRS2           1.4694     0.6805    1.1449    1.8859
##
## Concordance= 0.707  (se = 0.01 )
## Rsquare= 0.282   (max possible= 1 )
## Likelihood ratio test= 509.3  on 12 df,   p=0
## Wald test            = 528.4  on 12 df,   p=0
## Score (logrank) test = 589.2  on 12 df,   p=0
```

With AIC

```
coxAICOs <- step(c, scope= scopeStep, k = 2, trace=0)
summary(coxAICOs)
```

```
## Call:
## coxph(formula = os ~ AOD_10 + complex + wbc_100 + TP53 + inv16_t16_16 +
##     Date_1000 + t_15_17 + CEBPA_bi + NPM1 + FLT3_ITD + inv3_t3_3 +
##     SFRS2 + ATRA + BM_Blasts_100 + IDH2_p172 + CEBPA_mono + RAD21 +
##     Performance_ECOG + KRAS + sAML + minus7 + VPA + MissingCyto +
##     BRAF + HB_10 + platelet_100 + DNMT3A + ZRSR2 + plus21 + ASXL1 +
##     FLT3_other + IDH1 + plus22 + mono17_17p_abn17p + PTEN + t_v_11 +
##     minus18_18q, data = dataFrame[, mainIdxOs & osIdx])
##
##   n= 1540, number of events= 904
##
##                        coef  exp(coef)   se(coef)      z Pr(>|z|)
## AOD_10             2.917e-01  1.339e+00  3.274e-02  8.907  < 2e-16 ***
## complex            5.447e-01  1.724e+00  1.357e-01  4.015 5.95e-05 ***
## wbc_100            4.113e-01  1.509e+00  6.786e-02  6.060 1.36e-09 ***
## TP53               7.884e-01  2.200e+00  1.552e-01  5.079 3.79e-07 ***
## inv16_t16_16      -1.353e+00  2.583e-01  2.520e-01 -5.370 7.86e-08 ***
## Date_1000         -1.730e-01  8.412e-01  3.205e-02 -5.396 6.80e-08 ***
## t_15_17           -1.321e+00  2.669e-01  2.544e-01 -5.192 2.08e-07 ***
## CEBPA_bi          -1.067e+00  3.442e-01  2.251e-01 -4.739 2.15e-06 ***
## NPM1              -5.264e-01  5.907e-01  9.188e-02 -5.730 1.01e-08 ***
## FLT3_ITD           5.475e-01  1.729e+00  8.774e-02  6.240 4.37e-10 ***
## inv3_t3_3          1.033e+00  2.809e+00  2.686e-01  3.845  0.00012 ***
## SFRS2              3.922e-01  1.480e+00  1.303e-01  3.009  0.00262 **
## ATRA              -2.122e-01  8.088e-01  8.106e-02 -2.618  0.00885 **
## BM_Blasts_100      4.422e-01  1.556e+00  1.516e-01  2.918  0.00353 **
## IDH2_p172         -5.463e-01  5.791e-01  2.516e-01 -2.171  0.02989 *
## CEBPA_mono        -4.348e-01  6.474e-01  1.946e-01 -2.235  0.02545 *
## RAD21             -3.781e-01  6.851e-01  2.196e-01 -1.722  0.08510 .
## Performance_ECOG   1.542e-01  1.167e+00  5.599e-02  2.753  0.00590 **
## KRAS               3.733e-01  1.453e+00  1.555e-01  2.400  0.01639 *
## sAML               3.078e-01  1.360e+00  1.583e-01  1.945  0.05181 .
## minus7             3.358e-01  1.399e+00  1.537e-01  2.185  0.02892 *
## VPA                3.359e-01  1.399e+00  1.267e-01  2.652  0.00801 **
## MissingCyto        2.543e-01  1.290e+00  1.173e-01  2.168  0.03015 *
## BRAF               9.975e-01  2.711e+00  3.848e-01  2.592  0.00954 **
## HB_10              3.796e-01  1.462e+00  1.839e-01  2.064  0.03906 *
```

```
## platelet_100      -9.481e-02  9.095e-01  4.111e-02 -2.306  0.02111 *
## DNMT3A             1.672e-01  1.182e+00  8.820e-02  1.896  0.05803 .
## ZRSR2              7.085e-01  2.031e+00  3.138e-01  2.258  0.02395 *
## plus21             3.840e-01  1.468e+00  2.065e-01  1.859  0.06298 .
## ASXL1              2.545e-01  1.290e+00  1.429e-01  1.781  0.07485 .
## FLT3_other        -2.877e-01  7.500e-01  1.680e-01 -1.712  0.08683 .
## IDH1               2.226e-01  1.249e+00  1.310e-01  1.699  0.08938 .
## plus22             4.735e-01  1.606e+00  2.875e-01  1.647  0.09960 .
## mono17_17p_abn17p  3.082e-01  1.361e+00  1.763e-01  1.748  0.08046 .
## PTEN              -1.359e+01  1.249e-06  8.101e+02 -0.017  0.98661
## t_v_11             3.122e-01  1.366e+00  1.924e-01  1.623  0.10466
## minus18_18q       -3.497e-01  7.049e-01  2.363e-01 -1.480  0.13887
## ---
## Signif. codes:  0 '***' 0.001 '**' 0.01 '*' 0.05 '.' 0.1 ' ' 1
##
##                   exp(coef) exp(-coef) lower .95 upper .95
## AOD_10            1.339e+00  7.470e-01    1.2554    1.4274
## complex           1.724e+00  5.800e-01    1.3216    2.2494
## wbc_100           1.509e+00  6.628e-01    1.3208    1.7233
## TP53              2.200e+00  4.546e-01    1.6228    2.9819
## inv16_t16_16      2.583e-01  3.871e+00    0.1576    0.4234
## Date_1000         8.412e-01  1.189e+00    0.7900    0.8957
## t_15_17           2.669e-01  3.747e+00    0.1621    0.4394
## CEBPA_bi          3.442e-01  2.905e+00    0.2214    0.5350
## NPM1              5.907e-01  1.693e+00    0.4934    0.7073
## FLT3_ITD          1.729e+00  5.784e-01    1.4558    2.0533
## inv3_t3_3         2.809e+00  3.560e-01    1.6593    4.7552
## SFRS2             1.480e+00  6.756e-01    1.1465    1.9110
## ATRA              8.088e-01  1.236e+00    0.6900    0.9481
## BM_Blasts_100     1.556e+00  6.426e-01    1.1562    2.0945
## IDH2_p172         5.791e-01  1.727e+00    0.3537    0.9482
## CEBPA_mono        6.474e-01  1.545e+00    0.4421    0.9480
## RAD21             6.851e-01  1.460e+00    0.4455    1.0537
## Performance_ECOG  1.167e+00  8.571e-01    1.0454    1.3020
## KRAS              1.453e+00  6.885e-01    1.0708    1.9702
## sAML              1.360e+00  7.351e-01    0.9976    1.8552
## minus7            1.399e+00  7.148e-01    1.0351    1.8908
## VPA               1.399e+00  7.147e-01    1.0916    1.7935
## MissingCyto       1.290e+00  7.754e-01    1.0247    1.6229
## BRAF              2.711e+00  3.688e-01    1.2754    5.7645
## HB_10             1.462e+00  6.842e-01    1.0192    2.0961
## platelet_100      9.095e-01  1.099e+00    0.8391    0.9859
## DNMT3A            1.182e+00  8.460e-01    0.9943    1.4050
## ZRSR2             2.031e+00  4.924e-01    1.0980    3.7566
## plus21            1.468e+00  6.811e-01    0.9794    2.2008
## ASXL1             1.290e+00  7.753e-01    0.9748    1.7068
## FLT3_other        7.500e-01  1.333e+00    0.5396    1.0425
## IDH1              1.249e+00  8.005e-01    0.9663    1.6150
## plus22            1.606e+00  6.228e-01    0.9139    2.8206
## mono17_17p_abn17p 1.361e+00  7.348e-01    0.9633    1.9228
## PTEN              1.249e-06  8.008e+05    0.0000       Inf
## t_v_11            1.366e+00  7.318e-01    0.9372    1.9924
## minus18_18q       7.049e-01  1.419e+00    0.4436    1.1201
##
## Concordance= 0.724  (se = 0.01 )
## Rsquare= 0.329   (max possible= 1 )
## Likelihood ratio test= 613.5  on 37 df,   p=0
## Wald test            = 608.7  on 37 df,   p=0
## Score (logrank) test = 685.1  on 37 df,   p=0
```

Time-dep AIC and BIC, including allografts

```
c <- coxph(osTD ~ 1, data=dataFrameOsTD[mainIdxOsTD])
scopeStep <- as.formula(paste("osTD ~", paste(colnames(dataFrameOsTD)[mainIdxOsTD], collapse="+")))
coxBICOsTD <- step(c, scope=scopeStep, k = log(nrow(dataFrame)), trace=0)
coxAICOsTD <- step(coxBICOsTD, scope=scopeStep, k = 2, trace=0)
```

4.4.3.2 TCGA data

Load data

Unfortunately, data ***TCGA_Clinical.txt*** was not provided, and hence we could not rerun sections 4.4.3 and 4.4.4.

```
tcgaClinical <- read.table("../data/TCGA_Clinical.txt", sep="\t", header=TRUE)
tcgaGenetic <- read.table("../data/TCGA_Genetic.txt", sep="\t", header=TRUE)
tcgaGenetic$TCGA_ID <- factor(as.character(tcgaGenetic$TCGA_ID), levels = levels(tcgaClinical$TCGA_ID))
g <- as.character(tcgaGenetic$Hugo_Symbol)
g[tcgaGenetic$Hugo_Symbol=="FLT3" & tcgaGenetic$Variant_Type == 'INS'] <- "FLT3_ITD"
g[tcgaGenetic$Hugo_Symbol=="FLT3" & tcgaGenetic$Variant_Type == 'SNP'] <- "FLT3_TKD"
tcgaMutation <- (table(tcgaGenetic$TCGA_ID,g)) + 0
t <- data.frame(tcgaMutation[,]>0, CEBPA_mono = tcgaMutation[,"CEBPA"]==1,CEBPA_bi = tcgaMutation[,"CEBPA"]>1,tcg
aClinical[,-c(1,2,4,5,6,13,25)], MakeInteger(tcgaClinical$TypeAML)) + 0
w <- grep("_10+$",colnames(dataFrame), value=TRUE)
f <- as.numeric(sub(".+_","",w))
n <- sub("_10+","",w)
f <- f[n %in% colnames(tcgaClinical)]
n <- n[n %in% colnames(tcgaClinical)]
t[n] <- t[n] / rep(f, each=nrow(t))
colnames(t)[match(n,colnames(t))] <- paste(n,f,sep="_")
rm(w,n,f,g)

tcgaData <- dataFrame[1:nrow(t),]
tcgaData[,] <- NA
w <- intersect(names(t), names(tcgaData))
tcgaData[w] <- t[w]
tcgaData$TPL_os <- NA
tcgaData[groups=="Genetics"][is.na(tcgaData[groups=="Genetics"])] <- 0
tcgaData$MissingCyto <- (tcgaClinical$karyotype == '[Not Available]' )+0
rm(t,w)
tcgaSurvival <- Surv(tcgaClinical$OS/365, tcgaClinical$Status)

tb <- read.xlsx("../data/TCGA_SupplementalTable01.xlsx", 1, colIndex=1:29)
tb <- tb[order(tb$TCGA.Patient.ID),]

tt <- strsplit(as.character(tb$Trnsplt), ", ")
tp <-  strsplit(as.character(tb$Dz.Stat....trnsplt),", ")
tcgaTpl <- t(sapply(1:nrow(tb) , function(i){
                    transplantCR1=0; transplantRel=0
                    if(tt[i] != "0") {
                        a <- tt[i]%in%c("MUD","sib Allo") & !tp[i] %in% c("Refr dz","refr dz","refr dz post induc
tion","xxxxx","aplastic post chemo","0")
                        if(any(a)){
                            if(any(a & tp[i] %in% c("CR1","CR 1"))) transplantCR1 <- 1
                            if(any(a & !tp[i] %in% c("CR1","CR 1"))) transplantRel <- 1
                        }
                    }
                    return(c(transplantCR1=transplantCR1, transplantRel=transplantRel))
                }) )

tcgaData$TPL_os <- tcgaTpl[,"transplantCR1"]
```

Plot mutation frequencies

```
plot(colMeans(dataFrame[groups=="Genetics"])[colnames(tcgaMutation)], colMeans(tcgaMutation))
text(colMeans(dataFrame[groups=="Genetics"])[colnames(tcgaMutation)], colMeans(tcgaMutation), colnames(tcgaMutati
on))
```

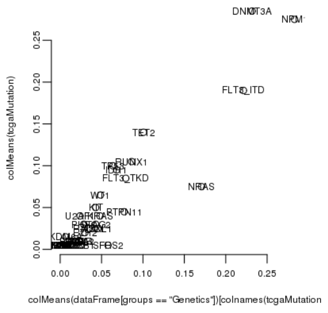


```
cor(colMeans(dataFrame[groups=="Genetics"])[colnames(tcgaMutation)], colMeans(tcgaMutation), use='c')
```

```
## [1] 0.9320114
```

NPM1 survival

```
plot(survfit(tcgaSurvival ~ NPM1, data=tcgaData), col=set1[1:2])
lines(survfit(osYr ~ NPM1, data=dataFrame), col=set1, lty=3,mark=NA)
legend("topright", col=c(set1[1:2],"black","black"), c("NPM1 wt", "NPM1 mut","TCGA","AML"), lty=c(1,1,1,3), bty='
n')
```

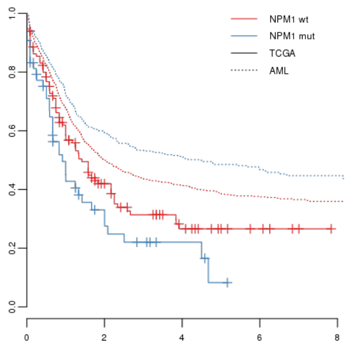

4.4.3.3 Analyse risk

CoxRFX model and covariance-based imputation

```
tcgaRiskRFXOs <- PredictRiskMissing(coxRFXFitOsTDGGc, tcgaData[whichRFXOsTDGG])
survConcordance(tcgaSurvival ~ tcgaRiskRFXOs[,1])
```

```
## Call:
## survConcordance(formula = tcgaSurvival ~ tcgaRiskRFXOs[, 1])
##
##   n=186 (14 observations deleted due to missingness)
## Concordance= 0.7010229 se= 0.02968944
## concordant discordant  tied.risk  tied.time   std(c-d)
##  9046.0000  3858.0000     0.0000   162.0000   766.2249
```

CPSS model

```
tcgaDataImputed <- as.data.frame(ImputeMissing(dataFrame[mainIdxOs], newX=tcgaData[mainIdxOs]))
tcgaRiskCPSSOs <- predict(coxCPSSIntOs, newdata=tcgaDataImputed)
survConcordance(tcgaSurvival ~ tcgaRiskCPSSOs)
```

```
## Call:
## survConcordance(formula = tcgaSurvival ~ tcgaRiskCPSSOs)
##
##   n=186 (14 observations deleted due to missingness)
## Concordance= 0.691646 se= 0.02968944
## concordant discordant  tied.risk  tied.time   std(c-d)
##  8925.0000  3979.0000     0.0000   162.0000   766.2249
```

Blind imputation (mean only)

```
f <- function(X) {X <- sapply(X, poorMansImpute);X[is.na(X)] <- 0; X}
survConcordance(tcgaSurvival ~ predict(coxCPSSIntOs, newdata=as.data.frame(f(tcgaData[mainIdxOs]))))
```

```
## Call:
## survConcordance(formula = tcgaSurvival ~ predict(coxCPSSIntOs,
##     newdata = as.data.frame(f(tcgaData[mainIdxOs]))))
##
##   n=186 (14 observations deleted due to missingness)
## Concordance= 0.687035 se= 0.02968941
## concordant discordant  tied.risk  tied.time   std(c-d)
##  8865.0000  4038.0000     1.0000   162.0000   766.2243
```

Cytogenetic risk

```
survConcordance(tcgaSurvival ~ c(3,1,2)[tcgaClinical$C_Risk])
```

```
## Call:
## survConcordance(formula = tcgaSurvival ~ c(3, 1, 2)[tcgaClinical$C_Risk])
##
##   n=183 (17 observations deleted due to missingness)
## Concordance= 0.5996288 se= 0.02657258
## concordant discordant  tied.risk  tied.time   std(c-d)
##  4741.0000  2272.0000  5378.0000   150.0000   658.5218
```

PINA score (Pastore et al. 2014) for NK AML.

```r
PINAOs <- function(X){
    coef <- c( NPM1=-1.2,
            FLT3_ITD=-.26,
            `NPM1:FLT3_ITD`=.89,
            CEBPA_bi=-1.3,
            wbc_log10=.57,
            age=0.044,
            ecog24=.4)
    x <- cbind(X[,colnames(X) %in% names(coef)], wbc_log10 = log10(100*1e3*pmax(X[,"wbc_100"], 0.001)), age = X[,
"AOD_10"]*10, ecog24 = X[,"Performance_ECOG"]>=2)
    risk <- as.matrix(x[,names(coef)]) %*% coef
    group <- cut(risk, c(min(risk), 4,5.4, max(risk)), labels = c("low","int","high"))
    return(data.frame(risk, group))
}
pinaOs <- PINAOs(dataFrame)
```

```r
nkIdx <- clinicalData$NK == 1
plot(survfit(os[nkIdx] ~ pinaOs[nkIdx,2]), col=rev(set1[1:3]))
```

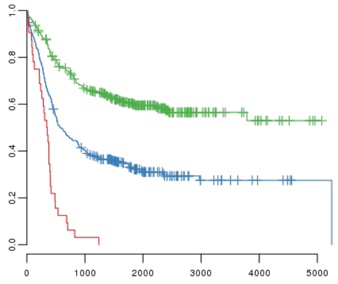


```r
survConcordance(os[nkIdx] ~ pinaOs[nkIdx,1])
```

```
## Call:
## survConcordance(formula = os[nkIdx] ~ pinaOs[nkIdx, 1])
## 
##   n=694 (92 observations deleted due to missingness)
## Concordance= 0.6611361 se= 0.01526872
## concordant discordant  tied.risk  tied.time   std(c-d)
## 123745.000  63425.000      1.000     78.000   5715.723
```

Compared to CPSS (AML data)

```r
survConcordance(os[nkIdx] ~ predict(coxCPSSIntOs, newdata=dataFrame)[nkIdx])
```

```
## Call:
## survConcordance(formula = os[nkIdx] ~ predict(coxCPSSIntOs, newdata = dataFrame)[nkIdx])
## 
##   n=694 (92 observations deleted due to missingness)
## Concordance= 0.6889582 se= 0.01526872
## concordant discordant  tied.risk  tied.time   std(c-d)
## 128953.000  58218.000      0.000     78.000   5715.723
```

And on TCGA data

```r
tcgaPinaOs <- PINAOs(cbind(tcgaDataImputed, `NPM1:FLT3_ITD` = tcgaDataImputed[,"NPM1"]*tcgaDataImputed[,"FLT3_ITD
"]))
tcgaNkIdx <- tcgaClinical$karyotype == "Normal"
survConcordance(tcgaSurvival[tcgaNkIdx] ~ tcgaPinaOs[tcgaNkIdx,1])
```

```
## Call:
## survConcordance(formula = tcgaSurvival[tcgaNkIdx] ~ tcgaPinaOs[tcgaNkIdx, 
##     1])
## 
##   n=96 (6 observations deleted due to missingness)
## Concordance= 0.6335756 se= 0.04038263
## concordant discordant  tied.risk  tied.time   std(c-d)
##  2179.0000  1260.0000     1.0000    29.0000   277.8325
```

```r
survConcordance(tcgaSurvival[tcgaNkIdx] ~ tcgaRiskCPSSOs[tcgaNkIdx])
```

```
## Call:
## survConcordance(formula = tcgaSurvival[tcgaNkIdx] ~ tcgaRiskCPSSOs[tcgaNkIdx])
##
##   n=96 (6 observations deleted due to missingness)
## Concordance= 0.6575581 se= 0.04038309
## concordant discordant  tied.risk  tied.time   std(c-d)
##  2262.0000  1178.0000     0.0000    29.0000   277.8357
```

ELN score (Döhner et al. 2010)

```
ELN <- function(X, nkIdx){
    factor(ifelse(X$MissingCyto, NA,
                    ifelse(X$inv3_t3_3==1 | X$t_6_9==1 | X$minus5_5q==1 | X$mono17_17p_abn17p==1 | X$minus7==1 |
X$complex==1 | X$t_v_11==1,
                            "Adverse",
                            ifelse(X$t_15_17==1 | X$t_8_21==1 | X$inv16_t16_16==1 | ((X$CEBPA_bi==1 |  X$CEBPA_mo
no==1 | (X$NPM1==1 & X$FLT3_ITD==0)) & nkIdx),
                                    "Favorable",
                                    ifelse(nkIdx & (X$FLT3_ITD==1 | X$NPM1==0 & X$FLT3_ITD==0),
                                            "Inter-1", "Inter-2")))), levels=rev(c("Adverse","Inter-1","Inter-2",
"Favorable")))
}

table(clinicalData$M_Risk, ELN(dataFrame, nkIdx))
```

```
##
##             Favorable Inter-2 Inter-1 Adverse
##   Adverse           0       0       0     253
##   Favorable       457       6       6       4
##   Inter-1          17       0     400       0
##   Inter-2           1     251       7       9
```

Other models

```
tcgaRisk <- data.frame(
        #stdRisk = c(3,1,2)[tcgaClinical$C_Risk],
        ELN = as.numeric(ELN(tcgaDataImputed, tcgaNkIdx)),
        tree = predict(tree, newdata=tcgaDataImputed),
        rForest = predict(rForest, newdata = tcgaDataImputed, importance="none")$predicted,
        PINAos = tcgaPinaOs[,1],
        coxRFX = tcgaRiskRFXOs[,1],
        coxBIC = predict(coxBICOs, newdata=tcgaDataImputed),
        coxAIC = predict(coxAICOs, newdata=tcgaDataImputed),
        coxCPSS = tcgaRiskCPSSOs
)
```

Concordance of all models

```
tcgaConcordance <- sapply(tcgaRisk, function(x) {c <- survConcordance(tcgaSurvival ~ x); c(c$concordance, c$std.e
rr)})
tcgaConcordance
```

```
##                   ELN       tree    rForest     PINAos     coxRFX     coxBIC     coxAIC    coxCPSS
## concordant 0.57927774 0.64813236 0.68087415 0.66126782 0.70102294 0.69032858 0.68769374 0.69164600
## std(c-d)   0.02815496 0.02739215 0.02968944 0.02968936 0.02968944 0.02968944 0.02968944 0.02968944
```

```
o <- order(tcgaConcordance[1,])
barplot(tcgaConcordance[1,o], border=NA, col= set1[-6], las=2, xaxt="n", ylab="Concordance", ylim=c(0.5,0.75), xp
d=FALSE) -> b
segments(b,tcgaConcordance[1,o]-tcgaConcordance[2,o],b,tcgaConcordance[1,o]+tcgaConcordance[2,o])
rotatedLabel(b, rep(0.49,length(b)), colnames(tcgaConcordance)[o], srt=45)
```

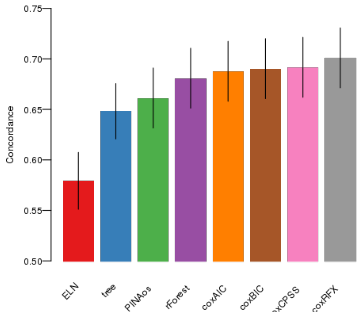

AUC of all models

```
library(survAUC)
library(survivalROC)
tcgaAUC <- sapply(tcgaRisk, function(x) AUC.uno(na.omit(os), tcgaSurvival[!is.na(x) & !is.na(tcgaSurvival)], scal
e(x)[!is.na(tcgaSurvival) &! is.na(x)], c(90,365,1000)/365)$auc)
tcgaAUCi <- sapply(tcgaRisk, function(x) AUC.uno(na.omit(os), tcgaSurvival[!is.na(x) & !is.na(tcgaSurvival)], sca
le(x)[!is.na(tcgaSurvival) &! is.na(x)], sort(na.omit(tcgaSurvival[,1])))$iauc)
o <- order(colMeans(tcgaAUC))
barplot(tcgaAUC[,o], border=1, col= rep(c("grey",set1[-6]),each=3), las=2, xaxt="n", ylab="AUC", beside=TRUE, den
sity=c(NA, 48,24), ylim=c(0.5,0.85), xpd=FALSE) -> b
legend("topleft", bty="n", c("3mo","1yr","3yr"), fill='black', density=c(NA, 48,24))
rotatedLabel(b[seq(3, length(b), 3)], rep(0.49,length(tcgaRisk)), names(tcgaRisk)[o], srt=45)
```

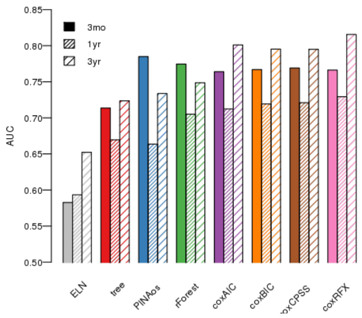

KM curves for four risk categories (quartiles)

```
risk <- cut(tcgaRiskRFXOs[,1], quantile(tcgaRiskRFXOs[,1]), labels=c("1st Q","2nd Q","3rd Q","4th Q"))
s <- survfit(tcgaSurvival ~ risk)
plot(s, col=set1[c(3,2,4,1)], mark=NA, xlab="Years", ylab="Survival")
legend("topright", bty="n", rownames(summary(s)$table), col=set1[c(3,2,4,1)], lty=1)
```

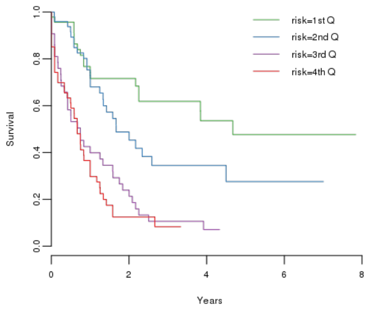

Distribution of risk v cytogenic categories

```
risk <- tcgaRiskRFXOs[,1] - mean(tcgaRiskRFXOs[,1])
x <- seq(from=-4,to=4, l=512)
r <- sapply(levels(tcgaClinical$C_Risk)[c(2,3,1)], function(r){
            i <- tcgaClinical$C_Risk==r
            d <- density(na.omit(risk[i]), from=-4,to=4)$y * mean(i, na.rm=TRUE)
        })
par(mar=c(4,4,3,4)+.1, bty="n")
plot(exp(x),rowSums(r), type='l', lty=0,xlab="Hazard", ylab="Prop. patients", log='x', ylim=c(0,.55))
for(i in 1:3)
    polygon(exp(c(x, rev(x))), c(rowSums(r[,1:i, drop=FALSE]), rev(rowSums(cbind(0,r)[,1:i, drop=FALSE]))), col=s
et1[c(3,2,1)][i], border=NA)

H0 <- basehaz(coxph(tcgaSurvival ~ risk), centered=TRUE)
hazardDist <- splinefun(H0$time, H0$hazard, method="monoH.FC")
invHazardDist <- splinefun(H0$hazard, H0$time, method="monoH.FC")
l <- c(0.1,.5,.9)#c(0.1,0.25,.5,.75,.9)
for(i in seq_along(l))
    lines(exp(x), pmax(0,invHazardDist(-log(l[i]) /exp(x) ))/10000*365, col='black', lty=c(2,1,2)[i])
axis(side=4, at=seq(0,.5,0.1), labels=seq(0,.5,.1)*10000*365)
mtext(side=4, "Time", line=2.5)
mtext(side=3, at = -log(l)/hazardDist(par("usr")[4]*10000*365), text=paste(100*l, "% survive", sep=""))
legend("topright", levels(tcgaClinical$C_Risk)[c(2,3,1)], fill=set1[c(3,2,1)], bty="n", title="M risk")
```

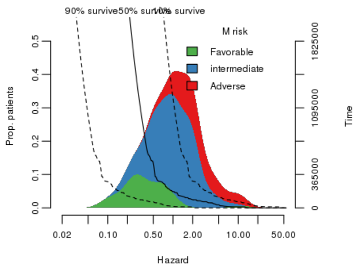

4.4.4 Multistage models

```
d <- tcgaData
d$transplantRel <- tcgaTpl[,"transplantRel"]
d$transplantCR1 <- tcgaTpl[,"transplantCR1"]
d$MissingCyto <- (tcgaClinical$karyotype == '[Not Available]' )+0
multiRfx5Tcga <- MultiRFX5(coxRFXNcdTD, coxRFXCrTD, coxRFXNrdTD, coxRFXRelTD, coxRFXPrdTD, d, tdPrmBaseline = tdP
rmBaseline, tdOsBaseline = tdOsBaseline, x=xmax)

mRFX3yr <- colSums(multiRfx5Tcga[3*365,1:3,])
survConcordance(tcgaSurvival ~ mRFX3yr)
```

```
## Call:
## survConcordance(formula = tcgaSurvival ~ mRFX3yr)
##
##   n=186 (14 observations deleted due to missingness)
## Concordance= 0.6878487 se= 0.02968944
## concordant discordant  tied.risk  tied.time   std(c-d)
##  8876.0000  4028.0000     0.0000   162.0000   766.2249
```

TCGA concordance time-dependent models

```
tcgaDataTdImputed <- as.data.frame(ImputeMissing(dataFrame[mainIdxOsTD], newX=tcgaData[mainIdxOsTD]))
tcgaRiskTD <- data.frame(
        coxBICTD = predict(coxBICOsTD, newdata=tcgaDataTdImputed),
        coxAICTD = predict(coxAICOsTD, newdata=tcgaDataTdImputed),
        coxRFXTD = PredictRiskMissing(coxRFXFitOsTDGGc, tcgaData)[,1],
        mRFX3yr = mRFX3yr
)

tcgaConcordanceTD <- sapply(tcgaRiskTD, function(x) unlist(survConcordance(tcgaSurvival ~ x)[c("concordance","std
.err")]))
```

Concordance

```
s <- rowMeans(colSums(aperm(multiRfx5Tcga[,1:3,],c(2,1,3))))
plot(survfit(tcgaSurvival ~ 1))
lines(seq(0,2000)/365.25/1.25,1-s)
```

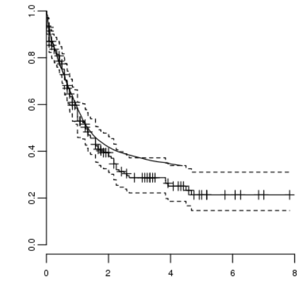

```
multiRfx5TcgaC <- sapply(seq(1,2000,10), function(i) survConcordance(tcgaSurvival ~  colSums(multiRfx5Tcga[i,1:3,
]))$concordance)
plot(seq(1,2000,10)/365.25,multiRfx5TcgaC, type='l', xlab="Years after diagnosis", ylab="Concordance", col=set1[1
], ylim=c(0.5, 0.73))
abline(h=tcgaConcordanceTD[1,"coxRFXTD"],col=set1[2])
tcgaEln <- ELN(tcgaDataImputed, tcgaNkIdx)
abline(h=survConcordance(tcgaSurvival ~ predict(coxph(os ~ ELN, data.frame(ELN=paste(clinicalData$M_Risk))), newd
ata=data.frame(ELN=tcgaEln)))$concordance,col=set1[3])
legend("bottomright",c("ELN","RFX OS","RFX Multistage"), col=set1[3:1], lty=1, bty="n")
```

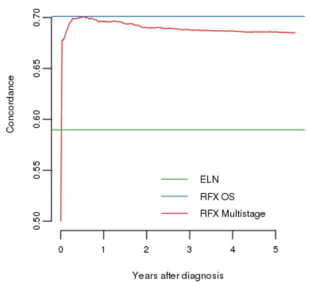

4.4.4.1 Figure 1C

Here we calculate the absolute prediction errors and coefficient of determination based on predictions derived from the AMLSG cohort and evaluated on the TCGA data set.

```
times <- seq(1,2000,10)
ss <- sapply(levels(eln),function(e) summary(survfit(os ~ 1, subset=eln==e), times=times)$surv)
ee <- sapply(times, function(t) ape(ss[times==t,paste(tcgaEln)], tcgaSurvival, t/365.25))

s0 <- summary(survfit(tcgaSurvival ~ 1), times=times/365.25)
s <- summary(survfit(os ~ 1), times=times)
c <- summary(survfit(coxRFXFitOsTDGGc), times=times)
e0 <- sapply(times, function(t) ape(s0$surv[times==t], tcgaSurvival, t/365.25))
e <- sapply(times, function(t) ape(s$surv[times==t], tcgaSurvival, t/365.25))
a <- sapply(times, function(t) ape(1-colSums(multiRfx5Tcga[t,1:3,]), tcgaSurvival, t/365.25))
b <- sapply(times, function(t) ape(c$surv[times==t] ^ exp(tcgaRiskTD$coxRFXTD - mean(coxRFXFitOsTDGGc$means %*% c
oxRFXFitOsTDGGc$coefficients)), tcgaSurvival, t/365.25))
for(i in 1:4){
    plot(times/365.25, e[i,], type='l', xlab="Time (yr)", ylab=rownames(a)[i], col=set1[9])
    lines(times/365.25, e0[i,], col=set1[9], lty=3)
    lines(times/365.25, a[i,], col=set1[1])
    lines(times/365.25, b[i,], col=set1[2])
    lines(times/365.25, ee[i,], col=set1[3])
    legend("bottomright",c("Kaplan-Meier (AML)", "Kaplan-Meier (TCGA)", "ELN","Multistage","RFX"), col=set1[c(9,9
,3,1:2)], lty=c(1,3,1,1,1), bty="n")
}
```

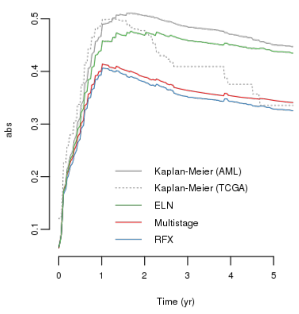

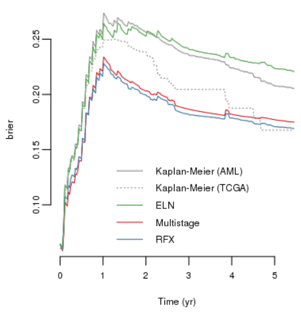

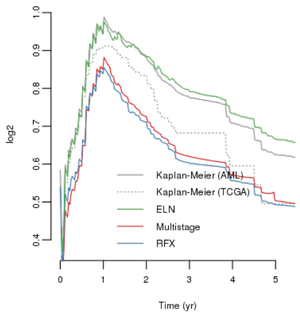

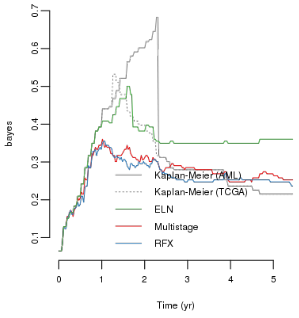


```
i <- 2; ## R2
plot(times/365.25, 1 - a[i,]/e[i,], type='l', xlab="Time (yr)", ylab="R2", col=set1[1], ylim=c(0,.3))
lines(times/365.25,  1 - b[i,]/e[i,], col=set1[2])
lines(times/365.25,  1 - ee[i,]/e[i,], col=set1[3])
legend("bottomright",c("ELN","Multistage","RFX OS"), col=set1[c(3,1:2)], lty=1, bty="n")
```

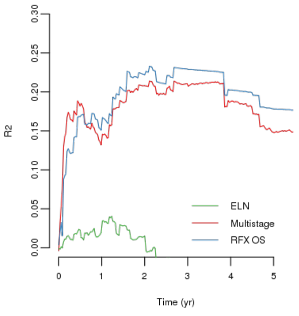

4.4.4.2 Figure 1A

Here we generate the overview shown in Figure 1A.

```r
library(abind)
par(mar=c(3,3.5,.5,.5),bty="n", mgp=c(2.5,.5,0), las=2,  lend=1, xpd=FALSE)
o <- c(1,7,2,3,4,6)
x <- rbind(allModelsCvC[o,], allModelsCvTdC[c("BIC","AIC","RFXgg","mRFX3yr"),])
col <- brewer.pal(4,"Set1")
#boxplot(t(x[o,]), notch=TRUE, ylab="Concordance", staplewex=0, lty=1, pch=16, xaxt="n", border="white", ylim=c(0
.5,0.75), boxwex=.5)
bplot <- function(x, at=1:ncol(x),..., ylim=range(x), xlab="", col="black", col.lines="grey"){
    y <- apply(x,2,fivenum)
    plot(at,y[3,], pch=NA, ..., ylim=ylim, xlab="", xaxt="n")
    segments(at,y[1,],at,y[5,], col=col.lines, lwd=2)
    segments(at,y[2,],at,y[4,], col=col.lines, lwd=5)
    points(at,y[3,], pch=15, col=col)
}
s <- .2 #space
a <- c(1:6, 7:10+.5)
bplot(t(x), at=a-1.5*s,ylab="Concordance", ylim=c(0.5,0.75), xlim=range(a)+c(-.5,.5))
abline(h=seq(.5,.75,.05), col="lightgrey")
par(xpd=NA)
t <- tcgaConcordance[,c(1,3,6,7,8,5)]
z <- abind(abind(TCGA=t, allModelsTrialC[,o,]), abind(TCGA=tcgaConcordanceTD, allModelsTrialTdC[,c("BIC","AIC","R
FXgg","mRFX3yr"),]), along=2)
m <- sapply(1:ncol(z),function(i){
            err <- 1 / sum(1/z[2,i,]^2)
            avg <- sum(z[1,i,] /z[2,i,]^2) * err
            c(avg,sqrt(err))})
#segments(1:6+s/2,m[1,]-m[2,],1:6+s/2,m[1,]+m[2,], lwd=2, col="#00000044")
#points(m[1,], pch=19, cex=1.5)
#segments(a-s/2,t[1,]-t[2,],a-s/2,t[1,]+t[2,], col=paste0(col[1],"FF"), lwd=2)
#points(a-s/2,t[1,], col=col[1], pch=16, cex=1)
i <- 0; for(n in dimnames(z)[[3]]) { i<-i+1;
    segments(a -s +s/2*i, z[1,,n] -  z[2,,n],a -s +s/2*i, z[1,,n]+ z[2,,n], col=mg14::colTrans(col[i]), lwd=2)
    points(a -s +s/2*i, z[1,,n], col=col[i], pch=16, cex=1)
}
segments(a -3/4*s, m[1,],a+s*5/4,m[1,], lwd=3)
mg14::rotatedLabel(a, labels= rownames(x))
legend("bottomright",
        c(
                "random CV 4/5 x100",
                paste0("TCGA, (n=",nrow(na.omit(tcgaSurvival)),")"),
                paste0(dimnames(allModelsTrialC)[[3]]," (n=",table(clinicalData$Study),")"),
                "average"),
        lty=c(1,1), bg="white", col=c("grey",col[1:4], "black"), pch=c(15,16,16,16,16,NA))
```

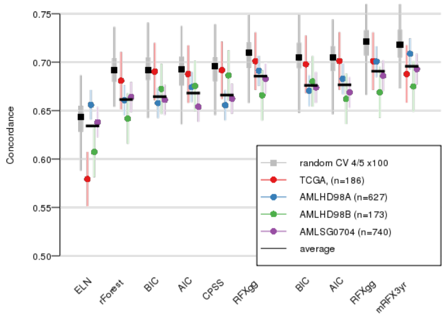

Short version

```r
par(mar=c(3,3.5,.5,.5),bty="n", mgp=c(2.5,.5,0), las=2,  lend=1, xpd=FALSE)
r <- sapply(as.data.frame(lapply(as.data.frame(t(apply(-x,2,rank, ties.method="random"))),factor, levels=1:nrow(x
))),table)
o <- 1:ncol(r) #order(apply(allModelsCvTdC[w,],1,median))
clr <- rev(brewer.pal(nrow(r),"PiYG"))#set1[c(3,2,4,1,5,7)]
barplot(r[,o]/replicates, col=clr[1:ncol(allModelsCvTdC)], ylab="Fraction", names.arg=rep("",ncol(r))) -> b
mg14::rotatedLabel(1:ncol(r), rep(par("usr")[3],ncol(x)), colnames(r)[o])
legend(par("usr")[1],1.5, fill=clr[1:nrow(r)], legend=1:nrow(r), bty="n", border=NA, horiz=TRUE, title="Rank")
```

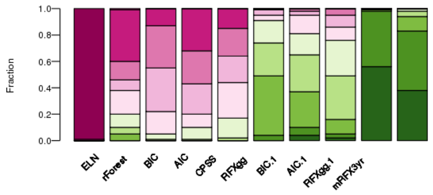

5 Simulations

We use simulations to assess different properties of our risk modelling approach.

5.1 Survival

Simulating survival times is useful, for example, to verify the consistency of our estimators and obtain empirical confidence intervals.

In the Cox proportional hazards model, the hazard is given by:

$$\lambda(t) = \lambda_0(t)\exp(uZ) = -\frac{dS(t)}{dt}\frac{1}{S(t)}. \tag{45}$$

On the transformed time-scale $\tau(t) = \int_0^t \lambda_0(t')dt'$, the hazard is constant and survival times are distributed exponentially. A strategy to model survival times according to the Cox proportional hazards model is therefore to draw unit survival times τ $\mathrm{Exp}(uZ)$ and to scale those according to τ^{-1}.

The observed survival times T_o are subject to censoring. The generative process can be thought of as $T_o = \min\{T, T_c\}$, where T_c is a censoring time and T the actual survival. This process may be simulated by estimating the cumulative distribution of censoring times $\hat{F}(T_c)$ using the Kaplan-Meier estimator and subsequently simulating censoring times $T_c = \hat{F}^{-1}(U); U \sim \mathrm{Unif}(0, 1)$.

The simulated times and events are then $T_o = \min\{T, T_c\}$ and the status is 1 if $T < T_c$ and 0 otherwise.

Hence our algorithm to simulate survival times can be summarised as follows:

1. KM estimate of baseline hazard `L_0(t)`
2. Monotonous spline interpolation and inversion `Linv_0(x)`
3. KM estimate of cumulative censoring distribution `Fcens(t)`
4. Monotonous spline interpolation of inverse `Finv`
5. Sample standardised exponential survival times with linear predictor `h` , transform using `T=Linv_0(rexp(h))`
6. Sample follow-up times `T_c=Finv(runif())`
7. Times: `pmin(T_c,T)` .
8. Status: `T < S`

This algorithm is implemented in `CoxHD::SimSurvNonp()` .

5.2 Interpolations

We use interpolations subsampling patients and genes to assess the influence of cohort size and breadth of genomic sequencing on our predictive performance.

5.2.1 Subsampling of genes

We take the following approach:

- Randomly take subsamples of $p' < p_{genes} = 58$ genetic covariates
- Extract gene:gene interaction terms with ≥ 8 occurrences
- Re-estimate the model using all patients.

We observed that the key determinant for the variance of the genetic log hazard is the average number of (genetic) drivers/patient

5.2.2 Subsampling of patients

For a given size `n` repeat `r` times:

- Draw a random subsample of patients of size `n` using `sample()`
- Train `CoxRFX` model
- Predict on the remaining patients and compute concordance

Compute the average concordance across all repetitions `r` . The number of repetitions `r` was chosen for each `n` such that the test size `1540 - n` was constant in order to achieve a similar error of the average concordance.

5.3 Extrapolations

Here we use a non-parametric approach to simulate data sets of larger cohorts to extrapolate influence of cohort size on prognostic accuracy. We also use a parametric approach to quantify the relation between the number of genes sequenced and model performance.

5.3.1 Patients

To extrapolate to larger cohort size we need to simulate new patients, distributed according to the empirical distribution. We observed that a simple resampling exaggerates the effect of interaction terms as particular constellations will be overrepresented. We therefore resampled patients and variables and used a multiple imputation package to impute the missing variables, noting that this will more likely generate non-duplicate data points that still satisfy the empirical distribution of the original data.

So we used the following steps:

- Set 10% of variables to NA; impute with `mice` (van Buuren and Groothuis-Oudshoorn 2011) using 10 chains.
- Sample from chains
- Sample effect sizes from RFX mean and variance parameters
- Keep covariates and interaction terms fixed

This protocol is implemented as `CoxHD::SimData()`

5.3.2 Genes

One observation made during [subsampling of genes][#subsampling-of-genes] was that the predicted variation of risk was a linear function of the average number of drivers/patient. Here we derive the theoretical groundwork supporting this observation.

Let Z be the set of genetic predictors and $u \sim N(\mu; \sigma)$ the distribution of effect sizes. Then the variation in log hazard is given by

$$
\begin{aligned}
Var[h] = Var[u^T Z] &= E[Var[u^T Z|Z]] + Var[E[u^T Z|Z]] && (46)\\
&= E[Z^T Var[u]Z] + \mu^2 Var[\textstyle\sum_i Z_i] && (47)\\
&= \sigma^2 E[\textstyle\sum_i Z_i^2] + \mu^2 Var[\textstyle\sum_i Z_i] && (48)\\
&= \sigma^2 E[\textstyle\sum_i Z_i] + \mu^2 Var[\textstyle\sum_i Z_i] \qquad Z_i^2 = Z_i \in \{0,1\} && (49)\\
&= \sigma^2 E[D] + \mu^2 Var[D] && (50)
\end{aligned}
$$

Where $D = \sum_i Z_i$ denotes the total number of drivers per patient. The latter term $\mu^2 Var[D]$ can be ignored as long as $D \approx 1$ and $|\mu| < 1$. Hence the variation in the log hazard increases proportionally to the mean number of drivers.

Note: These derivations hold for an additive model.

For interactions

$$E[Var[Z^T BZ|Z]] = \sigma^2 E[\sum ZZ^T] = \sigma^2 E[I] \qquad (51)$$

I being the number of interaction terms. $I < D(D-1)/2$.

5.3.2.1 TCGA

On TCGA data we can estimate the number of drivers by means of the sum of the excess of non-synonymous over synonymous mutations at each gene (Martincorena and others 2015). We use the total number of indels as an upper bound for the number of driver indels.

Using this approach we detect an average of 2.3 point mutations and 1.4 indels adding to 3.7 drivers per AML case when considering the entire exome. This compares to an average of 1.55 driver substitutions and 0.94 driver indels, with a total of 2.3 mutations (excluding multiple mutations in the same gene) in our cohort, having sequenced the 111 most prevalent driver genes.

It therefore appears that the variance explained due to the number of genes considered could be increased by approximately 50%.

5.4 Code

5.4.1 Interpolations

5.4.1.1 Figure 6A

Subsampling patients

```
library(survivalROC)
set.seed(42)
subsets <- seq(100,1500,100)
subsetPatients <- lapply(subsets, function(s){
            mclapply(1:ceiling(50000/(1540-s)), function(foo){
                        set.seed(s*foo)
                        trn <- 1:nrow(dataFrame) %in% sample(nrow(dataFrame), s)
                        tst <-  !trn
                        fit <- CoxRFX(dataFrameOsTD[tplSplitOs[trn], whichRFXOsTDGG], osTD[tplSplitOs[trn]], grou
ps[whichRFXOsTDGG], which.mu=mainGroups, nu = 0.1)
                        C <- survConcordance(osTD[tplSplitOs[tst]]~predict(fit, newdata=dataFrameOsTD[tplSplitOs[
tst], whichRFXOsTDGG]))
                        ROC <- survivalROC(Stime=os[!is.na(os) & tst,1], status=os[!is.na(os) & tst,2], marker =
predict(fit, newdata=dataFrame[tst, whichRFXOsTDGG]), predict.time = 850, method="KM", cut.values=seq(-5,5,0.1))
                        list(C, ROC, trn, tst, coef(fit))}, mc.cores=10)
        })
```

```
#pdf("subsetConcordance.pdf", 2.5,2.5, pointsize=8)
col1 <- colorRampPalette(set1[c(3,2,4,1,5)])(length(subsets))
plot(NA,NA, xlim=c(0,1),ylim=c(0,1), xlab="FPR",ylab="TPR")
abline(0,1, lty=3)
for(i in seq_along(subsets)){
    x <- sapply(subsetPatients[[i]], function(x) x[[2]]$FP)
    y <- sapply(subsetPatients[[i]], function(x) x[[2]]$TP)
    lines(rowMeans(x),rowMeans(y), col=col1[i], type="l")
}
```

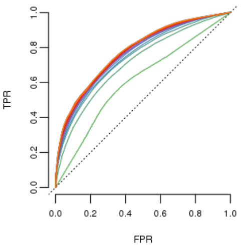

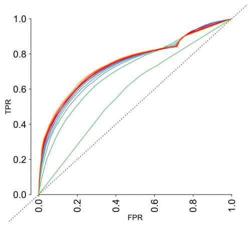

[outputs of the rerun (2021)]

```
#legend("bottomright", legend=rev(subsets), lty=1, col=col1[5:1], bty="n")


rangeplot2 <- function(x, y, col = 1, pch = 19, lty = 1, ylim=range(unlist(y)),...){
    plot(x, sapply(y, mean), col = col, pch=pch, ylim = ylim, ..., xaxt="n")
    points(jitter(unlist(sapply(seq_along(y), function(i) rep(x[i], length(y[[i]]))))),unlist(y),pch=1, col=unlis
t(sapply(seq_along(y), function(i) rep(col[i], length(y[[i]])))), cex=.2)
    lines(x, sapply(y, mean), lwd=2)
    lines(x, sapply(y, mean) + 2*sapply(y,  sd)/sqrt(sapply(y,length)))
    lines(x, sapply(y, mean) - 2*sapply(y,  sd)/sqrt(sapply(y,length)))
    axis(at = x, labels=x, side=1)
    #segments(x,apply(y,2,min),x,apply(y,2,max), col=col, lty = lty)
}

rangeplot3 <- function(x, y, col = 1, pch = 19, lty = 1, ylim=range(unlist(y)),...){
    plot(x, sapply(y, mean), col = col, pch=pch, ylim = ylim, ...)
    #points(jitter(unlist(sapply(seq_along(y), function(i) rep(x[i], length(y[[i]]))))),unlist(y),pch=1, col=unli
st(sapply(seq_along(y), function(i) rep(col[i], length(y[[i]])))), cex=.2)
    #lines(x, sapply(y, mean), lwd=2)
    s <- sapply(y,  sd)/sqrt(sapply(y,length))
    m <- sapply(y, mean)
    segments(x, m+s*2, x, m-s*2, col=col)
    #axis(at = x, labels=x, side=1)
    #segments(x,apply(y,2,min),x,apply(y,2,max), col=col, lty = lty)
}

rangeplot2(x=subsets, y = sapply(subsetPatients, function(x) sapply(x, function(y) y[[2]]$AUC)) , col=col1, xlab=
"Cohort", ylab="AUC", lty=1, ylim=c(0.7,0.85))
```

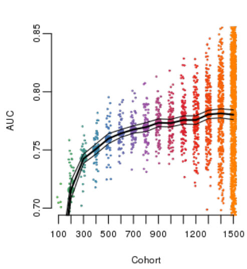

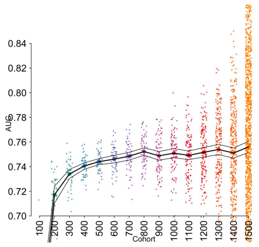

[outputs of the rerun (2021)]

```
rangeplot2(x=subsets, y = sapply(subsetPatients, function(x) sapply(x, function(y) y[[1]]$concordance)) , col=col
1, xlab="Cohort", ylab="Concordance", lty=1, ylim=c(0.65,.75), log='')
```

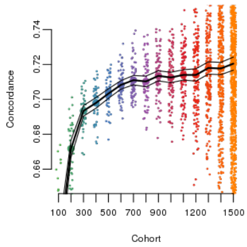

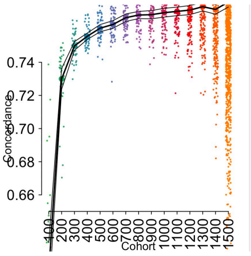

[outputs of the rerun (2021)]

```
rangeplot3(x=subsets, y = sapply(subsetPatients, function(x) sapply(x, function(y) y[[1]]$concordance)) , col=col
1, xlab="Cohort", ylab="Concordance", lty=1, ylim=c(0.67,.73), log='')
```

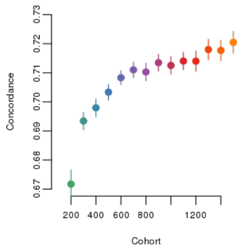

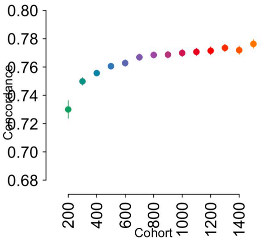

[outputs of the rerun (2021)]

```
#lines(x=subsets, y = concordanceFromVariance(sapply(subsetPatients, function(x) {
#                   mean(sapply(x, function(y) {
#                                       h <-  var(as.matrix(dataFrameOsTD[tplSplitOs[y[[3]]],whichRFXOsTDGG]) %*%
y[[5]])
#                                })) 
#               })) , col=1, xlab="Cohort", ylab="Concordance", ylim=c(0.65,.75))
#
```

5.4.1.2 Supplementary Figure S6A

Subsampling genes

```r
set.seed(42)
subsets <- seq(5,55,5)
genes <- names(whichRFXOsTDGG[groups=="Genetics"])
subsetGenes <- lapply(subsets, function(s){
            mclapply(1:100, function(foo){
                        g <- sample(genes, s)
                        ix <- !grepl(paste(g,collapse="|"), names(whichRFXOsTDGG))
                        trainIdx <- sample(1:nrow(dataFrame)%%5 +1 )!=1 ## sample 1/5
                        testIdx <-  !trainIdx
                        fit <- CoxRFX(dataFrameOsTD[tplSplitOs[trainIdx], whichRFXOsTDGG[ix]], osTD[tplSplitOs[trainIdx]], groups[whichRFXOsTDGG[ix]], which.mu=mainGroups, nu = 0.1)
                        C <- survConcordance(osTD[tplSplitOs[testIdx]]~predict(fit, newdata=dataFrameOsTD[tplSplitOs[testIdx], whichRFXOsTDGG[ix]]))
                        ROC <- survivalROC(Stime=os[!is.na(os) & testIdx,1], status=os[!is.na(os) & testIdx,2], marker = predict(fit, newdata=dataFrame[testIdx, whichRFXOsTDGG[ix]]), predict.time = 850, method="KM", cut.values=seq(-5,5,0.1))
                        fit <- CoxRFX(dataFrameOsTD[, whichRFXOsTDGG[ix]], osTD, groups[whichRFXOsTDGG[ix]], which.mu=mainGroups, nu = 0.1)
                        S <- cov(PartialRisk(fit))
                        list(C, ROC, S, trainIdx, testIdx, ix, mean(rowMeans(dataFrame[setdiff(genes,g)])))
                    }, mc.cores=10)
        })
```

```r
plot(sapply(subsetGenes, function(x) sapply(x, function(y) y[[7]]*sum(y[[6]][1:58]))), sapply(subsetGenes, function(x) sapply(x, function(y) {t <- try(sum(y[[3]][c("Genetics","GeneGene"),c("Genetics","GeneGene")])); ifelse(class(t)=="try-error",NA,t)})), xlab="Mean no. of drivers", ylab=expression(paste(Var,"[",h[g],"]")), xlim=c(0,3.8), ylim=c(0,.35), pch=16, col=c("#00000044"))
x <- c(0,3.7)
s <- coxRFXFitOsTDGGc$sigma2["Genetics"]
segments(c(2.3, 3.7), rep(par("usr")[3],2), c(2.3, 3.7), c(2.3, 3.7) * s, col="grey")
segments( rep(par("usr")[1],2),  c(2.3, 3.7) * s, c(2.3, 3.7), c(2.3, 3.7) * s, col="grey")
lines(x, x*s, col="red")
par(xpd=NA)
axis(at=c(2.3, 3.7), labels=c("111 genes", "TCGA (exome)"), tcl=0.5, side=1, mgp=c(-2.5,-2,0))
```

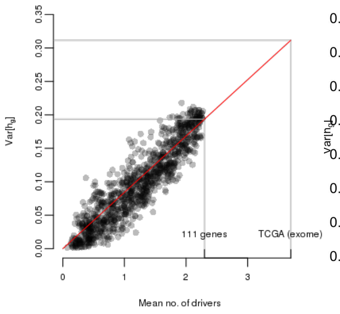

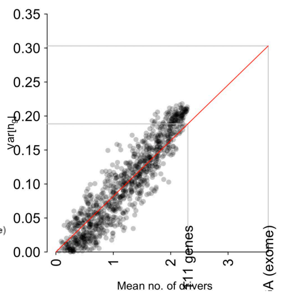

[outputs of the rerun (2021)]

5.4.2 Extrapolations

5.4.2.0.1 Generate new data

Simulate data using multiple imputation.

```r
set.seed(42)
SimDataNonp
```

```
## function (oldData, nData, percentMissing = 0.33, ...)
## {
##     require(mice)
##     oldData <- oldData[!apply(is.na(oldData), 1, all), ]
##     for (i in 1:nrow(oldData)) {
##         while (TRUE) {
##             naIdx <- sample(ncol(oldData), round(percentMissing *
##                 ncol(oldData)))
##             if (!all(1:ncol(oldData) %in% naIdx))
##                 break
##         }
##         oldData[i, naIdx] <- NA
##     }
##     m <- mice(as.data.frame(oldData), printFlag = FALSE, ...)
##     newData <- complete(m, action = "long")
##     newData <- newData[sample(nrow(newData), nData, replace = nrow(newData) <
##         nData), -2:-1]
##     return(newData)
## }
## <environment: namespace:CoxHD>
```

```r
d <- as.matrix(dataFrame[mainIdxOsTD])
w <- groups[mainIdxOsTD] %in% c("Genetics","Fusions","CNA")
d[,w][! as.matrix(d[,w]) %in% c(0,1)] <- NA # remove those imputed ones
simData <- SimDataNonp(d, nData = 10000, m=10)
```

```
## Loading required package: mice
## mice 2.22 2014-06-10
```

```
names(simData) <- names(dataFrame[mainIdxOsTD])
```

Merge into data.frame

```
set.seed(42)
g <- groups[mainIdxOsTD]
for(w in which(colSums(simData,na.rm=TRUE) == 0))
    simData[[w]] <- rbinom(nrow(simData),1,mean(dataFrame[mainIdxOsTD][,w]))
all(colSums(simData,na.rm=TRUE) != 0)
```

```
## [1] TRUE
```

```
> all(colSums(simData,na.rm=TRUE) != 0)
[1] FALSE
```

[outputs of the rerun (2021)]

```
simDataFrame <- cbind(simData,
        MakeInteractions(simData[,g=="Genetics"], simData[,g=="Genetics"])[,as.vector(upper.tri(matrix(0,ncol=sum
(g=="Genetics"), nrow=sum(g=="Genetics"))))])
for(n in unique(which(is.na(simDataFrame), arr.ind = TRUE)[,2]))
    simDataFrame[[n]] <- poorMansImpute(simDataFrame[[n]])
simDataFrame <- StandardizeMagnitude(simDataFrame)
simDataFrame <- simDataFrame[,colnames(simDataFrame)  %in% names(whichRFXOsTDGG) | colSums(simDataFrame)>=8]
simDataFrame$`NPM1:FLT3_ITD:DNMT3A` <- simDataFrame$NPM1 * simDataFrame$FLT3_ITD * simDataFrame$DNMT3A
dim(simDataFrame)
```

```
## [1] 10000   750
```

```
> dim(simDataFrame)
[1] 10000   733
```

[outputs of the rerun (2021)]

5.4.2.0.2 Basic simulations

```
set.seed(42)
simGroups <- factor(c(as.character(g), rep("GeneGene", ncol(simDataFrame)-length(g))))
names(simGroups) <- colnames(simDataFrame)
simCoef <- CoxHD:::SimCoef(coxRFXFitOsTDGGc, groups = simGroups)

simRisk <- as.matrix(simDataFrame[names(whichRFXOsTDGG)]) %*% simCoef[names(whichRFXOsTDGG)]
simSurv <- SimSurvNonp(simRisk, os)

survConcordance(simSurv ~ simRisk)
```

```
## Call:
## survConcordance(formula = simSurv ~ simRisk)
##
##   n= 10000
## Concordance= 0.7155292 se= 0.003360213
## concordant discordant  tied.risk  tied.time   std(c-d)
## 35433824.0 14087292.0      100.0        0.0   332803.7
```

```
> survConcordance(simSurv ~ simRisk)
$concordance
concordant
 0.7057051

$stats
concordant discordant  tied.risk  tied.time   std(c-d)
34989641.0 14591440.0       94.0        0.0   332830.8

$n
[1] 10000

$std.err
   std(c-d)
0.003356423
```

[outputs of the rerun (2021)]

5.4.2.0.3 Save output

```
save(coxRFXFitOsTDGGc, whichRFXOsTDGG, simDataFrame, simGroups, os, mainGroups, file="sim2Data.RData")
```

5.4.2.0.4 Simulation code

The following code is run on the farm

```
read_chunk('../code/Farmulations2.R', labels="farmulationsCode")
```

As we noted, codes in sections 5.4.2.0.4, 5.4.2.0.5, 5.4.2.1.1, 5.4.2.2.1, and 5.4.2.3.1 are also subject to an LSF environment for parallel computations. We were not able to tailor the entire R script to our own environments, as the computations are very intensive, and hence the modifications are prone to errors.

```
load("sim2Data.RData")
library(mg14)
library(CoxHD)
library(parallel)
nData <- c(100, 200, 500, 1000, 2000, 5000, 10000)
nJobs <- as.numeric(Sys.getenv("NJOBS"))

jobIndex <- as.numeric(Sys.getenv("LSB_JOBINDEX"))


set.seed(jobIndex)
simCoef <- CoxHD:::SimCoef(coxRFXFitOsTDGGc, groups = simGroups)
simRisk <- as.matrix(simDataFrame[names(whichRFXOsTDGG)]) %*% simCoef[names(whichRFXOsTDGG)]
simRisk <- simRisk - (colMeans(simDataFrame[names(whichRFXOsTDGG)]) %*% simCoef[names(whichRFXOsTDGG)])[1]
simSurv <- SimSurvNonp(simRisk, os)

for(n in nData){
    s <- if(n < 10000) sample(1:nrow(simDataFrame), n) else 1:10000
    f <- CoxRFX(simDataFrame[s,names(whichRFXOsTDGG)], simSurv[s], simGroups[names(whichRFXOsTDGG)], nu=1,which.m
u=mainGroups)
    f$X <- NULL
    assign(paste0("w",n), s)
    assign(paste0("fit",n),f)
}

rm(simDataFrame)

save.image(file=paste("simRFX/",Sys.getenv("LSB_JOBNAME"),"_",Sys.getenv("LSB_JOBINDEX"),".RData", sep=""))
```

5.4.2.0.5 Analysis

Read files

```
files <- dir("../code/simRFX", pattern="Farmulations\\[1-1000\\]*", full.names = TRUE)
tmp <- new.env()
load(files[1], envir = tmp)
```

5.4.2.1 Supplementary Figure S7B

5.4.2.1.1 P-values

Plot the P-values as a function of Npu^2.

```
w <- groups[whichRFXOsTDGG] %in% c("Genetics","Fusions","CNA", "GeneGene") ## Which groups
psi <- mean(os[,2]) ## Fraction of uncensored observations
plot(colSums(simDataFrame[names(whichRFXOsTDGG[w])]) * tmp$simCoef[whichRFXOsTDGG[w]]^2 , CoxHD:::WaldTest( tmp$f
it10000)$p[w], log="yx", pch=NA, xlab=expression(psi *N *p *beta^2), ylab="P-value", ylim=c(1e-50,1))
for(f in files[1:50]){
    load(f, envir = tmp)
    points(psi*colSums(simDataFrame[names(whichRFXOsTDGG[w])]) * tmp$simCoef[names(whichRFXOsTDGG[w])]^2 , CoxHD:
::WaldTest( tmp$fit10000)$p[w],  col=colGroups[as.character(groups)[whichRFXOsTDGG[w]]], pch=1, cex=.5)
    points(psi*colSums(simDataFrame[tmp$w1000, names(whichRFXOsTDGG[w])]) * tmp$simCoef[names(whichRFXOsTDGG[w])]
^2 , CoxHD:::WaldTest( tmp$fit1000)$p[w], col=colGroups[as.character(groups)[whichRFXOsTDGG[w]]], pch=2, cex=.5)
    if(tmp$fit100$iter[1] < 50) ## Exclude simulations without convergence
        points(psi*colSums(simDataFrame[tmp$w100, names(whichRFXOsTDGG[w])]) * tmp$simCoef[names(whichRFXOsTDGG[w
])]^2 ,CoxHD:::WaldTest( tmp$fit100)$p[w],  col=colGroups[as.character(groups)[whichRFXOsTDGG[w]]], pch=3, cex=.5
)
}
legend("bottomleft", lty=c(0,1),pch=c(1,NA), c("Simulations","Schoenfeld"), bty="n")
x <- 10^seq(-4,4,0.1)
lines(x, pnorm(sqrt(x), lower.tail = FALSE))
```

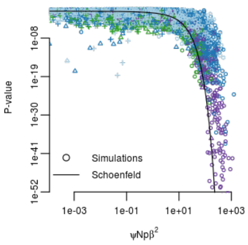

5.4.2.1.2 Power

The theoretical power according to Schoenfeld/Schmoor is given by (Schmoor, Sauerbrei, and Schumacher 2000):

```
power <- function(beta, N, p, psi=0.5, alpha=0.05){
    pnorm(sqrt(N*psi*beta^2*p*(1-p))-qnorm(1-alpha/2))
}
```

5.4.2.2 Supplementary Figure S7A

Plot for observed cases and overlay a few usual suspects

```
x <- seq(-2,2,0.01)
y <- 10^seq(-4,0,0.01)
colLevels <- colorRampPalette(brewer.pal(9, "Reds")[-(1:2)])(11)
g <- c("Fusions","CNA","Genetics","GeneGene")
xObs <- matrix(exp(rep(coxRFXFitOsTDGGc$mu[g], each=2) + c(-1,1) * rep(sqrt(coxRFXFitOsTDGGc$sigma2[g]),each=2)),
nrow=2) ## Mean log haz +/- sd
yObsQ <- sapply(split(colMeans(dataFrameOsTD[whichRFXOsTDGG]), groups[whichRFXOsTDGG]),quantile, c(0.05,0.5,0.95)
)[,g] ## 5,50,95% frequency quantiles

contour(outer(x,y,function(x,y) power(x,1540,y)), x=exp(x),y=y, log='xy', xlab="Hazard ratio", ylab="Mutation fre
quency", main="N=1540", col=colLevels)
```

```
## Warning in axis(side = side, at = at, labels = labels, ...): "log" is not a graphical parameter
```

```
## Warning in axis(side = side, at = at, labels = labels, ...): "log" is not a graphical parameter
```

```
## Warning in box(...): "log" is not a graphical parameter
```

```
rect(xObs[1,],yObsQ[1,],xObs[2,],yObsQ[3,], border = colGroups[c("Fusions","CNA","Genetics","GeneGene")])
#segments(exp(coxRFXFitOsTDGGc$mu[g]),yObsQ[1,],exp(coxRFXFitOsTDGGc$mu[g]),yObsQ[3,], col = colGroups[g])
#segments(xObs[1,],yObsQ[2,],xObs[2,],yObsQ[2,], col = colGroups[g])

effects <- c("NPM1","TP53","inv3_t3_3","t_15_17","inv16_t16_16","CEBPA_bi","FLT3_ITD","complex","NPM1:FLT3_ITD:DN
MT3A") ## A few interesting variables
points(exp(coef(coxRFXFitOsTDGGc)[effects]), colMeans(dataFrame[effects]), col=colGroups[as.character(groups[effe
cts])], pch=19)
text(labels=effects,exp(coef(coxRFXFitOsTDGGc)[effects]), colMeans(dataFrame[effects]), pos=ifelse(sign(coef(coxR
FXFitOsTDGGc)[effects])==1,4,2))
legend("bottom", lty=c(1,NA,NA,NA,NA,NA),pch=c(NA,19,22,22,22,22), c("Power","Selected variables", paste("Dist.",
g)), col=c(colLevels[10], "black", colGroups[g]), bty="n", ncol=2)
```

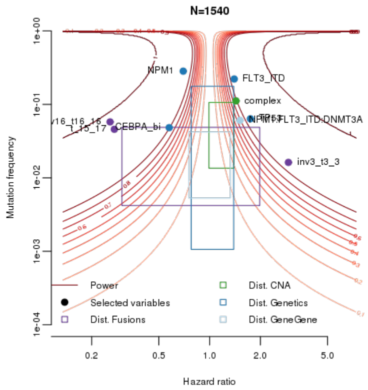

Compared to other cohort sizes

```
for(N in c(100,1000,10000)){
    contour(outer(x,y,function(x,y) power(x,N,y)), x=exp(x),y=y, log='xy', xlab="Hazard ratio", ylab="Mutation fr
equency", main=paste("N=",N,sep=""), col=colLevels, drawlabels=FALSE)
    rect(xObs[1,],yObsQ[1,],xObs[2,],yObsQ[3,], border = colGroups[g])
}
```

```
## Warning in axis(side = side, at = at, labels = labels, ...): "log" is not a graphical parameter
```

```
## Warning in axis(side = side, at = at, labels = labels, ...): "log" is not a graphical parameter
```

```
## Warning in box(...): "log" is not a graphical parameter
```

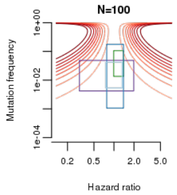


```
## Warning in axis(side = side, at = at, labels = labels, ...): "log" is not a graphical parameter
```

```
## Warning in axis(side = side, at = at, labels = labels, ...): "log" is not a graphical parameter
```

```
## Warning in box(...): "log" is not a graphical parameter
```

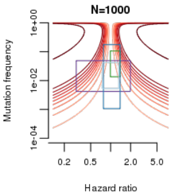


```
## Warning in axis(side = side, at = at, labels = labels, ...): "log" is not a graphical parameter
```

```
## Warning in axis(side = side, at = at, labels = labels, ...): "log" is not a graphical parameter
```

```
## Warning in box(...): "log" is not a graphical parameter
```

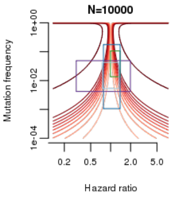

5.4.2.2.1 Concordance

```
C <- sapply(files[1:500], function(f){
            load(f)
            r <- c(sapply(tmp$nData, function(n){
                                survConcordance(SimSurvNonp(simRisk[get(paste0('w',n))], os)~get(paste0("fit",n))
$linear.predictors)$concordance
                            }),survConcordance(SimSurvNonp(simRisk, os)~simRisk)$concordance)
            names(r) <- c(nData,"Truth")
            return(r)
        })
boxplot(t(C), staplewex=0, pch=16, lty=1, ylab="", ylab="Concordance", xaxt="n")
rotatedLabel(labels=(sub(".concordant","", rownames(C))))
abline(h=CoxHD:::ConcordanceFromVariance(var(simRisk)))
```

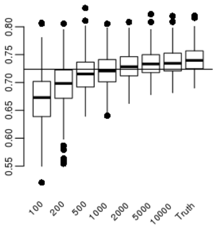

5.4.2.3 Figure 6C

5.4.2.3.1 Mean prediction error

```
load("../code/sim2Data.RData")
R <- sapply(files[1:100], function(f){
            load(f, envir=.GlobalEnv)
            r <- c(sapply(tmp$nData, function(n){
                                f <- get(paste0("fit",n))
                                assign("s", get(paste0("w",n)), envir=.GlobalEnv)
                                x <- as.matrix(simDataFrame[s, names(coef(f))])
                                h <- x %*% coef(f)
                                #z <- t(t(x)-colMeans(x))
                                #e <- rowSums(z %*% f$var2 * z)
                                #return(mean(e))
                                S <- try(survfit(f, newdata = as.data.frame(t(colMeans(x)))))
                                if(class(S)[1]=="try-error") return(NA)
                                hazardDist <- splinefun(H0$time, H0$hazard, method="monoH.FC")
                                sapply(seq(0,365*5, by=365/4), function(w){
                                            w <- which.min(abs(S$time-w))
                                            p <- S$surv[w]^exp(h - mean(h))
                                            q <- S$surv[w]^exp(simRisk[s])
                                            abs(p-q)})
                            }))
            names(r) <- c(nData)
            return(r)
        })
```

```
q <- sapply(1:nrow(R),function(i) apply(Reduce("rbind", R[i,]),2,quantile, c(0.025,0.25,0.5,0.75,0.975), na.rm=TR
UE), simplify="array")
contour(q[3,,],x=seq(0,365*5, by=365/4)/365, y=nData , log='y', xlab="Time (years)", ylab="Cohort size", las=1, x
lim=c(0,3))
```

```
## Warning in axis(side = side, at = at, labels = labels, ...): "log" is not a graphical parameter
```

```
## Warning in axis(side = side, at = at, labels = labels, ...): "log" is not a graphical parameter
```

```
## Warning in box(...): "log" is not a graphical parameter
```

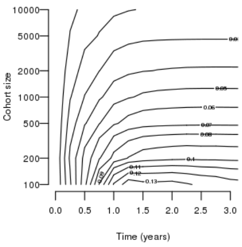


```
plot(nData,q[3,5,], log='xy', ylim=c(1e-2,1), type='l', ylab="Prediction error", lwd=2, xlab="Cohort size")
polygon(c(nData,rev(nData)), c(q[1,5,], rev(q[5,5,])), border=NA, col="#88888844")
polygon(c(nData,rev(nData)), c(q[2,5,], rev(q[4,5,])), border=NA, col="#88888844")
```

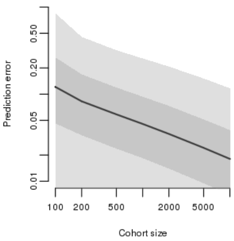


```
#rotatedLabel(labels=(sub(".concordant","", rownames(q))))
```

5.4.2.3.2 Cohort size

```
par(mar=c(3,3,1,1), bty='n', mgp=c(2,0.5,0))
cohort <- function(beta, p, psi=0.5, alpha=0.05, power=0.5){
    (qnorm(1-alpha/2) + qnorm(1-power) )^2 / (beta^2 * psi * p * (1-p))
}
x <- seq(-2,2, 0.01)
y <- 10^seq(-3,0, 0.01)
contour(outer(x,y,function(x,y) cohort(x,y, alpha=0.05/100)), x=exp(x),y=y, log='xy', xlab="Hazard ratio", ylab="
Mutation frequency",  col=colLevels, levels=c(10,20,50,100,200,500,1000,2000,5000,10000,20000))
```

```
## Warning in axis(side = side, at = at, labels = labels, ...): "log" is not a graphical parameter
```

```
## Warning in axis(side = side, at = at, labels = labels, ...): "log" is not a graphical parameter
```

```
## Warning in box(...): "log" is not a graphical parameter
```

```
rect(xObs[1,],yObsQ[1,],xObs[2,],yObsQ[3,], border = colGroups[c("Fusions","CNA","Genetics","GeneGene")])
effects <- c("NPM1","TP53","inv3_t3_3","t_15_17","inv16_t16_16","CEBPA_bi","FLT3_ITD","complex","NPM1:FLT3_ITD:DN
MT3A") ## A few interesting variables
points(exp(coef(coxRFXFitOsTDGGc)[effects]), colMeans(dataFrame[effects]), col=colGroups[as.character(groups[effe
cts])], pch=19)
text(labels=effects,exp(coef(coxRFXFitOsTDGGc)[effects]), colMeans(dataFrame[effects]), pos=ifelse(sign(coef(coxR
FXFitOsTDGGc)[effects])==1,4,2))
```

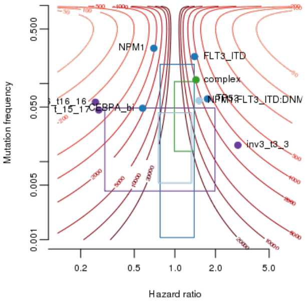


```
#legend("bottom", lty=c(1,NA,NA,NA,NA,NA),pch=c(NA,19,22,22,22,22), c("Power","Selected variables", paste("Dist."
, g)), col=c(colLevels[10], "black", colGroups[g]), bty="n", ncol=2)
```

5.4.2.4 Figure 6B

Number of cases needed

```
par(mar=c(3,5,1,1), bty='n', mgp=c(2.5,0.5,0))
cases <- function(beta, alpha=0.05, power=0.5, p = 1e-2, psi=0.5){
    (qnorm(1-alpha/2) + qnorm(1-power) )^2 / (beta^2 * (1-p) * psi)
}
x <- seq(-1,1,0.01)

x0 <- log(c(0.01,0.02,0.05,0.1,0.2,0.5,1)+1)
plot(exp(x), cases(x, alpha=5e-2), log='yx', type='l', xlab="Hazard ratio", ylab="Minimal number of cases", las=1
)
#lines(exp(x), cases(x, alpha=1e-2),  type='l', lty=2)
lines(exp(x), cases(x, alpha=1e-3),  type='l', lty=3)
segments(exp(x0), par("usr")[3],exp(x0),cases(x0, alpha=5e-2), col='grey')
segments(exp(x[1]), cases(x0, alpha=5e-2),exp(x0),cases(x0, alpha=5e-2), col='grey')
axis(side=2, at=cases(x0, alpha=5e-2), labels=exp(x0), tcl=.5, line=0, las=2, mgp=c(-2.5,-.5,0), hadj=0)
axis(side=1, at=c(seq(0.1,3,0.1)), labels=rep("",30), tcl=-.2, line=0, las=2)
axis(side=2, at=rep(c(1:10), 4) * 10^rep(1:4, each=10), labels=rep("",40), tcl=-.2, line=0, las=2)
legend("topright", legend=c("P < 0.05 *","P < 0.001 ***"), lty=c(1,3), bty="n")
```

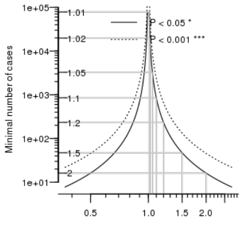

5.4.3 Multistage simulations

5.4.3.1 Simulation function

The following function simulates data from the 5-stage multistage RFX model

```
SimSurv5 <- function(coxRFXNcdTD, coxRFXCrTD, coxRFXNrdTD, coxRFXRelTD, coxRFXPrdTD, data, coxphOs, coxphPrs, cen
sInd, censCr, censRel){

    ## Step 1: Compute KM survival curves and log hazard
    getS <- function(coxRFX, data, max.x=5000) {
        if(!is.null(coxRFX$na.action)) coxRFX$Z <- coxRFX$Z[-coxRFX$na.action,]
        data <- as.matrix(data[,match(colnames(coxRFX$Z),colnames(data))])
        r <- PredictRiskMissing(coxRFX, data, var="var2")
        H0 <- basehaz(coxRFX, centered = FALSE)
        hazardDist <- splinefun(H0$time, H0$hazard, method="monoH.FC")
        invHazardDist <- splinefun(c(0,H0$hazard), c(0,H0$time), method="monoH.FC")
        x <- c(0:ceiling(max.x))
        S <- exp(-hazardDist(x))
        return(list(S=S, r=r, x=x, hazardDist=hazardDist, invHazardDist=invHazardDist, r0 = coxRFX$means %*% coef
(coxRFX)))
    }

    x <- 15000
    kmCr <- getS(coxRFX = coxRFXCrTD, data = data, max.x=max(x))
    kmEs <- getS(coxRFX = coxRFXNcdTD, data = data, max.x=max(x))
    kmCir <- getS(coxRFX = coxRFXRelTD, data = data, max.x=max(x))
    kmNrm <- getS(coxRFX = coxRFXNrdTD, data = data, max.x=max(x))
    kmPrs <- getS(coxRFX = coxRFXPrdTD, data = data, max.x=max(x))

    getCens <- function(surv, n){
        F <- survfit(surv~1)
        FCensInv <- splinefun(F$surv, F$time)
        censTimes <- FCensInv(runif(n,0,1)) ## Simulate censoring times
    }

    censIndTimes <- getCens(censInd, nrow(data))
    censCrTimes <- getCens(censCr, nrow(data))
    censRelTimes <- getCens(censRel, nrow(data))

    as.data.frame(t(sapply(1:nrow(data), function(i){
                                crTime <- edTime <- relTime <- nrdTime <- prdTime <- NA
                                status <- 1
                                crTime <- kmCr$invHazardDist(rexp(1, exp(kmCr$r[i,1])))
                                edTime <- kmEs$invHazardDist(rexp(1, exp(kmEs$r[i,1])))
                                firstTime <- pmin(edTime, crTime, censIndTimes[i])
                                if(firstTime==censIndTimes[i]){
                                    edTime <- firstTime
                                    status <- 0
                                    crTime <- NA
                                }
                                if(firstTime==edTime){
                                    crTime <- NA
                                }else{
                                    edTime <- NA
                                    rInd <- predict(coxphOs, newdata=data.frame(time0=crTime))
                                    relTime <- kmCir$invHazardDist(rexp(1, exp(kmCir$r[i,1] + rInd)))
                                    nrdTime <- kmNrm$invHazardDist(rexp(1, exp(kmNrm$r[i,1] + rInd)))
                                    secondTime <- pmin(relTime, nrdTime, censCrTimes[i])
                                    if(secondTime==censCrTimes[i]){
                                        nrdTime <- secondTime
                                        relTime <- NA
                                        status <- 0
                                    }
                                    if(secondTime==nrdTime){
                                        relTime <- NA
                                    }else{
                                        nrdTime <- NA
                                        rCr <- predict(coxphPrs, newdata=data.frame(time0=relTime))
                                        prdTime <- kmPrs$invHazardDist(rexp(1, exp(kmPrs$r[i,1] + rCr)))
                                        if(prdTime > censRelTimes[i]){
                                            prdTime <- min(prdTime, censRelTimes[i])
                                            status <- 0
                                        }
                                    }
                                }
                                times <- c(crTime=crTime, edTime=edTime, relTime=relTime+crTime, nrdTime=nrdTime+
crTime, prdTime=prdTime+crTime+relTime, status=status)
                                return(times)
                            }, simplify='array')))
}
```

5.4.3.2 Simulate outcomes

First prepare the data. Allograft indices:

```
alloIdx <- clinicalData$TPL_type %in% c("ALLO","FREMD") # only allografts
alloTimeRel <- clinicalData$TPL_date - clinicalData$Recurrence_date + .5 # +.5 to make > 0
alloTimeRel[!alloIdx | (clinicalData$TPL_date < clinicalData$Recurrence_date & !clinicalData$TPL_Phase %in% c("CR
1","RD"))] <- NA
```

Spline fitted transition probabilities.

```
coxphPrs <- coxph(Surv(time1, time2, status)~ pspline(time0, df=10), data=data.frame(prdData, time0=as.numeric(cl
inicalData$Recurrence_date-clinicalData$CR_date)[prdData$index]))
coxphOs <- coxph(Surv(time1,time2, status)~ pspline(time0, df=10), data=data.frame(osData, time0=pmin(500,cr[osDa
ta$index,1])))
```

Censoring distributions

```
censInd <- Surv(clinicalData$OS, 1-clinicalData$Status)[is.na(clinicalData$CR_date)]
censCr <- Surv(as.numeric(clinicalData$Date_LF - clinicalData$CR_date), 1-clinicalData$Status)[!is.na(clinicalDat
a$CR_date) & is.na(clinicalData$Recurrence_date)]
censRel <- Surv(as.numeric(clinicalData$Date_LF - clinicalData$Recurrence_date), 1-clinicalData$Status)[!is.na(cl
inicalData$CR_date) & !is.na(clinicalData$Recurrence_date)]
```

Simulate outcomes

```
set.seed(42)
simSurv5 <- SimSurv5(coxRFXNcdTD, coxRFXCrTD, coxRFXNrdTD, coxRFXRelTD, coxRFXPrdTD, data, coxphOs, coxphPrs, cen
sInd, censCr, censRel)

plot(survfit(Surv(apply(simSurv5[,1:5],1,max,na.rm=TRUE), simSurv5$status) ~ 1), xlim=c(0,5000))
lines(survfit(Surv(clinicalData$OS, clinicalData$Status) ~ 1), col='red')
```

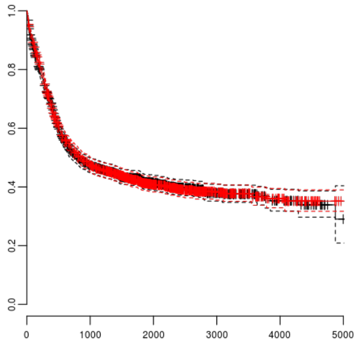

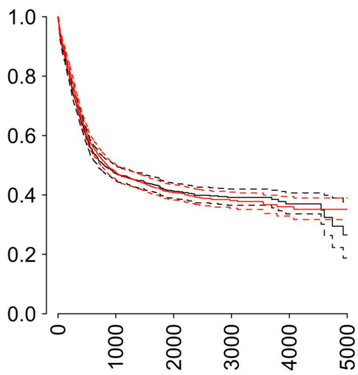

[outputs of the rerun (2021)]

5.4.3.3 Estimation based on simulated data

Now reestimate models in the scenario of a 10,000 patient cohort

```
set.seed(42)
simDataFrame$transplantCR1 <- rbinom(nrow(simDataFrame), 1, mean(data$transplantCR1))
simDataFrame$transplantRel <- rbinom(nrow(simDataFrame), 1, mean(data$transplantRel))
simDataSurv5 <- SimSurv5(coxRFXNcdTD, coxRFXCrTD, coxRFXNrdTD, coxRFXRelTD, coxRFXPrdTD, simDataFrame, coxphOs, c
oxphPrs, censInd, censCr, censRel)
```

Estimate RFX transition rates

```
simCr <- Surv(ifelse(!is.na(simDataSurv5$crTime), simDataSurv5$crTime, simDataSurv5$edTime), !is.na(simDataSurv5$
crTime))
simRfxCr <- CoxRFX(simDataFrame[names(crGroups)], simCr, groups=crGroups, which.mu = intersect(mainGroups, unique
(crGroups)))

simNcd <- Surv(ifelse(!is.na(simDataSurv5$edTime), simDataSurv5$edTime, simDataSurv5$crTime), simDataSurv5$status
& !is.na(simDataSurv5$edTime))
simRfxEs <- CoxRFX(simDataFrame[names(crGroups)], simNcd, groups=crGroups, which.mu = intersect(mainGroups, uniqu
e(crGroups)))

simRel <- Surv(ifelse(!is.na(simDataSurv5$relTime), simDataSurv5$relTime, simDataSurv5$nrdTime) - simDataSurv5$cr
Time, !is.na(simDataSurv5$relTime))
simRfxRel <- CoxRFX(simDataFrame[names(crGroups)], simRel, groups=crGroups, which.mu = intersect(mainGroups, uniq
ue(crGroups)))

simNrd <- Surv(ifelse(!is.na(simDataSurv5$relTime), simDataSurv5$relTime, simDataSurv5$nrdTime) - simDataSurv5$cr
Time, simDataSurv5$status & !is.na(simDataSurv5$nrdTime))
simRfxNrs <- CoxRFX(simDataFrame[names(crGroups)], simNrd, groups=crGroups, which.mu = intersect(mainGroups, uniq
ue(crGroups)))

simPrd <- Surv(simDataSurv5$prdTime - simDataSurv5$relTime, simDataSurv5$status)
simRfxPrs <- CoxRFX(simDataFrame[names(crGroups)], simPrd, groups=crGroups, which.mu = intersect(mainGroups, uniq
ue(crGroups)))

plot(coef(coxRFXCrTD),coef(simRfxCr))
```

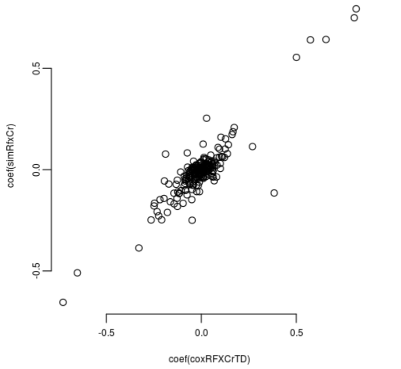

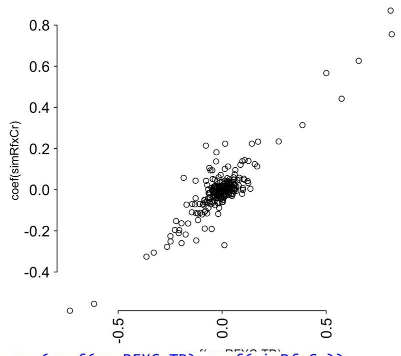

[outputs of the rerun (2021)]

```
> cor(coef(coxRFXCrTD),coef(simRfxCr))
[1] 0.9040149
```

```
cor(coef(coxRFXCrTD),coef(simRfxCr))
```

```
## [1] 0.906445
```

```
plot(coef(coxRFXNcdTD),coef(simRfxEs))
```

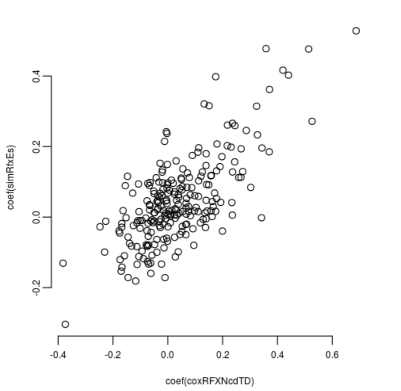

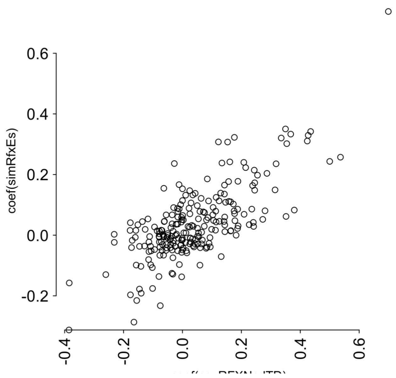

[outputs of the rerun (2021)]

```
> cor(coef(coxRFXNcdTD),coef(simRfxEs))
[1] 0.7389829
```

```
cor(coef(coxRFXNcdTD),coef(simRfxEs))
```

```
## [1] 0.7123169
```

```
plot(coef(coxRFXRelTD),coef(simRfxRel))
```

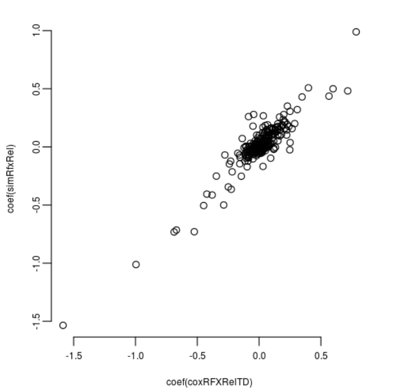

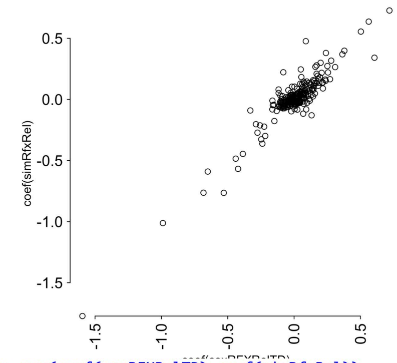

[outputs of the rerun (2021)]

```
> cor(coef(coxRFXRelTD),coef(simRfxRel))
[1] 0.9305525
```

```
cor(coef(coxRFXRelTD),coef(simRfxRel))
```

```
## [1] 0.9252248
```

```
plot(coef(coxRFXNrdTD),coef(simRfxNrs))
```

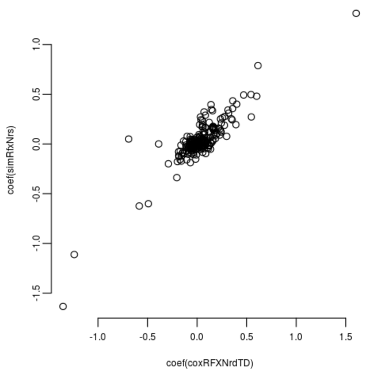

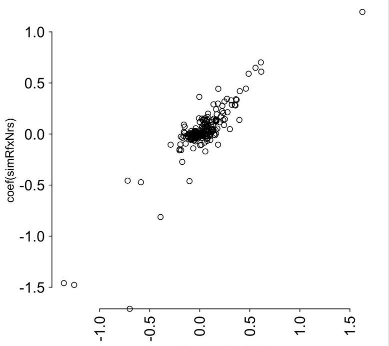

[outputs of the rerun (2021)]

```
> cor(coef(coxRFXNrdTD),coef(simRfxNrs))
[1] 0.8793642
```

```
cor(coef(coxRFXNrdTD),coef(simRfxNrs))
```

```
## [1] 0.8902996
```

```
plot(coef(coxRFXPrdTD),coef(simRfxPrs))
```

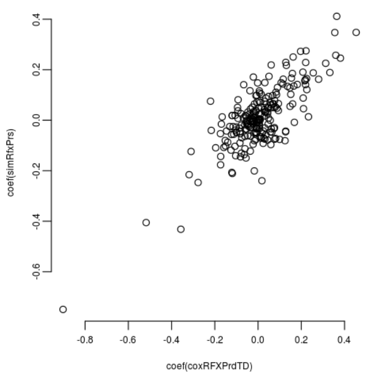

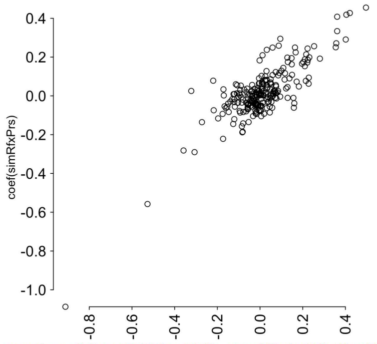

[outputs of the rerun (2021)]

```
> cor(coef(coxRFXPrdTD),coef(simRfxPrs))
[1] 0.8290316
```

```
cor(coef(coxRFXPrdTD),coef(simRfxPrs))
```

```
## [1] 0.7955286
```

Now compute the multistage RFX model

```
xmax <- 2000
xx <- 0:ceiling(xmax)
simPrs <- coxph(Surv(prdTime-relTime, status)~ pspline(relTime-crTime, df=10), data=simDataSurv5)
simPrsBaseline <- exp(predict(simPrs, newdata=data.frame(relTime=xx[-1], crTime=0))) ## Hazard (function of CR le
ngth)

simOs <- coxph(Surv(pmax(nrdTime, prdTime, na.rm=TRUE)-crTime, status)~ pspline(crTime, df=5), data=simDataSurv5)
simOsBaseline <- exp(predict(simOs, newdata=data.frame(crTime=xx[-1]))) ## Hazard (function of CR length)

simMultiRfx5 <- MultiRFX5(simRfxEs, simRfxCr, simRfxNrs, simRfxRel, simRfxPrs, data, tdPrmBaseline = simPrsBaseli
ne, tdOsBaseline = simOsBaseline, x=xmax)

plot(colSums(fiveStagePredicted[3*365,1:3,]), colSums(simMultiRfx5[3*365,1:3,]))
abline(0,1)
```

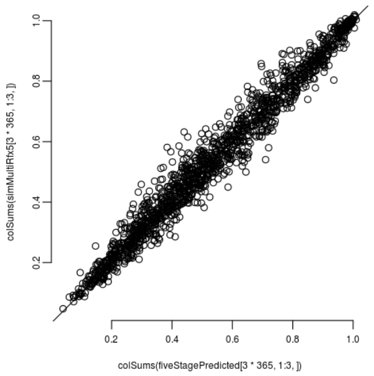

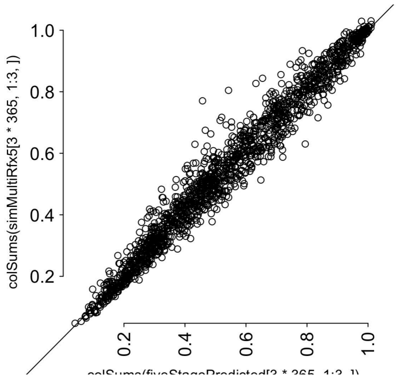

[outputs of the rerun (2021)]

Also compute the predicted benefit of allografts with confidence intervals

```
d <- osData[1:nrow(dataFrame),]
d$transplantCR1 <- 0
d$transplantRel <- 0
simMultiRFX3TplCi <- MultiRFX3TplCi(simRfxNrs, simRfxRel, simRfxPrs, data=d[,colnames(coxRFXNrdTD$Z)], x=3*365, n
Sim=200, prdData=prdData) ## others with 200
plot(multiRFX3TplCi["dCr1Rel","hat","os",] , simMultiRFX3TplCi["dCr1Rel","hat","os",], xlab="Benefit 1,540 patien
ts", ylab="Benefit 10,000 patients")
```

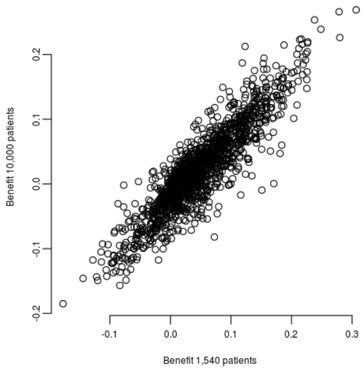

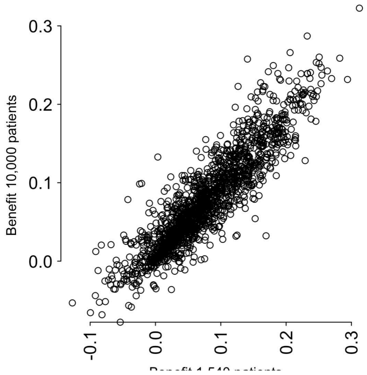

[outputs of the rerun (2021)]

```
plot(multiRFX3TplCi["dCr1Rel","upper","os",] - multiRFX3TplCi["dCr1Rel","lower","os",], simMultiRFX3TplCi["dCr1Re
l","upper","os",]-simMultiRFX3TplCi["dCr1Rel","lower","os",], xlab="CI width 1,540 patients", ylab="CI width 10,0
00 patients")
abline(0,0.5)
```

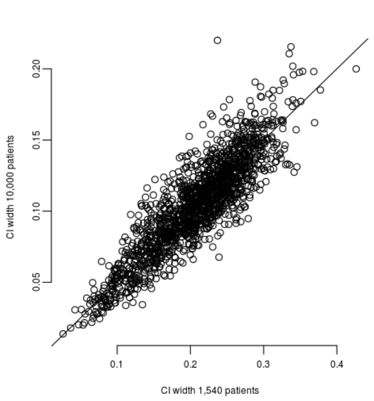

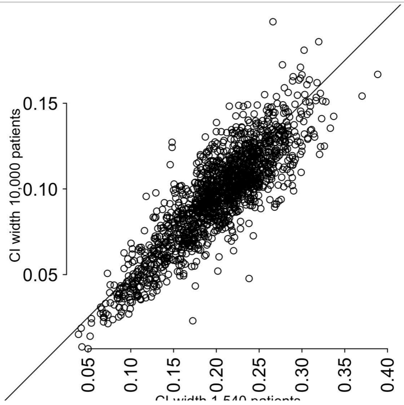

[outputs of the rerun (2021)]

5.4.3.4 HSCTs

Here we reassess the effect of HSCTs, also cosidering the magnitude of prediction errors in the current data set and based on extrapolated errors.

Benefit v number of allografts in CR1

```r
par(bty="L")
s <- clinicalData$AOD < 60 & !is.na(clinicalData$CR_date) & !clinicalData$TPL_Phase %in% c("PR1","RD1")
fAlloRelapse <- sum(prdData$transplantRel & s[!is.na(clinicalData$Recurrence_date)][prdData$index])/sum(relData$status & !relData$transplantCR1 & s[relData$index]) # fraction of patients that have received a salvage transplant
benefitAllo <- multiRFX3TplLoo[,"cr1"] - (fAlloRelapse*multiRFX3TplLoo[,"rel"] +(1-fAlloRelapse)*multiRFX3TplLoo[,"none"])
o <- order(-benefitAllo + ifelse(!s,NA,0), na.last=NA)
pRelapse <- 1+multiRFX3TplCiLoo[1:2,1,"aar",] - multiRFX3TplCiLoo[1:2,1,"rs",] ## Relapse probabilities
fRelapse <- sapply(seq_along(o), function(i) mean(c(pRelapse[2,o[1:i]], pRelapse[1,o[-(1:i)]]), na.rm=TRUE)) # Personalised

sIdeal <- sapply(seq_along(o), function(i) mean(c(multiRFX3TplLoo[o[1:i],"cr1"], (1-fAlloRelapse)*multiRFX3TplLoo[o[-(1:i)],"none"] + fAlloRelapse*multiRFX3TplLoo[o[-(1:i)],"rel"]), na.rm=TRUE))
x <- seq_along(sIdeal)/length(sIdeal)
plot(x + (1-x)*fRelapse*fAlloRelapse,sIdeal, type='l', xlab="Total fraction of allografts", ylab="Survival of eligible patients 3yrs after CR", col=set1[1], xaxs="i", yaxs="i", lty=3)

ci <- multiRFX3TplCiLoo["dCr1Rel","upper","os",]-multiRFX3TplCiLoo["dCr1Rel","lower","os",] # 1540 patients
sCi1540 <- rowMeans(sapply(1:10, function(foo){ set.seed(foo)
                    o <- order(-benefitAllo + ifelse(!s,NA,0) + rnorm(1540,sd=ci/4), na.last=NA)
                    s <- sapply(seq_along(o), function(i) mean(c(multiRFX3TplLoo[o[1:i],"cr1"], (1-fAlloRelapse)*multiRFX3TplLoo[o[-(1:i)],"none"] + fAlloRelapse*multiRFX3TplLoo[o[-(1:i)],"rel"]), na.rm=TRUE))
                }))
lines(x + (1-x)*fRelapse*fAlloRelapse, sCi1540, type='l',col=set1[1], lty=1)

simCi <- simMultiRFX3TplCi["dCr1Rel","upper","os",]-simMultiRFX3TplCi["dCr1Rel","lower","os",]

sCi10000 <- rowMeans(sapply(1:10, function(foo){ set.seed(foo)
                    o <- order(-benefitAllo + ifelse(!s,NA,0) + rnorm(1540,sd=simCi/4), na.last=NA)
                    s <- sapply(seq_along(o), function(i) mean(c(multiRFX3TplLoo[o[1:i],"cr1"], (1-fAlloRelapse)*multiRFX3TplLoo[o[-(1:i)],"none"] + fAlloRelapse*multiRFX3TplLoo[o[-(1:i)],"rel"]), na.rm=TRUE))
                }))
lines(x + (1-x)*fRelapse*fAlloRelapse, sCi10000, type='l',col=set1[1], lty=2)
p <- order(na.zero(c(1,4,2,3)[clinicalData$M_Risk])  + dataFrame$AOD_10/20 + ifelse(!s,NA,0), na.last=NA)
fRelapseEln <- sapply(seq_along(p), function(i) mean(c(pRelapse[2,p[1:i]], pRelapse[1,p[-(1:i)]]), na.rm=TRUE)) # ELN
sEln <- sapply(seq_along(p), function(i) mean(c(multiRFX3TplLoo[p[1:i],"cr1"], (1-fAlloRelapse)*multiRFX3TplLoo[p[-(1:i)],"none"] + fAlloRelapse*multiRFX3TplLoo[p[-(1:i)],"rel"]), na.rm=TRUE))
x <- seq_along(sEln)/length(sEln)

lines(x + (1-x)*fRelapseEln*fAlloRelapse,sEln, sEln, type='l', col=set1[2])
legend("bottomright", c("Personalised risk", "Idealised","10,000 patients","This cohort", "Standard risk","ELN and age"),  col=set1[c(NA,1,1,1,NA,2)],lty=c(NA,3,2,1,NA,1), bty="n", text.font=c(2,1,1,1,2,1))
```

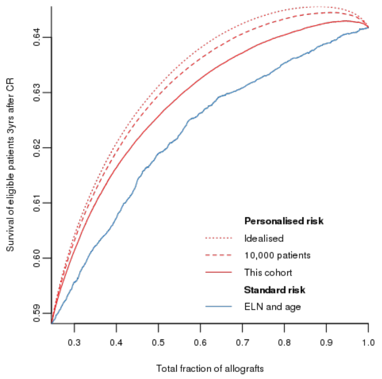

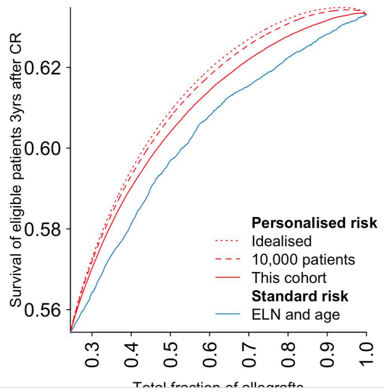

[outputs of the rerun (2021)]

Total numbers of transplants

```r
fAlloCR1 <- 0.3 ## Assume 30% allografts in CR1
i <- which(x > fAlloCR1)[1] - 1
c(`Knowlege bank`=(x + (1-x)*fRelapse*fAlloRelapse)[i], ELN=(x + (1-x)*fRelapseEln*fAlloRelapse)[i])
```

```
## Knowlege bank           ELN
##     0.4358391     0.4380818
```

```
> c(`Knowlege bank`=(x + (1-x)*fRelapse*fAlloRelapse)[i],
  ELN=(x + (1-x)*fRelapseEln*fAlloRelapse)[i])
Knowlege bank          ELN
    0.4351103    0.4380676
```

[outputs of the rerun (2021)]

Projected survival at 3yrs

```r
c(ELN=sEln[i], `This cohort`=sCi1540[i], `10000 patients` = sCi10000[i], Optimal=s[i])
```

```
##            ELN    This cohort 10000 patients        Optimal
##      0.6117393      0.6201484      0.6234637      1.0000000
```

Achieve same survival as ELN with the following number of allografts

```
> c(ELN=sEln[i], `This cohort`=sCi1540[i], `10000 patients` = sCi10000[i], Optimal=s[i])
          ELN    This cohort 10000 patients        Optimal
    0.5874657      0.5955505      0.5988003      1.0000000
```

[outputs of the rerun (2021)]

```
j <- c(`This cohort`=which(sCi1540 >= sEln[i])[1]-1, `10000 patients`=which(sCi10000 >= sEln[i])[1]-1, Optimal=wh
ich(sIdeal >= sEln[i])[1]-1)
fAlloCR1Pers <- (x + (1-x)*fRelapse*fAlloRelapse)[j]
names(fAlloCR1Pers) <- names(j)
fAlloCR1Pers
```

```
##    This cohort 10000 patients        Optimal
##      0.3634495      0.3463946      0.3392658
```

```
> fAlloCR1Pers
   This cohort 10000 patients        Optimal
     0.3824027      0.3684299      0.3625197
```
[outputs of the rerun (2021)]

6 Web tool

We were not able to rerun section 6 on our environments, as it performs clustering computations, and thus the modifications are prone to errors.

We have implemented the aformentioned multistage prediciton model as a shiny webserver.

6.1 Code

6.1.1 Data

The following data is saved for running the webtool:

```
save(coxRFXRelTD, coxRFXNrdTD, coxRFXPrdTD, coxRFXOsCR, coxRFXNcdTD, coxRFXCrTD, cr, nrdData, relData, prdData, o
sData, crGroups, data, clinicalData, file="../code/multistage/multistage.RData")
```

6.1.2 server.R

The server runs according to the following script

```
read_chunk('../code/multistage/server.R', labels="server.R")
```

```
library(shiny)
library(RColorBrewer)
library(CoxHD)
library(Rcpp)
load("multistage.RData", envir=globalenv())
cr <<- cr
set1 <- brewer.pal(8, "Set1")
pastel1 <- brewer.pal(8, "Pastel1")
s <- !crGroups %in% c("Nuisance","GeneGene")  & ! names(crGroups) %in% c("ATRA","VPA")
VARIABLES <- names(crGroups)[s]
rg <- c("Fusions"=5, "CNA"=4,"Genetics"=3, "Clinical"=7, "Demographics"=8, "Treatment"=6)
o <- order(rg[crGroups[s]],((coef(coxRFXPrdTD)^2/diag(coxRFXPrdTD$var2) + coef(coxRFXNrdTD)^2/diag(coxRFXNrdTD$va
r2) + coef(coxRFXRelTD)^2/diag(coxRFXRelTD$var2)) * apply(data[names(crGroups)], 2, var))[VARIABLES], decreasing=
TRUE)
VARIABLES <- VARIABLES[o]
NEWGRP <- c(0,diff(as.numeric(as.factor(crGroups))[s][o])) != 0
names(NEWGRP) <- VARIABLES
INTERACTIONS <- names(crGroups)[crGroups %in% "GeneGene"]
NUISANCE <- names(crGroups)[crGroups %in% "Nuisance" | names(crGroups) %in%  c("ATRA","VPA")]

SCALEFACTORS<- rep(1, length(VARIABLES))
names(SCALEFACTORS) <- VARIABLES
w <- crGroups[VARIABLES] %in% c("Demographics","Clinical")
r <- regexpr("(?<=_)[0-9]+$", VARIABLES[w], perl=TRUE)
SCALEFACTORS[w][r!=-1] <- as.numeric(regmatches(VARIABLES[w],r))

CATEGORIES <- sapply(VARIABLES, function(x){
            if(length(unique(data[,x])) <= 10){
                c <- min(data[,x]):max(data[,x])
                if(all(c %in% 0:1))
                    names(c) <- c("absent","present")
                else if(x =="gender")
                    names(c) <- c("male","female")
                return(c)
            }
            else
                NULL
        })

LABELS <- sapply(VARIABLES, function(x){
            r <- round(range(data[,x]*SCALEFACTORS[x], na.rm=TRUE),1)
            i <- paste0(" [",r[1],"-",r[2],"]")
            paste0(sub(paste0("_",SCALEFACTORS[x],"$"),"",x), ifelse(is.null(CATEGORIES[[x]]),i,""))
        })

LABELS["AOD_10"] <- sub("AOD", "Age at diagnosis (yr)", LABELS["AOD_10"])
LABELS["LDH_1000"] <- sub("LDH", "Lactic Acid Dehydrogenase (units/l)", LABELS["LDH_1000"])
LABELS["wbc_100"] <- sub("wbc", "White cell count (1e-9/l)", LABELS["wbc_100"])
LABELS["HB_10"] <- sub("HB", "Hemoglobin (g/l)", LABELS["HB_10"])
LABELS["BM_Blasts_100"] <- sub("BM_Blasts", "Bone marrow blasts (%)", LABELS["BM_Blasts_100"])
LABELS["PB_Blasts_100"] <- sub("PB_Blasts", "Peripheral blood blasts (%)", LABELS["PB_Blasts_100"])
LABELS["platelet_100"] <- sub("platelet", "Platelet count (1e-9/l)", LABELS["platelet_100"])
LABELS["VPA"] <- "VPA (Valproic acid)"
```

```
LABELS["transplantCR1"] <- "Allograft in CR1"
LABELS["transplantRel"] <- "Allograft after Relapse"
LABELS["gender"] <- "Gender"
LABELS <- sub("t_*([a-z,0-9]+)_([a-z,0-9]+)", "t(\\1;\\2)", LABELS)
LABELS[crGroups[VARIABLES] %in% c("Fusions","CNA")] <- gsub("_","/",LABELS[crGroups[VARIABLES] %in% c("Fusions","
CNA")])
LABELS <- sub("plus","+",LABELS)
LABELS <- sub("minus|^mono","-",LABELS)
LABELS <- sub("(_|/)*other"," (other)", LABELS)
LABELS <- sub("_([0-9a-zA-Z]+)"," (\\1)", LABELS)


COMPVAR <- list(`Allogeneic HSCT`=c(none="none", `in first CR`="transplantCR1", `after relapse`="transplantRel"),
`AML type`=c(primary='AML', secondary='sAML',tertiary='tAML',other='oAML')) ## Compound variables (factors)
COMPIDX <- numeric(length(VARIABLES))
names(COMPIDX) <- VARIABLES
COMPIDX[c("transplantRel","oAML")] <- 1 ## Index of last elements for display
VAR2COMP <- unlist(sapply(names(COMPVAR), function(n) rep(n, length(COMPVAR[[n]]))))
names(VAR2COMP) <- unlist(COMPVAR)

#* AOD: Age on diagnosis
#* LDH: Lactic Acid Dehydrogenase (units/l)
#* WBC: White cell count (1e-9/l),
#* HB: Hemoglobin (g/l),
#* BM_Blasts: Bone marrow blasts (%)
#* PB_Blasts: Peripheral blood blasts (%)


cppFunction('NumericVector computeHierarchicalSurvival(NumericVector x, NumericVector diffS0, NumericVector S1Sta
tic, NumericVector haz1TimeDep) {
                int xLen = x.size();
                double h;
                NumericVector overallSurvival(xLen);
                for(int i = 0; i < xLen; ++i) overallSurvival[i] = 1;
                    for(int j = 1; j < xLen; ++j){
                    if(diffS0[j-1] != 0){
                        h = haz1TimeDep[j-1];
                        for(int i = j; i < xLen; ++i){
                            overallSurvival[i] += diffS0[j-1] * (1-pow(S1Static[i-j], h));
                        }
                    }
                }
                return overallSurvival;
                }')

addGrid <- function(scale=1) {
    abline(h=seq(0,1,.2), lty=3)
    abline(v=seq(0,2000,365.25)/scale, lty=3)
}

# Define server logic required to generate and plot a random distribution
shinyServer(function(input, output) {
            getData <- reactive({
                        input$compute
                        isolate({
                                    l <- list()
                                    for(n in VARIABLES){
                                        if(!n %in% unlist(COMPVAR)){
                                            l[[n]] <- ifelse(input[[n]]=="NA",NA,as.numeric(input[[n]]))
                                            if(is.null(input[[n]])) l[[n]] <- NA
                                        }else{
                                            l[[n]] <- ifelse(input[[VAR2COMP[n]]]=="NA", NA, input[[VAR2COMP[n]]]
==n) + 0

                                            if(is.null(input[[VAR2COMP[n]]])) l[[n]] <- NA
                                        }
                                    }
                                    for(n in INTERACTIONS){
                                        s <- strsplit(n, ":")[[1]]
                                        l[[n]] <- l[[s[1]]] * l[[s[2]]]
                                    }
                                    for(n in NUISANCE)
                                        l[[n]] <- NA
                                    out <- do.call("data.frame",l)
                                    names(out) <- names(l)
                                    out[VARIABLES] <- out[VARIABLES]/SCALEFACTORS
                                    return(out)
                                })
                    })
            output$ui <- renderUI({
                        pdid <- input[["pdid"]]
                        if(is.null(pdid)) pdid <- "reset"
                        if( pdid=="reset"){
                            #cat("reset\n")
                            defaults <- data[1,]
                            defaults[] <- NA
                        }else
```

```
                        defaults <- data[pdid,]

                    defaults <- as.numeric(defaults)

                    ## Obfuscation
                    defaults <- signif(defaults * 20,1)/20

                    names(defaults) <- colnames(data)
                    defaults[VARIABLES] <- defaults[VARIABLES] * SCALEFACTORS
                    #cat(defaults,"\n")

                    makeMenu <- function(x) {
                        d <- defaults[x]
                        f <- if(x %in% unlist(COMPVAR)){
                                    if(!COMPIDX[x]) return(NULL)
                                    s <- defaults[COMPVAR[[VAR2COMP[x]]][-1]]
                                    w <- if(any(is.na(s))) 'N/A' else if(all(s==0)) 1 else if(any(!s %in% c(0
,1))) 'N/A' else which(s==1)+1
                                    c <- c(COMPVAR[[VAR2COMP[x]]], "N/A"="NA")
                                    radioButtons(VAR2COMP[x], label=VAR2COMP[x], choices=c, selected=c[w], in
line=FALSE)
                                }else if(crGroups[x] %in% c("Genetics","CNA","Fusions","Treatment")){
                                    if(!d %in% c(0,1)) d <- NA
                                    d <- paste(d)
                                    radioButtons(x, label=if(crGroups[x]=="Genetics") tags$em(LABELS[x]) else
LABELS[x], choices=c("present"= "1", "absent"="0", "N/A"="NA"), selected=d, inline=TRUE)
                                }else{
                                    r <- round(quantile(data[,x]*SCALEFACTORS[x], c(0.05,0.95), na.rm=TRUE),1
)
                                    if(is.null(CATEGORIES[[x]]))
                                        numericInput(inputId=x, label=LABELS[x], value=d, min=r[1], max=r[2],
step = if(round(min(data[,x]*SCALEFACTORS[x], na.rm=TRUE),1) %% 1 ==0) 1 else 0.1)
                                    else{
                                        if(!d %in% 0:10) d <- NA
                                        d <- paste(d)
                                        radioButtons(x, label=LABELS[x], choices=c(CATEGORIES[[x]],"N/A"="NA"
), selected=d, inline=TRUE)
                                    }
                                }
                        h <- if(NEWGRP[x]) list(tags$em(tags$b(crGroups[x]))) else NULL
                        list(h,f)}

                    list(wellPanel( list(
                                        tags$b("Clinical variables"),
                                        tags$hr(),
                                        tags$em(tags$b(crGroups[VARIABLES[1]])),
                                        lapply(VARIABLES[crGroups[VARIABLES] %in% c("Clinical","Demograph
ics")], makeMenu)
                                    ),
                                    style = "overflow-y:scroll; max-height: 400px; position:relative;"
                            ),
                            wellPanel(  list(
                                        tags$b("Genomic variables"),
                                        tags$hr(),
                                        #tags$em(tags$b(crGroups[VARIABLES[1]])),
                                        lapply(VARIABLES[crGroups[VARIABLES] %in% c("Genetics","Fusions",
"CNA")], makeMenu)
                                    ),
                                    style = "overflow-y:scroll; max-height: 400px; position:relative;"
                            ),
                            wellPanel(  list(
                                        tags$b("Treatment"),
                                        #tags$hr(),
                                        lapply(VARIABLES[crGroups[VARIABLES] == "Treatment"], makeMenu)
                                    ),
                                    style = "overflow-y:scroll; max-height: 400px; position:relative;"
                            )
                    )
                })
            x <- 0:2000

            computeIncidence <- function(coxRFX, r, x) {
                #r=PredictRiskMissing(coxRFX, data, var="var2")
                if(!is.null(coxRFX$na.action))
                    coxRFX$Z <- coxRFX$Z[-coxRFX$na.action,]
                #r <- PredictRiskMissing(coxRFX, data, var="var2")
                H0 <- basehaz(coxRFX, centered = FALSE)
                hazardDist <- splinefun(H0$time, H0$hazard, method="monoH.FC")
                lambda0 <- hazardDist(x)
                r0 <- coxRFX$means %*% coef(coxRFX)
                inc <- exp(-lambda0* exp(r[,1]))
                ciup2 <- exp(-lambda0*exp( rep(r[,1] + 2*sqrt(r[,2]) * c(1), each=length(x))))
                cilo2 <- exp(-lambda0*exp( rep(r[,1] + 2*sqrt(r[,2]) * c(-1), each=length(x))))
                ciup <- exp(-lambda0*exp( rep(r[,1] + sqrt(r[,2]) * c(1), each=length(x))))
                cilo <- exp(-lambda0*exp( rep(r[,1] + sqrt(r[,2]) * c(-1), each=length(x))))
                #p <- PartialRisk(coxRFX, dataImputed)
```

```
                return(list(inc=inc, r=r, x=x, hazardDist=hazardDist, r0 = r0, ciup=ciup, cilo=cilo, ciup2=ciup2,
cilo2=cilo2))
            }


            dataImputed <- reactive({
                        ImputeMissing(data[1:1540,], getData()[,colnames(data)])
                    })
            models <- c("Ncd","Cr","Rel","Nrd","Prd")
            riskMissing <- reactive({
                        sapply(models, function(m){
                                    fit <- get(paste("coxRFX",m,"TD", sep=""))
                                    if(!is.null(fit$na.action))
                                        fit$Z <- fit$Z[-fit$na.action,]
                                    PredictRiskMissing(fit, getData(),  var="var2")}, simplify = FALSE)})
            partialRiskMissing <- reactive({sapply(models, function(m){
                                    fit <- get(paste("coxRFX",m,"TD", sep=""))
                                    if(!is.null(fit$na.action))
                                        fit$Z <- fit$Z[-fit$na.action,]
                                    PartialRisk(fit, dataImputed())}, simplify = FALSE)})

            output$Tab <- renderDataTable({
                        x <- dataImputed()
                        data.frame(Covariate=colnames(x),signif(data.frame(Input=as.numeric(getData()[,colnames(d
ata)]), Imputed=as.numeric(x),
                                                        `Coef NCD`=coef(coxRFXNcdTD), `Value NCD`= as.numeric(x)*coef(cox
RFXNcdTD),
                                                        `Coef CR`=coef(coxRFXCrTD), `Value CR`= as.numeric(x)*coef(coxRFX
CrTD),
                                                        `Coef NRD`=coef(coxRFXNrdTD), `Value NRD`= as.numeric(x)*coef(cox
RFXNrdTD),
                                                        `Coef Rel`=coef(coxRFXRelTD), `Value Rel`= as.numeric(x)*coef(cox
RFXRelTD),
                                                        `Coef PRD`=coef(coxRFXPrdTD), `Value PRD`= as.numeric(x)*coef(cox
RFXPrdTD)),2))
                    })
            output$Risk <- renderDataTable({
                        t <- sapply(c("Ncd","Cr","Nrd","Rel","Prd"), function(m){
                                    r <- riskMissing()[[m]]
                                    x <- get(paste("coxRFX",m,"TD", sep=""))
                                    Z <- if(!is.null(x$na.action)) x$Z[-x$na.action,] else x$Z
                                    p <- PartialRisk(x, newZ= rbind(dataImputed(),colMeans(data[1:1540,])))
                                    p <- p[1,]-p[2,]
                                    #p <- p[-length(p)]
                                    c(round(p,3), `total`=round(r[1,1] - mean(Z %*% coef(x)),3),
                                          `sd`=round(sqrt(r[1,2]),3))
                                })
                        colnames(t) <- c("Death without CR (NCD)", "Complete remission (CR)", "Death without rela
pse (NRD)", "Relapse","Death after relapse (PRD)")
                        data.frame(Value=c(levels(coxRFXRelTD$groups),"total","s.d"), t, check.names=FALSE)
                    })
            ## Convolution approach to PRM
            survPredict <- function(surv){
                s <- survfit(surv~1)
                splinefun(s$time, s$surv, method="monoH.FC")
            }
            prsP <- survPredict(Surv(prdData$time1, prdData$time2, prdData$status))(x) # Baseline Prs (measured f
rom relapse)

            coxphPrs <- coxph(Surv(time1, time2, status)~ pspline(time0, df=10), data=data.frame(prdData, time0=a
s.numeric(clinicalData$Recurrence_date-clinicalData$CR_date)[prdData$index]))
            tdPrmBaseline <- exp(predict(coxphPrs, newdata=data.frame(time0=x[-1]))) ## Hazard (function of CR le
ngth)

            coxphOs <- coxph(Surv(time1,time2, status)~ pspline(time0, df=10), data=data.frame(osData, time0=pmin
(500,cr[osData$index,1])))
            tdOsBaseline <- exp(predict(coxphOs, newdata=data.frame(time0=x[-1])))   ## Hazard (function of induc
tion length), only for OS (could do CIR,NRM,PRS seperately)

            # CR adjustments to obtain absolute probabilities
            crAdjust <- function(x, y, time=x$x) {
                xadj <- .crAdjust(x$inc, y$inc, time)
            }

            .crAdjust <- function(inc1, inc2, time) {
                cumsum(c(1,diff(inc1) * splinefun(time, inc2)(time[-1])))
            }

            computeAbsoluteProbabilities <- reactive({
                        ## KM incidence of NCD and CR
                        kmNcd <- computeIncidence(coxRFX = coxRFXNcdTD, r = riskMissing()[["Ncd"]], x=x)
                        kmCr <- computeIncidence(coxRFX = coxRFXCrTD, r = riskMissing()[["Cr"]], x=x)

                        ## Correct KM estimate for competing risk
                        ncd <- crAdjust(x= kmNcd, time=x, y=kmCr) ## Correct KM estimate for competing risk
                        cr <- crAdjust(x= kmCr, time=x, y=kmNcd) ## Correct KM estimate for competing risk
```

```
                        ## KM incidence of Relapse and NRD
                        kmRel <- computeIncidence(coxRFX = coxRFXRelTD, r = riskMissing()[["Rel"]], x=x)
                        kmNrd <-  computeIncidence(coxRFX = coxRFXNrdTD, r = riskMissing()[["Nrd"]], x=x)

                        ## Correct KM estimate for competing risk
                        relCr <- crAdjust(x= kmRel, time=x, y=kmNrd) ## Correct KM estimate for competing risk
                        nrsCr <- crAdjust(x = kmNrd, time = x, y = kmRel)

                        ## KM incidence of PRS
                        kmPrs <-  computeIncidence(coxRFX = coxRFXPrdTD, r = riskMissing()[["Prd"]], x=x)

                        ## Outcome after Remission
                        rsCr <- computeHierarchicalSurvival(x = x, diffS0 = diff(relCr), S1Static = prsP, haz1Tim
eDep = tdPrmBaseline * exp(kmPrs$r[,1]-kmPrs$r0))
                        osCr <- 1-(1-nrsCr)-(1-rsCr)

                        ## Outcome from diagnosis
                        osDiag <- computeHierarchicalSurvival(x = x, diffS0 = diff(cr), S1Static = osCr, haz1Time
Dep = tdOsBaseline) - (1-ncd)
                        nrsDiag <- computeHierarchicalSurvival(x = x, diffS0 = diff(cr), S1Static = nrsCr, haz1Ti
meDep = tdOsBaseline)
                        rsDiag <- computeHierarchicalSurvival(x = x, diffS0 = diff(cr), S1Static = rsCr, haz1Time
Dep = tdOsBaseline)
                        relDiag <- computeHierarchicalSurvival(x = x, diffS0 = diff(cr), S1Static = relCr, haz1Ti
meDep = tdOsBaseline)


                        ## Confidence intervals
                        osLoDiag <- osUpDiag <- rep(NA, length(osDiag))

                        if("analytical" %in% input$ciType){
                            PlogP2 <- function(x) {(x * log(x))^2}
                            errOsCr <- kmNrd$r[,2] * PlogP2(kmNrd$inc) * (1-(1-kmRel$inc) * (1-kmPrs$inc))^2 + km
Rel$r[,2] * PlogP2(kmRel$inc) * (1-kmPrs$inc)^2* kmNrd$inc^2 +  kmPrs$r[,2] * PlogP2(kmPrs$inc) * (1-kmRel$inc)^2
* kmNrd$inc^2
                            errOsCr <- sqrt(errOsCr / PlogP2(osCr))
                            osUpCr <- osCr ^ exp(2* errOsCr)
                            osLoCr <- osCr ^ exp(-2*errOsCr)
                            #segments(z, osLo[z+1] ,z,osUp[z+1], col=1, lwd=2)
                        }
                        if("simulated" %in% input$ciType){
                            ## Simulate CI
                            nSim <- 200
                            osCrMc <- sapply(1:nSim, function(i){
                                        r <- exp(rnorm(5,0,sqrt(c(kmRel$r[,2],kmNrd$r[,2],kmPrs$r[,2], kmNcd$r[,2
], kmCr$r[,2]))))
                                        nrsCr <- .crAdjust(kmNrd$inc^r[2],  kmRel$inc^r[1], time=x) ## Correct KM
estimate for competing risk
                                        diffCir <- diff(kmRel$inc^r[1]) * kmNrd$inc[-1]^r[2] ## Correct KM estima
te for competing risk
                                        rsCr <- computeHierarchicalSurvival(x = x, diffS0 = diffCir, S1Static = p
rsP, haz1TimeDep = tdPrmBaseline * exp(kmPrs$r[,1]-kmPrs$r0+log(r[3])))
                                        osCr <- 1-(1-nrsCr)-(1-rsCr)

                                        cr <- .crAdjust(kmCr$inc^r[5], kmNcd$inc^r[4], time=x)
                                        ncd <- .crAdjust(kmNcd$inc^r[4], kmCr$inc^r[5], time=x)
                                        osDiag <- computeHierarchicalSurvival(x = x, diffS0 = diff(cr), S1Static
= osCr, haz1TimeDep = tdOsBaseline)

                                        return(cbind(osCr, osDiag - (1-ncd)))
                                    }, simplify="array")
                            osCrMcQ <- apply(osCrMc,1:2,quantile, c(0.025,0.975))
                            osLoCr <- osCrMcQ[1,,1]
                            osUpCr <- osCrMcQ[2,,1]
                            osLoDiag <- osCrMcQ[1,,2]
                            osUpDiag <- osCrMcQ[2,,2]
                        }

                        absolutePredictions=data.frame(x=x, cr=cr, ncd=ncd, osDiag=osDiag, nrsDiag=nrsDiag, rsDia
g=rsDiag, relDiag=relDiag, osCr=osCr, nrsCr=nrsCr, relCr=relCr, rsCr=rsCr, osUpCr=osUpCr, osLoCr=osLoCr, osLoDiag
=osLoDiag, osUpDiag=osUpDiag)

                        return(absolutePredictions)


                    })

            output$KM <- renderPlot({
                        par(bty="n", mar=c(3,3,2,1), mgp=c(2,0.5,0), tcl=-.25, xaxs="i", yaxs="i")
                        layout(matrix(1:3, ncol=3), widths=c(1,1,0.5))
                        par(cex=1)

                        with(computeAbsoluteProbabilities(),{

                        xLen <- length(x)
```

```
                        scale <- 365.25/12
                        xScaled <- x/scale

                        ## Plot probabilities
                        plot(xScaled, 1-(1-ncd)-(1-osDiag), type="l", xlab="Months from diagnosis", ylab="Probabi
lity", main="Outcome after diagnosis", ylim=c(0,1), lwd=3, lty=0)
                        y0 <- 1
                        y <- ncd
                        polygon(c(xScaled, xScaled[xLen]), c(y,y0), border=NA, col=pastel1[1])
                        y0 <- y0 - (1-ncd)
                        y <- y - (1-nrsDiag)
                        polygon(c(xScaled, rev(xScaled)), c(y, rev(y0))  , border=NA, col=pastel1[2])
                        y0 <- y0 - (1-nrsDiag)
                        y <- y - (1-rsDiag)
                        osDiag <- y
                        polygon(c(xScaled, rev(xScaled)), c(y, rev(y0)),  border=NA, col=pastel1[3])
                        y0 <- y0 - (1-rsDiag)
                        y <- y - (1-relDiag) + (1-rsDiag)
                        polygon(c(xScaled, rev(xScaled)), c(y, rev(y0)),  border=NA, col=pastel1[5])
                        polygon(c(xScaled, rev(xScaled)), c(cr - (1-ncd), rev(y)),  border=NA, col=pastel1[4])
                        polygon(c(xScaled, rev(xScaled)), c(cr - (1-ncd), rev(rep(0, length(xScaled)))),  border=
NA, col="#DDDDDD")
                        lines(xScaled, osDiag, lwd=3)

                        z <- round(c(365.25,3*365.25))
                        y <- (osDiag)[z+1]
                        points(z/scale,y, pch=16, col=1)
                        text(z/scale, y, labels=round(y,2), pos=1)
                        addGrid(scale)


                        ## CI
                        lines(xScaled, osUpDiag, col=1, lty=2)
                        lines(xScaled, osLoDiag, col=1, lty=2)

                        ## Plot outcome after remission
                        plot(NA,NA, xlab="Months from remission", ylab="Probability",  xlim=c(0,2000)/scale, ylim
=c(0,1), lty=2)
                        polygon(c(xScaled, xScaled[xLen]), c(nrsCr,1)  , border=NA, col=pastel1[2])
                        polygon(c(xScaled, rev(xScaled)), c(nrsCr, rev(osCr)),  border=NA, col=pastel1[3])
                        polygon(c(xScaled, rev(xScaled)), c(osCr, rev(1-(1-nrsCr)-(1-relCr))),  border=NA, col=pa
stel1[5])
                        polygon(c(xScaled, rev(xScaled)), c(1-(1-nrsCr)-(1-relCr), rep(0,length(xScaled))),  bord
er=NA, col=pastel1[4])
                        addGrid(scale)
                        lines(xScaled, osCr, col=1, lwd=3)
                        title("Outcome after remission")

                        y <- (osCr)[z+1]
                        points(z/scale,y, pch=16, col=1)
                        text(z/scale, y, labels=round(y,2), pos=1)

                        ## CI
                        lines(xScaled, osUpCr, col=1, lty=2)
                        lines(xScaled, osLoCr, col=1, lty=2)


                        par(mar=c(0,0,0,0))
                        plot(NA,NA, xlab="",ylab="", xaxt="n", yaxt="n", xlim=c(0,1), ylim=c(0,1))
                        legend(x=0,y=1, col=c(NA,NA,NA,NA,NA,NA,"black","black"), lty=c(NA,NA,NA,NA,NA,NA,1,4), f
ill=c(pastel1[c(1,2,3,5,4)],"#DDDDDD",NA,NA), border=c(1,1,1,1,1,1,NA,NA), lwd=c(NA,NA,NA,NA,NA,NA,3,1), y.inters
p = 1.5, c("Death without \nremission","Death without \nrelapse","Death after \nrelapse","Alive after \nrelapse",
"Alive in CR1", "Alive in \ninduction", "Overall survival", "95% C.I."), box.lwd = 0,  bg="#FFFFFF88", seg.len=1)

                        })
                    })

                    printMutations <- function(data) paste(LABELS[colnames(data)[which(data[1,]==1)]], collapse="
, ")

                    output$patientSummary <- renderText({
                                d <- getData()[, colnames(data)]
                                #x <- dataImputed()
                                bloodVariables <- c("BM_Blasts_100","PB_Blasts_100","wbc_100","LDH_1000","HB_10",
"platelet_100")
                                paste0( "Patient:           ", paste(c(na.omit(d[["AOD_10"]]*10), if(is.na(d[["AO
D_10"]])) "" else "yr old ", c(`1`="male",`2`='female',`NA`="")[paste(d[["gender"]])]), collapse=""), "\n",
                                        "Genomic variables: ", paste(printMutations(d[,crGroups %in% "Genetics",
drop=FALSE]),
                                                printMutations(d[,crGroups %in% "Fusions", drop=FALSE]),
                                                printMutations(d[,crGroups %in% "CNA", drop=FALSE]), collapse=";
"),"\n",
                                        "Blood counts:      ", paste((d[, bloodVariables] * SCALEFACTORS[bloodVar
iables])[!is.na(d[, bloodVariables])], sub("(.+) \\((.+)\\).+", "\\2 \\1",LABELS[bloodVariables][!is.na(d[, blood
Variables])], perl=TRUE), collapse=", "), "\n",
```

```
                                        "Treatment:         ", if(!is.na(d[,'transplantRel'])) if(d[,"transplantR
el"]) "HSCT after relapse" else if(d[,"transplantCR1"]) "HSCT in CR1" else if(d[,"transplantCR1"]==0 & d[,"transp
lantRel"]==0) "No HSCT")
                            })

                    round100 <- function(x){
                        y <- floor(x)
                        d <- x-y
                        o <- order(d, decreasing=TRUE)
                        i <- 1
                        while(sum(y) < 100){
                            y[o[i]] <- y[o[i]] + 1
                            i <- i+1
                        }
                        return(y)
                    }

                    printRisk <- function(x) paste0(paste0(c("|",rep("X", x)), collapse=""), " ",x, "%")

                    output$absoluteRiskDiag <- renderText({
                                r <- computeAbsoluteProbabilities()[round(3*365.25)+1,,drop=FALSE]
                                p <- c((1-r[,"ncd"]),(1-r[,"nrsDiag"]),(1-r[,"rsDiag"]),(1-r[,"relDiag"] - (1-r[,
"rsDiag"])),(r[,"osDiag"] -(1-r[,"relDiag"] - (1-r[,"rsDiag"])) - (r[,"cr"] - (1-r[,"ncd"]))  ),(r[,"cr"] - (1-r[
,"ncd"]) ))
                                p <- round100(p*100)
                                paste0( "Death without remission: ", printRisk(p[1]), "\n",
                                        "Death without relapse:   ", printRisk(p[2]),"\n",
                                        "Death after relapse:     ", printRisk(p[3]),"\n",
                                        "Alive after relapse:     ", printRisk(p[4]) ,"\n",
                                        "Alive in CR1:            ", printRisk(p[5]),"\n",
                                        "Alive without CR:        ", printRisk(p[6]),"\n")
                            })

                    output$absoluteRiskCr <- renderText({
                                r <- computeAbsoluteProbabilities()[round(3*365.25)+1,,drop=FALSE]
                                p <- c((1-r[,"nrsCr"]),(1-r[,"rsCr"]),(1-r[,"relCr"] - (1-r[,"rsCr"])),(r[,"osCr"
] -(1-r[,"relCr"] - (1-r[,"rsCr"])) ))
                                p <- round100(p*100)
                                paste0( "Death without relapse:   ", printRisk(p[1]),"\n",
                                        "Death after relapse:     ", printRisk(p[2]),"\n",
                                        "Alive after relapse:     ", printRisk(p[3]) ,"\n",
                                        "Alive in CR1:            ", printRisk(p[4]),"\n")
                            })

        })
```

6.1.3 ui.R

The shiny user interface is defined by the following script

```
read_chunk('../code/multistage/ui.R', labels="ui.R")
```

```r
library(shiny)
library(CoxHD)
load("multistage.RData", envir=globalenv())


# Define UI for application that plots random distributions
fluidPage(
	tags$head(
		includeHTML("www/popup.html")
	),
	includeHTML("www/disclaimer.html"),
	
	# Application title
	titlePanel("AML multistage predictions (beta)"),
	div(HTML('<h4 style="color:red;""> For research use only</h4>')),
	
	
	fluidRow(
		# Sidebar with a slider input for number of observations
		column(3,
			wellPanel(
				tags$b("Select sample"),
				tags$em(tags$small("Data may be rounded for privacy reasons.")),
				selectizeInput(inputId="pdid", label="", choices=c("reset",rownames(data)[order(a
s.numeric(gsub("[A-z]","", rownames(data))))]),  multiple=FALSE,
					options = list(maxOptions = nrow(data)+1,
						placeholder = 'Please select',
						onInitialize = I('function() { this.setValue(""); }'))),
				#tags$hr(),
				tags$br(),
				actionButton("compute", "Compute survival")
			),
				uiOutput("ui"),
			wellPanel(
				radioButtons("ciType", tags$b("Confidence intervals"), choices=c("analytical (fas
t, CR only)"="analytical","simulated (slow)"="simulated"), selected = "analytical"), ## CI type
				#tags$hr(),
				#div(HTML('<b><a href="help.html">Help</a></b>')),
				div(HTML('<b><a id="disclaimer">Disclaimer</a></b>')))
		),
		
		# Show a plot of the generated distribution
		column(8,
			tabsetPanel(
				tabPanel('Results',
					tags$h4("Patient summary"),
					textOutput(outputId="patientSummary", container=pre),
					tags$h4("Multistage probabilities"),
					plotOutput(outputId="KM",height="300px"),
					tags$h4("3-year post diagnosis risk estimates"),
					textOutput(outputId="absoluteRiskDiag", container=pre),
					tags$h4("3-year post CR risk estimates"),
					textOutput(outputId="absoluteRiskCr", container=pre)),
				
				tabPanel('Log hazard',
					dataTableOutput("Risk")),
				tabPanel("Coefficients",
					dataTableOutput("Tab")),
				tabPanel("Help",
					includeHTML("www/help.html"))
			))
	
	)
)
```

7 R session

This document was written entirely in R with markdown annotation. It was compiled with `knitr::spin()` (Xie 2015) and `pandoc` using the `rmarkdown` package (Allaire et al. 2015):

```r
rmarkdown::render("SupplementaryMethodsCode.R")
```

The total runtime is approximately 24h using 10 cores. This excludes the extrapolations, which were run on a a computing grid.

The packages and specifics of the R session are:

```r
library(devtools)
devtools::session_info()
```

```
## Session info ---------------------------------------------------------------------------------------------
----------
```

```
##  setting  value
##  version  R version 3.1.2 (2014-10-31)
##  system   x86_64, linux-gnu
##  ui       X11
##  language (EN)
##  collate  en_GB.UTF-8
##  tz       Europe/London
##  date     2016-09-07
```

```
## Packages -----------------------------------------------------------------------------------------------------
----------
```

```
##  package         * version date       source
##  abind           * 1.4-3   2015-03-13 CRAN (R 3.1.2)
##  BiocGenerics      0.12.1  2015-12-17 Bioconductor
##  car               2.0-25  2015-03-03 CRAN (R 3.1.2)
##  class             7.3-11  2014-07-21 CRAN (R 3.1.2)
##  codetools         0.2-9   2014-08-21 CRAN (R 3.1.2)
##  CoxHD           * 0.0.60  2016-02-09 Github (mg14/CoxHD@d4c307a)
##  devtools        * 1.9.1   2015-09-11 CRAN (R 3.1.2)
##  digest            0.6.8   2014-12-31 CRAN (R 3.1.2)
##  DT              * 0.1     2015-06-09 CRAN (R 3.1.2)
##  evaluate          0.8     2015-09-18 CRAN (R 3.1.2)
##  foreach         * 1.4.3   2015-10-13 CRAN (R 3.1.2)
##  formatR           1.2.1   2015-09-18 CRAN (R 3.1.2)
##  glmnet          * 2.0-2   2015-04-12 CRAN (R 3.1.2)
##  graph           * 1.44.1  2015-12-17 Bioconductor
##  HilbertVis      * 1.24.0  2015-12-17 Bioconductor
##  htmltools         0.2.6   2014-09-08 CRAN (R 3.1.2)
##  htmlwidgets     * 0.5     2015-06-21 CRAN (R 3.1.2)
##  iterators         1.0.8   2015-10-13 CRAN (R 3.1.2)
##  jsonlite          0.9.19  2015-11-28 CRAN (R 3.1.2)
##  KernSmooth        2.23-13 2014-09-14 CRAN (R 3.1.2)
##  knitr           * 1.11    2015-08-14 CRAN (R 3.1.2)
##  lattice         * 0.20-29 2014-04-04 CRAN (R 3.1.2)
##  lme4              1.1-10  2015-10-06 CRAN (R 3.1.2)
##  magrittr          1.5     2014-11-22 CRAN (R 3.1.2)
##  MASS            * 7.3-35  2014-09-30 CRAN (R 3.1.2)
##  Matrix          * 1.2-3   2015-11-28 CRAN (R 3.1.2)
##  MatrixModels      0.4-1   2015-08-22 CRAN (R 3.1.2)
##  memoise           0.2.1   2014-04-22 CRAN (R 3.1.2)
##  mg14            * 0.0.4   2015-12-17 Github (mg14/mg14@ae963a1)
##  mgcv              1.8-3   2014-08-29 CRAN (R 3.1.2)
##  minqa             1.2.4   2014-10-09 CRAN (R 3.1.2)
##  msSurv          * 1.2-2   2015-04-11 CRAN (R 3.1.2)
##  mvtnorm         * 1.0-5   2016-02-02 CRAN (R 3.1.2)
##  nlme              3.1-118 2014-10-07 CRAN (R 3.1.2)
##  nloptr            1.0.4   2014-08-04 CRAN (R 3.1.2)
##  nnet              7.3-8   2014-03-28 CRAN (R 3.1.2)
##  pbkrtest          0.4-4   2015-12-12 CRAN (R 3.1.2)
##  quantreg          5.19    2015-08-31 CRAN (R 3.1.2)
##  randomForestSRC * 2.0.0   2015-12-07 CRAN (R 3.1.2)
##  RColorBrewer    * 1.1-2   2014-12-07 CRAN (R 3.1.2)
##  Rcpp            * 0.12.2  2015-11-15 CRAN (R 3.1.2)
##  rj              * 2.0.4-2 2016-02-08 local
##  rj.gd             2.0.0-1 2016-02-08 local
##  rJava           * 0.9-7   2015-07-29 CRAN (R 3.1.2)
##  rmarkdown         0.9.2   2016-01-01 CRAN (R 3.1.2)
##  rpart           * 4.1-8   2014-03-28 CRAN (R 3.1.2)
##  rstudioapi        0.4.0   2015-12-09 CRAN (R 3.1.2)
##  SparseM           1.7     2015-08-15 CRAN (R 3.1.2)
##  stringi           1.0-1   2015-10-22 CRAN (R 3.1.2)
##  stringr           1.0.0   2015-04-30 CRAN (R 3.1.2)
##  survAUC         * 1.0-5   2012-09-04 CRAN (R 3.1.2)
##  survival        * 2.37-7  2014-01-22 CRAN (R 3.1.2)
##  survivalROC     * 1.0.3   2013-01-13 CRAN (R 3.1.2)
##  xlsx            * 0.5.7   2014-08-02 CRAN (R 3.1.2)
##  xlsxjars        * 0.6.1   2014-08-22 CRAN (R 3.1.2)
##  yaml              2.1.13  2014-06-12 CRAN (R 3.1.2)
```

```
sessionInfo()
```

```
## R version 3.1.2 (2014-10-31)
## Platform: x86_64-redhat-linux-gnu (64-bit)
##
## locale:
##  [1] LC_CTYPE=en_GB.UTF-8       LC_NUMERIC=C               LC_TIME=en_GB.UTF-8        LC_COLLATE=en_GB.UTF-8
##  [5] LC_MONETARY=en_GB.UTF-8    LC_MESSAGES=en_GB.UTF-8    LC_PAPER=en_GB.UTF-8       LC_NAME=C
##  [9] LC_ADDRESS=C               LC_TELEPHONE=C             LC_MEASUREMENT=en_GB.UTF-8 LC_IDENTIFICATION=C
##
## attached base packages:
##  [1] grid      parallel  splines   stats     graphics  grDevices utils     datasets  methods   base
##
## other attached packages:
##  [1] devtools_1.9.1        abind_1.4-3           survivalROC_1.0.3     survAUC_1.0-5         randomForestSRC_2
.0.0
##  [6] rpart_4.1-8           Rcpp_0.12.2           msSurv_1.2-2          graph_1.44.1          HilbertVis_1.24.0
## [11] lattice_0.20-29       xlsx_0.5.7            xlsxjars_0.6.1        rJava_0.9-7           htmlwidgets_0.5
## [16] DT_0.1                mg14_0.0.4            CoxHD_0.0.60          mvtnorm_1.0-5         RColorBrewer_1.1-
2
## [21] MASS_7.3-35           survival_2.37-7       glmnet_2.0-2          foreach_1.4.3         Matrix_1.2-3
## [26] knitr_1.11            rj_2.0.4-2
##
## loaded via a namespace (and not attached):
##  [1] BiocGenerics_0.12.1 car_2.0-25          class_7.3-11        codetools_0.2-9     digest_0.6.8
##  [6] evaluate_0.8        formatR_1.2.1       htmltools_0.2.6     iterators_1.0.8     jsonlite_0.9.19
## [11] KernSmooth_2.23-13  lme4_1.1-10         magrittr_1.5        MatrixModels_0.4-1  memoise_0.2.1
## [16] mgcv_1.8-3          minqa_1.2.4         nlme_3.1-118        nloptr_1.0.4        nnet_7.3-8
## [21] pbkrtest_0.4-4      quantreg_5.19       rj.gd_2.0.0-1       rmarkdown_0.9.2     rstudioapi_0.4.0
## [26] SparseM_1.7         stats4_3.1.2        stringi_1.0-1       stringr_1.0.0       tools_3.1.2
## [31] yaml_2.1.13
```

References

Allaire, JJ, Joe Cheng, Yihui Xie, Jonathan McPherson, Winston Chang, Jeff Allen, Hadley Wickham, and Rob Hyndman. 2015. *Rmarkdown: Dynamic Documents for R*. http://CRAN.R-project.org/package=rmarkdown (http://CRAN.R-project.org/package=rmarkdown).

Burnett, Alan K, Anthony Goldstone, Robert K Hills, Donald Milligan, Archie Prentice, John Yin, Keith Wheatley, Ann Hunter, and Nigel Russell. 2013. "Curability of Patients with Acute Myeloid Leukemia Who Did Not Undergo Transplantation in First Remission." *J Clin Oncol* 31 (10): 1293–1301. doi:10.1200/JCO.2011.40.5977 (http://dx.doi.org/10.1200/JCO.2011.40.5977).

Cancer Genome Atlas Research Network. 2013. "Genomic and Epigenomic Landscapes of Adult de Novo Acute Myeloid Leukemia." *N Engl J Med* 368 (22): 2059–74. doi:10.1056/NEJMoa1301689 (http://dx.doi.org/10.1056/NEJMoa1301689).

Döhner, Hartmut, Elihu H Estey, Sergio Amadori, Frederick R Appelbaum, Thomas Büchner, Alan K Burnett, Hervé Dombret, et al. 2010. "Diagnosis and Management of Acute Myeloid Leukemia in Adults: Recommendations from an International Expert Panel, on Behalf of the European LeukemiaNet." *Blood* 115 (3): 453–74. doi:10.1182/blood-2009-07-235358 (http://dx.doi.org/10.1182/blood-2009-07-235358).

Eddelbuettel, Dirk, and Romain Francois. 2011. "Rcpp: Seamless R and C++ Integration." *Journal of Statistical Software* 40 (8): 1–18. http://www.jstatsoft.org/v40/i08 (http://www.jstatsoft.org/v40/i08).

Ferguson, Nicole, Somnath Datta, and Guy Brock. 2012. "MsSurv: An R Package for Nonparametric Estimation of Multistate Models." *Journal of Statistical Software* 50 (14): 1–24. http://www.jstatsoft.org/v50/i14 (http://www.jstatsoft.org/v50/i14).

Friedman, Jerome, Trevor Hastie, and Rob Tibshirani. 2010. "Regularization Paths for Generalized Linear Models via Coordinate Descent." *J Stat Softw* 33 (1): 1–22.

Gerds, Thomas A, and Martin Schumacher. 2006. "Consistent Estimation of the Expected Brier Score in General Survival Models with Right-Censored Event Times." *Biom J* 48 (6): 1029–40.

Gönen, Mithat, and Glenn Heller. 2005. "Concordance Probability and Discriminatory Power in Proportional Hazards Regression." *Biometrika* 92 (4): 965–70. doi:10.1093/biomet/92.4.965 (http://dx.doi.org/10.1093/biomet/92.4.965).

Gray, Robert J. 1992. "Flexible Methods for Analyzing Survival Data Using Splines, with Applications to Breast Cancer Prognosis." *Journal of the American Statistical Association* 87 (420): 942–51. doi:10.1080/01621459.1992.10476248 (http://dx.doi.org/10.1080/01621459.1992.10476248).

Harrell, F E, Jr, K L Lee, and D B Mark. 1996. "Multivariable Prognostic Models: Issues in Developing Models, Evaluating Assumptions and Adequacy, and Measuring and Reducing Errors." *Stat Med* 15 (4): 361–87. doi:10.1002/(SICI)1097-0258(19960229)15:4<361::AID-SIM168>3.0.CO;2-4 (http://dx.doi.org/10.1002/(SICI)1097-0258(19960229)15:4<361::AID-SIM168>3.0.CO;2-4).

Ishwaran, Hemant, Udaya B Kogalur, Eugene H Blackstone, and Michael S Lauer. 2008. "Random Survival Forests." *The Annals of Applied Statistics* 2 (3). JSTOR: 841–60. doi:10.1214/08-AOAS169 (http://dx.doi.org/10.1214/08-AOAS169).

Martincorena, Iñigo, and others. 2015. "Manuscript in Preparation."

Meinshausen, Nicolai, and Peter Bühlmann. 2010. "Stability Selection." *Journal of the Royal Statistical Society: Series B (Statistical Methodology)* 72 (4). Blackwell Publishing Ltd: 417–73. doi:10.1111/j.1467-9868.2010.00740.x (http://dx.doi.org/10.1111/j.1467-9868.2010.00740.x).

Nagelkerke, N. J. D. 1991. "A Note on a General Definition of the Coefficient of Determination." *Biometrika* 78 (3): 691–92. doi:10.1093/biomet/78.3.691 (http://dx.doi.org/10.1093/biomet/78.3.691).

O'Quigley, John, Ronghui Xu, and Janez Stare. 2005. "Explained Randomness in Proportional Hazards Models." *Stat Med* 24 (3): 479–89. doi:10.1002/sim.1946 (http://dx.doi.org/10.1002/sim.1946).

Papaemmanuil, Elli, Moritz Gerstung, Luca Malcovati, Sudhir Tauro, Gunes Gundem, Peter Van Loo, Chris J Yoon, et al. 2013. "Clinical and Biological Implications of Driver Mutations in Myelodysplastic Syndromes." *Blood* 122 (22): 3616–27. doi:10.1182/blood-2013-08-518886 (http://dx.doi.org/10.1182/blood-2013-08-518886).

Papaemmanuil, Elli, Moritz Gerstung, Richard Schlenk, and others. 2015. *Submitted Manuscript*.

Pastore, Friederike, Annika Dufour, Tobias Benthaus, Klaus H Metzeler, Kati S Maharry, Stephanie Schneider, Bianka Ksienzyk, et al. 2014. “Combined Molecular and Clinical Prognostic Index for Relapse and Survival in Cytogenetically Normal Acute Myeloid Leukemia.” *J Clin Oncol*, Apr. doi:10.1200/JCO.2013.52.3480 (http://dx.doi.org/10.1200/JCO.2013.52.3480).

Perperoglou, Aris. 2014. “Cox Models with Dynamic Ridge Penalties on Time-Varying Effects of the Covariates.” *Stat Med* 33 (1): 170–80. doi:10.1002/sim.5921 (http://dx.doi.org/10.1002/sim.5921).

Potapov, Sergej, Werner Adler, and Matthias Schmid. 2012. *SurvAUC: Estimators of Prediction Accuracy for Time-to-Event Data.* http://CRAN.R-project.org/package=survAUC (http://CRAN.R-project.org/package=survAUC).

Schall, Robert. 1991. “Estimation in Generalized Linear Models with Random Effects.” *Biometrika* 78 (4): 719–27. doi:10.1093/biomet/78.4.719 (http://dx.doi.org/10.1093/biomet/78.4.719).

Schmoor, C, W Sauerbrei, and M Schumacher. 2000. “Sample Size Considerations for the Evaluation of Prognostic Factors in Survival Analysis.” *Stat Med* 19 (4): 441–52.

Shah, Rajen D., and Richard J. Samworth. 2013. “Variable Selection with Error Control: Another Look at Stability Selection.” *Journal of the Royal Statistical Society: Series B (Statistical Methodology)* 75 (1). Blackwell Publishing Ltd: 55–80. doi:10.1111/j.1467-9868.2011.01034.x (http://dx.doi.org/10.1111/j.1467-9868.2011.01034.x).

Simon, Noah, Jerome H. Friedman, Trevor Hastie, and Rob Tibshirani. 2011. “Regularization Paths for Cox’s Proportional Hazards Model via Coordinate Descent.” *Journal of Statistical Software* 39 (5): 1–13. http://www.jstatsoft.org/v39/i05 (http://www.jstatsoft.org/v39/i05).

Therneau, Terry. 2012. *Coxme: Mixed Effects Cox Models.* http://CRAN.R-project.org/package=coxme (http://CRAN.R-project.org/package=coxme).

———. 2014. *A Package for Survival Analysis in S*.

Therneau, Terry M, Patricia M Grambsch, and V. Shane Pankratz. 2003. “Penalized Survival Models and Frailty.” *Journal of Computational and Graphical Statistics* 12 (1): 156–75. doi:10.1198/1061860031365 (http://dx.doi.org/10.1198/1061860031365).

Therneau, Terry, Beth Atkinson, and Brian Ripley. 2014. *Rpart: Recursive Partitioning and Regression Trees*. http://CRAN.R-project.org/package=rpart (http://CRAN.R-project.org/package=rpart).

Uno, Hajime, Tianxi Cai, Lu Tian, and L. J Wei. 2007. “Evaluating Prediction Rules for T-Year Survivors with Censored Regression Models.” *Journal of the American Statistical Association* 102 (478): 527–37. doi:10.1198/016214507000000149 (http://dx.doi.org/10.1198/016214507000000149).

van Buuren, Stef, and Karin Groothuis-Oudshoorn. 2011. “Mice: Multivariate Imputation by Chained Equations in R.” *Journal of Statistical Software* 45 (3): 1–67. http://www.jstatsoft.org/v45/i03/ (http://www.jstatsoft.org/v45/i03/).

Wood, Simon N. 2013. “A Simple Test for Random Effects in Regression Models.” *Biometrika* 100 (4): 1005–10. doi:10.1093/biomet/ast038 (http://dx.doi.org/10.1093/biomet/ast038).

Xie, Yihui. 2015. *Knitr: A General-Purpose Package for Dynamic Report Generation in R*. http://CRAN.R-project.org/package=knitr (http://CRAN.R-project.org/package=knitr).
